# Pathway and network analysis of more than 2500 whole cancer genomes

**DOI:** 10.1038/s41467-020-14367-0

**Published:** 2020-02-05

**Authors:** Matthew A. Reyna, David Haan, Marta Paczkowska, Lieven P. C. Verbeke, Miguel Vazquez, Abdullah Kahraman, Sergio Pulido-Tamayo, Jonathan Barenboim, Lina Wadi, Priyanka Dhingra, Raunak Shrestha, Gad Getz, Michael S. Lawrence, Jakob Skou Pedersen, Mark A. Rubin, David A. Wheeler, Søren Brunak, Jose M. G. Izarzugaza, Ekta Khurana, Kathleen Marchal, Christian von Mering, S. Cenk Sahinalp, Alfonso Valencia, PCAWG Drivers and Functional Interpretation Working Group, Federico Abascal, Samirkumar B. Amin, Gary D. Bader, Pratiti Bandopadhayay, Rameen Beroukhim, Johanna Bertl, Keith A. Boroevich, John Busanovich, Peter J. Campbell, Joana Carlevaro-Fita, Dimple Chakravarty, Calvin Wing Yiu Chan, Ken Chen, Jung Kyoon Choi, Jordi Deu-Pons, Klev Diamanti, Lars Feuerbach, J. Lynn Fink, Nuno A. Fonseca, Joan Frigola, Carlo Gambacorti-Passerini, Dale W. Garsed, Mark Gerstein, Qianyun Guo, Ivo G. Gut, Mark P Hamilton, Nicholas J. Haradhvala, Arif O. Harmanci, Mohamed Helmy, Carl Herrmann, Julian M. Hess, Asger Hobolth, Ermin Hodzic, Chen Hong, Henrik Hornshøj, Keren Isaev, Rory Johnson, Todd A. Johnson, Malene Juul, Randi Istrup Juul, Andre Kahles, Manolis Kellis, Jaegil Kim, Jong K. Kim, Youngwook Kim, Jan Komorowski, Jan O. Korbel, Sushant Kumar, Andrés Lanzós, Erik Larsson, Donghoon Lee, Kjong-Van Lehmann, Shantao Li, Xiaotong Li, Ziao Lin, Eric Minwei Liu, Lucas Lochovsky, Shaoke Lou, Tobias Madsen, Iñigo Martincorena, Alexander Martinez-Fundichely, Yosef E. Maruvka, Patrick D. McGillivray, William Meyerson, Ferran Muiños, Loris Mularoni, Hidewaki Nakagawa, Morten Muhlig Nielsen, Keunchil Park, Kiejung Park, Tirso Pons, Iker Reyes-Salazar, Esther Rheinbay, Carlota Rubio-Perez, Gordon Saksena, Leonidas Salichos, Chris Sander, Steven E. Schumacher, Mark Shackleton, Ofer Shapira, Ciyue Shen, Shimin Shuai, Nikos Sidiropoulos, Lina Sieverling, Nasa Sinnott-Armstrong, Lincoln D. Stein, David Tamborero, Grace Tiao, Tatsuhiko Tsunoda, Husen M. Umer, Liis Uusküla-Reimand, Claes Wadelius, Jiayin Wang, Jonathan Warrell, Sebastian M. Waszak, Joachim Weischenfeldt, Guanming Wu, Jun Yu, Jing Zhang, Xuanping Zhang, Yan Zhang, Zhongming Zhao, Lihua Zou, Jüri Reimand, Joshua M. Stuart, Benjamin J. Raphael, PCAWG Consortium, Lauri A. Aaltonen, Federico Abascal, Adam Abeshouse, Hiroyuki Aburatani, David J. Adams, Nishant Agrawal, Keun Soo Ahn, Sung-Min Ahn, Hiroshi Aikata, Rehan Akbani, Kadir C. Akdemir, Hikmat Al-Ahmadie, Sultan T. Al-Sedairy, Fatima Al-Shahrour, Malik Alawi, Monique Albert, Kenneth Aldape, Ludmil B. Alexandrov, Adrian Ally, Kathryn Alsop, Eva G. Alvarez, Fernanda Amary, Samirkumar B. Amin, Brice Aminou, Ole Ammerpohl, Matthew J. Anderson, Yeng Ang, Davide Antonello, Pavana Anur, Samuel Aparicio, Elizabeth L. Appelbaum, Yasuhito Arai, Axel Aretz, Koji Arihiro, Shun-ichi Ariizumi, Joshua Armenia, Laurent Arnould, Sylvia Asa, Yassen Assenov, Gurnit Atwal, Sietse Aukema, J. Todd Auman, Miriam R. R. Aure, Philip Awadalla, Marta Aymerich, Gary D. Bader, Adrian Baez-Ortega, Matthew H. Bailey, Peter J. Bailey, Miruna Balasundaram, Saianand Balu, Pratiti Bandopadhayay, Rosamonde E. Banks, Stefano Barbi, Andrew P. Barbour, Jonathan Barenboim, Jill Barnholtz-Sloan, Hugh Barr, Elisabet Barrera, John Bartlett, Javier Bartolome, Claudio Bassi, Oliver F. Bathe, Daniel Baumhoer, Prashant Bavi, Stephen B. Baylin, Wojciech Bazant, Duncan Beardsmore, Timothy A. Beck, Sam Behjati, Andreas Behren, Beifang Niu, Cindy Bell, Sergi Beltran, Christopher Benz, Andrew Berchuck, Anke K. Bergmann, Erik N. Bergstrom, Benjamin P. Berman, Daniel M. Berney, Stephan H. Bernhart, Rameen Beroukhim, Mario Berrios, Samantha Bersani, Johanna Bertl, Miguel Betancourt, Vinayak Bhandari, Shriram G. Bhosle, Andrew V. Biankin, Matthias Bieg, Darell Bigner, Hans Binder, Ewan Birney, Michael Birrer, Nidhan K. Biswas, Bodil Bjerkehagen, Tom Bodenheimer, Lori Boice, Giada Bonizzato, Johann S. De Bono, Arnoud Boot, Moiz S. Bootwalla, Ake Borg, Arndt Borkhardt, Keith A. Boroevich, Ivan Borozan, Christoph Borst, Marcus Bosenberg, Mattia Bosio, Jacqueline Boultwood, Guillaume Bourque, Paul C. Boutros, G. Steven Bova, David T. Bowen, Reanne Bowlby, David D. L. Bowtell, Sandrine Boyault, Rich Boyce, Jeffrey Boyd, Alvis Brazma, Paul Brennan, Daniel S. Brewer, Arie B. Brinkman, Robert G. Bristow, Russell R. Broaddus, Jane E. Brock, Malcolm Brock, Annegien Broeks, Angela N. Brooks, Denise Brooks, Benedikt Brors, Søren Brunak, Timothy J. C. Bruxner, Alicia L. Bruzos, Alex Buchanan, Ivo Buchhalter, Christiane Buchholz, Susan Bullman, Hazel Burke, Birgit Burkhardt, Kathleen H. Burns, John Busanovich, Carlos D. Bustamante, Adam P. Butler, Atul J. Butte, Niall J. Byrne, Anne-Lise Børresen-Dale, Samantha J. Caesar-Johnson, Andy Cafferkey, Declan Cahill, Claudia Calabrese, Carlos Caldas, Fabien Calvo, Niedzica Camacho, Peter J. Campbell, Elias Campo, Cinzia Cantù, Shaolong Cao, Thomas E. Carey, Joana Carlevaro-Fita, Rebecca Carlsen, Ivana Cataldo, Mario Cazzola, Jonathan Cebon, Robert Cerfolio, Dianne E. Chadwick, Dimple Chakravarty, Don Chalmers, Calvin Wing Yiu Chan, Kin Chan, Michelle Chan-Seng-Yue, Vishal S. Chandan, David K. Chang, Stephen J. Chanock, Lorraine A. Chantrill, Aurélien Chateigner, Nilanjan Chatterjee, Kazuaki Chayama, Hsiao-Wei Chen, Jieming Chen, Ken Chen, Yiwen Chen, Zhaohong Chen, Andrew D. Cherniack, Jeremy Chien, Yoke-Eng Chiew, Suet-Feung Chin, Juok Cho, Sunghoon Cho, Jung Kyoon Choi, Wan Choi, Christine Chomienne, Zechen Chong, Su Pin Choo, Angela Chou, Angelika N. Christ, Elizabeth L. Christie, Eric Chuah, Carrie Cibulskis, Kristian Cibulskis, Sara Cingarlini, Peter Clapham, Alexander Claviez, Sean Cleary, Nicole Cloonan, Marek Cmero, Colin C. Collins, Ashton A. Connor, Susanna L. Cooke, Colin S. Cooper, Leslie Cope, Vincenzo Corbo, Matthew G. Cordes, Stephen M. Cordner, Isidro Cortés-Ciriano, Kyle Covington, Prue A. Cowin, Brian Craft, David Craft, Chad J. Creighton, Yupeng Cun, Erin Curley, Ioana Cutcutache, Karolina Czajka, Bogdan Czerniak, Rebecca A. Dagg, Ludmila Danilova, Maria Vittoria Davi, Natalie R. Davidson, Helen Davies, Ian J. Davis, Brandi N. Davis-Dusenbery, Kevin J. Dawson, Francisco M. De La Vega, Ricardo De Paoli-Iseppi, Timothy Defreitas, Angelo P. Dei Tos, Olivier Delaneau, John A. Demchok, Jonas Demeulemeester, German M. Demidov, Deniz Demircioğlu, Nening M. Dennis, Robert E. Denroche, Stefan C. Dentro, Nikita Desai, Vikram Deshpande, Amit G. Deshwar, Christine Desmedt, Jordi Deu-Pons, Noreen Dhalla, Neesha C. Dhani, Priyanka Dhingra, Rajiv Dhir, Anthony DiBiase, Klev Diamanti, Li Ding, Shuai Ding, Huy Q. Dinh, Luc Dirix, HarshaVardhan Doddapaneni, Nilgun Donmez, Michelle T. Dow, Ronny Drapkin, Oliver Drechsel, Ruben M. Drews, Serge Serge, Tim Dudderidge, Ana Dueso-Barroso, Andrew J. Dunford, Michael Dunn, Lewis Jonathan Dursi, Fraser R. Duthie, Ken Dutton-Regester, Jenna Eagles, Douglas F. Easton, Stuart Edmonds, Paul A. Edwards, Sandra E. Edwards, Rosalind A. Eeles, Anna Ehinger, Juergen Eils, Roland Eils, Adel El-Naggar, Matthew Eldridge, Kyle Ellrott, Serap Erkek, Georgia Escaramis, Shadrielle M. G. Espiritu, Xavier Estivill, Dariush Etemadmoghadam, Jorunn E. Eyfjord, Bishoy M. Faltas, Daiming Fan, Yu Fan, William C. Faquin, Claudiu Farcas, Matteo Fassan, Aquila Fatima, Francesco Favero, Nodirjon Fayzullaev, Ina Felau, Sian Fereday, Martin L. Ferguson, Vincent Ferretti, Lars Feuerbach, Matthew A. Field, J. Lynn Fink, Gaetano Finocchiaro, Cyril Fisher, Matthew W. Fittall, Anna Fitzgerald, Rebecca C. Fitzgerald, Adrienne M. Flanagan, Neil E. Fleshner, Paul Flicek, John A. Foekens, Kwun M. Fong, Nuno A. Fonseca, Christopher S. Foster, Natalie S. Fox, Michael Fraser, Scott Frazer, Milana Frenkel-Morgenstern, William Friedman, Joan Frigola, Catrina C. Fronick, Akihiro Fujimoto, Masashi Fujita, Masashi Fukayama, Lucinda A. Fulton, Robert S. Fulton, Mayuko Furuta, P. Andrew Futreal, Anja Füllgrabe, Stacey B. Gabriel, Steven Gallinger, Carlo Gambacorti-Passerini, Jianjiong Gao, Shengjie Gao, Levi Garraway, Øystein Garred, Erik Garrison, Dale W. Garsed, Nils Gehlenborg, Josep L. L. Gelpi, Joshy George, Daniela S. Gerhard, Clarissa Gerhauser, Jeffrey E. Gershenwald, Mark Gerstein, Moritz Gerstung, Gad Getz, Mohammed Ghori, Ronald Ghossein, Nasra H. Giama, Richard A. Gibbs, Bob Gibson, Anthony J. Gill, Pelvender Gill, Dilip D. Giri, Dominik Glodzik, Vincent J. Gnanapragasam, Maria Elisabeth Goebler, Mary J. Goldman, Carmen Gomez, Santiago Gonzalez, Abel Gonzalez-Perez, Dmitry A. Gordenin, James Gossage, Kunihito Gotoh, Ramaswamy Govindan, Dorthe Grabau, Janet S. Graham, Robert C. Grant, Anthony R. Green, Eric Green, Liliana Greger, Nicola Grehan, Sonia Grimaldi, Sean M. Grimmond, Robert L. Grossman, Adam Grundhoff, Gunes Gundem, Qianyun Guo, Manaswi Gupta, Shailja Gupta, Ivo G. Gut, Marta Gut, Jonathan Göke, Gavin Ha, Andrea Haake, David Haan, Siegfried Haas, Kerstin Haase, James E. Haber, Nina Habermann, Faraz Hach, Syed Haider, Natsuko Hama, Freddie C. Hamdy, Anne Hamilton, Mark P. Hamilton, Leng Han, George B. Hanna, Martin Hansmann, Nicholas J. Haradhvala, Olivier Harismendy, Ivon Harliwong, Arif O. Harmanci, Eoghan Harrington, Takanori Hasegawa, David Haussler, Steve Hawkins, Shinya Hayami, Shuto Hayashi, D. Neil Hayes, Stephen J. Hayes, Nicholas K. Hayward, Steven Hazell, Yao He, Allison P. Heath, Simon C. Heath, David Hedley, Apurva M. Hegde, David I. Heiman, Michael C. Heinold, Zachary Heins, Lawrence E. Heisler, Eva Hellstrom-Lindberg, Mohamed Helmy, Seong Gu Heo, Austin J. Hepperla, José María Heredia-Genestar, Carl Herrmann, Peter Hersey, Julian M. Hess, Holmfridur Hilmarsdottir, Jonathan Hinton, Satoshi Hirano, Nobuyoshi Hiraoka, Katherine A. Hoadley, Asger Hobolth, Ermin Hodzic, Jessica I. Hoell, Steve Hoffmann, Oliver Hofmann, Andrea Holbrook, Aliaksei Z. Holik, Michael A. Hollingsworth, Oliver Holmes, Robert A. Holt, Chen Hong, Eun Pyo Hong, Jongwhi H. Hong, Gerrit K. Hooijer, Henrik Hornshøj, Fumie Hosoda, Yong Hou, Volker Hovestadt, William Howat, Alan P. Hoyle, Ralph H. Hruban, Jianhong Hu, Taobo Hu, Xing Hua, Kuan-lin Huang, Mei Huang, Mi Ni Huang, Vincent Huang, Yi Huang, Wolfgang Huber, Thomas J. Hudson, Michael Hummel, Jillian A. Hung, David Huntsman, Ted R. Hupp, Jason Huse, Matthew R. Huska, Barbara Hutter, Carolyn M. Hutter, Daniel Hübschmann, Christine A. Iacobuzio-Donahue, Charles David Imbusch, Marcin Imielinski, Seiya Imoto, William B. Isaacs, Keren Isaev, Shumpei Ishikawa, Murat Iskar, S. M. Ashiqul Islam, Michael Ittmann, Sinisa Ivkovic, Jose M. G. Izarzugaza, Jocelyne Jacquemier, Valerie Jakrot, Nigel B. Jamieson, Gun Ho Jang, Se Jin Jang, Joy C. Jayaseelan, Reyka Jayasinghe, Stuart R. Jefferys, Karine Jegalian, Jennifer L. Jennings, Seung-Hyup Jeon, Lara Jerman, Yuan Ji, Wei Jiao, Peter A. Johansson, Amber L. Johns, Jeremy Johns, Rory Johnson, Todd A. Johnson, Clemency Jolly, Yann Joly, Jon G. Jonasson, Corbin D. Jones, David R. Jones, David T. W. Jones, Nic Jones, Steven J. M. Jones, Jos Jonkers, Young Seok Ju, Hartmut Juhl, Jongsun Jung, Malene Juul, Randi Istrup Juul, Sissel Juul, Natalie Jäger, Rolf Kabbe, Andre Kahles, Abdullah Kahraman, Vera B. Kaiser, Hojabr Kakavand, Sangeetha Kalimuthu, Christof von Kalle, Koo Jeong Kang, Katalin Karaszi, Beth Karlan, Rosa Karlić, Dennis Karsch, Katayoon Kasaian, Karin S. Kassahn, Hitoshi Katai, Mamoru Kato, Hiroto Katoh, Yoshiiku Kawakami, Jonathan D. Kay, Stephen H. Kazakoff, Marat D. Kazanov, Maria Keays, Electron Kebebew, Richard F. Kefford, Manolis Kellis, James G. Kench, Catherine J. Kennedy, Jules N. A. Kerssemakers, David Khoo, Vincent Khoo, Narong Khuntikeo, Ekta Khurana, Helena Kilpinen, Hark Kyun Kim, Hyung-Lae Kim, Hyung-Yong Kim, Hyunghwan Kim, Jaegil Kim, Jihoon Kim, Jong K. Kim, Youngwook Kim, Tari A. King, Wolfram Klapper, Kortine Kleinheinz, Leszek J. Klimczak, Stian Knappskog, Michael Kneba, Bartha M. Knoppers, Youngil Koh, Jan Komorowski, Daisuke Komura, Mitsuhiro Komura, Gu Kong, Marcel Kool, Jan O. Korbel, Viktoriya Korchina, Andrey Korshunov, Michael Koscher, Roelof Koster, Zsofia Kote-Jarai, Antonios Koures, Milena Kovacevic, Barbara Kremeyer, Helene Kretzmer, Markus Kreuz, Savitri Krishnamurthy, Dieter Kube, Kiran Kumar, Pardeep Kumar, Sushant Kumar, Yogesh Kumar, Ritika Kundra, Kirsten Kübler, Ralf Küppers, Jesper Lagergren, Phillip H. Lai, Peter W. Laird, Sunil R. Lakhani, Christopher M. Lalansingh, Emilie Lalonde, Fabien C. Lamaze, Adam Lambert, Eric Lander, Pablo Landgraf, Luca Landoni, Anita Langerød, Andrés Lanzós, Denis Larsimont, Erik Larsson, Mark Lathrop, Loretta M. S. Lau, Chris Lawerenz, Rita T. Lawlor, Michael S. Lawrence, Alexander J. Lazar, Ana Mijalkovic Lazic, Xuan Le, Darlene Lee, Donghoon Lee, Eunjung Alice Lee, Hee Jin Lee, Jake June-Koo Lee, Jeong-Yeon Lee, Juhee Lee, Ming Ta Michael Lee, Henry Lee-Six, Kjong-Van Lehmann, Hans Lehrach, Dido Lenze, Conrad R. Leonard, Daniel A. Leongamornlert, Ignaty Leshchiner, Louis Letourneau, Ivica Letunic, Douglas A. Levine, Lora Lewis, Tim Ley, Chang Li, Constance H. Li, Haiyan Irene Li, Jun Li, Lin Li, Shantao Li, Siliang Li, Xiaobo Li, Xiaotong Li, Xinyue Li, Yilong Li, Han Liang, Sheng-Ben Liang, Peter Lichter, Pei Lin, Ziao Lin, W. M. Linehan, Ole Christian Lingjærde, Dongbing Liu, Eric Minwei Liu, Fei-Fei Fei Liu, Fenglin Liu, Jia Liu, Xingmin Liu, Julie Livingstone, Dimitri Livitz, Naomi Livni, Lucas Lochovsky, Markus Loeffler, Georgina V. Long, Armando Lopez-Guillermo, Shaoke Lou, David N. Louis, Laurence B. Lovat, Yiling Lu, Yong-Jie Lu, Youyong Lu, Claudio Luchini, Ilinca Lungu, Xuemei Luo, Hayley J. Luxton, Andy G. Lynch, Lisa Lype, Cristina López, Carlos López-Otín, Eric Z. Ma, Yussanne Ma, Gaetan MacGrogan, Shona MacRae, Geoff Macintyre, Tobias Madsen, Kazuhiro Maejima, Andrea Mafficini, Dennis T. Maglinte, Arindam Maitra, Partha P. Majumder, Luca Malcovati, Salem Malikic, Giuseppe Malleo, Graham J. Mann, Luisa Mantovani-Löffler, Kathleen Marchal, Giovanni Marchegiani, Elaine R. Mardis, Adam A. Margolin, Maximillian G. Marin, Florian Markowetz, Julia Markowski, Jeffrey Marks, Tomas Marques-Bonet, Marco A. Marra, Luke Marsden, John W. M. Martens, Sancha Martin, Jose I. Martin-Subero, Iñigo Martincorena, Alexander Martinez-Fundichely, Yosef E. Maruvka, R. Jay Mashl, Charlie E. Massie, Thomas J. Matthew, Lucy Matthews, Erik Mayer, Simon Mayes, Michael Mayo, Faridah Mbabaali, Karen McCune, Ultan McDermott, Patrick D. McGillivray, Michael D. McLellan, John D. McPherson, John R. McPherson, Treasa A. McPherson, Samuel R. Meier, Alice Meng, Shaowu Meng, Andrew Menzies, Neil D. Merrett, Sue Merson, Matthew Meyerson, William Meyerson, Piotr A. Mieczkowski, George L. Mihaiescu, Sanja Mijalkovic, Tom Mikkelsen, Michele Milella, Linda Mileshkin, Christopher A. Miller, David K. Miller, Jessica K. Miller, Gordon B. Mills, Ana Milovanovic, Sarah Minner, Marco Miotto, Gisela Mir Arnau, Lisa Mirabello, Chris Mitchell, Thomas J. Mitchell, Satoru Miyano, Naoki Miyoshi, Shinichi Mizuno, Fruzsina Molnár-Gábor, Malcolm J. Moore, Richard A. Moore, Sandro Morganella, Quaid D. Morris, Carl Morrison, Lisle E. Mose, Catherine D. Moser, Ferran Muiños, Loris Mularoni, Andrew J. Mungall, Karen Mungall, Elizabeth A. Musgrove, Ville Mustonen, David Mutch, Francesc Muyas, Donna M. Muzny, Alfonso Muñoz, Jerome Myers, Ola Myklebost, Peter Möller, Genta Nagae, Adnan M. Nagrial, Hardeep K. Nahal-Bose, Hitoshi Nakagama, Hidewaki Nakagawa, Hiromi Nakamura, Toru Nakamura, Kaoru Nakano, Tannistha Nandi, Jyoti Nangalia, Mia Nastic, Arcadi Navarro, Fabio C. P. Navarro, David E. Neal, Gerd Nettekoven, Felicity Newell, Steven J. Newhouse, Yulia Newton, Alvin Wei Tian Ng, Anthony Ng, Jonathan Nicholson, David Nicol, Yongzhan Nie, G. Petur Nielsen, Morten Muhlig Nielsen, Serena Nik-Zainal, Michael S. Noble, Katia Nones, Paul A. Northcott, Faiyaz Notta, Brian D. O’Connor, Peter O’Donnell, Maria O’Donovan, Sarah O’Meara, Brian Patrick O’Neill, J. Robert O’Neill, David Ocana, Angelica Ochoa, Layla Oesper, Christopher Ogden, Hideki Ohdan, Kazuhiro Ohi, Lucila Ohno-Machado, Karin A. Oien, Akinyemi I. Ojesina, Hidenori Ojima, Takuji Okusaka, Larsson Omberg, Choon Kiat Ong, Stephan Ossowski, German Ott, B. F. Francis Ouellette, Christine P’ng, Marta Paczkowska, Salvatore Paiella, Chawalit Pairojkul, Marina Pajic, Qiang Pan-Hammarström, Elli Papaemmanuil, Irene Papatheodorou, Nagarajan Paramasivam, Ji Wan Park, Joong-Won Park, Keunchil Park, Kiejung Park, Peter J. Park, Joel S. Parker, Simon L. Parsons, Harvey Pass, Danielle Pasternack, Alessandro Pastore, Ann-Marie Patch, Iris Pauporté, Antonio Pea, John V. Pearson, Chandra Sekhar Pedamallu, Jakob Skou Pedersen, Paolo Pederzoli, Martin Peifer, Nathan A. Pennell, Charles M. Perou, Marc D. Perry, Gloria M. Petersen, Myron Peto, Nicholas Petrelli, Robert Petryszak, Stefan M. Pfister, Mark Phillips, Oriol Pich, Hilda A. Pickett, Todd D. Pihl, Nischalan Pillay, Sarah Pinder, Mark Pinese, Andreia V. Pinho, Esa Pitkänen, Xavier Pivot, Elena Piñeiro-Yáñez, Laura Planko, Christoph Plass, Paz Polak, Tirso Pons, Irinel Popescu, Olga Potapova, Aparna Prasad, Shaun R. Preston, Manuel Prinz, Antonia L. Pritchard, Stephenie D. Prokopec, Elena Provenzano, Xose S. Puente, Sonia Puig, Montserrat Puiggròs, Sergio Pulido-Tamayo, Gulietta M. Pupo, Colin A. Purdie, Michael C. Quinn, Raquel Rabionet, Janet S. Rader, Bernhard Radlwimmer, Petar Radovic, Benjamin Raeder, Keiran M. Raine, Manasa Ramakrishna, Kamna Ramakrishnan, Suresh Ramalingam, Benjamin J. Raphael, W. Kimryn Rathmell, Tobias Rausch, Guido Reifenberger, Jüri Reimand, Jorge Reis-Filho, Victor Reuter, Iker Reyes-Salazar, Matthew A. Reyna, Sheila M. Reynolds, Esther Rheinbay, Yasser Riazalhosseini, Andrea L. Richardson, Julia Richter, Matthew Ringel, Markus Ringnér, Yasushi Rino, Karsten Rippe, Jeffrey Roach, Lewis R. Roberts, Nicola D. Roberts, Steven A. Roberts, A. Gordon Robertson, Alan J. Robertson, Javier Bartolomé Rodriguez, Bernardo Rodriguez-Martin, F. Germán Rodríguez-González, Michael H. A. Roehrl, Marius Rohde, Hirofumi Rokutan, Gilles Romieu, Ilse Rooman, Tom Roques, Daniel Rosebrock, Mara Rosenberg, Philip C. Rosenstiel, Andreas Rosenwald, Edward W. Rowe, Romina Royo, Steven G. Rozen, Yulia Rubanova, Mark A. Rubin, Carlota Rubio-Perez, Vasilisa A. Rudneva, Borislav C. Rusev, Andrea Ruzzenente, Gunnar Rätsch, Radhakrishnan Sabarinathan, Veronica Y. Sabelnykova, Sara Sadeghi, S. Cenk Sahinalp, Natalie Saini, Mihoko Saito-Adachi, Gordon Saksena, Adriana Salcedo, Roberto Salgado, Leonidas Salichos, Richard Sallari, Charles Saller, Roberto Salvia, Michelle Sam, Jaswinder S. Samra, Francisco Sanchez-Vega, Chris Sander, Grant Sanders, Rajiv Sarin, Iman Sarrafi, Aya Sasaki-Oku, Torill Sauer, Guido Sauter, Robyn P. M. Saw, Maria Scardoni, Christopher J. Scarlett, Aldo Scarpa, Ghislaine Scelo, Dirk Schadendorf, Jacqueline E. Schein, Markus B. Schilhabel, Matthias Schlesner, Thorsten Schlomm, Heather K. Schmidt, Sarah-Jane Schramm, Stefan Schreiber, Nikolaus Schultz, Steven E. Schumacher, Roland F. Schwarz, Richard A. Scolyer, David Scott, Ralph Scully, Raja Seethala, Ayellet V. Segre, Iris Selander, Colin A. Semple, Yasin Senbabaoglu, Subhajit Sengupta, Elisabetta Sereni, Stefano Serra, Dennis C. Sgroi, Mark Shackleton, Nimish C. Shah, Sagedeh Shahabi, Catherine A. Shang, Ping Shang, Ofer Shapira, Troy Shelton, Ciyue Shen, Hui Shen, Rebecca Shepherd, Ruian Shi, Yan Shi, Yu-Jia Shiah, Tatsuhiro Shibata, Juliann Shih, Eigo Shimizu, Kiyo Shimizu, Seung Jun Shin, Yuichi Shiraishi, Tal Shmaya, Ilya Shmulevich, Solomon I. Shorser, Charles Short, Raunak Shrestha, Suyash S. Shringarpure, Craig Shriver, Shimin Shuai, Nikos Sidiropoulos, Reiner Siebert, Anieta M. Sieuwerts, Lina Sieverling, Sabina Signoretti, Katarzyna O. Sikora, Michele Simbolo, Ronald Simon, Janae V. Simons, Jared T. Simpson, Peter T. Simpson, Samuel Singer, Nasa Sinnott-Armstrong, Payal Sipahimalani, Tara J. Skelly, Marcel Smid, Jaclyn Smith, Karen Smith-McCune, Nicholas D. Socci, Heidi J. Sofia, Matthew G. Soloway, Lei Song, Anil K. Sood, Sharmila Sothi, Christos Sotiriou, Cameron M. Soulette, Paul N. Span, Paul T. Spellman, Nicola Sperandio, Andrew J. Spillane, Oliver Spiro, Jonathan Spring, Johan Staaf, Peter F. Stadler, Peter Staib, Stefan G. Stark, Lucy Stebbings, Ólafur Andri Stefánsson, Oliver Stegle, Lincoln D. Stein, Alasdair Stenhouse, Chip Stewart, Stephan Stilgenbauer, Miranda D. Stobbe, Michael R. Stratton, Jonathan R. Stretch, Adam J. Struck, Joshua M. Stuart, Henk G. Stunnenberg, Hong Su, Xiaoping Su, Ren X. Sun, Stephanie Sungalee, Hana Susak, Akihiro Suzuki, Fred Sweep, Monika Szczepanowski, Holger Sültmann, Takashi Yugawa, Angela Tam, David Tamborero, Benita Kiat Tee Tan, Donghui Tan, Patrick Tan, Hiroko Tanaka, Hirokazu Taniguchi, Tomas J. Tanskanen, Maxime Tarabichi, Roy Tarnuzzer, Patrick Tarpey, Morgan L. Taschuk, Kenji Tatsuno, Simon Tavaré, Darrin F. Taylor, Amaro Taylor-Weiner, Jon W. Teague, Bin Tean Teh, Varsha Tembe, Javier Temes, Kevin Thai, Sarah P. Thayer, Nina Thiessen, Gilles Thomas, Sarah Thomas, Alan Thompson, Alastair M. Thompson, John F. F. Thompson, R. Houston Thompson, Heather Thorne, Leigh B. Thorne, Adrian Thorogood, Grace Tiao, Nebojsa Tijanic, Lee E. Timms, Roberto Tirabosco, Marta Tojo, Stefania Tommasi, Christopher W. Toon, Umut H. Toprak, David Torrents, Giampaolo Tortora, Jörg Tost, Yasushi Totoki, David Townend, Nadia Traficante, Isabelle Treilleux, Jean-Rémi Trotta, Lorenz H. P. Trümper, Ming Tsao, Tatsuhiko Tsunoda, Jose M. C. Tubio, Olga Tucker, Richard Turkington, Daniel J. Turner, Andrew Tutt, Masaki Ueno, Naoto T. Ueno, Christopher Umbricht, Husen M. Umer, Timothy J. Underwood, Lara Urban, Tomoko Urushidate, Tetsuo Ushiku, Liis Uusküla-Reimand, Alfonso Valencia, David J. Van Den Berg, Steven Van Laere, Peter Van Loo, Erwin G. Van Meir, Gert G. Van den Eynden, Theodorus Van der Kwast, Naveen Vasudev, Miguel Vazquez, Ravikiran Vedururu, Umadevi Veluvolu, Shankar Vembu, Lieven P. C. Verbeke, Peter Vermeulen, Clare Verrill, Alain Viari, David Vicente, Caterina Vicentini, K. VijayRaghavan, Juris Viksna, Ricardo E. Vilain, Izar Villasante, Anne Vincent-Salomon, Tapio Visakorpi, Douglas Voet, Paresh Vyas, Ignacio Vázquez-García, Nick M. Waddell, Nicola Waddell, Claes Wadelius, Lina Wadi, Rabea Wagener, Jeremiah A. Wala, Jian Wang, Jiayin Wang, Linghua Wang, Qi Wang, Wenyi Wang, Yumeng Wang, Zhining Wang, Paul M. Waring, Hans-Jörg Warnatz, Jonathan Warrell, Anne Y. Warren, Sebastian M. Waszak, David C. Wedge, Dieter Weichenhan, Paul Weinberger, John N. Weinstein, Joachim Weischenfeldt, Daniel J. Weisenberger, Ian Welch, Michael C. Wendl, Johannes Werner, Justin P. Whalley, David A. Wheeler, Hayley C. Whitaker, Dennis Wigle, Matthew D. Wilkerson, Ashley Williams, James S. Wilmott, Gavin W. Wilson, Julie M. Wilson, Richard K. Wilson, Boris Winterhoff, Jeffrey A. Wintersinger, Maciej Wiznerowicz, Stephan Wolf, Bernice H. Wong, Tina Wong, Winghing Wong, Youngchoon Woo, Scott Wood, Bradly G. Wouters, Adam J. Wright, Derek W. Wright, Mark H. Wright, Chin-Lee Wu, Dai-Ying Wu, Guanming Wu, Jianmin Wu, Kui Wu, Yang Wu, Zhenggang Wu, Liu Xi, Tian Xia, Qian Xiang, Xiao Xiao, Rui Xing, Heng Xiong, Qinying Xu, Yanxun Xu, Hong Xue, Shinichi Yachida, Sergei Yakneen, Rui Yamaguchi, Takafumi N. Yamaguchi, Masakazu Yamamoto, Shogo Yamamoto, Hiroki Yamaue, Fan Yang, Huanming Yang, Jean Y. Yang, Liming Yang, Lixing Yang, Shanlin Yang, Tsun-Po Yang, Yang Yang, Xiaotong Yao, Marie-Laure Yaspo, Lucy Yates, Christina Yau, Chen Ye, Kai Ye, Venkata D. Yellapantula, Christopher J. Yoon, Sung-Soo Yoon, Fouad Yousif, Jun Yu, Kaixian Yu, Willie Yu, Yingyan Yu, Ke Yuan, Yuan Yuan, Denis Yuen, Christina K. Yung, Olga Zaikova, Jorge Zamora, Marc Zapatka, Jean C. Zenklusen, Thorsten Zenz, Nikolajs Zeps, Cheng-Zhong Zhang, Fan Zhang, Hailei Zhang, Hongwei Zhang, Hongxin Zhang, Jiashan Zhang, Jing Zhang, Junjun Zhang, Xiuqing Zhang, Xuanping Zhang, Yan Zhang, Zemin Zhang, Zhongming Zhao, Liangtao Zheng, Xiuqing Zheng, Wanding Zhou, Yong Zhou, Bin Zhu, Hongtu Zhu, Jingchun Zhu, Shida Zhu, Lihua Zou, Xueqing Zou, Anna deFazio, Nicholas van As, Carolien H. M. van Deurzen, Marc J. van de Vijver, L. van’t Veer, Christian von Mering

**Affiliations:** 1 Department of Computer Science, Princeton University, Princeton, NJ 08540 USA; 2 Department of Biomedical Informatics, Emory University, Atlanta, GA 30322 USA; 3 Department of Biomolecular Engineering and UC Santa Cruz Genomics Institute, University of California, Santa Cruz, Santa Cruz, CA 95060 USA; 4 Computational Biology Program, Ontario Institute for Cancer Research, Toronto, Ontario, Canada; 5 Department of Information Technology, IDLab, Ghent University, IMEC, Ghent, the Netherlands; 6 Department of Plant Biotechnology and Bioinformatics, Ghent University, Ghent, the Netherlands; 7 Barcelona Supercomputing Center (BSC), Barcelona, 08034 Spain; 8 Department of Clinical and Molecular Medicine, Faculty of Medicine and Health Sciences, Norwegian University of Science and Technology, Trondheim, Norway; 9 Institute of Molecular Life Sciences and Swiss Institute of Bioinformatics, University of Zurich, CH-8057 Zurich, Switzerland; 10 Department of Pathology and Molecular Pathology, University Hospital Zurich, CH-8091 Zurich, Switzerland; 11 Department of Physiology and Biophysics, Weill Cornell Medicine, New York, NY 10065 USA; 12 Vancouver Prostate Centre, 2660 Oak Street, Vancouver, BC V6H 3Z6 Canada; 13 The Broad Institute of MIT and Harvard, Cambridge, MA 02124 USA; 14 Massachusetts General Hospital Center for Cancer Research, Charlestown, MA 02129 USA; 15 Harvard Medical School, 250 Longwood Avenue, Boston, MA 02115 USA; 16 Massachusetts General Hospital, Department of Pathology, Boston, MA 02114 USA; 17 Department of Molecular Medicine (MOMA), Aarhus University Hospital, Aarhus, Denmark; 18 Bioinformatics Research Centre (BiRC), Aarhus University, Aarhus, Denmark; 19 Human Genome Sequencing Center, Baylor College of Medicine, Houston, TX 77030 USA; 20 DTU Bioinformatics, Department of Bio and Health Informatics, Technical University of Denmark, Kemitorvet, 2800 Kongens Lyngby, Denmark; 21 Novo Nordisk Foundation Center for Protein Research, Faculty of Health and Medical Sciences, University of Copenhagen, 2200 Copenhagen, Denmark; 22 Department of Computer Science, Indiana University, Bloomington, IN 47405 USA; 23 ICREA, Barcelona, 08010 Spain; 24 Department of Medical Biophysics, University of Toronto, Toronto, Ontario, Canada; 25 Wellcome Sanger Institute, Wellcome Genome Campus, Hinxton, Cambridge, CB10 1SA UK; 26 Department of Genomic Medicine, The University of Texas MD Anderson Cancer Center, Houston, TX 77030 USA; 27 The Jackson Laboratory for Genomic Medicine, Farmington, CT 06032 USA; 28 Quantitative & Computational Biosciences Graduate Program, Baylor College of Medicine, Houston, TX 77030 USA; 29 Department of Molecular Genetics, University of Toronto, Toronto, ON M5S 1A8 Canada; 30 Dana-Farber/Boston Children’s Cancer and Blood Disorders Center, Boston, MA 02215 USA; 31 Department of Pediatrics, Harvard Medical School, Boston, MA 02115 USA; 32 Broad Institute of MIT and Harvard, Cambridge, MA 02142 USA; 33 Department of Medical Oncology, Dana-Farber Cancer Institute, Boston, MA 02115 USA; 34 Harvard Medical School, Boston, MA 02115 USA; 35 Department of Mathematics, Aarhus University, Aarhus, 8000 Denmark; 36 Laboratory for Medical Science Mathematics, RIKEN Center for Integrative Medical Sciences, Yokohama, Kanagawa 230-0045 Japan; 37 RIKEN Center for Integrative Medical Sciences, Yokohama, Kanagawa 230-0045 Japan; 38 Foundation Medicine, Inc, Cambridge, MA 02141 USA; 39 Department of Haematology, University of Cambridge, Cambridge, CB2 2XY UK; 40 Department for BioMedical Research, University of Bern, Bern, 3008 Switzerland; 41 Department of Medical Oncology, Inselspital, University Hospital and University of Bern, Bern, 3010 Switzerland; 42 Graduate School for Cellular and Biomedical Sciences, University of Bern, Bern, 3012 Switzerland; 43 Department of Genitourinary Medical Oncology - Research, Division of Cancer Medicine, The University of Texas MD Anderson Cancer Center, Houston, TX 77030 USA; 44 Division of Theoretical Bioinformatics, German Cancer Research Center (DKFZ), Heidelberg, 69120 Germany; 45 Faculty of Biosciences, Heidelberg University, Heidelberg, 69120 Germany; 46 University of Texas MD Anderson Cancer Center, Houston, TX 77030 USA; 47 Korea Advanced Institute of Science and Technology, Daejeon, 34141 South Korea; 48 Institute for Research in Biomedicine (IRB Barcelona), The Barcelona Institute of Science and Technology, Barcelona, 8003 Spain; 49 Research Program on Biomedical Informatics, Universitat Pompeu Fabra, Barcelona, 08002 Spain; 50 Science for Life Laboratory, Department of Cell and Molecular Biology, Uppsala University, Uppsala, SE-75124 Sweden; 51 Division of Applied Bioinformatics, German Cancer Research Center (DKFZ), Heidelberg, 69120 Germany; 52 Queensland Centre for Medical Genomics, Institute for Molecular Bioscience, The University of Queensland, St Lucia, QLD 4072 Australia; 53 CIBIO/InBIO - Research Center in Biodiversity and Genetic Resources, Universidade do Porto, Vairão, 4485-601 Portugal; 54 European Molecular Biology Laboratory, European Bioinformatics Institute (EMBL-EBI), Wellcome Genome Campus, Hinxton, Cambridge, CB10 1SD UK; 55 University of Milano Bicocca, Monza, 20052 Italy; 56 Peter MacCallum Cancer Centre and University of Melbourne, Melbourne,, VIC 3000 Australia; 57 Sir Peter MacCallum Department of Oncology, The University of Melbourne, Melbourne, VIC 3052 Australia; 58 Department of Computer Science, Yale University, New Haven, CT 06520 USA; 59 Department of Molecular Biophysics and Biochemistry, Yale University, New Haven, CT 06520 USA; 60 Program in Computational Biology and Bioinformatics, Yale University, New Haven, CT 06520 USA; 61 CNAG-CRG, Centre for Genomic Regulation (CRG), Barcelona Institute of Science and Technology (BIST), Barcelona, 08028 Spain; 62 Universitat Pompeu Fabra (UPF), Barcelona, 08003 Spain; 63 Department of Internal Medicine, Stanford University, Stanford, CA 94305 USA; 64 Massachusetts General Hospital, Boston, MA 02114 USA; 65 Center for Precision Health, School of Biomedical Informatics, University of Texas Health Science Center, Houston, TX 77030 USA; 66 The Donnelly Centre, University of Toronto, Toronto, ON M5S 3E1 Canada; 67 Health Data Science Unit, University Clinics, Heidelberg, 69120 Germany; 68 Institute of Pharmacy and Molecular Biotechnology and BioQuant, Heidelberg University, Heidelberg, 69120 Germany; 69 Simon Fraser University, Burnaby, BC V5A 1S6 Canada; 70 Computational Biology Program, Ontario Institute for Cancer Research, Toronto, ON M5G 0A3 Canada; 71 Computational Biology Center, Memorial Sloan Kettering Cancer Center, New York, NY 10065 USA; 72 ETH Zurich, Department of Biology, Wolfgang-Pauli-Strasse 27, Zürich, 8093 Switzerland; 73 ETH Zurich, Department of Computer Science, Zurich, 8092 Switzerland; 74 SIB Swiss Institute of Bioinformatics, Lausanne, 1015 Switzerland; 75 University Hospital Zurich, Zurich, 8091 Switzerland; 76 MIT Computer Science and Artificial Intelligence Laboratory, Massachusetts Institute of Technology, Cambridge, MA 02139 USA; 77 Research Core Center, National Cancer Centre Korea, Goyang-si, 410-769 South Korea; 78 Department of Health Sciences and Technology, Sungkyunkwan University School of Medicine, Seoul, 06351 South Korea; 79 Samsung Genome Institute, Samsung Medical Center, Seoul, Korea; 80 Institute of Computer Science, Polish Academy of Sciences, Warsawa, 01-248 Poland; 81 Genome Biology Unit, European Molecular Biology Laboratory (EMBL), Heidelberg, 69117 Germany; 82 Institute of Biomedicine, Sahlgrenska Academy at University of Gothenburg, Gothenburg, Sweden; 83 Harvard University, Cambridge, MA 02138 USA; 84 Department of Physiology and Biophysics, Weill Cornell Medicine, New York, NY 10065 USA; 85 Institute for Computational Biomedicine, Weill Cornell Medicine, New York, NY 10021 USA; 86 Memorial Sloan Kettering Cancer Center, New York, NY 10065 USA; 87 Yale University, New Haven, CT 06520 USA; 88 Englander Institute for Precision Medicine, Weill Cornell Medicine, New York, NY 10065 USA; 89 Yale School of Medicine, Yale University, New Haven, CT 06520 USA; 90 Institute for Research in Biomedicine (IRB Barcelona), The Barcelona Institute of Science and Technology, Barcelona, Spain; 91 Division of Hematology-Oncology, Samsung Medical Center, Sungkyunkwan University School of Medicine, Seoul, 06351 South Korea; 92 Samsung Advanced Institute for Health Sciences and Technology, Sungkyunkwan University School of Medicine, Seoul, 06351 South Korea; 93 Cheonan Industry-Academic Collaboration Foundation, Sangmyung University, Cheonan, 31066 South Korea; 94 Spanish National Cancer Research Centre, Madrid, 28029 Spain; 95 Vall d’Hebron Institute of Oncology: VHIO, Barcelona, 08035 Spain; 96 cBio Center, Dana-Farber Cancer Institute, Harvard Medical School, Boston, MA 02115 USA; 97 Department of Cell Biology, Harvard Medical School, Boston, MA 02115 USA; 98 Department of Cancer Biology, Dana-Farber Cancer Institute, Boston, MA 02215 USA; 99 cBio Center, Dana-Farber Cancer Institute, Boston, MA 02215 USA; 100 Finsen Laboratory and Biotech Research & Innovation Centre (BRIC), University of Copenhagen, Copenhagen, 2200 Denmark; 101 Department of Genetics, Stanford University School of Medicine, Stanford, CA 94305 USA; 102 CREST, Japan Science and Technology Agency, Tokyo, 113-0033 Japan; 103 Department of Medical Science Mathematics, Medical Research Institute, Tokyo Medical and Dental University, Bunkyo-ku, Tokyo, 113-8510 Japan; 104 Laboratory for Medical Science Mathematics, Department of Biological Sciences, Graduate School of Science, The University of Tokyo, Bunkyo-ku, Tokyo, 113-0033 Japan; 105 Department of Oncology-Pathology, Science for Life Laboratory, Karolinska Institute, Stockholm, Sweden; 106 Department of Gene Technology, Tallinn University of Technology, Tallinn, 12616 Estonia; 107 Genetics & Genome Biology Program, SickKids Research Institute, The Hospital for Sick Children, Toronto, ON M5G 1X8 Canada; 108 Science for Life Laboratory, Department of Immunology, Genetics and Pathology, Uppsala University, Uppsala, SE-75108 Sweden; 109 School of Computer Science and Technology, Xi’an Jiaotong University, Xi’an, 710048 China; 110 School of Electronic and Information Engineering, Xi’an Jiaotong University, Xi’an, 710048 China; 111 The McDonnell Genome Institute at Washington University, St Louis, MO 63108 USA; 112 Department of Urology, Charité Universitätsmedizin Berlin, Berlin, 10117 Germany; 113 Oregon Health & Sciences University, Portland, OR 97239 USA; 114 Department of Medicine and Therapeutics, The Chinese University of Hong Kong, Shatin, NT Hong Kong, China; 115 Second Military Medical University, Shanghai, 200433 China; 116 The University of Texas Health Science Center at Houston, Houston, TX 77030 USA; 117 Department of Biomedical Informatics, College of Medicine, The Ohio State University, Columbus, OH 43210 USA; 118 The Ohio State University Comprehensive Cancer Center (OSUCCC – James), Columbus, OH 43210 USA; 119 School of Biomedical Informatics, The University of Texas Health Science Center at Houston, Houston, TX 77030 USA; 120 Department of Biochemistry and Molecular Genetics, Feinberg School of Medicine, Northwestern University, Chicago, IL 60637 USA; 200 Applied Tumor Genomics Research Program, Research Programs Unit, University of Helsinki, Helsinki, Finland; 201 Wellcome Sanger Institute, Wellcome Genome Campus, Hinxton, UK; 202 Memorial Sloan Kettering Cancer Center, New York, NY USA; 203 Genome Science Division, Research Center for Advanced Science and Technology, University of Tokyo, Tokyo, Japan; 204 Department of Surgery, University of Chicago, Chicago, IL USA; 205 Department of Surgery, Division of Hepatobiliary and Pancreatic Surgery, School of Medicine, Keimyung University Dongsan Medical Center, Daegu, South Korea; 206 Department of Oncology, Gil Medical Center, Gachon University, Incheon, South Korea; 207 Hiroshima University, Hiroshima, Japan; 208 Department of Bioinformatics and Computational Biology, The University of Texas MD Anderson Cancer Center, Houston, TX USA; 209 University of Texas MD Anderson Cancer Center, Houston, TX USA; 210 King Faisal Specialist Hospital and Research Centre, Al Maather, Riyadh, Saudi Arabia; 211 Bioinformatics Unit, Spanish National Cancer Research Centre (CNIO), Madrid, Spain; 212 Bioinformatics Core Facility, University Medical Center Hamburg, Hamburg, Germany; 213 Heinrich Pette Institute, Leibniz Institute for Experimental Virology, Hamburg, Germany; 214 Ontario Tumour Bank, Ontario Institute for Cancer Research, Toronto, ON Canada; 215 Department of Pathology, The University of Texas MD Anderson Cancer Center, Houston, TX USA; 216 Laboratory of Pathology, Center for Cancer Research, National Cancer Institute, Bethesda, MD USA; 217 Department of Cellular and Molecular Medicine and Department of Bioengineering, University of California San Diego, La Jolla, CA USA; 218 UC San Diego Moores Cancer Center, San Diego, CA USA; 219 Canada’s Michael Smith Genome Sciences Centre, BC Cancer, Vancouver, BC Canada; 220 Sir Peter MacCallum Department of Oncology, Peter MacCallum Cancer Centre, University of Melbourne, Melbourne, VIC Australia; 221 Centre for Research in Molecular Medicine and Chronic Diseases (CiMUS), Universidade de Santiago de Compostela, Santiago de Compostela, Spain; 222 Department of Zoology, Genetics and Physical Anthropology, (CiMUS), Universidade de Santiago de Compostela, Santiago de Compostela, Spain; 223 The Biomedical Research Centre (CINBIO), Universidade de Vigo, Vigo, Spain; 224 Royal National Orthopaedic Hospital - Bolsover, London, UK; 225 Department of Genomic Medicine, The University of Texas MD Anderson Cancer Center, Houston, TX USA; 226 Quantitative and Computational Biosciences Graduate Program, Baylor College of Medicine, Houston, TX USA; 227 The Jackson Laboratory for Genomic Medicine, Farmington, CT USA; 228 Genome Informatics Program, Ontario Institute for Cancer Research, Toronto, ON Canada; 229 Institute of Human Genetics, Christian-Albrechts-University, Kiel, Germany; 230 Institute of Human Genetics, Ulm University and Ulm University Medical Center, Ulm, Germany; 231 Queensland Centre for Medical Genomics, Institute for Molecular Bioscience, University of Queensland, St. Lucia, Brisbane, QLD Australia; 232 Salford Royal NHS Foundation Trust, Salford, UK; 233 Department of Surgery, Pancreas Institute, University and Hospital Trust of Verona, Verona, Italy; 234 Molecular and Medical Genetics, OHSU Knight Cancer Institute, Oregon Health and Science University, Portland, OR USA; 235 Department of Molecular Oncology, BC Cancer Research Centre, Vancouver, BC Canada; 236 The McDonnell Genome Institute at Washington University, St. Louis, MO USA; 237 University College London, London, UK; 238 Division of Cancer Genomics, National Cancer Center Research Institute, National Cancer Center, Tokyo, Japan; 239 DLR Project Management Agency, Bonn, Germany; 240 Tokyo Women’s Medical University, Tokyo, Japan; 241 Center for Molecular Oncology, Memorial Sloan Kettering Cancer Center, New York, NY USA; 242 Los Alamos National Laboratory, Los Alamos, NM USA; 243 Department of Pathology, University Health Network, Toronto General Hospital, Toronto, ON Canada; 244 Nottingham University Hospitals NHS Trust, Nottingham, UK; 245 Epigenomics and Cancer Risk Factors, German Cancer Research Center (DKFZ), Heidelberg, Germany; 246 Computational Biology Program, Ontario Institute for Cancer Research, Toronto, ON Canada; 247 Department of Molecular Genetics, University of Toronto, Toronto, ON Canada; 248 Vector Institute, Toronto, ON Canada; 249 Hematopathology Section, Institute of Pathology, Christian-Albrechts-University, Kiel, Germany; 250 Department of Pathology and Laboratory Medicine, School of Medicine, University of North Carolina at Chapel Hill, Chapel Hill, NC USA; 251 Department of Cancer Genetics, Institute for Cancer Research, Oslo University Hospital, The Norwegian Radium Hospital, Oslo, Norway; 252 Pathology, Hospital Clinic, Institut d’Investigacions Biomèdiques August Pi i Sunyer (IDIBAPS), University of Barcelona, Barcelona, Spain; 253 Department of Veterinary Medicine, Transmissible Cancer Group, University of Cambridge, Cambridge, UK; 254 Alvin J. Siteman Cancer Center, Washington University School of Medicine, St. Louis, MO USA; 255 Wolfson Wohl Cancer Research Centre, Institute of Cancer Sciences, University of Glasgow, Glasgow, UK; 256 Lineberger Comprehensive Cancer Center, University of North Carolina at Chapel Hill, Chapel Hill, NC USA; 257 Broad Institute of MIT and Harvard, Cambridge, MA USA; 258 Dana-Farber/Boston Children’s Cancer and Blood Disorders Center, Boston, MA USA; 259 Department of Pediatrics, Harvard Medical School, Boston, MA USA; 260 Leeds Institute of Medical Research @ St. James’s, University of Leeds, St. James’s University Hospital, Leeds, UK; 261 Department of Pathology and Diagnostics, University and Hospital Trust of Verona, Verona, Italy; 262 Department of Surgery, Princess Alexandra Hospital, Brisbane, QLD Australia; 263 Surgical Oncology Group, Diamantina Institute, University of Queensland, Brisbane, QLD Australia; 264 Department of Population and Quantitative Health Sciences, Case Western Reserve University School of Medicine, Cleveland, OH USA; 265 Research Health Analytics and Informatics, University Hospitals Cleveland Medical Center, Cleveland, OH USA; 266 Gloucester Royal Hospital, Gloucester, UK; 267 European Molecular Biology Laboratory, European Bioinformatics Institute (EMBL-EBI), Cambridge, UK; 268 Diagnostic Development, Ontario Institute for Cancer Research, Toronto, ON Canada; 269 Barcelona Supercomputing Center (BSC), Barcelona, Spain; 270 Arnie Charbonneau Cancer Institute, University of Calgary, Calgary, AB Canada; 271 Departments of Surgery and Oncology, University of Calgary, Calgary, AB Canada; 272 Department of Pathology, Oslo University Hospital, The Norwegian Radium Hospital, Oslo, Norway; 273 PanCuRx Translational Research Initiative, Ontario Institute for Cancer Research, Toronto, ON Canada; 274 Department of Oncology, Sidney Kimmel Comprehensive Cancer Center at Johns Hopkins University School of Medicine, Baltimore, MD USA; 275 University Hospital Southampton NHS Foundation Trust, Southampton, UK; 276 Royal Stoke University Hospital, Stoke-on-Trent, UK; 277 Genome Sequence Informatics, Ontario Institute for Cancer Research, Toronto, ON Canada; 278 Human Longevity Inc, San Diego, CA USA; 279 Olivia Newton-John Cancer Research Institute, La Trobe University, Heidelberg, VIC Australia; 280 Computer Network Information Center, Chinese Academy of Sciences, Beijing, China; 281 Genome Canada, Ottawa, ON Canada; 282 CNAG-CRG, Centre for Genomic Regulation (CRG), Barcelona Institute of Science and Technology (BIST), Barcelona, Spain; 283 Universitat Pompeu Fabra (UPF), Barcelona, Spain; 284 Buck Institute for Research on Aging, Novato, CA USA; 285 Duke University Medical Center, Durham, NC USA; 286 Department of Human Genetics, Hannover Medical School, Hannover, Germany; 287 Center for Bioinformatics and Functional Genomics, Cedars-Sinai Medical Center, Los Angeles, CA USA; 288 Department of Biomedical Sciences, Cedars-Sinai Medical Center, Los Angeles, CA USA; 289 The Hebrew University Faculty of Medicine, Jerusalem, Israel; 290 Barts Cancer Institute, Barts and the London School of Medicine and Dentistry, Queen Mary University of London, London, UK; 291 Department of Computer Science, Bioinformatics Group, University of Leipzig, Leipzig, Germany; 292 Interdisciplinary Center for Bioinformatics, University of Leipzig, Leipzig, Germany; 293 Transcriptome Bioinformatics, LIFE Research Center for Civilization Diseases, University of Leipzig, Leipzig, Germany; 294 Department of Medical Oncology, Dana-Farber Cancer Institute, Boston, MA USA; 295 Department of Cancer Biology, Dana-Farber Cancer Institute, Boston, MA USA; 296 Harvard Medical School, Boston, MA USA; 297 USC Norris Comprehensive Cancer Center, University of Southern California, Los Angeles, CA USA; 298 Department of Diagnostics and Public Health, University and Hospital Trust of Verona, Verona, Italy; 299 Department of Mathematics, Aarhus University, Aarhus, Denmark; 300 Department of Molecular Medicine (MOMA), Aarhus University Hospital, Aarhus N, Denmark; 301 Instituto Carlos Slim de la Salud, Mexico City, Mexico; 302 Department of Medical Biophysics, University of Toronto, Toronto, ON Canada; 303 Cancer Division, Garvan Institute of Medical Research, Kinghorn Cancer Centre, University of New South Wales (UNSW Sydney), Sydney, NSW Australia; 304 South Western Sydney Clinical School, Faculty of Medicine, University of New South Wales (UNSW Sydney), Liverpool, NSW Australia; 305 West of Scotland Pancreatic Unit, Glasgow Royal Infirmary, Glasgow, UK; 306 Center for Digital Health, Berlin Institute of Health and Charitè - Universitätsmedizin Berlin, Berlin, Germany; 307 Heidelberg Center for Personalized Oncology (DKFZ-HIPO), German Cancer Research Center (DKFZ), Heidelberg, Germany; 308 The Preston Robert Tisch Brain Tumor Center, Duke University Medical Center, Durham, NC USA; 309 Massachusetts General Hospital, Boston, MA USA; 310 National Institute of Biomedical Genomics, Kalyani, West Bengal India; 311 Institute of Clinical Medicine and Institute of Oral Biology, University of Oslo, Oslo, Norway; 312 University of North Carolina at Chapel Hill, Chapel Hill, NC USA; 313 ARC-Net Centre for Applied Research on Cancer, University and Hospital Trust of Verona, Verona, Italy; 314 The Institute of Cancer Research, London, UK; 315 Centre for Computational Biology, Duke-NUS Medical School, Singapore, Singapore; 316 Programme in Cancer and Stem Cell Biology, Duke-NUS Medical School, Singapore, Singapore; 317 Division of Oncology and Pathology, Department of Clinical Sciences Lund, Lund University, Lund, Sweden; 318 Department of Pediatric Oncology, Hematology and Clinical Immunology, Heinrich-Heine-University, Düsseldorf, Germany; 319 Laboratory for Medical Science Mathematics, RIKEN Center for Integrative Medical Sciences, Yokohama, Japan; 320 RIKEN Center for Integrative Medical Sciences, Yokohama, Japan; 321 Department of Internal Medicine/Hematology, Friedrich-Ebert-Hospital, Neumünster, Germany; 322 Departments of Dermatology and Pathology, Yale University, New Haven, CT USA; 323 Centre for Genomic Regulation (CRG), The Barcelona Institute of Science and Technology, Barcelona, Spain; 324 Radcliffe Department of Medicine, University of Oxford, Oxford, UK; 325 Canadian Center for Computational Genomics, McGill University, Montreal, QC Canada; 326 Department of Human Genetics, McGill University, Montreal, QC Canada; 327 Department of Human Genetics, University of California Los Angeles, Los Angeles, CA USA; 328 Department of Pharmacology, University of Toronto, Toronto, ON Canada; 329 Faculty of Medicine and Health Technology, Tampere University and Tays Cancer Center, Tampere University Hospital, Tampere, Finland; 330 Haematology, Leeds Teaching Hospitals NHS Trust, Leeds, UK; 331 Translational Research and Innovation, Centre Léon Bérard, Lyon, France; 332 Fox Chase Cancer Center, Philadelphia, PA USA; 333 International Agency for Research on Cancer, World Health Organization, Lyon, France; 334 Earlham Institute, Norwich, UK; 335 Norwich Medical School, University of East Anglia, Norwich, UK; 336 Department of Molecular Biology, Faculty of Science, Radboud Institute for Molecular Life Sciences, Radboud University, Nijmegen, HB The Netherlands; 337 CRUK Manchester Institute and Centre, Manchester, UK; 338 Department of Radiation Oncology, University of Toronto, Toronto, ON Canada; 339 Division of Cancer Sciences, Manchester Cancer Research Centre, University of Manchester, Manchester, UK; 340 Radiation Medicine Program, Princess Margaret Cancer Centre, Toronto, ON Canada; 341 Department of Pathology, Brigham and Women’s Hospital, Harvard Medical School, Boston, MA USA; 342 Department of Surgery, Division of Thoracic Surgery, The Johns Hopkins University School of Medicine, Baltimore, MD USA; 343 Division of Molecular Pathology, The Netherlands Cancer Institute, Oncode Institute, Amsterdam, CX The Netherlands; 344 Department of Biomolecular Engineering, University of California Santa Cruz, Santa Cruz, CA USA; 345 UC Santa Cruz Genomics Institute, University of California Santa Cruz, Santa Cruz, CA USA; 346 Division of Applied Bioinformatics, German Cancer Research Center (DKFZ), Heidelberg, Germany; 347 German Cancer Consortium (DKTK), German Cancer Research Center (DKFZ), Heidelberg, Germany; 348 National Center for Tumor Diseases (NCT) Heidelberg, Heidelberg, Germany; 349 Center for Biological Sequence Analysis, Department of Bio and Health Informatics, Technical University of Denmark, Lyngby, Denmark; 350 Novo Nordisk Foundation Center for Protein Research, University of Copenhagen, Copenhagen, Denmark; 351 Institute for Molecular Bioscience, University of Queensland, St. Lucia, Brisbane, QLD Australia; 352 Biomedical Engineering, Oregon Health and Science University, Portland, OR USA; 353 Division of Theoretical Bioinformatics, German Cancer Research Center (DKFZ), Heidelberg, Germany; 354 Institute of Pharmacy and Molecular Biotechnology and BioQuant, Heidelberg University, Heidelberg, Germany; 355 Federal Ministry of Education and Research, Berlin, Germany; 356 Melanoma Institute Australia, University of Sydney, Sydney, NSW Australia; 357 Pediatric Hematology and Oncology, University Hospital Muenster, Muenster, Germany; 358 Department of Pathology, Johns Hopkins University School of Medicine, Baltimore, MD USA; 359 McKusick-Nathans Institute of Genetic Medicine, Sidney Kimmel Comprehensive Cancer Center at Johns Hopkins University School of Medicine, Baltimore, MD USA; 360 Foundation Medicine, Inc, Cambridge, MA USA; 361 Department of Biomedical Data Science, Stanford University School of Medicine, Stanford, CA USA; 362 Department of Genetics, Stanford University School of Medicine, Stanford, CA USA; 363 Bakar Computational Health Sciences Institute and Department of Pediatrics, University of California, San Francisco, CA USA; 364 Institute of Clinical Medicine, Faculty of Medicine, University of Oslo, Oslo, Norway; 365 National Cancer Institute, National Institutes of Health, Bethesda, MD USA; 366 Royal Marsden NHS Foundation Trust, London and Sutton, UK; 367 Genome Biology Unit, European Molecular Biology Laboratory (EMBL), Heidelberg, Germany; 368 Department of Oncology, University of Cambridge, Cambridge, UK; 369 Li Ka Shing Centre, Cancer Research UK Cambridge Institute, University of Cambridge, Cambridge, UK; 370 Institut Gustave Roussy, Villejuif, France; 371 Cambridge University Hospitals NHS Foundation Trust, Cambridge, UK; 372 Department of Haematology, University of Cambridge, Cambridge, UK; 373 Anatomia Patológica, Hospital Clinic, Institut d’Investigacions Biomèdiques August Pi i Sunyer (IDIBAPS), University of Barcelona, Barcelona, Spain; 374 Spanish Ministry of Science and Innovation, Madrid, Spain; 375 University of Michigan Comprehensive Cancer Center, Ann Arbor, MI USA; 376 Department for BioMedical Research, University of Bern, Bern, Switzerland; 377 Department of Medical Oncology, Inselspital, University Hospital and University of Bern, Bern, Switzerland; 378 Graduate School for Cellular and Biomedical Sciences, University of Bern, Bern, Switzerland; 379 University of Pavia, Pavia, Italy; 380 University of Alabama at Birmingham, Birmingham, AL USA; 381 UHN Program in BioSpecimen Sciences, Toronto General Hospital, Toronto, ON Canada; 382 Department of Urology, Icahn School of Medicine at Mount Sinai, New York, NY USA; 383 Centre for Law and Genetics, University of Tasmania, Sandy Bay Campus, Hobart, TAS Australia; 384 Faculty of Biosciences, Heidelberg University, Heidelberg, Germany; 385 Department of Biochemistry, Microbiology and Immunology, Faculty of Medicine, University of Ottawa, Ottawa, ON Canada; 386 Division of Anatomic Pathology, Mayo Clinic, Rochester, MN USA; 387 Division of Cancer Epidemiology and Genetics, National Cancer Institute, National Institutes of Health, Bethesda, MD USA; 388 Illawarra Shoalhaven Local Health District L3 Illawarra Cancer Care Centre, Wollongong Hospital, Wollongong, NSW Australia; 389 BioForA, French National Institute for Agriculture, Food, and Environment (INRAE), ONF, Orléans, France; 390 Department of Biostatistics, Bloomberg School of Public Health, Johns Hopkins University, Baltimore, MD USA; 391 University of California San Diego, San Diego, CA USA; 392 Division of Experimental Pathology, Mayo Clinic, Rochester, MN USA; 393 Centre for Cancer Research, The Westmead Institute for Medical Research, University of Sydney, Sydney, NSW Australia; 394 Department of Gynaecological Oncology, Westmead Hospital, Sydney, NSW Australia; 395 PDXen Biosystems Inc, Seoul, South Korea; 396 Korea Advanced Institute of Science and Technology, Daejeon, South Korea; 397 Electronics and Telecommunications Research Institute, Daejeon, South Korea; 398 Institut National du Cancer (INCA), Boulogne-Billancourt, France; 399 Department of Genetics, Informatics Institute, University of Alabama at Birmingham, Birmingham, AL USA; 400 Division of Medical Oncology, National Cancer Centre, Singapore, Singapore; 401 Medical Oncology, University and Hospital Trust of Verona, Verona, Italy; 402 Department of Pediatrics, University Hospital Schleswig-Holstein, Kiel, Germany; 403 Hepatobiliary/Pancreatic Surgical Oncology Program, University Health Network, Toronto, ON Canada; 404 School of Biological Sciences, University of Auckland, Auckland, New Zealand; 405 Department of Surgery, University of Melbourne, Parkville, VIC Australia; 406 The Murdoch Children’s Research Institute, Royal Children’s Hospital, Parkville, VIC Australia; 407 Walter and Eliza Hall Institute, Parkville, VIC Australia; 408 Vancouver Prostate Centre, Vancouver, Canada; 409 Lunenfeld-Tanenbaum Research Institute, Mount Sinai Hospital, Toronto, ON Canada; 410 University of East Anglia, Norwich, UK; 411 Norfolk and Norwich University Hospital NHS Trust, Norwich, UK; 412 Victorian Institute of Forensic Medicine, Southbank, VIC Australia; 413 Department of Biomedical Informatics, Harvard Medical School, Boston, MA USA; 414 Department of Chemistry, Centre for Molecular Science Informatics, University of Cambridge, Cambridge, UK; 415 Ludwig Center at Harvard Medical School, Boston, MA USA; 416 Human Genome Sequencing Center, Baylor College of Medicine, Houston, TX USA; 417 Peter MacCallum Cancer Centre, University of Melbourne, Melbourne, VIC Australia; 418 Physics Division, Optimization and Systems Biology Lab, Massachusetts General Hospital, Boston, MA USA; 419 Department of Medicine, Baylor College of Medicine, Houston, TX USA; 420 University of Cologne, Cologne, Germany; 421 International Genomics Consortium, Phoenix, AZ USA; 422 Genomics Research Program, Ontario Institute for Cancer Research, Toronto, ON Canada; 423 Barking Havering and Redbridge University Hospitals NHS Trust, Romford, UK; 424 Children’s Hospital at Westmead, University of Sydney, Sydney, NSW Australia; 425 Department of Medicine, Section of Endocrinology, University and Hospital Trust of Verona, Verona, Italy; 426 Computational Biology Center, Memorial Sloan Kettering Cancer Center, New York, NY USA; 427 Department of Biology, ETH Zurich, Zürich, Switzerland; 428 Department of Computer Science, ETH Zurich, Zurich, Switzerland; 429 SIB Swiss Institute of Bioinformatics, Lausanne, Switzerland; 430 Weill Cornell Medical College, New York, NY USA; 431 Academic Department of Medical Genetics, University of Cambridge, Addenbrooke’s Hospital, Cambridge, UK; 432 MRC Cancer Unit, University of Cambridge, Cambridge, UK; 433 Departments of Pediatrics and Genetics, University of North Carolina at Chapel Hill, Chapel Hill, NC USA; 434 Seven Bridges Genomics, Charlestown, MA USA; 435 Annai Systems, Inc, Carlsbad, CA USA; 436 Department of Pathology, General Hospital of Treviso, Department of Medicine, University of Padua, Treviso, Italy; 437 Department of Computational Biology, University of Lausanne, Lausanne, Switzerland; 438 Department of Genetic Medicine and Development, University of Geneva Medical School, Geneva, CH Switzerland; 439 Swiss Institute of Bioinformatics, University of Geneva, Geneva, CH Switzerland; 440 The Francis Crick Institute, London, UK; 441 University of Leuven, Leuven, Belgium; 442 Institute of Medical Genetics and Applied Genomics, University of Tübingen, Tübingen, Germany; 443 Computational and Systems Biology, Genome Institute of Singapore, Singapore, Singapore; 444 School of Computing, National University of Singapore, Singapore, Singapore; 445 Big Data Institute, Li Ka Shing Centre, University of Oxford, Oxford, UK; 446 Biomedical Data Science Laboratory, Francis Crick Institute, London, UK; 447 Bioinformatics Group, Department of Computer Science, University College London, London, UK; 448 The Edward S. Rogers Sr. Department of Electrical and Computer Engineering, University of Toronto, Toronto, ON Canada; 449 Breast Cancer Translational Research Laboratory JC Heuson, Institut Jules Bordet, Brussels, Belgium; 450 Department of Oncology, Laboratory for Translational Breast Cancer Research, KU Leuven, Leuven, Belgium; 451 Institute for Research in Biomedicine (IRB Barcelona), The Barcelona Institute of Science and Technology, Barcelona, Spain; 452 Research Program on Biomedical Informatics, Universitat Pompeu Fabra, Barcelona, Spain; 453 Division of Medical Oncology, Princess Margaret Cancer Centre, Toronto, ON Canada; 454 Department of Physiology and Biophysics, Weill Cornell Medicine, New York, NY USA; 455 Institute for Computational Biomedicine, Weill Cornell Medicine, New York, NY USA; 456 Department of Pathology, UPMC Shadyside, Pittsburgh, PA USA; 457 Independent Consultant, Wellesley, USA; 458 Department of Cell and Molecular Biology, Science for Life Laboratory, Uppsala University, Uppsala, Sweden; 459 Department of Medicine and Department of Genetics, Washington University School of Medicine, St. Louis, St. Louis, MO USA; 460 Hefei University of Technology, Anhui, China; 461 Translational Cancer Research Unit, GZA Hospitals St.-Augustinus, Center for Oncological Research, Faculty of Medicine and Health Sciences, University of Antwerp, Antwerp, Belgium; 462 Simon Fraser University, Burnaby, BC Canada; 463 University of Pennsylvania, Philadelphia, PA USA; 464 Faculty of Science and Technology, University of Vic—Central University of Catalonia (UVic-UCC), Vic, Spain; 465 The Wellcome Trust, London, UK; 466 The Hospital for Sick Children, Toronto, ON Canada; 467 Department of Pathology, Queen Elizabeth University Hospital, Glasgow, UK; 468 Department of Genetics and Computational Biology, QIMR Berghofer Medical Research Institute, Brisbane, QLD Australia; 469 Department of Oncology, Centre for Cancer Genetic Epidemiology, University of Cambridge, Cambridge, UK; 470 Department of Public Health and Primary Care, Centre for Cancer Genetic Epidemiology, University of Cambridge, Cambridge, UK; 471 Prostate Cancer Canada, Toronto, ON Canada; 472 University of Cambridge, Cambridge, UK; 473 Department of Laboratory Medicine, Translational Cancer Research, Lund University Cancer Center at Medicon Village, Lund University, Lund, Sweden; 474 Heidelberg University, Heidelberg, Germany; 475 New BIH Digital Health Center, Berlin Institute of Health (BIH) and Charité - Universitätsmedizin Berlin, Berlin, Germany; 476 CIBER Epidemiología y Salud Pública (CIBERESP), Madrid, Spain; 477 Research Group on Statistics, Econometrics and Health (GRECS), UdG, Barcelona, Spain; 478 Quantitative Genomics Laboratories (qGenomics), Barcelona, Spain; 479 Icelandic Cancer Registry, Icelandic Cancer Society, Reykjavik, Iceland; 480 State Key Laboratory of Cancer Biology, and Xijing Hospital of Digestive Diseases, Fourth Military Medical University, Shaanxi, China; 481 Department of Medicine (DIMED), Surgical Pathology Unit, University of Padua, Padua, Italy; 482 Rigshospitalet, Copenhagen, Denmark; 483 Center for Cancer Genomics, National Cancer Institute, National Institutes of Health, Bethesda, MD USA; 484 Department of Biochemistry and Molecular Medicine, University of Montreal, Montreal, QC Canada; 485 Australian Institute of Tropical Health and Medicine, James Cook University, Douglas, QLD Australia; 486 Department of Neuro-Oncology, Istituto Neurologico Besta, Milano, Italy; 487 Bioplatforms Australia, North Ryde, NSW Australia; 488 Department of Pathology (Research), University College London Cancer Institute, London, UK; 489 Department of Surgical Oncology, Princess Margaret Cancer Centre, Toronto, ON Canada; 490 Department of Medical Oncology, Josephine Nefkens Institute and Cancer Genomics Centre, Erasmus Medical Center, Rotterdam, CN The Netherlands; 491 The University of Queensland Thoracic Research Centre, The Prince Charles Hospital, Brisbane, QLD Australia; 492 CIBIO/InBIO - Research Center in Biodiversity and Genetic Resources, Universidade do Porto, Vairão, Portugal; 493 HCA Laboratories, London, UK; 494 University of Liverpool, Liverpool, UK; 495 The Azrieli Faculty of Medicine, Bar-Ilan University, Safed, Israel; 496 Department of Neurosurgery, University of Florida, Gainesville, FL USA; 497 Department of Pathology, Graduate School of Medicine, University of Tokyo, Tokyo, Japan; 498 University of Milano Bicocca, Monza, Italy; 499 BGI-Shenzhen, Shenzhen, China; 500 Department of Pathology, Oslo University Hospital Ulleval, Oslo, Norway; 501 Center for Biomedical Informatics, Harvard Medical School, Boston, MA USA; 502 Department Biochemistry and Molecular Biomedicine, University of Barcelona, Barcelona, Spain; 503 Office of Cancer Genomics, National Cancer Institute, National Institutes of Health, Bethesda, MD USA; 504 Cancer Epigenomics, German Cancer Research Center (DKFZ), Heidelberg, Germany; 505 Department of Cancer Biology, The University of Texas MD Anderson Cancer Center, Houston, TX USA; 506 Department of Surgical Oncology, The University of Texas MD Anderson Cancer Center, Houston, TX USA; 507 Department of Computer Science, Yale University, New Haven, CT USA; 508 Department of Molecular Biophysics and Biochemistry, Yale University, New Haven, CT USA; 509 Program in Computational Biology and Bioinformatics, Yale University, New Haven, CT USA; 510 Center for Cancer Research, Massachusetts General Hospital, Boston, MA USA; 511 Department of Pathology, Massachusetts General Hospital, Boston, MA USA; 512 Department of Pathology, Memorial Sloan Kettering Cancer Center, New York, NY USA; 513 Division of Gastroenterology and Hepatology, Mayo Clinic, Rochester, MN USA; 514 University of Sydney, Sydney, NSW Australia; 515 University of Oxford, Oxford, UK; 516 Department of Surgery, Academic Urology Group, University of Cambridge, Cambridge, UK; 517 Department of Medicine II, University of Würzburg, Wuerzburg, Germany; 518 Sylvester Comprehensive Cancer Center, University of Miami, Miami, FL USA; 519 Institut Hospital del Mar d’Investigacions Mèdiques (IMIM), Barcelona, Spain; 520 Genome Integrity and Structural Biology Laboratory, National Institute of Environmental Health Sciences (NIEHS), Durham, NC USA; 521 St. Thomas’s Hospital, London, UK; 522 Osaka International Cancer Center, Osaka, Japan; 523 Department of Pathology, Skåne University Hospital, Lund University, Lund, Sweden; 524 Department of Medical Oncology, Beatson West of Scotland Cancer Centre, Glasgow, UK; 525 National Human Genome Research Institute, National Institutes of Health, Bethesda, MD USA; 526 Centre for Cancer Research, Victorian Comprehensive Cancer Centre, University of Melbourne, Melbourne, VIC Australia; 527 Department of Medicine, Section of Hematology/Oncology, University of Chicago, Chicago, IL USA; 528 German Center for Infection Research (DZIF), Partner Site Hamburg-Borstel-Lübeck-Riems, Hamburg, Germany; 529 Bioinformatics Research Centre (BiRC), Aarhus University, Aarhus, Denmark; 530 Department of Biotechnology, Ministry of Science and Technology, Government of India, New Delhi, Delhi India; 531 National Cancer Centre Singapore, Singapore, Singapore; 532 Brandeis University, Waltham, MA USA; 533 Department of Urologic Sciences, University of British Columbia, Vancouver, BC Canada; 534 Department of Internal Medicine, Stanford University, Stanford, CA USA; 535 The University of Texas Health Science Center at Houston, Houston, TX USA; 536 Imperial College NHS Trust, Imperial College, London, INY UK; 537 Senckenberg Institute of Pathology, University of Frankfurt Medical School, Frankfurt, Germany; 538 Department of Medicine, Division of Biomedical Informatics, UC San Diego School of Medicine, San Diego, CA USA; 539 Center for Precision Health, School of Biomedical Informatics, The University of Texas Health Science Center, Houston, TX USA; 540 Oxford Nanopore Technologies, New York, NY USA; 541 Institute of Medical Science, University of Tokyo, Tokyo, Japan; 542 Howard Hughes Medical Institute, University of California Santa Cruz, Santa Cruz, CA USA; 543 Wakayama Medical University, Wakayama, Japan; 544 Department of Internal Medicine, Division of Medical Oncology, Lineberger Comprehensive Cancer Center, University of North Carolina at Chapel Hill, Chapel Hill, NC USA; 545 University of Tennessee Health Science Center for Cancer Research, Memphis, TN USA; 546 Department of Histopathology, Salford Royal NHS Foundation Trust, Salford, UK; 547 Faculty of Biology, Medicine and Health, University of Manchester, Manchester, UK; 548 BIOPIC, ICG and College of Life Sciences, Peking University, Beijing, China; 549 Peking-Tsinghua Center for Life Sciences, Peking University, Beijing, China; 550 Children’s Hospital of Philadelphia, Philadelphia, PA USA; 551 Department of Bioinformatics and Computational Biology and Department of Systems Biology, The University of Texas MD Anderson Cancer Center, Houston, TX USA; 552 Karolinska Institute, Stockholm, Sweden; 553 The Donnelly Centre, University of Toronto, Toronto, ON Canada; 554 Department of Medical Genetics, College of Medicine, Hallym University, Chuncheon, South Korea; 555 Department of Experimental and Health Sciences, Institute of Evolutionary Biology (UPF-CSIC), Universitat Pompeu Fabra, Barcelona, Spain; 556 Health Data Science Unit, University Clinics, Heidelberg, Germany; 557 Massachusetts General Hospital Center for Cancer Research, Charlestown, MA USA; 558 Hokkaido University, Sapporo, Japan; 559 Department of Pathology and Clinical Laboratory, National Cancer Center Hospital, Tokyo, Japan; 560 Department of Genetics, University of North Carolina at Chapel Hill, Chapel Hill, NC USA; 561 Computational Biology, Leibniz Institute on Aging - Fritz Lipmann Institute (FLI), Jena, Germany; 562 University of Melbourne Centre for Cancer Research, Melbourne, VIC Australia; 563 University of Nebraska Medical Center, Omaha, NE USA; 564 Syntekabio Inc, Daejeon, South Korea; 565 Department of Pathology, Academic Medical Center, Amsterdam, AZ The Netherlands; 566 China National GeneBank-Shenzhen, Shenzhen, China; 567 Division of Molecular Genetics, German Cancer Research Center (DKFZ), Heidelberg, Germany; 568 Division of Life Science and Applied Genomics Center, Hong Kong University of Science and Technology, Clear Water Bay, Hong Kong, China; 569 Icahn School of Medicine at Mount Sinai, New York, NY USA; 570 Geneplus-Shenzhen, Shenzhen, China; 571 School of Computer Science and Technology, Xi’an Jiaotong University, Xi’an, China; 572 AbbVie, North Chicago, IL USA; 573 Institute of Pathology, Charité – University Medicine Berlin, Berlin, Germany; 574 Centre for Translational and Applied Genomics, British Columbia Cancer Agency, Vancouver, BC Canada; 575 Edinburgh Royal Infirmary, Edinburgh, UK; 576 Berlin Institute for Medical Systems Biology, Max Delbrück Center for Molecular Medicine, Berlin, Germany; 577 Department of Pediatric Immunology, Hematology and Oncology, University Hospital, Heidelberg, Germany; 578 German Cancer Research Center (DKFZ), Heidelberg, Germany; 579 Heidelberg Institute for Stem Cell Technology and Experimental Medicine (HI-STEM), Heidelberg, Germany; 580 Institute for Computational Biomedicine, Weill Cornell Medical College, New York, NY USA; 581 New York Genome Center, New York, NY USA; 582 Department of Urology, James Buchanan Brady Urological Institute, Johns Hopkins University School of Medicine, Baltimore, MD USA; 583 Department of Preventive Medicine, Graduate School of Medicine, The University of Tokyo, Tokyo, Japan; 584 Department of Molecular and Cellular Biology, Baylor College of Medicine, Houston, TX USA; 585 Department of Pathology and Immunology, Baylor College of Medicine, Houston, TX USA; 586 Michael E. DeBakey Veterans Affairs Medical Center, Houston, TX USA; 587 Technical University of Denmark, Lyngby, Denmark; 588 Department of Pathology, College of Medicine, Hanyang University, Seoul, South Korea; 589 Academic Unit of Surgery, School of Medicine, College of Medical, Veterinary and Life Sciences, University of Glasgow, Glasgow Royal Infirmary, Glasgow, UK; 590 Department of Pathology, Asan Medical Center, College of Medicine, Ulsan University, Songpa-gu, Seoul South Korea; 591 Science Writer, Garrett Park, MD USA; 592 International Cancer Genome Consortium (ICGC)/ICGC Accelerating Research in Genomic Oncology (ARGO) Secretariat, Ontario Institute for Cancer Research, Toronto, ON Canada; 593 University of Ljubljana, Ljubljana, Slovenia; 594 Department of Public Health Sciences, University of Chicago, Chicago, IL USA; 595 Research Institute, NorthShore University HealthSystem, Evanston, IL USA; 596 Department for Biomedical Research, University of Bern, Bern, Switzerland; 597 Centre of Genomics and Policy, McGill University and Génome Québec Innovation Centre, Montreal, QC Canada; 598 Carolina Center for Genome Sciences, University of North Carolina at Chapel Hill, Chapel Hill, NC USA; 599 Hopp Children’s Cancer Center (KiTZ), Heidelberg, Germany; 600 Pediatric Glioma Research Group, German Cancer Research Center (DKFZ), Heidelberg, Germany; 601 Cancer Research UK, London, UK; 602 Indivumed GmbH, Hamburg, Germany; 603 Genome Integration Data Center, Syntekabio, Inc, Daejeon, South Korea; 604 University Hospital Zurich, Zurich, Switzerland; 605 Clinical Bioinformatics, Swiss Institute of Bioinformatics, Geneva, Switzerland; 606 Institute for Pathology and Molecular Pathology, University Hospital Zurich, Zurich, Switzerland; 607 Institute of Molecular Life Sciences, University of Zurich, Zurich, Switzerland; 608 MRC Human Genetics Unit, MRC IGMM, University of Edinburgh, Edinburgh, UK; 609 Women’s Cancer Program at the Samuel Oschin Comprehensive Cancer Institute, Cedars-Sinai Medical Center, Los Angeles, CA USA; 610 Department of Biology, Bioinformatics Group, Division of Molecular Biology, Faculty of Science, University of Zagreb, Zagreb, Croatia; 611 Department for Internal Medicine II, University Hospital Schleswig-Holstein, Kiel, Germany; 612 Genetics and Molecular Pathology, SA Pathology, Adelaide, SA Australia; 613 Department of Gastric Surgery, National Cancer Center Hospital, Tokyo, Japan; 614 Department of Bioinformatics, Division of Cancer Genomics, National Cancer Center Research Institute, Tokyo, Japan; 615 A.A. Kharkevich Institute of Information Transmission Problems, Moscow, Russia; 616 Oncology and Immunology, Dmitry Rogachev National Research Center of Pediatric Hematology, Moscow, Russia; 617 Skolkovo Institute of Science and Technology, Moscow, Russia; 618 Department of Surgery, The George Washington University, School of Medicine and Health Science, Washington, DC USA; 619 Endocrine Oncology Branch, Center for Cancer Research, National Cancer Institute, National Institutes of Health, Bethesda, MD USA; 620 Melanoma Institute Australia, Macquarie University, Sydney, NSW Australia; 621 MIT Computer Science and Artificial Intelligence Laboratory, Massachusetts Institute of Technology, Cambridge, MA USA; 622 Tissue Pathology and Diagnostic Oncology, Royal Prince Alfred Hospital, Sydney, NSW Australia; 623 Cholangiocarcinoma Screening and Care Program and Liver Fluke and Cholangiocarcinoma Research Centre, Faculty of Medicine, Khon Kaen University, Khon Kaen, Thailand; 624 Controlled Department and Institution, New York, NY USA; 625 Englander Institute for Precision Medicine, Weill Cornell Medicine, New York, NY USA; 626 National Cancer Center, Gyeonggi, South Korea; 627 Department of Biochemistry, College of Medicine, Ewha Womans University, Seoul, South Korea; 628 Health Sciences Department of Biomedical Informatics, University of California San Diego, La Jolla, CA USA; 629 Research Core Center, National Cancer Centre Korea, Goyang-si, South Korea; 630 Department of Health Sciences and Technology, Sungkyunkwan University School of Medicine, Seoul, South Korea; 631 Samsung Genome Institute, Seoul, South Korea; 632 Breast Oncology Program, Dana-Farber/Brigham and Women’s Cancer Center, Boston, MA USA; 633 Department of Surgery, Memorial Sloan Kettering Cancer Center, New York, NY USA; 634 Division of Breast Surgery, Brigham and Women’s Hospital, Boston, MA USA; 635 Integrative Bioinformatics Support Group, National Institute of Environmental Health Sciences (NIEHS), Durham, NC USA; 636 Department of Clinical Science, University of Bergen, Bergen, Norway; 637 Center For Medical Innovation, Seoul National University Hospital, Seoul, South Korea; 638 Department of Internal Medicine, Seoul National University Hospital, Seoul, South Korea; 639 Institute of Computer Science, Polish Academy of Sciences, Warsawa, Poland; 640 Functional and Structural Genomics, German Cancer Research Center (DKFZ), Heidelberg, Germany; 641 Laboratory of Translational Genomics, Division of Cancer Epidemiology and Genetics, National Cancer Institute, , National Institutes of Health, Bethesda, MD USA; 642 Institute for Medical Informatics Statistics and Epidemiology, University of Leipzig, Leipzig, Germany; 643 Morgan Welch Inflammatory Breast Cancer Research Program and Clinic, The University of Texas MD Anderson Cancer Center, Houston, TX USA; 644 Department of Hematology and Oncology, Georg-Augusts-University of Göttingen, Göttingen, Germany; 645 Institute of Cell Biology (Cancer Research), University of Duisburg-Essen, Essen, Germany; 646 King’s College London and Guy’s and St. Thomas’ NHS Foundation Trust, London, UK; 647 Center for Epigenetics, Van Andel Research Institute, Grand Rapids, MI USA; 648 The University of Queensland Centre for Clinical Research, Royal Brisbane and Women’s Hospital, Herston, QLD Australia; 649 Department of Pediatric Oncology and Hematology, University of Cologne, Cologne, Germany; 650 University of Düsseldorf, Düsseldorf, Germany; 651 Department of Pathology, Institut Jules Bordet, Brussels, Belgium; 652 Institute of Biomedicine, Sahlgrenska Academy at University of Gothenburg, Gothenburg, Sweden; 653 Children’s Medical Research Institute, Sydney, NSW Australia; 654 ILSbio, LLC Biobank, Chestertown, MD USA; 655 Division of Genetics and Genomics, Boston Children’s Hospital, Harvard Medical School, Boston, MA USA; 656 Institute for Bioengineering and Biopharmaceutical Research (IBBR), Hanyang University, Seoul, South Korea; 657 Department of Statistics, University of California Santa Cruz, Santa Cruz, CA USA; 658 National Genotyping Center, Institute of Biomedical Sciences, Academia Sinica, Taipei, Taiwan; 659 Department of Vertebrate Genomics/Otto Warburg Laboratory Gene Regulation and Systems Biology of Cancer, Max Planck Institute for Molecular Genetics, Berlin, Germany; 660 McGill University and Genome Quebec Innovation Centre, Montreal, QC Canada; 661 biobyte solutions GmbH, Heidelberg, Germany; 662 Gynecologic Oncology, NYU Laura and Isaac Perlmutter Cancer Center, New York University, New York, NY USA; 663 Division of Oncology, Stem Cell Biology Section, Washington University School of Medicine, St. Louis, MO USA; 664 Department of Systems Biology, The University of Texas MD Anderson Cancer Center, Houston, TX USA; 665 Harvard University, Cambridge, MA USA; 666 Urologic Oncology Branch, Center for Cancer Research, National Cancer Institute, National Institutes of Health, Bethesda, MD USA; 667 University of Oslo, Oslo, Norway; 668 University of Toronto, Toronto, ON Canada; 669 Peking University, Beijing, China; 670 School of Life Sciences, Peking University, Beijing, China; 671 Leidos Biomedical Research, Inc, McLean, VA USA; 672 Hematology, Hospital Clinic, Institut d’Investigacions Biomèdiques August Pi i Sunyer (IDIBAPS), University of Barcelona, Barcelona, Spain; 673 Second Military Medical University, Shanghai, China; 674 Chinese Cancer Genome Consortium, Shenzhen, China; 675 Department of Medical Oncology, Beijing Hospital, Beijing, China; 676 Laboratory of Molecular Oncology, Key Laboratory of Carcinogenesis and Translational Research (Ministry of Education), Peking University Cancer Hospital and Institute, Beijing, China; 677 School of Medicine/School of Mathematics and Statistics, University of St. Andrews, St, Andrews, Fife UK; 678 Institute for Systems Biology, Seattle, WA USA; 679 Department of Biochemistry and Molecular Biology, Faculty of Medicine, University Institute of Oncology-IUOPA, Oviedo, Spain; 680 Institut Bergonié, Bordeaux, France; 681 Cancer Unit, MRC University of Cambridge, Cambridge, UK; 682 Department of Pathology and Laboratory Medicine, Center for Personalized Medicine, Children’s Hospital Los Angeles, Los Angeles, CA USA; 683 John Curtin School of Medical Research, Canberra, ACT Australia; 684 MVZ Department of Oncology, PraxisClinic am Johannisplatz, Leipzig, Germany; 685 Department of Information Technology, Ghent University, Ghent, Belgium; 686 Department of Plant Biotechnology and Bioinformatics, Ghent University, Ghent, Belgium; 687 Institute for Genomic Medicine, Nationwide Children’s Hospital, Columbus, OH USA; 688 Computational Biology Program, School of Medicine, Oregon Health and Science University, Portland, OR USA; 689 Department of Surgery, Duke University, Durham, NC USA; 690 Institució Catalana de Recerca i Estudis Avançats (ICREA), Barcelona, Spain; 691 Institut Català de Paleontologia Miquel Crusafont, Universitat Autònoma de Barcelona, Barcelona, Spain; 692 University of Glasgow, Glasgow, UK; 693 Institut d’Investigacions Biomèdiques August Pi i Sunyer (IDIBAPS), Barcelona, Spain; 694 Division of Oncology, Washington University School of Medicine, St. Louis, MO USA; 695 Department of Surgery and Cancer, Imperial College, London, INY UK; 696 Applications Department, Oxford Nanopore Technologies, Oxford, UK; 697 Department of Obstetrics, Gynecology and Reproductive Services, University of California San Francisco, San Francisco, CA USA; 698 Department of Biochemistry and Molecular Medicine, University California at Davis, Sacramento, CA USA; 699 STTARR Innovation Facility, Princess Margaret Cancer Centre, Toronto, ON Canada; 700 Discipline of Surgery, Western Sydney University, Penrith, NSW Australia; 701 Yale School of Medicine, Yale University, New Haven, CT USA; 702 Department of Genetics, Lineberger Comprehensive Cancer Center, University of North Carolina at Chapel Hill, Chapel Hill, NC USA; 703 Departments of Neurology and Neurosurgery, Henry Ford Hospital, Detroit, MI USA; 704 Precision Oncology, OHSU Knight Cancer Institute, Oregon Health and Science University, Portland, OR USA; 705 Institute of Pathology, University Medical Center Hamburg-Eppendorf, Hamburg, Germany; 706 Department of Health Sciences, Faculty of Medical Sciences, Kyushu University, Fukuoka, Japan; 707 Heidelberg Academy of Sciences and Humanities, Heidelberg, Germany; 708 Department of Clinical Pathology, University of Melbourne, Melbourne, VIC, Australia; 709 Department of Pathology, Roswell Park Cancer Institute, Buffalo, NY USA; 710 Department of Computer Science, University of Helsinki, Helsinki, Finland; 711 Institute of Biotechnology, University of Helsinki, Helsinki, Finland; 712 Organismal and Evolutionary Biology Research Programme, University of Helsinki, Helsinki, Finland; 713 Department of Obstetrics and Gynecology, Division of Gynecologic Oncology, Washington University School of Medicine, St. Louis, MO USA; 714 Penrose St. Francis Health Services, Colorado Springs, CO USA; 715 Institute of Pathology, Ulm University and University Hospital of Ulm, Ulm, Germany; 716 National Cancer Center, Tokyo, Japan; 717 Genome Institute of Singapore, Singapore, Singapore; 718 32Program in Computational Biology and Bioinformatics, Yale University, New Haven, CT USA; 719 German Cancer Aid, Bonn, Germany; 720 Programme in Cancer and Stem Cell Biology, Centre for Computational Biology, Duke-NUS Medical School, Singapore, Singapore; 721 The Chinese University of Hong Kong, Shatin, NT, Hong Kong China; 722 Fourth Military Medical University, Shaanxi, China; 723 The University of Cambridge School of Clinical Medicine, Cambridge, UK; 724 St. Jude Children’s Research Hospital, Memphis, TN USA; 725 University Health Network, Princess Margaret Cancer Centre, Toronto, ON Canada; 726 Center for Biomolecular Science and Engineering, University of California Santa Cruz, Santa Cruz, CA USA; 727 Department of Medicine, University of Chicago, Chicago, IL USA; 728 Department of Neurology, Mayo Clinic, Rochester, MN USA; 729 Cambridge Oesophagogastric Centre, Cambridge University Hospitals NHS Foundation Trust, Cambridge, UK; 730 Department of Computer Science, Carleton College, Northfield, MN USA; 731 Institute of Cancer Sciences, College of Medical Veterinary and Life Sciences, University of Glasgow, Glasgow, UK; 732 Department of Epidemiology, University of Alabama at Birmingham, Birmingham, AL USA; 733 HudsonAlpha Institute for Biotechnology, Huntsville, AL USA; 734 O’Neal Comprehensive Cancer Center, University of Alabama at Birmingham, Birmingham, AL USA; 735 Department of Pathology, Keio University School of Medicine, Tokyo, Japan; 736 Department of Hepatobiliary and Pancreatic Oncology, National Cancer Center Hospital, Tokyo, Japan; 737 Sage Bionetworks, Seattle, WA USA; 738 Lymphoma Genomic Translational Research Laboratory, National Cancer Centre, Singapore, Singapore; 739 Department of Clinical Pathology, Robert-Bosch-Hospital, Stuttgart, Germany; 740 Department of Cell and Systems Biology, University of Toronto, Toronto, ON Canada; 741 Department of Biosciences and Nutrition, Karolinska Institutet, Stockholm, Sweden; 742 Center for Liver Cancer, Research Institute and Hospital, National Cancer Center, Gyeonggi, South Korea; 743 Division of Hematology-Oncology, Samsung Medical Center, Sungkyunkwan University School of Medicine, Seoul, South Korea; 744 Samsung Advanced Institute for Health Sciences and Technology, Sungkyunkwan University School of Medicine, Seoul, South Korea; 745 Cheonan Industry-Academic Collaboration Foundation, Sangmyung University, Cheonan, South Korea; 746 NYU Langone Medical Center, New York, NY USA; 747 Department of Hematology and Medical Oncology, Cleveland Clinic, Cleveland, OH USA; 748 Department of Radiation Oncology, University of California San Francisco, San Francisco, CA USA; 749 Department of Health Sciences Research, Mayo Clinic, Rochester, MN USA; 750 Helen F. Graham Cancer Center at Christiana Care Health Systems, Newark, DE USA; 751 Heidelberg University Hospital, Heidelberg, Germany; 752 CSRA Incorporated, Fairfax, VA USA; 753 Research Department of Pathology, University College London Cancer Institute, London, UK; 754 Department of Research Oncology, Guy’s Hospital, King’s Health Partners AHSC, King’s College London School of Medicine, London, UK; 755 Faculty of Medicine and Health Sciences, Macquarie University, Sydney, NSW Australia; 756 University Hospital of Minjoz, INSERM UMR 1098, Besançon, France; 757 Spanish National Cancer Research Centre, Madrid, Spain; 758 Center of Digestive Diseases and Liver Transplantation, Fundeni Clinical Institute, Bucharest, Romania; 759 Cureline, Inc, South San Francisco, CA USA; 760 St. Luke’s Cancer Centre, Royal Surrey County Hospital NHS Foundation Trust, Guildford, UK; 761 Cambridge Breast Unit, Addenbrooke’s Hospital, Cambridge University Hospital NHS Foundation Trust and NIHR Cambridge Biomedical Research Centre, Cambridge, UK; 762 East of Scotland Breast Service, Ninewells Hospital, Aberdeen, UK; 763 Department of Genetics, Microbiology and Statistics, University of Barcelona, IRSJD, IBUB, Barcelona, Spain; 764 Department of Obstetrics and Gynecology, Medical College of Wisconsin, Milwaukee, WI USA; 765 Hematology and Medical Oncology, Winship Cancer Institute of Emory University, Atlanta, GA USA; 766 Department of Computer Science, Princeton University, Princeton, NJ USA; 767 Vanderbilt Ingram Cancer Center, Vanderbilt University, Nashville, TN USA; 768 Ohio State University College of Medicine and Arthur G. James Comprehensive Cancer Center, Columbus, OH USA; 769 Department of Surgery, Yokohama City University Graduate School of Medicine, Kanagawa, Japan; 770 Division of Chromatin Networks, German Cancer Research Center (DKFZ) and BioQuant, Heidelberg, Germany; 771 Research Computing Center, University of North Carolina at Chapel Hill, Chapel Hill, NC USA; 772 School of Molecular Biosciences and Center for Reproductive Biology, Washington State University, Pullman, WA USA; 773 Finsen Laboratory and Biotech Research and Innovation Centre (BRIC), University of Copenhagen, Copenhagen, Denmark; 774 Department of Laboratory Medicine and Pathobiology, University of Toronto, Toronto, ON Canada; 775 Department of Pathology, Human Oncology and Pathogenesis Program, Memorial Sloan Kettering Cancer Center, New York, NY USA; 776 University Hospital Giessen, Pediatric Hematology and Oncology, Giessen, Germany; 777 Oncologie Sénologie, ICM Institut Régional du Cancer, Montpellier, France; 778 Institute of Clinical Molecular Biology, Christian-Albrechts-University, Kiel, Germany; 779 Institute of Pathology, University of Wuerzburg, Wuerzburg, Germany; 780 Department of Urology, North Bristol NHS Trust, Bristol, UK; 781 SingHealth, Duke-NUS Institute of Precision Medicine, National Heart Centre Singapore, Singapore, Singapore; 782 Department of Computer Science, University of Toronto, Toronto, ON Canada; 783 Bern Center for Precision Medicine, University Hospital of Bern, University of Bern, Bern, Switzerland; 784 Englander Institute for Precision Medicine, Weill Cornell Medicine and New York Presbyterian Hospital, New York, NY USA; 785 Meyer Cancer Center, Weill Cornell Medicine, New York, NY USA; 786 Pathology and Laboratory, Weill Cornell Medical College, New York, NY USA; 787 Vall d’Hebron Institute of Oncology: VHIO, Barcelona, Spain; 788 General and Hepatobiliary-Biliary Surgery, Pancreas Institute, University and Hospital Trust of Verona, Verona, Italy; 789 National Centre for Biological Sciences, Tata Institute of Fundamental Research, Bangalore, India; 790 Indiana University, Bloomington, IN USA; 791 Department of Pathology, GZA-ZNA Hospitals, Antwerp, Belgium; 792 Analytical Biological Services, Inc, Wilmington, DE USA; 793 Sydney Medical School, University of Sydney, Sydney, NSW Australia; 794 cBio Center, Dana-Farber Cancer Institute, Harvard Medical School, Boston, MA USA; 795 Department of Cell Biology, Harvard Medical School, Boston, MA USA; 796 Advanced Centre for Treatment Research and Education in Cancer, Tata Memorial Centre, Navi Mumbai, Maharashtra India; 797 School of Environmental and Life Sciences, Faculty of Science, The University of Newcastle, Ourimbah, NSW Australia; 798 Department of Dermatology, University Hospital of Essen, Essen, Germany; 799 Bioinformatics and Omics Data Analytics, German Cancer Research Center (DKFZ), Heidelberg, Germany; 800 Department of Urology, Charité Universitätsmedizin Berlin, Berlin, Germany; 801 Martini-Clinic, Prostate Cancer Center, University Medical Center Hamburg-Eppendorf, Hamburg, Germany; 802 Department of General Internal Medicine, University of Kiel, Kiel, Germany; 803 German Cancer Consortium (DKTK), Partner site Berlin, Berlin, Germany; 804 Cancer Research Institute, Beth Israel Deaconess Medical Center, Boston, MA USA; 805 University of Pittsburgh, Pittsburgh, PA USA; 806 Department of Ophthalmology and Ocular Genomics Institute, Massachusetts Eye and Ear, Harvard Medical School, Boston, MA USA; 807 Center for Psychiatric Genetics, NorthShore University HealthSystem, Evanston, IL USA; 808 Van Andel Research Institute, Grand Rapids, MI USA; 809 Laboratory of Molecular Medicine, Human Genome Center, Institute of Medical Science, University of Tokyo, Tokyo, Japan; 810 Japan Agency for Medical Research and Development, Tokyo, Japan; 811 Korea University, Seoul, South Korea; 812 Murtha Cancer Center, Walter Reed National Military Medical Center, Bethesda, MD USA; 813 Human Genetics, University of Kiel, Kiel, Germany; 814 Department of Oncologic Pathology, Dana-Farber Cancer Institute, Harvard Medical School, Boston, MA USA; 815 Oregon Health and Science University, Portland, OR USA; 816 Center for RNA Interference and Noncoding RNA, The University of Texas MD Anderson Cancer Center, Houston, TX USA; 817 Department of Experimental Therapeutics, The University of Texas MD Anderson Cancer Center, Houston, TX USA; 818 Department of Gynecologic Oncology and Reproductive Medicine, The University of Texas MD Anderson Cancer Center, Houston, TX USA; 819 University Hospitals Coventry and Warwickshire NHS Trust, Coventry, UK; 820 Department of Radiation Oncology, Radboud University Nijmegen Medical Centre, Nijmegen, GA The Netherlands; 821 Institute for Genomics and Systems Biology, University of Chicago, Chicago, IL USA; 822 Clinic for Hematology and Oncology, St.-Antonius-Hospital, Eschweiler, Germany; 823 Computational and Systems Biology Program, Memorial Sloan Kettering Cancer Center, New York, NY USA; 824 University of Iceland, Reykjavik, Iceland; 825 Division of Computational Genomics and Systems Genetics, German Cancer Research Center (DKFZ), Heidelberg, Germany; 826 Dundee Cancer Centre, Ninewells Hospital, Dundee, UK; 827 Department for Internal Medicine III, University of Ulm and University Hospital of Ulm, Ulm, Germany; 828 Institut Curie, INSERM Unit 830, Paris, France; 829 Department of Gastroenterology and Hepatology, Yokohama City University Graduate School of Medicine, Kanagawa, Japan; 830 Department of Laboratory Medicine, Radboud University Nijmegen Medical Centre, Nijmegen, GA The Netherlands; 831 Division of Cancer Genome Research, German Cancer Research Center (DKFZ), Heidelberg, Germany; 832 Department of General Surgery, Singapore General Hospital, Singapore, Singapore; 833 Cancer Science Institute of Singapore, National University of Singapore, Singapore, Singapore; 834 Department of Medical and Clinical Genetics, Genome-Scale Biology Research Program, University of Helsinki, Helsinki, Finland; 835 East Anglian Medical Genetics Service, Cambridge University Hospitals NHS Foundation Trust, Cambridge, UK; 836 Irving Institute for Cancer Dynamics, Columbia University, New York, NY USA; 837 Institute of Molecular and Cell Biology, Singapore, Singapore; 838 Laboratory of Cancer Epigenome, Division of Medical Science, National Cancer Centre Singapore, Singapore, Singapore; 839 Universite Lyon, INCa-Synergie, Centre Léon Bérard, Lyon, France; 840 Department of Urology, Mayo Clinic, Rochester, MN USA; 841 Royal National Orthopaedic Hospital - Stanmore, Stanmore, Middlesex UK; 842 Department of Biochemistry, Genetics and Immunology, University of Vigo, Vigo, Spain; 843 Giovanni Paolo II / I.R.C.C.S. Cancer Institute, Bari, BA Italy; 844 Neuroblastoma Genomics, German Cancer Research Center (DKFZ), Heidelberg, Germany; 845 Fondazione Policlinico Universitario Gemelli IRCCS, Rome, Italy, Rome, Italy; 846 University of Verona, Verona, Italy; 847 Centre National de Génotypage, CEA - Institute de Génomique, Evry, France; 848 CAPHRI Research School, Maastricht University, Maastricht, ER The Netherlands; 849 Department of Biopathology, Centre Léon Bérard, Lyon, France; 850 Université Claude Bernard Lyon 1, Villeurbanne, France; 851 Core Research for Evolutional Science and Technology (CREST), JST, Tokyo, Japan; 852 Department of Biological Sciences, Laboratory for Medical Science Mathematics, Graduate School of Science, University of Tokyo, Yokohama, Japan; 853 Department of Medical Science Mathematics, Medical Research Institute, Tokyo Medical and Dental University (TMDU), Tokyo, Japan; 854 Cancer Ageing and Somatic Mutation Programme, Wellcome Sanger Institute, Hinxton, UK; 855 University Hospitals Birmingham NHS Foundation Trust, Birmingham, UK; 856 Centre for Cancer Research and Cell Biology, Queen’s University, Belfast, UK; 857 Breast Medical Oncology, The University of Texas MD Anderson Cancer Center, Houston, TX USA; 858 Department of Surgery, Johns Hopkins University School of Medicine, Baltimore, MD USA; 859 Department of Oncology-Pathology, Science for Life Laboratory, Karolinska Institute, Stockholm, Sweden; 860 School of Cancer Sciences, Faculty of Medicine, University of Southampton, Southampton, UK; 861 Department of Gene Technology, Tallinn University of Technology, Tallinn, Estonia; 862 Genetics and Genome Biology Program, SickKids Research Institute, The Hospital for Sick Children, Toronto, ON Canada; 863 Departments of Neurosurgery and Hematology and Medical Oncology, Winship Cancer Institute and School of Medicine, Emory University, Atlanta, GA USA; 864 Department of Clinical and Molecular Medicine, Faculty of Medicine and Health Sciences, Norwegian University of Science and Technology, Trondheim, Norway; 865 Argmix Consulting, North Vancouver, BC Canada; 866 Department of Information Technology, Ghent University, Interuniversitair Micro-Electronica Centrum (IMEC), Ghent, Belgium; 867 Nuffield Department of Surgical Sciences, John Radcliffe Hospital, University of Oxford, Oxford, UK; 868 Institute of Mathematics and Computer Science, University of Latvia, Riga, LV Latvia; 869 Discipline of Pathology, Sydney Medical School, University of Sydney, Sydney, NSW Australia; 870 Department of Applied Mathematics and Theoretical Physics, Centre for Mathematical Sciences, University of Cambridge, Cambridge, UK; 871 Department of Epidemiology and Biostatistics, Memorial Sloan Kettering Cancer Center, New York, NY USA; 872 Department of Statistics, Columbia University, New York, NY USA; 873 Department of Immunology, Genetics and Pathology, Science for Life Laboratory, Uppsala University, Uppsala, Sweden; 874 School of Electronic and Information Engineering, Xi’an Jiaotong University, Xi’an, China; 875 Department of Histopathology, Cambridge University Hospitals NHS Foundation Trust, Cambridge, UK; 876 Oxford NIHR Biomedical Research Centre, University of Oxford, Oxford, UK; 877 Georgia Regents University Cancer Center, Augusta, GA USA; 878 Wythenshawe Hospital, Manchester, UK; 879 Department of Genetics, Washington University School of Medicine, St.Louis, MO USA; 880 Department of Biological Oceanography, Leibniz Institute of Baltic Sea Research, Rostock, Germany; 881 Wellcome Centre for Human Genetics, University of Oxford, Oxford, UK; 882 Department of Molecular and Human Genetics, Baylor College of Medicine, Houston, TX USA; 883 Thoracic Oncology Laboratory, Mayo Clinic, Rochester, MN USA; 884 Institute for Genomic Medicine, Nationwide Children’s Hospital, Columbus, OH USA; 885 Department of Obstetrics and Gynecology, Division of Gynecologic Oncology, Mayo Clinic, Rochester, MN USA; 886 International Institute for Molecular Oncology, Poznań, Poland; 887 Poznan University of Medical Sciences, Poznań, Poland; 888 Genomics and Proteomics Core Facility High Throughput Sequencing Unit, German Cancer Research Center (DKFZ), Heidelberg, Germany; 889 NCCS-VARI Translational Research Laboratory, National Cancer Centre Singapore, Singapore, Singapore; 890 Edison Family Center for Genome Sciences and Systems Biology, Washington University, St. Louis, MO USA; 891 MRC-University of Glasgow Centre for Virus Research, Glasgow, UK; 892 Department of Medical Informatics and Clinical Epidemiology, Division of Bioinformatics and Computational Biology, OHSU Knight Cancer Institute, Oregon Health and Science University, Portland, OR USA; 893 School of Electronic Information and Communications, Huazhong University of Science and Technology, Wuhan, China; 894 Department of Applied Mathematics and Statistics, Johns Hopkins University, Baltimore, MD USA; 895 Department of Cancer Genome Informatics, Graduate School of Medicine, Osaka University, Osaka, Japan; 896 Institute of Computer Science, Heidelberg University, Heidelberg, Germany; 897 School of Mathematics and Statistics, University of Sydney, Sydney, NSW Australia; 898 Ben May Department for Cancer Research, University of Chicago, Chicago, IL USA; 899 Department of Human Genetics, University of Chicago, Chicago, IL USA; 900 Tri-Institutional PhD Program in Computational Biology and Medicine, Weill Cornell Medicine, New York, NY USA; 901 The First Affiliated Hospital, Xi’an Jiaotong University, Xi’an, China; 902 Department of Medicine and Therapeutics, The Chinese University of Hong Kong, Shatin, NT, Hong Kong China; 903 Department of Biostatistics, The University of Texas MD Anderson Cancer Center, Houston, TX USA; 904 Duke-NUS Medical School, Singapore, Singapore; 905 Department of Surgery, Ruijin Hospital, Shanghai Jiaotong University School of Medicine, Shanghai, China; 906 School of Computing Science, University of Glasgow, Glasgow, UK; 907 Division of Orthopaedic Surgery, Oslo University Hospital, Oslo, Norway; 908 Eastern Clinical School, Monash University, Melbourne, VIC Australia; 909 Epworth HealthCare, Richmond, VIC Australia; 910 Department of Biostatistics and Computational Biology, Dana-Farber Cancer Institute and Harvard Medical School, Boston, MA USA; 911 Department of Biomedical Informatics, College of Medicine, The Ohio State University, Columbus, OH USA; 912 The Ohio State University Comprehensive Cancer Center (OSUCCC – James), Columbus, OH USA; 913 The University of Texas School of Biomedical Informatics (SBMI) at Houston, Houston, TX USA; 914 Department of Biostatistics, University of North Carolina at Chapel Hill, Chapel Hill, NC USA; 915 Department of Biochemistry and Molecular Genetics, Feinberg School of Medicine, Northwestern University, Chicago, IL USA; 916 Faculty of Medicine and Health, University of Sydney, Sydney, NSW Australia; 917 Department of Pathology, Erasmus Medical Center Rotterdam, Rotterdam, GD The Netherlands; 918 Division of Molecular Carcinogenesis, The Netherlands Cancer Institute, Amsterdam, CX The Netherlands; 919 Institute of Molecular Life Sciences and Swiss Institute of Bioinformatics, University of Zurich, Zurich, Switzerland

**Keywords:** Cancer genomics, Cellular signalling networks

## Abstract

The catalog of cancer driver mutations in protein-coding genes has greatly expanded in the past decade. However, non-coding cancer driver mutations are less well-characterized and only a handful of recurrent non-coding mutations, most notably *TERT* promoter mutations, have been reported. Here, as part of the ICGC/TCGA Pan-Cancer Analysis of Whole Genomes (PCAWG) Consortium, which aggregated whole genome sequencing data from 2658 cancer across 38 tumor types, we perform multi-faceted pathway and network analyses of non-coding mutations across 2583 whole cancer genomes from 27 tumor types compiled by the ICGC/TCGA PCAWG project that was motivated by the success of pathway and network analyses in prioritizing rare mutations in protein-coding genes. While few non-coding genomic elements are recurrently mutated in this cohort, we identify 93 genes harboring non-coding mutations that cluster into several modules of interacting proteins. Among these are promoter mutations associated with reduced mRNA expression in *TP53*, *TLE4*, and *TCF4*. We find that biological processes had variable proportions of coding and non-coding mutations, with chromatin remodeling and proliferation pathways altered primarily by coding mutations, while developmental pathways, including Wnt and Notch, altered by both coding and non-coding mutations. RNA splicing is primarily altered by non-coding mutations in this cohort, and samples containing non-coding mutations in well-known RNA splicing factors exhibit similar gene expression signatures as samples with coding mutations in these genes. These analyses contribute a new repertoire of possible cancer genes and mechanisms that are altered by non-coding mutations and offer insights into additional cancer vulnerabilities that can be investigated for potential therapeutic treatments.

## Abstract (web summary)

Understanding deregulation of biological pathways in cancer can provide insight into disease etiology and potential therapies. Here, as part of the PanCancer Analysis of Whole Genomes (PCAWG) consortium, the authors present pathway and network analysis of 2583 whole cancer genomes from 27 tumour types.

## Introduction

Over the past decade, cancer genome sequencing efforts such as The Cancer Genome Atlas (TCGA) have identified millions of somatic aberrations; however, the annotation and interpretation of these aberrations remain a major challenge^1^. Specifically, while some somatic aberrations occur frequently in specific cancer types, there is a “long tail” of rare aberrations that are difficult to distinguish from random passenger aberrations in modestly sized patient cohorts^2,3^. In many cancers, a significant proportion of patients do not have known driver mutations in protein-coding regions^4^, suggesting that additional driver mutations remain undiscovered. The vast majority of known driver mutations affect protein-coding regions. Only a few recurrent non-coding driver mutations, most notably mutations in the *TERT* promoter^5,6^, have been identified. In other studies, a genome-wide analysis has identified recurrent mutations in several regulatory elements, and expression quantitative trait loci (eQTLs) analysis has identified non-coding somatic mutations that correlate with gene expression changes in some cancer types^7^.

Cancer driver mutations unlock oncogenic properties of cells by altering the activity of hallmark pathways^8^. Accordingly, cancer genes have been shown to cluster in a small number of cellular pathways and interacting subnetworks^3,9^. Consequently, pathway and network analysis has proven useful for implicating infrequently mutated genes as cancer genes based on their pathway membership and physical or regulatory interactions with recurrently mutated genes^10–14^. However, the interactions between coding and non-coding driver mutations in known or novel pathways have not yet been systematically explored.

As part of the Pan-Cancer Analysis of Whole Genomes (PCAWG) project of the International Cancer Genome Consortium (ICGC)^15^, we performed pathway and network analysis of coding and non-coding somatic mutations from 2583 tumors from 27 tumor types. The PCAWG consortium curated whole-genome sequencing data from a total of 2658 cancers across 38 tumor types. In the marker paper^15^, this work provided the largest collection of uniformly processed cancer whole genomes to date with germline and somatic variants from reanalyzed sequencing data aligned to the human genome (reference build hs37d5) using standardized and highly accurate pipelines. Recent work from the PCAWG project of the ICGC reveals few recurrent non-coding drivers in analyses of individual genes and regulatory regions^16^. Here, we employ seven distinct pathways and network analysis methods and derive consensus sets of *pathway-implicated driver* (PID) genes from the predictions of these methods. Specifically, we identify a consensus set of 93 high-confidence *pathway-implicated driver genes using non-coding variants* (PID-N) and a consensus set of 87 *pathway-implicated driver genes using coding variants* (PID-C). Both sets of PID genes, particularly the PID-N set, contain rarely mutated genes that interact with well-known cancer genes, but were not identified as significantly mutated by single gene tests^16^. In total, 121 novel PID-N and PID-C genes are revealed as promising candidates, expanding the landscape of driver mutations in cancer.

We examined the relative contributions of coding and non-coding mutations in altering biological processes, finding that while chromatin remodeling and some well-known signaling and proliferation pathways are altered primarily by coding mutations, other important cancer pathways, including developmental pathways, such as Wnt and Notch, are altered by both coding and non-coding mutations in PID genes. Intriguingly, we find many non-coding mutations in PID-N genes with roles in RNA splicing, and samples with these non-coding mutations exhibit similar gene expression signatures as samples with well-known coding mutations in RNA splicing factors. Our analysis demonstrates that somatic non-coding mutations in untranslated and in cis regulatory regions constitute a complementary set of genetic perturbations with respect to coding mutations, affect several biological pathways and molecular interaction networks, and should be further investigated for their role in the onset and progression of cancer.

## Results

### The long tail of coding and non-coding driver mutations

We analyzed the genes targeted by single-nucleotide variants (SNVs) and short insertions and deletions (indels) identified by whole-genome sequencing in the 2538 ICGC PCAWG tumor samples from 27 tumor types. Our pathway and network analyses focused on a subset of 2252 tumors that excluded melanomas and lymphomas due to their atypical distributions of mutations in regulatory regions^17^. We utilized the pan-cancer driver *p*-values of single protein-coding and non-coding elements from the PCAWG Drivers and Functional Interpretation Working Group analysis^16^, including exons, promoters, untranslated regions (5′ UTR and 3′ UTR), and enhancers. This analysis integrates predictions from 16 driver discovery methods, resulting in consensus driver *p*-values for coding and non-coding elements; see ref. ^16^ for further details. The *p*-values of individual genes and non-coding elements indicate their statistical significance as drivers, according to diverse methods that account for positive selection, functional impact of mutations, regional mutation rates, and mutational processes and signatures^16^. Among protein-coding driver *p*-values of the pan-cancer cohort, 75 genes were statistically significant (FDR < 0.1; Supplementary Fig. 1) and an additional 7 genes were observed at near-significant levels (0.1 ≤ FDR < 0.25). These numbers are consistent with previous reports of a “long tail” of driver genes with few highly mutated genes and many genes with infrequent mutations across cancer types^2,18^. Non-coding mutations exhibit a similar long-tail distribution with even fewer significant genes (eight genes at FDR < 0.1 and two genes at 0.1 ≤ FDR < 0.25). No single gene has both significant or near-significant coding and non-coding driver *p*-values (FDR < 0.25), suggesting that non-coding mutations target a complementary set of genes as coding mutations.

Earlier studies have demonstrated that proteins harboring coding driver mutations interact with each other in molecular pathways and networks significantly more frequently than expected by chance^2,3,9–11,13^. We observed significant numbers of interactions between both significantly mutated coding and/or non-coding elements, suggesting that the pathway and network methods may be useful in prioritizing rare driver events that are not significant by single-element analyses (Supplementary Fig. 2; Coding and non-coding mutations cluster on networks in Supplementary file).

### Pathway and network analysis of potential driver mutations

**Fig. 1:**
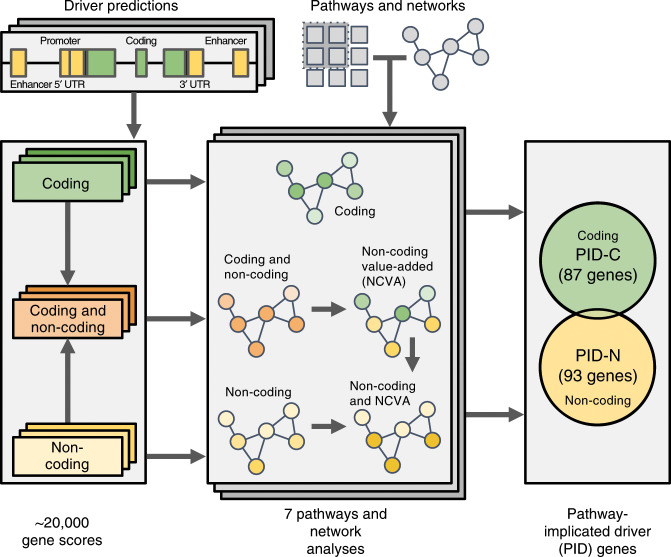
Overview of the pathway and network analysis approach. Coding, non-coding, and combined gene scores were derived for each gene by aggregating driver *p*-values from the PCAWG driver predictions in individual elements, including annotated coding and non-coding elements (promoter, 5′ UTR, 3′ UTR, and enhancer). These gene scores were input to five network analysis algorithms (CanIsoNet^20^, Hierarchical HotNet^21^, an induced subnetwork analysis (Reyna and Raphael, in preparation), NBDI^22^, and SSA-ME^23^), which utilize multiple protein–protein interaction networks, and to two pathway analysis algorithms (ActivePathways^19^ and a hypergeometric analysis (Vazquez)), which utilize multiple pathway/gene-set databases. We defined a non-coding value-added (NCVA) procedure to determine genes whose non-coding scores contribute significantly to the results of the combined coding and non-coding analysis, where NCVA results for a method augment its results on non-coding data. We defined a consensus procedure to combine significant pathways and networks identified by these seven algorithms. The 87 pathway-implicated driver genes with coding variants (PID-C) are the set of genes reported by a majority (≥4/7) of methods on the coding data. The 93 pathway-implicated driver genes with non-coding variants (PID-N) are the set of genes reported by a majority of methods on non-coding data or in their NCVA results. Only five genes (*CTNNB1*, *DDX3X*, *SF3B1*, *TGFBR2*, and *TP53*) are both PID-C and PID-N genes.

We performed a comprehensive pathway and network analysis of cancer drivers using the single-element driver *p*-values computed by the PCAWG Drivers and Functional Interpretation Working Group^16^ as input. We applied seven distinct pathways and network methods (ActivePathways^19^, CanIsoNet^20^, Hierarchical HotNet^21^, a hypergeometric analysis (Vazquez), an induced subnetwork analysis (Reyna and Raphael, in preparation), NBDI^22^, and SSA-ME^23^) that each leverage information from molecular pathways or protein interaction networks (Fig. 1, Methods section) to amplify weak signals in the single-element analysis. All methods were calibrated on randomized data (Pathway and network methods in Supplementary file).

Since the prioritization of non-coding somatic mutations in cancer is not yet a solved problem, it was difficult to know in advance which analysis methodologies, if any, would be best suited to distinguish drivers from passengers by aggregating weak signals across pathways or networks. Thus, we formed a consensus of multiple methods, following the “wisdom of crowds” ensemble approach of machine learning^24^ to improve the specificity of the results. We included methods that used different sources of pathway or network information and different prioritization criteria (see Supplementary Data 1 for a complete list). Each method nominated genes, and consensus sets of genes with possible coding and non-coding driver mutations were defined as the genes found by at least four of the seven methods (Supplementary Data 2–5). We use the term *pathway-implicated driver (PID) genes* to describe these candidate driver genes.

One potential concern with a consensus procedure is that the results may be dominated by a few highly correlated methods. Our pathway or network analysis methods use varied sources of prior knowledge (i.e., pathway databases or interaction networks), and input data (e.g., driver *p*-values, point mutations, and/or gene expression), and rely on different techniques to integrate these data sources. We found only a modest overlap between the output of the seven methods (Method results comparison and Consensus procedure in Supplementary file; Supplementary Data 6–8), suggesting a non-uniform weighting of the consensus to mitigate the influence of redundant methods was not necessary.

**Fig. 2:**
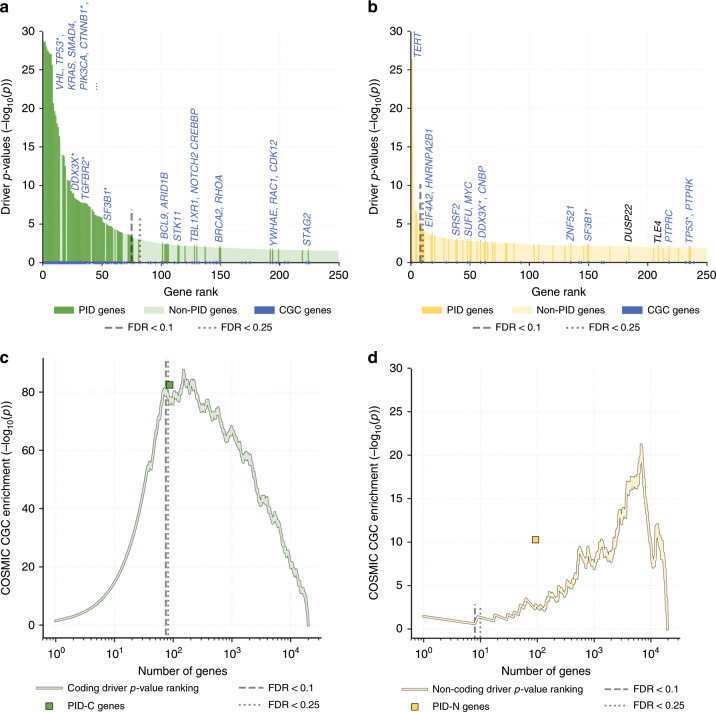
Pathway and driver analysis identifies driver genes in the long tail of the driver *p*-values for coding and non-coding mutations. **a** Pathway and network methods identify significant coding driver mutations. Driver *p*-values on protein-coding elements for 250 genes with most significant coding driver *p*-values; dashed and dotted lines indicate FDR = 0.1 and 0.25, respectively. Dark green bars are PID-C genes, and light green bars are non-PID-C genes. Blue squares below the *x*-axis indicate COSMIC Cancer Gene (CGC) Census genes. In total, 31 of 87 PID-C genes have coding driver *p*-values with FDR > 0.1. Several PID-C genes are labeled, including all CGC genes with coding FDR > 0.1. Overlap between PID-C and PID-N genes is indicated with asterisks. Source data are provided as a Source Data file. **b** Pathway and network methods identify rare non-coding driver mutations. Driver *p*-values on non-coding elements (promoter, 5′ UTR, and 3′ UTR of gene) for 250 genes with most significant non-coding driver *p*-values; dashed and dotted lines indicate FDR = 0.1 and 0.25, respectively. Dark yellow bars are PID-N genes, and light yellow bars are non-PID-N genes. Blue squares are as above. In total, 3 (*TERT*, *HES1*, and *TOB1*) of 93 PID-N genes have non-coding driver *p*-values with FDR ≤ 0.1, while 90 have FDR > 0.1 . Several PID-N genes are labeled, including PID-N genes with significant in cis gene expression changes (see Fig. 3) and all PID-N genes with non-coding FDR > 0.25. Overlap between PID-C and PID-N genes is indicated with asterisks. Source data are provided as a Source Data file. **c** Statistical significance of overlap between top-ranked genes according to coding driver *p*-values and PID-C genes with CGC genes. Fisher’s exact test *p*-values and driver FDR thresholds of 0.1 and 0.25 are highlighted. Green squares indicate overlap between PID-C genes and CGC genes. Source data are provided as a Source Data file. **d** Statistical significance of overlap of genes ranked by driver *p*-values on non-coding (promoter, 5′ UTR, 3′ UTR) elements and CGC genes. Driver FDR thresholds of 0.1 and 0.25 are highlighted. Yellow square indicates overlap between PID-N genes and CGC genes. Source data are provided as a Source Data file.

Using coding mutations alone, we identify a set of 87 pathway-implicated driver genes with coding variants (PID-C genes). The 87 PID-C genes (Supplementary Data 2, Supplementary Fig. 6a) include 68 previously identified cancer genes as catalogued by the COSMIC Cancer Gene Census (CGC) database (v83, 699 genes from Tier 1 and Tier 2)^25^ (2.98 genes expected; Fisher′s exact test *p* = 3.57 × 10^−83^; Fig. 2a, c; Supplementary Fig. 7a). The PID-C genes have significantly higher coding gene scores than non-PID-C genes (rank-sum test *p* = 1.72 × 10^−58^; median rank 48 of PID-C genes), and each of the 87 PID-C genes improves the score of its network neighborhood (19.7 genes expected; *p* < 10^−6^; Supplementary Data 9). This network neighborhood analysis shows that PID-C genes are not implicated solely by their network neighbors^14^, but themselves contribute significantly to their discovery by the pathway and network methods. The 87 PID-C genes also include 31 genes that are not statistically significant (FDR > 0.1) in the PCAWG Drivers and Functional Interpretation Working Group analysis; Fig. 2a, c; Supplementary Figs. 8a, 9), illustrating that the network neighborhoods can nominate genes with infrequent mutations, i.e., those in the “long tail”, as possible driver genes. Interestingly, 13 of these 31 genes with FDR > 0.1 are also known drivers according to the CGC database (3.0 genes expected; Fisher’s exact test *p* = 2.1 × 10^−14^). Thus, the consensus pathway and network analysis recovers many known protein-coding driver mutations and identifies additional possible drivers that are infrequently mutated and thus remain below the statistical significance threshold of gene-specific driver analyses.

Using non-coding mutations alone, we identify a set of 62 genes using our consensus pathway and network analysis, resulting in fewer genes than our analysis with coding mutations. However, when we performed a joint analysis of coding and non-coding mutations, we found that the much stronger signal in coding mutations dominated the combined signal in coding and non-coding mutations. To increase sensitivity to detect contributions of non-coding mutations, we devised a “non-coding value-added” (NCVA) procedure (Fig. 1; Supplementary Fig. 3; Non-coding value-added (NCVA) procedure in the Methods section). Our NCVA procedure asks if the coding mutations enhance the discovery of potential non-coding driver genes beyond what is found with only the non-coding mutations. This procedure identified an additional set of 31 genes that, when merged with the 62 genes found with non-coding mutations alone, resulted in a set of 93 pathway-implicated driver genes with non-coding variants (PID-N) (Supplementary Fig. 4, Consensus results in the Methods section). PID-N genes appear as a robust and biologically relevant set, unbiased by any particular mutational process reflecting a particular carcinogen or DNA damage processes (Supplementary Fig. 5, Mutational signatures in the Methods section).

The 93 PID-N genes (Supplementary Data 3, Supplementary Fig. 6b) include 19 previously identified cancer genes according to the COSMIC Cancer Gene Census (CGC) database a significant enrichment over the 3.2 genes expected (*p* = 5.3 × 10^−11^; Fisher’s exact test, Fig. 2b, d; Supplementary Figs. 7b, c). Excluding the eight genes with individually significant non-coding elements in the PCAWG Drivers and Functional Interpretation Working Group analysis^16^, 19 genes are both PID-N genes and CGC genes, a significant enrichment over the 3.1 genes expected (*p* = 5.3 × 10^−11^; Fisher’s exact test), suggesting that non-coding mutations may alter genes with recurrent coding or structural variants in some samples. The PID-N genes have significantly higher non-coding gene scores than non-PID-N genes (rank-sum test *p* = 1.47 × 10^−58^; median rank 165 of PID-N genes), and 92/93 PID-N genes (except for *HIST1H2BO*) improve the scores of their network neighborhoods (28.5 genes expected; *p* < 10^−6^; Supplementary Data 10). This shows that PID-N genes are not implicated solely by their network neighbors^14^. The vast majority of PID-N genes (90/93, including the 19 CGC genes) are distinct from the PCAWG Drivers and Functional Interpretation Working Group analysis (Fig. 2b; Supplementary Figs. 8b, 9), with only three genes in common: *TERT*, *HES1*, and *TOB1*. Of these three, only *TERT* is recorded as a known cancer gene in the CGC database. Moreover, the 93 PID-N genes are more strongly enriched (*p* = 5.3 × 10^−11^; Fisher’s exact test) for COSMIC CGC genes than the 93 genes with the smallest non-coding driver *p*-values of promoters, 5′ UTRs, or 3′ UTRs (*p* = 4.8 × 10^−3^; Fisher’s exact test). Thus, our consensus procedure of the pathway and network analyses appreciably augments the significantly mutated elements in the PCAWG Drivers and Functional Interpretation Working Group results^16^.

Taken together, the PID-C and PID-N genes contain an additional 121 genes over what was found in the PCAWG Drivers and Functional Interpretation Working Group analysis, including 90 new possible non-coding drivers (Consensus Results in the Methods section). In total, non-coding mutations in PID-N genes cover an additional 151 samples (9.1% of samples) than PID-C genes. We found that most coding mutations in PID-C genes and most non-coding mutations in PID-N genes are clonal (median > 80% for both PID-C and PID-N genes^26^). In addition, the overwhelming majority of the PID-N genes were distinct from PID-C genes (Supplementary Fig. 4) with only five genes in common: *CTNNB1*, *DDX3X*, *SF3B1*, *TGFBR2*, and *TP53*. While this suggests that coding and non-coding driver mutations occur in largely distinct sets of cancer genes, we show below that both types of mutations affect genes underlying many of the same hallmark cancer processes.

### Impact of non-coding mutations on gene expression

**Fig. 3:**
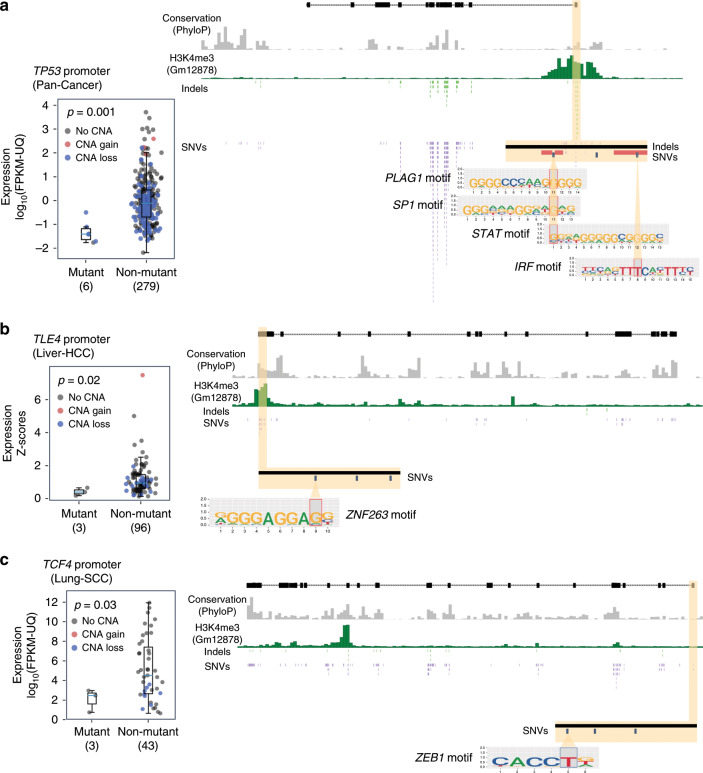
Gene expression changes are correlated with mutations in PID-N genes. Evolutionary conservation of genomic elements estimated with PhyloP are shown as gray features. H3 histone lysine 4 tri-methylation sites (H3K4me3) measured in GM12878 HapMap B-lymphocytes cell lines are highlighted in the green track, indicating active promoter regions near transcription start sites^49^. Boxplot center lines show the median, boxplot bounds show the first quartile Q1 and the third quartile Q3, and whiskers show 1.5 (Q3–Q1) below and above Q1 and Q3, respectively. **a** TP53 promoter. *TP53* coding and non-coding genomic loci with zoomed-in view of the *TP53* promoter region. *TP53* promoter mutations (six mutations in Biliary-AdenoCA, ColoRect-AdenoCA, Kidney-ChRCC, Lung-SCC, Ovary-AdenoCA, and Panc-AdenoCA cancer types) correlate significantly (Wilcoxon rank-sum test *p* = 0.001, FDR = 0.087) with reduced *TP53* gene expression, where FPKM-UQ is upper quartile normalized fragments per kilobase million. Samples with copy-number gains and losses in the *TP53* promoter region are annotated in red and blue, respectively. Two of the six *TP53* promoter mutations overlap with transcription factor-binding sites (with one mutation matching three motifs). Source data are provided as a Source Data file. **b** *TLE4* promoter. *TLE4* coding and non-coding genomic loci with zoomed-in view of *TLE4* promoter region. *TLE4* promoter mutations in Liver-HCC samples (three mutations) correlate (Wilcoxon rank-sum test *p* = 0.02, FDR = 0.2) with lower *TLE4* gene expression. Samples with copy-number gains and losses annotated in red and blue, respectively. One of the three *TLE4* promoter mutations has a transcription factor-binding site for *ZNF263*. Source data are provided as a Source Data file. **c** *TCF4* promoter. *TCF4* coding and non-coding genomic loci with zoomed-in view of *TCF4* promoter region. *TCF4* promoter mutations in Lung-SCC samples (three mutations) correlate (Wilcoxon rank-sum test *p* = 0.03, FDR = 0.27) with lower *TCF4* gene expression. Samples with copy-number gains and losses annotated in red and blue, respectively. One of the three *TCF4* promoter mutations has a transcription factor-binding site for *ZEB1*. Source data are provided as a Source Data file.

Non-coding mutations may act by altering transcription factor-binding sites or other types of regulatory sites. Thus, we evaluated whether non-coding mutations in PID-N genes were associated with in cis expression changes in the same gene. We found that five PID-N genes (FDR < 0.3) showed significant in cis expression correlations out of the 90 that could be tested using RNA-Seq data (Fig. 3; Supplementary Fig. 10; Supplementary Data 11–16). In contrast, 34 out of 87 PID-C genes exhibited significant or near-significant in cis expression correlations (FDR < 0.3) (Supplementary Data 17, 18).

Unsurprisingly, the most significant in cis expression correlation for a PID-N gene is the correlation between *TERT* promoter mutations and increased expression, which we find in 11 Thy-AdenoCA tumors (*p* = 1.3 × 10^−10^, FDR = 3.2 × 10^−9^; Wilcoxon rank-sum test), 11 CNS-Oligo tumors (*p* = 6.8 × 10^−3^, FDR = 9.7 × 10^−2^; Wilcoxon rank-sum test), and 22 CNS-GBM tumors (Wilcoxon rank-sum test *p* = 2.3 × 10^−2^, FDR = 0.19; Wilcoxon rank-sum test) (Supplementary Fig. 8), consistent with previous reports^5,6,27^. Note that these associations were limited by the unavailability of RNA expression data for some samples with *TERT* mutations as well as the low-sequencing coverage in promoter regions that limited the detection of *TERT* promoter mutations. The PCAWG Drivers and Functional Interpretation Working Group investigated the latter issue for two mutation hotspot sites in the *TERT* promoter, and estimated that 216 mutations in these sites were likely not called^16^, a large underrepresentation relative to the total of 97 samples with *TERT* promoter mutations (71 samples with expression data) in our analyses.

We found significant in cis expression correlations in four other PID-N genes: *TP53*, *TLE4*, *TCF4*, and *DUSP22* (Fig. 3, Supplementary Fig. 10). *TP53* shows significantly reduced expression (*p* = 1.0 × 10^−3^; FDR = 8.7 × 10^−2^; Wilcoxon rank-sum test) across six tumors with *TP53* promoter mutations from six different tumor types (Fig. 3a; Supplementary Fig. 10). The reduced expression of mutated samples is consistent with *TP53*’s well-known role as a tumor suppressor gene, and links between *TP53* promoter methylation and expression have been previously investigated^28^. This expression change was also described in the study by the PCAWG Drivers and Functional Interpretation Working Group^16^. *TLE4* shows significantly reduced expression (*p* = 1.7 × 10^−2^; FDR = 0.20; Wilcoxon rank-sum test) in three Liver-HCC tumors with *TLE4* promoter mutations (Fig. 3b; Supplementary Fig. 10). *TLE4* is a transcriptional co-repressor that binds to several transcription factors^29^, and *TLE4* functions as a tumor suppressor gene in acute myeloid lymphoma through its interactions with Wnt signaling^30^. Furthermore, in an acute myeloid lymphoma cell line, *TLE4* knockdown increased cell division rates, while forced *TLE4* expression induced apoptosis^31^. However, the role of *TLE4* in solid tumors is not well understood. *TCF4* shows significantly reduced expression (*p* = 3.4 × 10^−2^; FDR = 0.27; Wilcoxon rank-sum test) in three Lung-SCC tumors with *TCF4* promoter mutations (Fig. 3c; Supplementary Fig. 10). *TCF4* is part of the TCF4/β-catenin complex and encodes a transcription factor that is downstream of the Wnt signaling pathway. Low *TCF4* expression has been observed in Lung-SCC tumors^32^. Finally, *DUSP22* has significantly reduced expressed (*p* = 6.3 × 10^−3^; FDR = 0.024; Wilcoxon rank-sum test) in five Lung-AdenoCA patients with *DUSP22* 3′ UTR mutations and significantly over-expressed (*p* = 7.8 × 10^−4^; FDR = 0.075; Wilcoxon rank-sum test) in three Lung-AdenoCA patients with *DUSP22* 5′ UTR mutations. These UTR mutations were mutually exclusive. *DUSP22* encodes a phosphatase signaling protein, and was recently proposed to be a tumor suppressor in lymphoma^33^.

While these gene expression correlations provide additional support for a subset of PID-N genes, the variant allele frequency of a mutation and the copy number of the gene are additional covariates for gene expression. We found that these covariates did not play a role in of the correlations that we identified: the majority of mutations in each PID gene were clonal (Supplementary Fig. 11) and copy-number changes did not affect the expression correlations for the five PID-N genes described above (Fig. 3; Supplementary Fig. 10). In addition, the low number of PID-N genes exhibiting associated gene expression changes is explained by the low number of samples with mutations in PID-N genes, the uneven availability of expression data across the tumor types, and decreased sequence coverage in promoter regions^16^. These issues further reduced the number of samples with non-coding mutations and RNA expression, limiting the power of in cis gene expression correlation analysis.

### Modular organization of coding and non-coding mutations

**Fig. 4:**
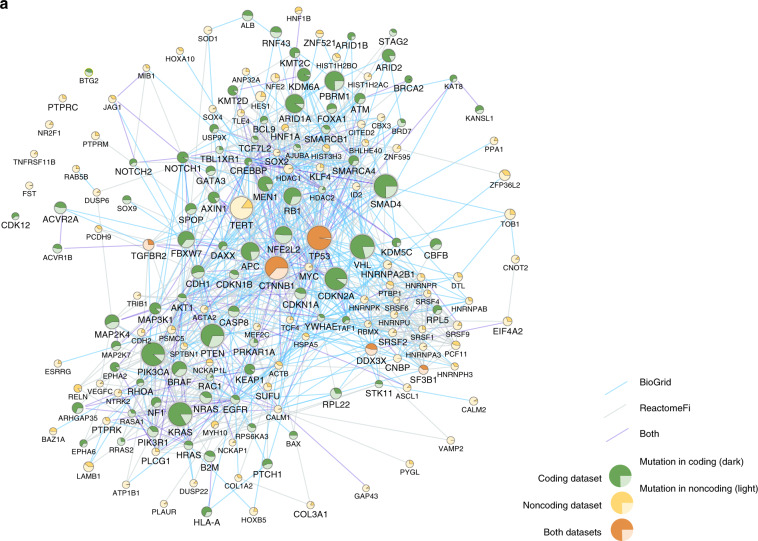

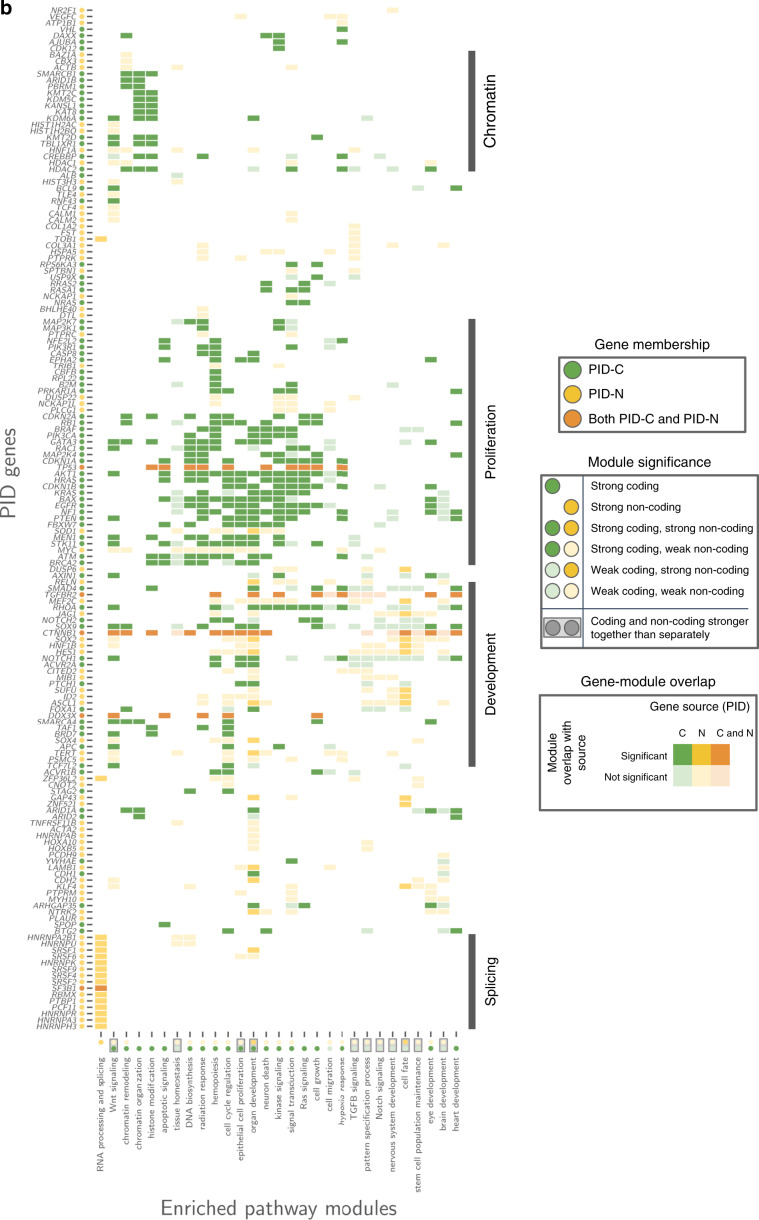
Pathway and network modules containing PID-C and PID-N genes. **a** Network of functional interactions between PID-C and PID-N genes. Nodes represent PID-C and PID-N genes and edges show functional interactions from the ReactomeFI network (gray), physical protein–protein interactions from the BioGRID network (blue), or interactions recorded in both networks (purple). Node color indicates PID-C genes (green), PID-N genes (yellow), or both PID-C and PID-N genes (orange); node size is proportional to the score of the gene; and the pie chart diagram in each node represents the relative proportions of coding and non-coding mutations associated with the corresponding gene. Dotted outlines indicate clusters of genes with roles in chromatin organization and cell proliferation, which predominantly contain PID-C genes; development, which includes comparable amounts of PID-C and PID-N genes; and RNA splicing, which contains PID-N genes. A core cluster of genes with many known drivers is also indicated. **b** Pathway modules containing PID-C and PID-N genes. Each row in the matrix corresponds to a PID-C or PID-N gene, and each column in the matrix corresponds to a pathway module enriched in PID-C and/or PID-N genes (see the Methods section). A filled entry indicates a gene (row) that belongs to one or more pathways (column) colored according to gene membership in PID-C genes (green), PID-N genes (yellow), or both PID-C and PID-N genes (orange). A dark colored entry indicates that a PID-C or PID-N gene belongs to a pathway that is significantly enriched for PID-C or PID-N genes, respectively. A lightly colored entry indicates that a PID-C or PID-N gene belongs to a pathway that is significantly enriched for the union of PID-C and PID-N genes, but not for PID-C or PID-N genes separately. Enrichments are summarized by circles adjacent each pathway module name and PID gene name. Boxed circles indicate that a pathway module contains a pathway that is significantly more enriched for the union of the PID-C and PID-N genes than the PID-C and PID-N results separately. The enriched modules and PID genes are clustered into four biological processes: chromatin, development, proliferation, and RNA splicing as indicated.

We identified specific protein–protein interaction subnetworks and biological pathways that were altered by coding mutations, non-coding mutations, or a combination of both types of mutations. We found significantly more interactions between PID-C genes that expected by chance using a node-degree preserving permutation test (64 interactions observed vs. 40 interactions expected, *p* < 10^−6^), a near-significant number of interactions between PID-N genes (18 vs. 12 expected, *p* = 6.8 × 10^−2^), and significantly more interactions between both PID-C and PID-N genes (67 vs. 40 expected, *p* = 6 × 10^−4^), demonstrating an interplay between coding and non-coding mutations on physical protein–protein interaction networks (Network annotation in the Methods section). We organized the interacting subnetworks involving PID-C and PID-N genes into five biological processes: core drivers, chromatin organization, cell proliferation, development, and RNA splicing (Fig. 4a). While the high frequency of molecular interactions between PID-C and PID-N genes is expected since such interactions were used as a signal in pathway and network methods, the organization of these interactions illustrates the relative contributions of coding and non-coding mutations in individual subnetworks.

We further characterized the molecular pathways enriched among our PID-C and PID-N using the g:Profiler web server^34^ (Fig. 4b; Supplementary Fig. 12, Supplementary Data 19–24, Pathway annotation in the Methods section). Overall, 63 pathways were enriched for PID-C genes and 13 pathways were enriched for PID-N genes (FDR < 10^−6^). Since our gene-prioritization methods use pathway databases and interaction networks as prior knowledge, it is not surprising that PID-C and PID-N genes are enriched in multiple molecular pathways. However, the enrichment results provide clues about the modular organization of the pathways and allow us to assess the relative contributions of coding and non-coding mutations in each pathway.

We further grouped these molecular pathways into 29 modules using overlaps between annotated pathways recorded in the pathway enrichment map (Supplementary Fig. 12). For each enriched module, we examined whether PID-C, PID-N, or both types of genes were responsible for the observed enrichment. This produced a clustering of modules and PID genes into four biological processes: chromatin organization, cell proliferation, development, and RNA splicing (Fig. 4b).

We found that pathways in the chromatin and cell proliferation processes—including chromatin remodeling and organization, histone modification, apoptotic signaling, signal transduction, Ras signaling, and cell growth—were altered primarily by coding mutations in PID-C genes. This is not surprising as these pathways contain many well-known cancer genes, such as *TP53*, *KRAS*, *BRAF*, cyclin-dependent kinase inhibitors, *EGFR*, *PTEN*, and *RB1*.

At the same time, we found that multiple signaling pathways include significant numbers of both PID-C and PID-N genes, suggesting that non-coding mutations provide an alternative to coding mutations in disrupting these pathways. In particular, the Wnt signaling pathway (FDR = 6.8 × 10^−13^), which was predominantly targeted by coding mutations, was also targeted by non-coding mutations in several PID-N genes, including *TERT* (103 mutations), *HNF1A/B* (24 mutations), *TLE4* (32 mutations), *TCF4* (93 mutations), and *CTNNB1* (17 mutations) (Supplementary Fig. 13a). The Notch signaling pathway (FDR = 6.8 × 10^−7^) was associated with comparable numbers of PID-C and PID-N genes, including the PID-N genes *JAG1* and *MIB1* that encode ligands and the PID-N transcription factors *ACL1*, *HES1*, and *HNF1B* (66 non-coding mutations in total) (Supplementary Fig. 13b). The TGF-β signaling pathway (FDR = 3.2 × 10^−7^) also contained both PID-C and PID-N genes, including the PID-N genes *HES1*, *HNF1A/B*, *HSPA5*, *MEF2C*, and the genes *TGFBR2* and *CTNNB1*, which are both PID-C and PID-N genes (214 coding mutations and 166 non-coding mutations).

We found that several developmental processes were altered by significant numbers of both PID-C and PID-N genes. Cell fate determination (FDR = 2.0 × 10^−7^) was predominantly affected by non-coding mutations in the PID-N genes *DUSP6*, *MEF2C*, *JAG1*, *SOX2*, *HES1*, *ACL1*, *ID2*, *SUFU,* and *KLF4* (total 191 non-coding mutations), but also by coding mutations in PID-C genes *BRAF*, *GATA3*, and *NOTCH1/2*. Pathways related to nervous system development (FDR = 5.8 × 10^−8^) were enriched for the PID-N genes *ASCL1*, *CTNNB1*, *ID2*, *SUFU*, and *TERT* that have known roles in cancer^35,36^, complementing the PID-C genes *NOTCH1*, *PTEN*, and *RHOA* that also have known cancer roles. The pattern specification process (FDR = 8.8 × 10^−8^) was also affected by both coding and non-coding mutations, including the PID-N genes *ASCL1*, *SUFU*, and *RELN* and the PID-C genes *ATM* and *SMAD4*. In these cases, non-coding mutations complement the coding mutations that disrupt these pathways, covering additional patients.

Intriguingly, we find that RNA splicing pathways were affected primarily by non-coding mutations (FDR = 7.6 × 10^−9^). A total of 17 PID-N genes involved in splicing-related pathways (Supplementary Fig. 13c), including several heterogeneous nuclear ribonucleoproteins (hnNRP) and serine- and arginine-rich splicing factors (SRSFs). None of these PID-N genes were significantly mutated according to single-element tests used in the PCAWG Drivers and Functional Interpretation Working Group analysis^16^.

**Fig. 5:**
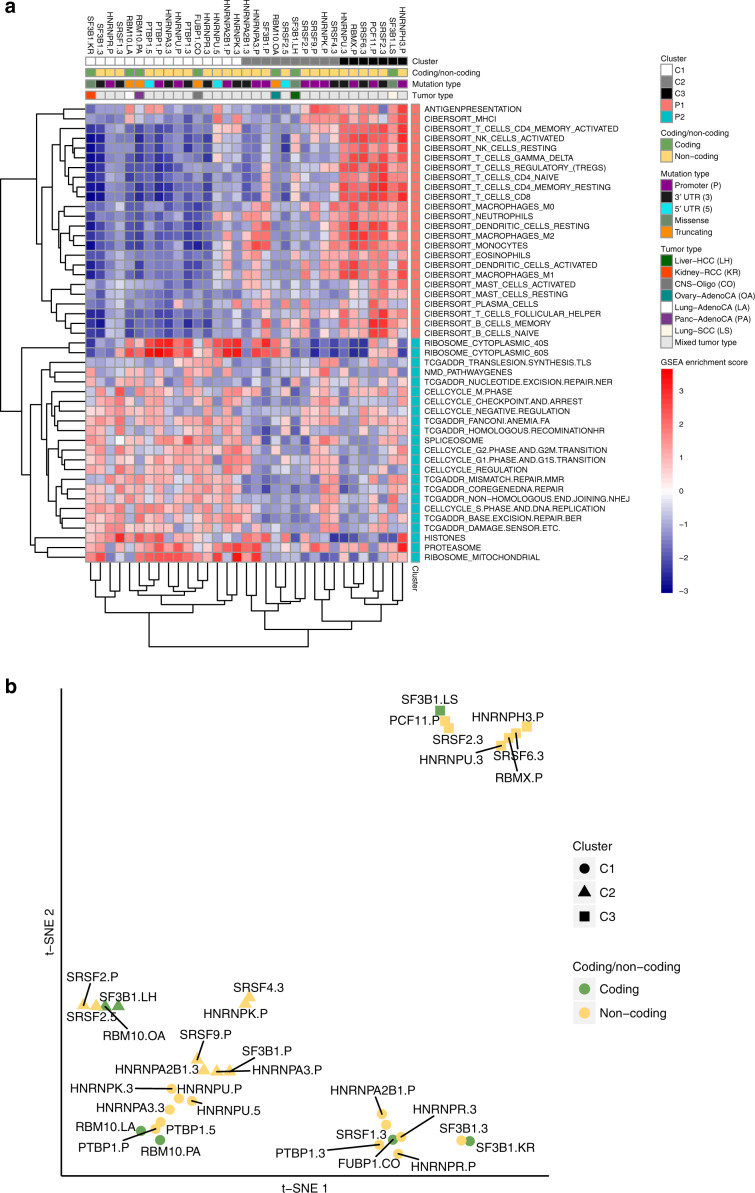
RNA splicing factors are targeted primarily by non-coding mutations and alter expression of similar pathways as coding mutations in splicing factors. **a** Heatmap of Gene Set Enrichment Analysis (GSEA) Normalized Enrichment Scores (NES). The columns of the matrix indicate non-coding mutations in splicing-related PID-N genes and coding mutations in splicing genes reported in ref. ^37^, and the rows of the matrix indicate 47 curated gene sets^37^. Red heatmap entries represent an upregulation of the pathway in the mutated samples with respect to the non-mutated samples, and blue heatmap entries represent a downregulation. The first column annotation indicates mutation cluster membership according to common pathway regulation. The second column annotation indicates whether a mutation is a non-coding mutation in a PID-N gene or a coding mutation, with the third column annotation specifies the aberration type (promoter, 5′ UTR, 3′ UTR, missense, or truncating). The fourth column annotation indicates the cancer type for coding mutations. The mutations cluster into three groups: C1, C2, and C3. The pathways cluster into two groups: P1 and P2, where P1 contains an immune signature gene sets and P2 contains cell-autonomous gene sets. **b** t-SNE plot of mutated elements. Gene expression signatures for samples with non-coding mutations clusters in splicing-related PID-N genes with gene expression signatures for coding mutations in previously published splicing factors. The shape of each point denotes the mutation cluster assignment (C1, C2, or C3), and the color represents whether the corresponding gene is a PID-N gene with non-coding mutations or a splicing factor gene with coding mutations.

As we did not find any significant (FDR < 0.1) in cis associations between non-coding mutations and altered expression in splicing-related PID-N genes, we explored potential in trans effects between non-coding mutations in these genes and expression of other genes. We found that non-coding mutations in splicing-related PID-N genes largely recapitulate a recently published association from a TCGA PanCanAtlas analysis^37^ between coding mutations in several splicing factors and differential expression of 47 pathways (Fig. 5). In particular, we identified three clusters of mutations in RNA splicing genes (C1, C2, and C3; Fig. 5a, b) using hierarchical clustering of differential expression patterns across these pathways. A highly overlapping set of clusters was found using *t*-distributed stochastic neighbor embedding (top annotation bar in Fig. 5a) showing that the clusters were robust to the choice of the clustering method. Further support for robustness of clusters was found via silhouette scores and bootstrapping (Supplementary Fig. 14). Each of these clusters contained at least one coding mutation in the splicing genes *SF3B1*, *FUBP1*, and *RBM10*, as reported previously^37^, along with non-coding mutations in splicing-related PID-N genes, demonstrating that both types of mutations resulted in similar gene expression signatures. The joint analysis of coding and non-coding mutations in splicing factors also recovered the two groups of enriched pathways^37^ (P1 and P2 in Fig. 5a; Supplementary Fig. 15). One group (P1) is characterized by immune cell signatures and the other group (P2) reflects mostly cell-autonomous gene signatures of cell cycle, DDR, and essential cellular machineries^37^. This similarity between the gene expression signatures for non-coding mutations in several PID-N splicing factors and the signatures previously reported for coding mutations in splicing factor genes^37^ supports a functional role for splicing-related PID-N genes in altering similar gene expression programs.

In addition to the above modules, we also found that transcription factors were well represented among both the PID-C and PID-N genes. In total, nine PID-C genes are transcription factors (*ARHGAP35*, *ARID2*, *FOXA1*, *GATA3*, *NFE2L2*, *SMAD4*, *SOX9*, *TCF7L2*, *TP53*; FDR = 2.1 × 10^−10^), while 19 PID-N genes are transcription factors (*ASCL1*, *BHLHE40*, *ESRRG*, *HES1*, *HNF1A*, *HNF1B*, *HOXA10*, *HOXB5*, *KLF4*, *MEF2C*, *MYC*, *NFE2*, *NR2F1*, *SOX2*, *SOX4*, *TCF4*, *TP53*, *ZNF521*, *ZNF595*; FDR = 4.1 × 10^−20^). This observation suggests that non-coding mutations may affect transcriptional regulatory networks.

## Discussion

We present an integrative pathway and network analysis that expands the list of genes with possible non-coding driver mutations into the “long tail” of rarely mutated elements that are not significant by single-element analysis. We find that genes harboring both coding or non-coding mutations overlap in pathways and networks; thus, pathway databases and interaction networks serve as useful sources of prior knowledge to implicate additional mutated elements beyond those identified by single-element tests. In total, our integrative pathway and network analysis identified 87 pathway-implicated driver genes with coding variants (PID-C) and 93 pathway-implicated driver genes with non-coding variants (PID-N). Importantly, 90 PID-N genes were not statistically significant (FDR > 0.1) by single-element tests on non-coding mutation data, and these genes are key candidates for future experimental characterization. Among them, we find that promoter mutations in *TP53*, *TLE4*, and *TCF4* are associated with reduced expression of these genes.

We find that coding and non-coding driver mutations largely target different genes and make varying contributions to pathways and networks perturbed in cancer. While some cancer pathways are targeted by both coding and non-coding mutations, such as the Wnt and Notch signaling pathways, other pathways appear to be predominantly altered by one class of mutations. In particular, we find non-coding mutations in multiple genes in the RNA splicing pathway, and samples with these mutations exhibit gene expression signatures that are concordant with gene expression changes observed in samples with coding mutations splicing factors *SF3B1*, *FUBP1*, and *RBM10*^37^. Together these results demonstrate that rare non-coding mutations may result in similar perturbations to both common and complementary biological processes.

There are several caveats to the results reported in this study. First, there is relatively low power to detect non-coding mutations in the cohort, particularly in cancer types with small numbers of patients. Second, transcriptomic data were available for only a subset of samples, further reducing our ability to validate our predictions using gene expression data. Third, our pathway and network analysis relied on the driver *p*-values from the PCAWG Drivers and Functional Interpretation Working Group analysis^16^. While this analysis accounts for regional variations in the background mutation rate across the genome, it is possible that these corrections are incomplete. Furthermore, if the uncorrected confounding variables are correlated with gene membership in pathways and subnetworks, then the false positive rates in our analysis may be higher than estimated. All of these factors, plus other unknown confounding variables, make it difficult to assess the false discovery rate of our predictions, particularly for PID-N genes. Further experimental validation of these predictions is necessary to determine the true positives from false positives in our PID gene lists.

Because of limited power in individual cancer types, our pathway and network analysis focused on associations found across cancer and tissue types. Thus, we primarily utilized generic, tissue-independent network and pathway information. However, it is well known that gene–gene interactions vary across tissues and that cancer cells rewire signaling and regulatory networks. Future investigations that consider the variable landscape of regulatory and physical interactions across tissues may offer a new perspective on the data. Specifically, each cell type has a different epigenetic wiring and regulatory machinery, and non-coding mutations may target cell type-dependent vulnerabilities. Approaches that incorporate tissue-specific, cancer-specific, or patient-specific gene–gene regulatory information may reveal new classes of drivers unexplored with our current approaches.

Our pathway- and network-driven strategies enable us to interpret the coding and non-coding landscape of tumor genomes to discover driver mechanisms in interconnected systems of genes. This approach has multiple benefits. First, by broadening our mutation analysis from single genomic elements to pathways and networks of multiple genes, we identify new components of known cancer pathways that are recurrently altered by both coding and non-coding mutations, and thus likely to be important in cancer. Second, we identify new pathways and subnetworks that would remain unseen in an analysis focusing on coding sequences. Investigation of the coding and non-coding mutations that perturb these pathways and networks will enable more accurate patient-stratification strategies, pathway-focused biomarkers, and therapeutic approaches.

## Methods

### Mutation and pathway data

We used gene scores derived from the PCAWG Drivers and Functional Interpretation Working Group analysis^16^ and combined several pathways and interaction networks for our pathway and network analyses. Here, we use the term “pathway methods” to refer to approaches that utilize sets of related genes for their analysis and use the term “network methods” to refer to approaches that utilize pairwise interactions among genes and/or their products.

### Somatic mutation data

We obtained consensus driver *p*-values (syn8494939) from the PCAWG Drivers and Functional Interpretation Working Group analysis^16^ for coding and non-coding (core promoter, 5′ UTR, 3′ UTR, enhancers) genomic elements for the Pancan-no-skin–melanoma–lymph cohort. We removed driver *p*-values for several elements (*H3F3A* and *HIST1H4D* coding; *LEPROTL1*, *TBC1D12*, and *WDR74* 5′ UTR; and chr6:142705600-142706400 enhancer, which targets *ADGRG6*) that the PCAWG Drivers and Functional Interpretation Working Group analysis had manually examined and discarded. We included enhancers with ≤ 5 gene targets (syn7201027), which covered 89% of enhancers elements from the PCAWG Drivers and Functional Interpretation Working Group analysis^16^. In cases where the PCAWG Drivers and Functional Interpretation Working Group analysis reported multiple *p*-values for the same genomic element, we used the smallest reported *p*-value for that element.

### Derivation of gene scores

Pathway databases and gene interaction networks typically record information at the level of individual genes. Thus, we formed coding and non-coding gene scores by combining PCAWG driver *p*-values across coding and/or non-coding (core promoter, 5′ UTR, 3′ UTR, enhancer) genomic elements as follows. Let *p*_*x*_(*g*) be the driver *p*-value for element *x* of gene *g* from the PCAWG Drivers and Functional Interpretation Working Group analysis^16^. We combined *p*-values from multiple elements using Fisher’s method, where we selected the minimum *p*-value min(*p*_promoter_(*g*), *p*_5’UTR_(*g*)) for overlapping core promoter and 5′ UTR elements on gene *g* and the minimum *p*-value *p*_enhancer_(*g*) of all enhancers targeting gene *g*. Using this approach, we defined the following gene scores on coding (GS-C), non-coding, (GS-N), and combined coding and non-coding (GS-CN) genomic elements:

1. GS-C: *p*_C_(*g*) = *p*_coding_(*g*)
2. GS-N: *p*_N_(*g*) = fisher(min(*p*_promoter_(*g*), *p*_5′UTR_(*g*)), *p*_3′UTR_(*g*), *p*_enhancer_(*g*))
3. GS-CN: *p*_CN_(*g*) = fisher(*p*_coding_(g), min(*p*_promoter_(*g*), *p*_5′UTR_(*g*)), *p*_3′UTR_(*g*), *p*_enhancer_(*g*)).

Here, $p = {{{\mathrm{fisher}}}}(p_1, \ldots ,p_k) = - 2\mathop {\sum}\nolimits_{i = 1}^k {{{{\mathrm{ln}}}}} \left( {p_i} \right)\sim \chi _{2k}^2$, is Fisher’s method for combining *p*-values, where *2k* is the degrees of freedom in the calculation. When the driver *p*-value for a genomic element was undefined, we omitted that element from the calculation and reduced the number of degrees of freedom.

For the pathway and networks methods that analyze individual mutations, we used mutations from the PCAWG MAF (syn7364923) on the same genomic elements as the PCAWG Drivers and Functional Interpretation Working Group analysis, i.e., coding, core promoter, 5′ UTR, 3′ UTR, and enhancer. We removed melanoma and lymphoma samples as well as 69 hypermutated samples with over 30 mutations/MB (syn7894281, syn7814911). We also removed mutations in elements that the PCAWG Drivers and Functional Interpretation Working Group analysis had manually examined and discarded (see above), resulting in lists of mutations used for later assessing biological relevance of our results (syn8103141, syn9684700).

### Pathway and network databases

Pathway methods used gene sets from six databases: CORUM^38^, GO^39^, InterPro^40^, KEGG^41^, NCI Nature^42^, and Reactome^43^ (syn3164548, syn11426307), where small (<3 genes) and large (>1000 genes) pathways were removed.

Network methods used interactions from three interaction networks: the largest connected subnetwork of the ReactomeFI 2015 interaction network^44^ (syn3254781) with high-confidence (≥ 0.75 confidence score) interactions, which we treated as undirected; the largest connected subnetwork of the iRefIndex14 interaction network^45^, which we augmented with interactions from the KEGG pathway database^41^ (syn10903761). The BioGRID interaction network^46^ (syn3164609) was also used to evaluate and annotate results.

### Individual pathway and network algorithms

We applied seven pathway and network methods to the gene scores and mutation data. We used two pathway methods: ActivePathways^19^ and a hypergeometric analysis (Vazquez). We also used five network methods: CanIsoNet^20^, Hierarchical HotNet^21^, an induced subnetwork analysis (Reyna and Raphael, in preparation), NBDI^22^, and SSA-ME^23^. Table 1 shows pathway databases and interaction networks used by each method.

**Table 1:** Summary of pathway database and interaction network data for each method.

Method	Pathway databases or interaction networks
ActivePathways	Gene Ontology (GO)^39^ biological processes, Reactome^43^ pathways
CanIsoNet	STRING v10^50^, DIMA^51^, 3did^52^
Hierarchical HotNet	ReactomeFI 2015^43^, iRefIndex14+KEGG^41, 45^
Hypergeometric analysis	GO biological processes; CORUM^38^, KEGG^41^, InterPro^41^, Nature NCI^42^ pathways
Induced subnetwork analysis	ReactomeFI 2015^43^, iRefIndex14+KEGG^41, 45^
NBDI	ReactomeFI 2015^43^
SSA-ME	ReactomeFI 2015^43^

Using these pathway and network databases, we ran each method on the GS-C, GS-N, and GS-CN gene scores to identify three corresponding lists of genes. Each method evaluated the statistical significance of its results on each data set.

### Non-coding value-added (NCVA) procedure

The GS-CN results leverage both coding and non-coding mutation data, improving the detection of weaker pathway and network signals. We devised a non-coding value-added (NCVA) procedure to separate the coding and non-coding signals in this combined analysis, resulting in a set of NCVA genes for which the non-coding mutation data make a statistically significant contribution to their discovery in the GS-CN results. Specifically, we evaluated the statistical significance of genes in the GS-CN results using a permutation test where the driver *p*-values for coding elements were fixed and the driver *p*-values for non-coding elements were permuted. This procedure identified the subset of the GS-CN results that were reported infrequently (*p* < 0.1) on permuted data, and thus more likely to be true positives. Each method’s NCVA results were added to that method’s overall set of non-coding results (GS-N).

### Consensus results for pathway and network methods

We defined a consensus set of genes for each set of results: GS-C results, GS-N results, GS-CN results, and GS-N combined with NCVA results, across our seven pathway and network methods. Specifically, we defined a gene to be a consensus gene if it was found by a majority (≥ 4/7) of the pathway and network methods. For our analysis, we focused on the consensus GS-C results, which we call the pathway-implicated driver genes with coding variants (PID-C), and the consensus from the GS-N results combined with NCVA results, which we call the pathway-implicated driver genes with non-coding variants (PID-N). We defined PID-C genes as the 87 genes in the consensus of the GS-C results, and we defined PID-N genes as the 93 genes in the consensus of each method’s GS-N results combined with its NCVA results. We performed several analyses to assess the biological relevance of PID-C and PID-N genes.

### Identification of mutational signatures of PID genes

We performed a permutation-based enrichment test for mutation signatures from PCAWG mutation signatures analysis^47^. We identified the most likely mutation signature for each non-coding mutation in PID-N genes and compared them to randomly chosen non-coding mutations in non-PID-N genes.

### Improved network neighborhood scores of PID genes

To assess the extent to which gene scores on PID genes contribute to their detection by pathway and network methods, we considered the contribution of each PID gene’s score to the score of its network neighborhood in the BioGRID interaction network.

For each PID gene *g*, we used Fisher’s method to combine the gene scores of the first-order network neighbors of *g* both with and without the score of *g* itself. In particular, for gene *g*, let *p*(*g*) be the gene score for *g* and *N*(*g*) be the network neighborhood of *g*. Then

$$p_{N(g)}^{{{{\mathrm{with}}}}} = {{{\mathrm{fisher}}}}\left( {p(v):v \in N\left( g \right) \cup \left\{ g \right\}} \right)$$

is a score for the network neighborhood of *g* when including gene *g* and

$$p_{N(g)}^{{{{\mathrm{without}}}}} = {{{\mathrm{fisher}}}}\left( {p(v):v \in N\left( g \right)} \right)$$

is a score for the network neighborhood of *g* when excluding gene *g*.

If the network neighborhood of *g* has a smaller *p*-value with *g* than without *g*, i.e., $p_{N(g)}^{{{{\mathrm{with}}}}} \, < \, p_{N(g)}^{{{{\mathrm{without}}}}}$, then gene *g* improves the score of the network neighborhood, suggesting that the gene score of *g* plays a role in its detection by pathway and network methods. Alternatively, if the network neighborhood of *g* has a larger *p*-value with *g* than without *g*, i.e., $p_{N(g)}^{{{{\mathrm{with}}}}} \, > \, p_{N(g)}^{{{{\mathrm{without}}}}}$, then gene *g* worsens the score of the network neighborhood, suggesting that the gene scores of the network neighbors of *g* are predominantly responsible for the detection of *g* by pathway and network methods.

We performed this test for every PID-C gene with GS-C gene scores and every PID-N gene with GS-N gene scores. We also sampled genes uniformly at random from the network (87 for PID-C genes and 93 for PID-N genes; 10^6^ trials) to ascertain whether significantly more PID genes that improved the scores of their network neighborhoods than expected by chance.

### Expression analysis of PID genes

We evaluated whether mutation status of each PID gene was correlated with RNA expression. We used PCAWG-3 gene expression data (syn5553991), which was averaged from TopHat2 and STAR-based alignments, with FPKM-UQ normalization. Tumor type and copy-number aberrations are known to be covariates for gene expression, so we conditioned on tumor types and annotated copy-number aberrations.

We used the following procedure to assess expression correlations on individual tumor types. We only considered cases with at least three mutated samples and three non-mutated samples to restrict our analysis to cases with sufficient statistical power. For each PID-C gene or each non-coding element in a PID-N gene, we partitioned the samples with expression data into a set *A* of samples with mutation(s) in the element and a set *B* of samples without mutations in the element. We performed the Wilcoxon rank-sum test for the expression of the gene in sets *A* and *B* and performed the Benjamini–Hochberg correction on each coding or non-coding element to provide FDRs.

We used the following procedure to assess expression correlations across tumor types. We only considered cases with at least one mutated sample and one non-mutated sample to restrict our analysis to cases with sufficient statistical power. For each PID-C gene and each non-coding element in a PID-N gene, we partitioned the samples with expression data into sets *A*_*c*_ of samples in cohort *c* with mutation(s) in the element and sets *B*_*c*_ of samples in cohort *c* without mutations in the element. We converted the expression values into *z*-scores using the expression from non-mutated samples in cohort *c*, and we computed the Wilcoxon rank-sum test on the expression of the gene in sets from *A* = ⋃_*c*∊*C*_ *A*_*c*_ and *B* = ⋃_*c*∊*C*_ *B*_*c*_, where *C* is the set of all cohorts containing samples with mutation(s) in the element. We then performed the Benjamini–Hochberg correction on each coding or non-coding element to provide FDRs.

### Network annotation of PID genes

We performed a permutation test to evaluate the statistical significance of the number of interactions in the BioGRID high-confidence interaction network between PID-C genes, the number of interactions between PID-N genes, and the number of interactions between PID-C and PID-N genes, i.e., when a PID-C gene interacts with a PID-N gene. To compute the permutation *p*-value, we sampled random networks uniformly at random from the collection of networks with the same degree sequence as the BioGRID network.

We found connected subnetworks of 46 PID-C genes (31 genes expected, *p* = 9 × 10^−4^) and 16 PID-N genes (10 genes expected, *p* = 6.1 × 10^−2^) in the high-confidence BioGRID^48^ protein–protein interaction (PPI) network. The union of the PID-C and PID-N genes formed a larger connected subnetwork of 73 genes (Fig. 4a). These connected subnetworks were significantly larger than expected by chance according to this permutation test (57 genes expected, *p* = 2.2 × 10^−3^). Furthermore, we observed statistically significant numbers of protein–protein interactions between PID-C and PID-N genes (67 interactions observed vs. 45 expected, *p* = 6 × 10^−4^), suggesting that the associated mutations may target an overlapping set of underlying pathways. The PID-C genes were connected by significantly more interactions than expected (64 vs. 40 expected, *p* < 10^−4^), and the PID-N genes were interconnected at a sub-significant level (18 vs 12 expected, *p* = 6.8 × 10^−2^). Thus, certain pathways are affected by either coding or non-coding mutations, but some pathways are affected by a complement of both coding and non-coding mutations.

### Pathway annotation of PID genes

Using g:Profiler^34^, we performed a pathway enrichment analysis for PID genes and 12,061 gene sets representing GO biological processes and Reactome pathways. We used the Benjamini–Hochberg correction to control the FDR of the results.

### Characterization of PID genes in RNA splicing

GSEA enrichment analysis was performed with the default parameters using the curated pathway gene lists^37^ for samples harboring non-synonymous coding mutations in five genes (*FUBP1*, *RBM10*, *SF3B1*, *SRSF2*, and *U2AF1*) with confirmed on-target splicing deregulation. Due to limited number of samples with RNA-seq data in individual tumor types, we restricted our analysis to missense mutations in *SF3B1*, truncating mutations in *RBM10*, and truncating mutations in *FUBP1* for tumor types contained at least three samples with these classes of mutations. Each tumor type containing such mutations was considered separately^37^.

We performed the same GSEA analysis for non-coding mutations in 17 PID-N genes that were annotated as involved in RNA splicing. Due to limited number of samples from individual tumor types containing mutations in these genes (often there was only one per tumor type), we performed GSEA analysis jointly on all tumor types containing mutations in an individual PID-N gene, restricting the non-mutated group to samples from the same tumor types as the mutant samples. The GSEA Normalized Enrichment Scores (NES) were clustered using hierarchical complete linkage clustering on the Euclidean distance between the NES scores. Separately, we computed a 2D projection of NES scores using t-Distributed Stochastic Neighbor Embedding (t-SNE).

### Ethical review

Sequencing of human subjects' tissue was performed by ICGC and TCGA consortium members under approval of local Institutional Review Boards (IRBs). Informed consent was obtained from all human participants. All data were deidentified for this study, and data access for participating researchers was obtained through data access agreements between local institutions, the ICGC Data Access Compliance Office (DACO), and the NIH dbGaP.

## Supplementary information

Supplementary Information

Description of Additional Supplementary Files

Supplementary Data 1

Supplementary Data 2

Supplementary Data 3

Supplementary Data 4

Supplementary Data 5

Supplementary Data 6

Supplementary Data 7

Supplementary Data 8

Supplementary Data 9

Supplementary Data 10

Supplementary Data 11

Supplementary Data 12

Supplementary Data 13

Supplementary Data 14

Supplementary Data 15

Supplementary Data 16

Supplementary Data 17

Supplementary Data 18

Supplementary Data 19

Supplementary Data 20

Supplementary Data 21

Supplementary Data 22

Supplementary Data 23

Supplementary Data 24

Supplementary Data 25

Supplementary Data 26

## Data Availability

Raw data are available through the PCAWG data portal https://dcc.icgc.org/pcawg. Processed data from the PCAWG Networks and Pathways working groups as well as other PCAWG working groups are available on www.synapse.org at the Synapse links provided in this section. The source data underlying Figs. 2a–c, 3a, b, c, and Supplementary Figs. 1a–c, 7a–c, 10a–h are provided as a Source Data file. Somatic and germline variant calls, mutational signatures, subclonal reconstructions, transcript abundance, splice calls and other core data generated by the ICGC/TCGA Pan-cancer Analysis of Whole Genomes Consortium is described here^15^ and available for download at https://dcc.icgc.org/releases/PCAWG. Additional information on accessing the data, including raw read files, can be found at https://docs.icgc.org/pcawg/data/. In accordance with the data access policies of the ICGC and TCGA projects, most molecular, clinical and specimen data are in an open tier, which does not require access approval. To access potentially identification information, such as germline alleles and underlying sequencing data, researchers will need to apply to the TCGA Data Access Committee (DAC) via dbGaP (https://dbgap.ncbi.nlm.nih.gov/aa/wga.cgi?page=login) for access to the TCGA portion of the data set, and to the ICGC Data Access Compliance Office (DACO; http://icgc.org/daco) for the ICGC portion. In addition, to access somatic single-nucleotide variants derived from TCGA donors, researchers will also need to obtain dbGaP authorization. Derived data sets described specifically in this paper can be found at these locations:

**Table:** 

Label	Synapse ID	ICGC DCC URL	ICGC DCC file name	Access (open/controlled)
PCAWG driver p-values	syn8494939	https://dcc.icgc.org/releases/PCAWG/networks/	final_integration_results_2017_03_16.tar.gz	Open
Enhancer-gene mappings	syn7201027	https://dcc.icgc.org/releases/PCAWG/networks/	map.enhancer.gene.txt.gz	Open
Somatic MAF file	syn7364923	https://dcc.icgc.org/releases/PCAWG/consensus_snv_indel/	final_consensus_passonly.snv_mnv_indel.icgc.public.maf.gz	Open
Somatic MAF file	syn7364923	https://dcc.icgc.org/releases/PCAWG/consensus_snv_indel/	final_consensus_passonly.snv_mnv_indel.tcga.controlled.maf.gz	Controlled
Hypermutated donors	syn7894281	https://dcc.icgc.org/releases/PCAWG/networks/	Hypermutated_spls_removed_ActiveDriver2_AllScores_211216.txt	Open
Hypermutated samples	syn7814911	https://dcc.icgc.org/releases/PCAWG/networks/	Hypermutated_spls_removed_ActiveDriver2_AllScores_291116.aliquotid.txt	Open
Mutations to coding and noncoding elements	syn8103141	https://dcc.icgc.org/releases/PCAWG/networks/	PCAWG_mutations_to_elements.icgc.public.txt.gz	Open
Mutations to coding and noncoding elements	syn8103141	https://dcc.icgc.org/releases/PCAWG/networks/	PCAWG_mutations_to_elements.tcga.controlled.txt.gz	Controlled
Mutation matrix	syn9684700	https://dcc.icgc.org/releases/PCAWG/networks/	PCAWG.gene_status.all.tsv.gz	Controlled
Primary pathway databases	syn3164548	https://dcc.icgc.org/releases/PCAWG/networks/	Gene_sets_pathways_processes_functions.zip	Open
Secondary pathway databases	syn11426307	https://dcc.icgc.org/releases/PCAWG/networks/	PCAWG-5.pathway.data.CNIO.tar.gz	Open
ReactomeFI 2015 network	syn3254781	https://dcc.icgc.org/releases/PCAWG/networks/	Functional_interaction_network_Reactome_FI_Network_2015.zip	Open
iRefIndex14 network	syn10903761	https://dcc.icgc.org/releases/PCAWG/networks/	irefindex14-kegg.tsv.gz	Open
BioGRID network	syn3164609	https://dcc.icgc.org/releases/PCAWG/networks/	Protein_Protein_interaction_network_BIOGRID_filtered.zip	Open
STRING v10 network	syn11712027	https://dcc.icgc.org/releases/PCAWG/networks/	string10_ppi_high_confident_edges.tsv	Open
PCAWG gene expression data	syn5553991	https://dcc.icgc.org/releases/PCAWG/transcriptome/gene_expression/	tophat_star_fpkm_uq.v2_aliquot_gl.tsv.gz	Controlled
PCAWG pathway and network method results	syn21413360	https://dcc.icgc.org/releases/PCAWG/networks/	pathway_and_network_method_results.tar.gz	Open
PCAWG pathway and network consensus results	syn11654843	https://dcc.icgc.org/releases/PCAWG/networks/	method_results_2017_10_10.tar.gz	Open
Coding and noncoding elements	syn21416282	https://dcc.icgc.org/releases/PCAWG/networks/	gene-coding-and-non-coding-elements.tar.gz	Open
Transcript expression data (Counts)	syn7536588	https://dcc.icgc.org/releases/PCAWG/transcriptome/transcript_expression/	pcawg.rnaseq.transcript.expr.counts.tsv.gz	Controlled
Transcript expression data (FPKM)	syn7536589	https://dcc.icgc.org/releases/PCAWG/transcriptome/transcript_expression/	pcawg.rnaseq.transcript.expr.fpkm.tsv.gz	Controlled
eQTL data	syn17096221	https://dcc.icgc.org/releases/PCAWG/transcriptome/eQTL/summarystats/somatic/	all_somatic_eqtl.tsv.tar.gz	Controlled
Gene-level copy-number data	syn8291899	https://dcc.icgc.org/releases/PCAWG/consensus_cnv/gene_level_calls/	all_samples.consensus_CN.by_gene.170214.txt.gz	Open
CanIsoNet PCAWG Ensembl transcripts	syn7536587	https://dcc.icgc.org/releases/PCAWG/transcriptome/transcript_expression/	pcawg.rnaseq.transcript.expr.tpm.tsv.gz	Open
CanIsoNet GTEx Ensembl transcripts	syn7596599	https://dcc.icgc.org/releases/PCAWG/transcriptome/transcript_expression/	GTEX_v4.pcawg.transcripts.tpm.tsv.gz	Open
CanIsoNet filtered PCAWG samples	syn7416381	https://dcc.icgc.org/releases/PCAWG/transcriptome/metadata/	rnaseq.extended.metadata.aliquot_id.V4.tsv	Open
CanIsoNet filtered GTEx samples	syn7596611	https://dcc.icgc.org/releases/PCAWG/transcriptome/metadata/	GTEX_v4.metadata.tsv.gz	Open
CanIsoNet protein–protein isoforms	syn10245952	https://dcc.icgc.org/releases/PCAWG/networks/	isoNet.tsv.gz	Open
CanIsoNet shortest path results	syn9770515	https://dcc.icgc.org/releases/PCAWG/networks/	string_cosmic_neighbourhood_min900_shell3_20160527.tsv.xz	Open
CanIsoNet functional regions	syn7345646	https://dcc.icgc.org/releases/PCAWG/networks/	allCombined.zip	Open
CanIsoNet results (noncoding region)	syn9765614	CanIsoNet results (noncoding region)	non_canIsoNet_mdi_results_noLymNoMel.tsv	Open
CanIsoNet results (coding region)	syn9765615	CanIsoNet results (coding region)	cds_canIsoNet_mdi_results_noLymNoMel.tsv	Open

## Source data

Source Data

## Code availability

Code for the contributing methods in this analysis can be found from their respective papers or by request to the contributing author. The core computational pipelines used by the PCAWG Consortium for alignment, quality control and variant calling are available to the public at https://dockstore.org/search?search=pcawg under the GNU General Public License v3.0, which allows for reuse and distribution.
