# Supplementary material for: Pathway and network analysis of more than 2500 whole cancer genomes
Source: Nat Commun. 2020 Feb 5;11:729. doi: 10.1038/s41467-020-14367-0 (PMC7002574; doi:10.1038/s41467-020-14367-0)
Supplement: Supplementary file 1 — Supplementary Information [file 41467_2020_14367_MOESM1_ESM.pdf]

Supplementary methods

Somatic mutation and gene expression data

This section describes the somatic mutation and gene expression data used in our pathway and network analysis.

Gene-level mutation data

Pathway and network databases record interactions at the gene or protein level. Therefore, we combine somatic mutation data for coding and non-coding elements into gene-level scores using the following procedure. *P*-values from the PCAWG-2-5-9-14 analysis summarize the statistical significance of somatic mutations on these regions. For each gene, we use Fisher's method to combine *P*-values for multiple regions that are associated to the gene to create three gene scores: (1) a coding gene score (GS-C); (2) a non-coding (promoter, 5' UTR, 3' UTR, and enhancer) gene score (GS-N); and (3) a combined coding-and-non-coding (coding, promoter, 5' UTR, 3' UTR, and enhancer) gene score (GS-CN).

Mutation data

We obtained and processed two sources of somatic mutation data on various coding and non-coding regions associated with one or more genes: (1) binary mutation data that describe the presence or absence of mutations in a region for each sample in a tumor cohort and (2) integrated driver score *P*-values that describe the statistical significance of mutations in a region across samples in a cohort.

1. For binary mutation data we used the following procedure:
    a. We obtained somatic mutations from the PCAWG MAF (syn7364923).
    b. We retained mutations in a pan-cancer tumor cohort that excludes samples from the lymphoma and melanoma tumor cohorts, i.e., the Lymph-BNHL, Lymph-CLL, Lymph-NOS, and Skin-Melanoma cohorts, as well as 69 hypermutated samples with over 30 mutations/MB, which are listed by donor (syn7894281) or aliquot ID (syn7814911).
    c. We retained mutations in defined coding and non-coding elements (syn8103141), i.e., coding, core promoter, 5' UTR, 3' UTR, and enhancer

elements. We use core instead of domain regions because driver scores are only defined on core promoter regions. We will refer to core promoter mutations as promoter mutations for the rest of the supplement.

   d. We removed mutations from six elements that the PCAWG driver discovery group removed as part of their analysis. These elements have significant driver scores (FDR < 0.1) that were attributed to technical artifacts or unmodeled mutational processes. The removed elements are the coding regions of *H3F3A* and *HIST1H4D* (coding); the 5’ UTRs of *LEPROTL1*, *TBC1D12*, *WDR74*; and chr6:142705600-142706400, which is an enhancer region that targets *ADGRG6*.

2. Driver score *P*-values:
   a. We obtained integrated driver score *p*-values (syn8494939) for each cohort.
   b. We used the consensus Brown_observed scores (syn8494939) from the Pancan-no-skin-melanoma-lymph cohort on coding, core promoter, 5’ UTR, 3’ UTR, and enhancer elements. We use core instead of domain regions because driver scores are only defined on core promoter regions. We will refer to core promoter mutations as promoter mutations for the rest of the supplement.
   c. We removed mutations from six elements that the PCAWG driver discovery group removed as part of their analysis. These elements have significant driver scores (FDR < 0.1) that were attributed to technical artifacts or unmodeled mutational processes. The removed elements are the coding regions of *H3F3A* and *HIST1H4D* (coding); the 5’ UTRs of *LEPROTL1*, *TBC1D12*, WDR74; and chr6:142705600-142706400, which is an enhancer region that targets *ADGRG6*.

Aggregated mutation data

We combined binary mutation data and driver score *P*-values across multiple coding and/or non-coding regions associated with a gene to generate gene-level data.

We defined coding, non-coding, and combined coding-and-non-coding data on the following elements:

1. Coding elements: coding elements;
2. Non-coding elements: promoter, 5’ UTR, 3’ UTR, and enhancer elements; and
3. Combined coding-and-non-coding elements: coding, promoter, 5’ UTR, 3’ UTR, and enhancer elements.

We combine element-level mutation data into gene-level mutation data using the following procedure.

1. Binary mutation data
    a. We associate mutations in enhancer regions with mutations in their gene targets using the following procedure. We consider the set of enhancers with 5 or fewer predicted gene targets (syn7201027) with HUGO symbols[1], which includes 89.0% of scored enhancers. If a sample has a mutation in an enhancer, then we say that the sample has an enhancer mutation in each of the enhancer's predicted gene targets.
    b. For each gene, we say that a sample has a mutation in a gene if the sample has one or more mutations in one of the genes' coding and/or non-coding regions:
        i. Coding: a sample has one or more non-synonymous mutations in the coding elements of the gene.
        ii. Non-coding: a sample has one or more mutations in the core promoter, 5' UTR, 3' UTR, and/or enhancer elements of the gene.
        iii. Combined coding-and-non-coding: a sample has one or more mutations in the coding, core promoter, 5' UTR, 3' UTR, and/or enhancer elements of the gene.
2. Driver score *P*-values
    a. If there are multiple driver scores for the same element, then we use the minimum driver score on that element. For example, there are multiple *HOXC4* 3' UTR transcripts with nearly identical scores, and we use the smallest score. By only considering one score for each element, we reduce the number of tests, so there may be more genes with elements satisfying various FDR threshold than if we were to correct for the number of distinct transcripts.
    b. We associate driver scores for enhancer regions with driver scores for their gene targets using the following procedure. We consider the set of enhancers with 5 or fewer predicted gene targets (syn7201027) with HUGO symbols[2], which includes 89.0% of scored enhancers. If a gene is targeted by one or more enhancers,

[1] HUGO symbols from https://www.genenames.org: ftp://ftp.ebi.ac.uk/pub/databases/genenames/new/tsv/hgnc_complete_set.txt
[2] HUGO symbols from https://www.genenames.org: ftp://ftp.ebi.ac.uk/pub/databases/genenames/new/tsv/hgnc_complete_set.txt

than we assign the minimum driver score of that enhancers targeting that gene to that gene.

c. For each gene g, we have P-values on coding (p_{coding}(g)), core promoter (p_{promoter}(g)), 5' UTR ($p_{5'\text{UTR}}$(g)), 3' UTR ($p_{3'\text{UTR}}$(g)), and/or enhancer (p_{enhancer}(g)) regions associated with that gene.
d. We combine driver scores across multiple coding and/or non-coding elements using Fisher's method, i.e., $\chi^2_{2k} \sim -2 \sum_{p \in P} \ln(p)$, where P is a set of k P-values. Since the core promoter and 5' UTR elements overlap (syn8103141), we take the smaller of core promoter and 5' UTR p-values.
e. In particular, for gene g, we define the following coding, non-coding, and coding and non-coding driver scores using the following procedure:
    i. Coding scores: GS-C
    $p_{\text{C}}(g) = p_{\text{coding}}(g)$
    ii. Non-coding: GS-N
    $p_{\text{N}}(g) = \text{fisher}(\min(p_{\text{promoter}}(g), p_{5'\text{UTR}}(g)), p_{3'\text{UTR}}(g), p_{\text{enhancer}}(g))$
    iii. Combined coding-and-non-coding: GS-CN
    $p_{\text{CN}}(g) = \text{fisher}(p_{\text{coding}}(g), \min(p_{\text{promoter}}(g), p_{5'\text{UTR}}(g)), p_{3'\text{UTR}}(g), p_{\text{enhancer}}(g))$
f. If there is no driver score for a particular element of a particular gene, then we perform Fisher's method without the driver score. For example, if there is no 5' UTR score for gene g, then we compute $p_{\text{N}}(g) = \text{fisher}(p_{\text{promoter}}(g), p_{3'\text{UTR}}(g), p_{\text{enhancer}}(g))$, where there are $2 \cdot 3 = 6$ degrees of freedom for the chi-squared distribution in Fisher's method. Alternatively, if there is no 3' UTR score for gene g, then we compute $p_{\text{N}}(g) = \text{fisher}(\min(p_{\text{promoter}}(g), p_{5'\text{UTR}}(g)), p_{\text{enhancer}}(g))$, where there are $2 \cdot 2 = 4$ degrees of freedom for the chi-squared distribution in Fisher's method.

Gene-level expression data

We use gene-level and transcript-level expression data from the following sources:

1. gene-level expression data (syn5553991)
2. transcript-level expression data (syn7536588, syn7536589)
3. eQTL data (syn17096221)

We perform the following processing steps on gene-level expression data.

1. Obtain gene-level expression data (syn5553991) and gene-level copy-number data (syn8291899, syn8495585, syn8291804).
2. We retain mutations in a pan-cancer tumor cohort that excludes samples from the lymphoma and melanoma tumor cohorts, i.e., the Lymph-BNHL, Lymph-CLL, Lymph-NOS, and Skin-Melanoma cohorts, as well as 69 hypermutated samples with over 30 mutations/MB, which are listed by donor (syn7894281) or aliquot ID (syn7814911).
3. We consider the set of ENSEMBL IDs with HUGO gene symbols[3]. If multiple ENSEMBL IDs map to the same HUGO gene symbol, then we consider the mean expression across the multiple ENSEMBL IDs.
4. For each gene, we perform the following steps to correct for copy-number for methods:
    a. Calculate the Spearman rank correlation coefficient between the gene expression values and gene copy number values across patients.
    b. If the correlation is larger than 0.1 (or 0.2 or 0.3), then perform linear regression on gene expression values between the 5% and 95% quantile to reduce the influence of outliers, and use the residuals for this linear model as corrected gene expression values
    c. If the correlation is smaller than 0.1, then use the uncorrected gene expression values.

Pathway and network data

We used several pathway and network databases as input for gene-gene or protein-protein interactions for our analyses.

Pathway data

Pathway methods, those that make use of gene sets and ignore interactions, used sets of genes extracted from distinct categories or pathways from the following pathway databases:

1. CORUM (syn11426307)
2. GO (syn3164548)
3. InterPro (syn11426307)
4. KEGG (syn11426307)
5. NCI Nature (syn11426307)
6. Reactome (syn3164548)

[3] HUGO symbols from https://www.genenames.org:
ftp://ftp.ebi.ac.uk/pub/databases/genenames/new/tsv/hgnc_complete_set.txt

Network data

Our network methods use the following network interaction data:

1. the large connected subnetwork of the ReactomeFI 2015 interaction network (syn3254781) for the set of interactions with confidence scores ≥ 0.75, where each interaction is considered to be undirected
2. the largest connected subnetwork of the iRefIndex14 interaction network augmented with interactions from the KEGG pathway database, which we provide as a single interaction network (syn10903761)
3. the large connected subnetwork of the STRING v10 network (syn11712027), retaining only the high confidence edges (edge score > 9)

Other data

Some of the pathway and network analysis methods use additional data.

1. DIMA (see CanIsoNet data processing steps)
2. 3did (see CanIsoNet data processing steps)

Coding and non-coding mutations cluster on interaction networks

Earlier studies have demonstrated that proteins harboring coding driver mutations interact with each other in molecular pathways and networks significantly more frequently than expected by chance[1–3]. We asked whether a similar clustering is apparent among the genes with mutated non-coding elements on a protein-protein interaction (PPI) network. To this end, we counted the number of direct interactions among, and the subnetworks formed by, the 75 genes with the smallest coding driver *P*-values (FDR < 0.1; *P*-value = 3.4×10^{-4}; Supplementary Figure S2) in the network and found 44 interactions (2.6 interactions expected; $P < 10^{-6}$, permutation test of *P*-values) in a high-confidence subset of BioGRID PPIs[4]. While there were no direct interactions among the 8 genes with the highest non-coding driver scores (FDR < 0.1; *P*-value = 8.1×10^{-6}), the number of significant non-coding elements is appreciably lower. Therefore, to more compare the ranking of genes by coding and non-coding *P*-values and not these *P*-value distributions themselves, we counted the number of direct interactions between the subnetworks formed by the 75 genes with the smallest non-coding (promoter, 5' UTR, 3' UTR) driver *P*-values (FDR = 1; *P*-value = 2.7×10^{-3}) in the network and found 12 interactions (2.6 interactions expected; $P = 1.6 \times 10^{-3}$). Interestingly, when we further consider the top 75 genes from both the coding and non-

coding sets, we find that there are 37 interactions between them (5.8 expected; $P < 10^{-6}$). Taken together, network clustering reveals that genes with non-coding mutations significantly interact with, and are complementary to, the genes mutated primarily by coding mutations.

Pathway and network methods

The section briefly describes our pathway and network methods. The end of this document provides a more complete description.

Summary of methods

1. **ActivePathways:** ActivePathways evaluates the enrichment of mutations in functionally related sets of genes such as biological processes and molecular pathways. It analyses coding and non-coding driver scores of all genes and determines mutation enrichments of gene sets using non-parametric tests and *P*-value integration methods.
2. **CanIsoNet:** CanIsoNet uses an isoform-specific PPI network, binary mutation data, and transcript expression data from PCAWG and corresponding GTEx tissue samples to identify regions in a PPI network whose interactions are considered to be disrupted by alternative isoforms.
3. **Hierarchical HotNet:** Hierarchical HotNet uses interaction networks and gene mutation scores to find significantly mutated subnetworks. It simultaneously considers network topology and gene scores to construct a hierarchy of topologically close and highly mutated gene sets.
4. **Hypergeometric analysis:** This gene set enrichment analysis finds pathways that have a statistically significant number of genes whose scores exceed a fixed threshold.
5. **Induced subnetwork test:** The score-induced subgraph method uses interaction networks and gene mutations scores to find significantly mutated subnetworks. It finds subnetworks induced by the set of genes whose scores exceed a statistically determined threshold.
6. **NBDI:** NBDI uses a PPI network, binary mutation data, gene mutation scores, and gene expression data to find gene sets corresponding to potential driver genes that interact with differentially expressed genes. NBDI uses an integrated data model to combine these different data at the patient level.
7. **SSA-ME:** SSA-ME uses interaction network and gene mutation scores to find high scoring subnetworks in the interaction network. It subsequently prioritizes individual

genes using a score that reflects their likelihood of belonging to a high-scoring subnetwork.

Pathway and network data

Each of our methods use coding and/or non-coding somatic mutation data and one or more pathway databases or gene-gene or protein-protein interaction networks. Some of our methods also use expression or other data. Table S1 summarizes the data that our methods use.

Method	Driver score mutation data	Binary mutation data	Expression data	Prior pathway or network data
ActivePathways	✓			Gene Ontology (GO) biological processes, Reactome pathways
CanIsoNet		✓*	✓**	STRING v10, DIMA, 3did
Hierarchical HotNet	✓			ReactomeFI 2015, iRefIndex14+KEGG
Hypergeometric analysis	✓			GO biological processes, CORUM, InterPro, Nature NCI, KEGG pathways
Induced subnetwork analysis	✓			ReactomeFI 2015, iRefIndex14+KEGG
NBDI	✓***	✓***	✓****	ReactomeFI 2015
SSA-ME	✓			ReactomeFI 2015

Table S1: Summary of the mutation, expression, and pathway/network data used by each of the seven analysis methods.

*Includes splice-site and other mutation data

**Transcript-level expression data, eQTL data

***Excludes 5' UTR mutation data

****Gene-level expression data

Analysis of individual gene scores

To evaluate the effects of non-coding compared to coding mutations, each method performed distinct analyses of coding, non-coding (UTRs, promoters, enhancers), and combined coding-and-non-coding gene scores (GS-C, GS-N, and GS-CN, respectively).

Non-coding value-added (NCVA) procedure

The identification of subsets of driver genes by each of the respective network or pathway-based methods is driven by the clustering of mutations in a network neighborhood or pathway. However, where it is well known that mutations in coding regions cluster in networks and pathways, this clustering is weaker for mutations in non-coding regions. Combining the coding and non-coding signal in one analysis has as advantage that both signals can complement each other and improve the detection of weaker network/pathway signals in the network. In order to separate the contributions of the coding and non-coding data in this combined analysis, we perform the following analysis, which allows us to identify a subset of the results on combined coding-and-non-coding data for which the non-coding data makes a statistically significant contribution to the discovery of the genes in the subset. A method's non-coding value-added (NCVA) results augment or extend its N results.

Specifically, we compute a *P*-value for each gene from a method's results on combined coding-and-non-coding data to quantify the statistical significance of the contribution of the non-coding data while conditioned on the coding data. For each gene g that a method identifies on observed coding and observed non-coding data, we compute an empirical p-value P_g as the fraction of times that the method identifies gene g on a dataset consisting of the combined observed coding and permuted non-coding data. In particular, if we consider the collection of driver score *P*-values as a matrix where genes are rows and elements (coding, promoter, 5' UTR, 3' UTR, enhancer) are columns, then we permute the sub-rows of this matrix corresponding to non-coding elements, which preserves correlations between scores on non-coding elements.

For example, after permuting the driver score *P*-values on non-coding elements, the scores in Table S75A may become the scores in Table S27B:

Observed driver scores:

Gene	Coding	Promoter	5’ UTR	3’ UTR	Enhancer
A	0.1	0.2	0.3	0.4	0.5
B	0.6	0.7	0.8	0.9	1.0

Table S27A: Example of observed coding and observed non-coding driver scores

Observed coding driver scores and permuted non-coding driver scores:

Gene	Coding	Promoter	5’ UTR	3’ UTR	Enhancer
A	0.1	0.7	0.8	0.9	1.0
B	0.6	0.2	0.3	0.4	0.5

Table S27B: Example of observed coding and permuted non-coding driver scores

Given a significance threshold α, if *A* is the set of genes in a method’s results on combined coding-and-non-coding data, then we define the set $B_\alpha = \{g \in A: p_g < \alpha\}$ as the subset of the method’s combined coding-and-non-coding results for which the non-coding data makes a statistically significant contribution to the discovery of the genes in the subset. We set $\alpha = 0.1$ for our analysis, and we define B_α as the NCVA results for a method.

Figure S3 illustrates several reasons why a method may identify a gene as a NCVA gene:

1. The central node has a high non-coding score, and its neighbors have high coding scores. It is detected as part of a large subnetwork on observed data but not on permuted non-coding data because it no longer has a high non-coding score on permuted data, so it is a NCVA gene.
2. The central node has a high coding score, and its neighbors have high non-coding scores. It is detected as part of a large subnetwork on observed data but not on permuted non-coding data because its neighbors no longer have high non-coding scores on permuted data, so it is a NCVA gene.
3. The central node has a moderately high coding and non-coding score, and its neighbors have high coding and/or non-coding scores. It is detected as part of a large subnetwork

on observed data but not on permuted non-coding data because it no longer has a moderately strong non-coding score on permuted data, so it is a NCVA gene.

Individual pathway analysis algorithms

This section describes the statistical significance and calibration results of our methods. When evaluating statistical significance and calibration, we preserve the distribution of the observed driver score *P*-values, which is more conservative than considering randomized driver score *P*-values from the PCAWG consensus driver analysis.

ActivePathways

Jüri Reimand (OICR)
Juri.Reimand@oicr.on.ca

ActivePathways [manuscript in prep] evaluates a ranked list of genes ordered by decreasing significance of driver scores and performs pathway enrichment analysis using the ordered hypergeometric statistic. The ordered hypergeometric statistic considers one pathway gene set at a time and performs a hypergeometric test with increasingly larger subsets of the ranked gene list starting from the most significant gene in the list.

ActivePathways was separately executed for three lists of candidate genes: genes with driver scores of coding mutations, non-coding (promoter, 5' UTR, 3' UTR) mutations, and with the combined coding-plus-non-coding mutations. *P*-values across multiple elements per gene were merged into one *P*-value using Brown's procedure[5]. Input gene lists were filtered to include genes with lenient *P*-values (uncorrected $P < 0.1$) and ranked in order of decreasing significance of merged *P*-values. The ranked hypergeometric enrichment analysis considered biological processes from Gene Ontology and molecular pathways from Reactome as gene sets (GO terms from Ensembl version 87, Reactome version 56). Resulting significantly enriched pathways were removed (FDR < 0.05).

The method was benchmarked on randomly simulated driver scores that were obtained by shuffling gene names among driver scores (Supplementary Figure S14). Permutation analysis was conducted on non-coding scores by randomly reassigning scores among genes for 10,000 times.

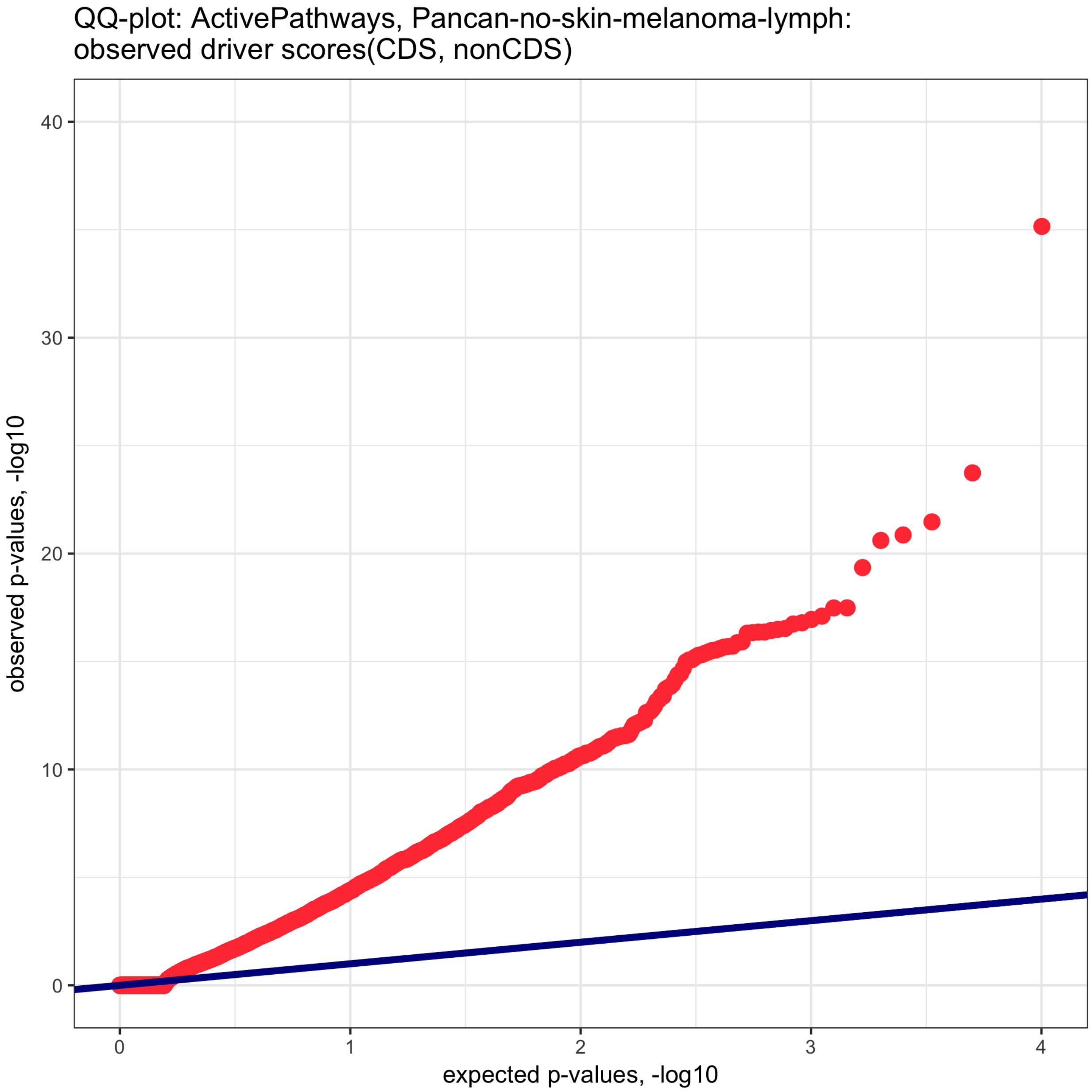

QQ-plot: ActivePathways, Pancan-no-skin-melanoma-lymph:
observed driver scores(CDS, nonCDS)
observed p-values, -log10
expected p-values, -log10
40
30
20
10
0
0
1
2
3
4

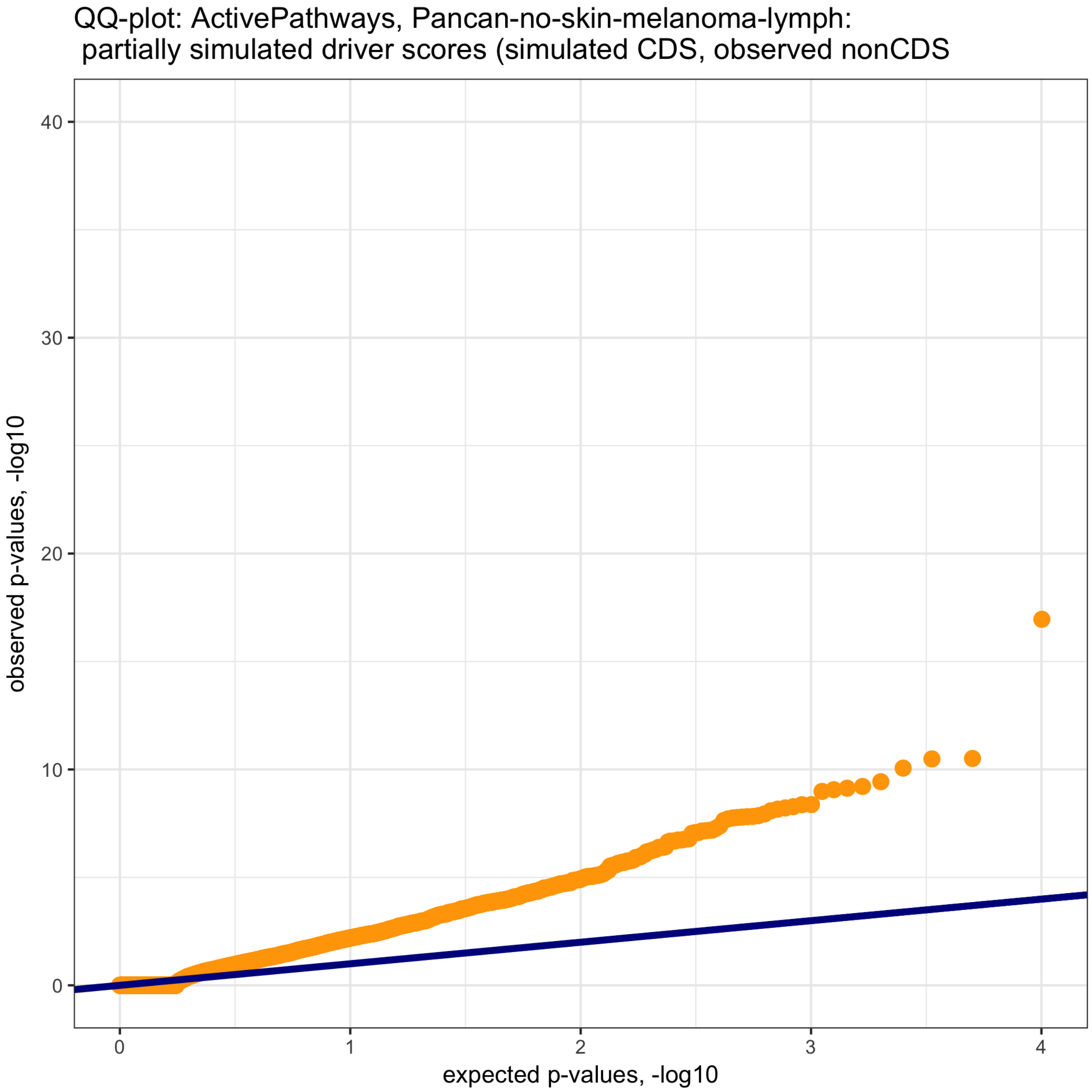
QQ-plot: ActivePathways, Pancan-no-skin-melanoma-lymph:
partially simulated driver scores (simulated CDS, observed nonCDS
observed p-values, -log10
expected p-values, -log10
0
10
20
30
40
0
1
2
3
4

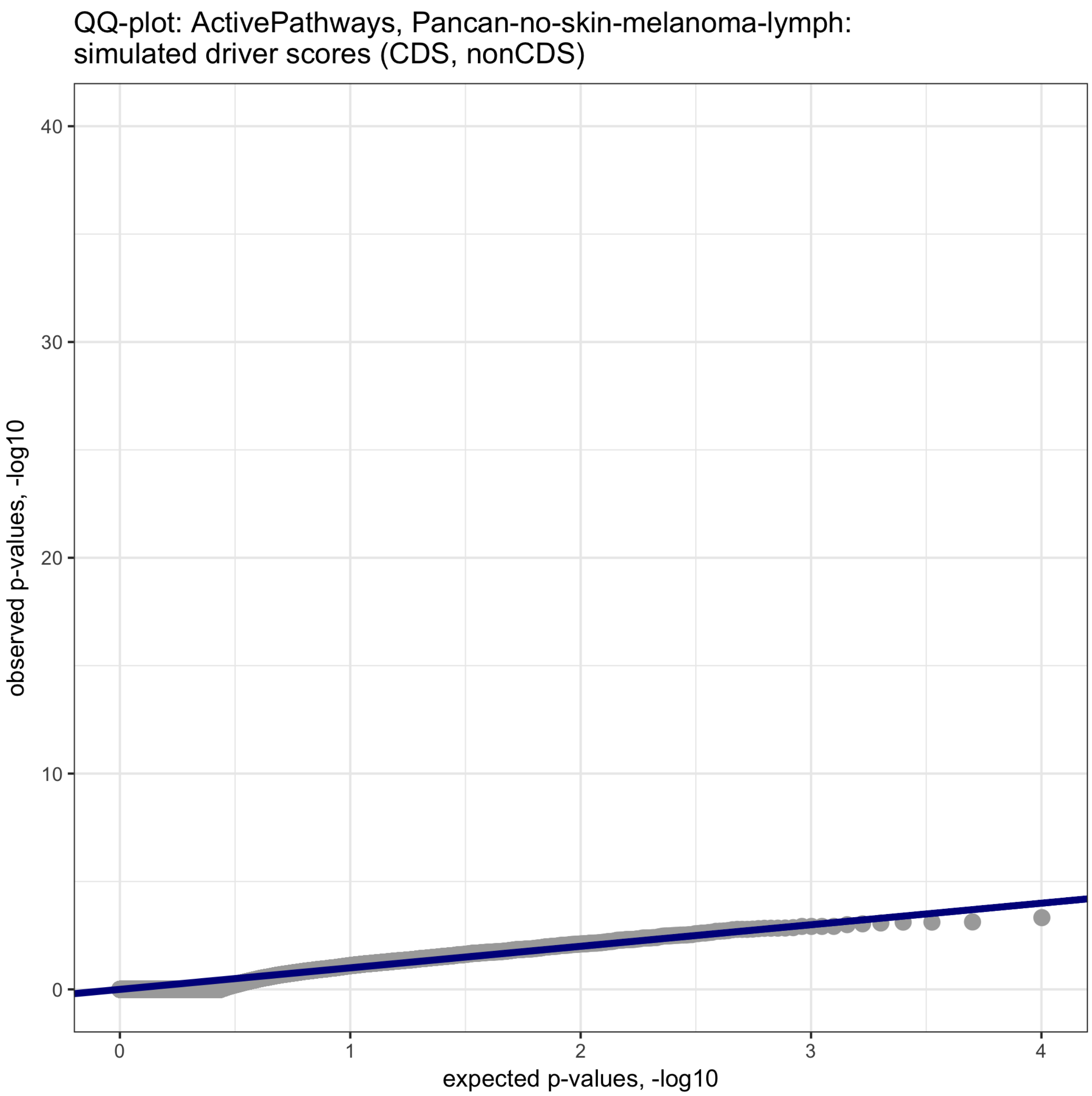

Figure S14: ActivePathways QQ plots. P-values from ActivePathways representing statistical enrichment of functional gene lists among cancer driver gene scores were evaluated in three analyses: observed coding and non-coding driver gene scores (top, red); simulated coding and observed non-coding driver gene scores (middle, orange); and simulated coding and simulated non-coding driver gene scores (bottom, grey). *P*-values were computed across Gene Ontology and Reactome gene sets. Observed driver gene scores (red, top) show statistical inflation due to highly redundant gene sets that break the assumption of independent tests in QQ-plots. Simulation of protein-coding driver gene scores combined with observed non-coding driver gene scores (orange, middle) reduces the extracted signal and apparent statistical inflation as many

fewer cancer pathways are detected. Simulated coding and non-coding driver gene scores (bottom, gray) show no statistical inflation and follow the expected diagonal in QQ-plots.

CanIsoNet

Abdullah Kahraman, Christian von Mering
abdullah.kahraman@uzh.ch, mering@imls.uzh.ch

The CanIsoNet method utilizes a novel isoform-specific protein-protein interaction database to automatically assess the pathological impact of alternatively spliced isoforms on a genome and proteome-wide scale. CanIsoNet analyzes the degree of destruction that an alternative spliced isoform induces on a protein-protein interaction network when it is differential used in a cancer cell (manuscript in preparation).

Cancer-type-specific most dominant transcripts

The impact of differential alternative splicing on cancer cells was studies by comparing the Most Dominant Transcripts (MDT) in primary cancer samples with MDT in corresponding normal tissues from the GTEx project. The aim was to identify pathological cancer-type-specific MDTs (cMDT) that rewire interaction networks by over-expressing binding site lacking transcripts that disrupt protein interactions. To identify such MDT switches, Transcript Per Million (TPM) counts were computed for all Ensembl transcripts (v.75) in 1393 PCAWG (syn7536587) and 3249 GTEx RNAseq samples (syn7596599) using Kallisto (v.0.42.1) with default parameters (see PCAWG-3 paper for more details). 184 PCAWG samples were filtered out as non-tumor or blacklisted samples (syn7416381), while 1017 GTEx samples were removed that had no matching tissue type in PCAWG (syn7596611). Transcripts were called MDT in a sample, only if they had at least a two-fold expression difference to any other alternative transcript from the same gene in the same sample. A cMDT was identified, if it was unique to PCAWG and its expression was significantly different to its expressions in the corresponding normal tissue samples in GTEx. To study the degree to which a cMDT rewires an interaction network, a novel isoform-specific interaction network was developed as described next.

Development of an isoform-specific interaction database

For the isoform-specific interaction network, human STRING protein-protein interactions (version 10.0) (1) with a STRING combined score ≥ 900 were retrieved and enriched with domain-domain interaction information from the DIMA database (v. 3.0) (2) and the 3did database (v.2016_06) (3). The domain interaction information was used to identify the molecular regions driving the interaction between protein pairs and label protein domains as binding site containing domains. For the integration PFAM (4) domain annotations (version 27.0) in the STRING database were updated by running the pfam_scal.pl script (version 1.5, downloaded from PFAM.org) on the canonical isoform sequences in STRING. Interacting STRING proteins having PFAM domains with binding site information were regarded as physical interactions having molecular region information on the interaction origin.

To create an isoform-specific interaction database, a compendium of all known human transcripts was downloaded from the ENSEMBL database (version 75, which was also used by the latest STRING database). Protein isoforms having all binding site containing domain sequences of their canonical isoform sister were considered to not disrupt interactions. While alternative isoforms lacking one or more amino acids of the binding site containing domain were predicted to disrupt protein interactions. A table of protein isoforms - protein isoform interactions can be found in the Synapse repository (syn10245952).

Predicting the pathological impact of MDT switches

To predict the pathological impact of cMDT, we assessed their proximity to 595 genes from the COSMIC gene census list (v77) in the domain enriched STRING interaction network and checked whether they were located in densely populated network regions, following the idea that cMDT with pathological impact should disrupt many interactions. For the cancer gene proximity calculations, we computed the shortest path with a breadth-first-search algorithm between a cMDT and all known COSMIC census genes within the aforementioned isoform-specific interaction network. The shortest path results can be found in Synapse (syn9770515). cMDTs were significantly closer to COSMIC census genes than random, with 65% being a

consensus gene themselves or an interaction partner (manuscript in preparation). Next, we computed a Network Density Score (*NDS*) at each network node *A* using the following formula:

$$NDS(A) = int(A) + \sum_{s=1}^{3}\sum_{b=1}^{B}\frac{1}{2^s} * int(b_s) * score(b_s, b_{s-1})$$

where *A* is the gene of interest, *b* is an interactor being *s* interactions apart, *int()* is the number of interactors of *b* and *score()* is the STRING combined interaction score between *b* and its interaction partners. The minimum NDS was set to the rank position 70%, which covered 50% of COSMIC census genes and their direct interaction partners, while including only 3% of NON-COSMIC genes. The minimum NDS value corresponds to the top 30% densest network regions in the entire STRING-DIMA network.

Predicting causative mutations for most dominant transcript switches in cancer cells

We identified from the list of consensus coding and non-coding mutation calls (syn8103141) from the PCAWG-1 working group, those that were located in functional regions (promoter, 5'UTR, splice site, 3'UTR and enhancer) (syn7345646) of cMDT affected genes. These mutations were considered as potential cMDT causing mutations. Truly causative non-coding mutations for each cMDT were identified for those genes for which non-coding mutations could be found in cis- and trans-eQTL regions (multicomparison adjusted p-value ≤ 0.05) of the affected genes. eQTL data were provided by the PCAWG-3 working group (syn8047691). Mutations that fall within intron, promoter and enhancer eQTL regions were selected as causative non-coding mutations inducing cMDT switches.

Network consensus run

For the network consensus run, we took cMDT that had associated coding and non-coding mutations as described in the previous section and listed for the cMDT all protein interactions that are lost due to the lack of binding site contain protein domains. A subnetwork consists of the cMDT and the lost interaction partners and is scored by the percentage of interaction lost that the cMDT induces by its over-expression relative to the canonical transcript. For the coding consensus run, we considered only cMDT with associated coding and splice site mutations.

While for the non-coding consensus run, we considered cMDT with associated non-coding mutations in promoter, 5'UTR, 3'UTR and enhancer functional regions. All results were uploaded to Synapse (syn21413360).

For each gene in Ensembl (v75), CanIsoNet compares the normalized expression values (Transcripts per Million [TPM]) of its transcripts and calls the most dominant transcript (MDT) for the gene if that transcript has 2 fold higher expression than any other transcript of the same gene. Following this procedure, MDT were identified for 1209 PCAWG and 2232 GTEx samples for which RNAseq data was available. An MDT in a PCAWG sample was called cancer specific, if it was unique to PCAWG while having an expression that was different (FDR < 0.1) from its expressions in all GTEx samples from the same primary organ. In total, 17,146 cancer specific MDTs were found. For the significance test, the standard sign test was used as follows: a vector was constructed for each MDT in PCAWG, where the vector elements corresponded to the difference between the TPM of the PCAWG sample and the TPMs of all GTEx samples originating from the same primary organ. For each MDT the number of positive and negative differences were summed up and a p-value was computed using R's binom.test function (two-sided and 0.5 hypothesized probability of success). Subsequently a multiple testing correction was performed using the Benjamini-Hochberg FDR method (R function p.adjust). No further calibration was performed as the sign test is well calibrated.

Hierarchical HotNet

Matthew Reyna (Princeton), Max Leiserson (University of Maryland), Ben Raphael (Princeton)
mreyna@princeton.edu, mdml@cs.umd.edu, braphael@princeton.edu

Hierarchical HotNet is a method for finding *de novo* subnetworks of significantly mutated genes in cancer[6]. It uses both network topology and gene mutation scores to cluster genes hierarchically, identifying statistically significant, topologically close, and significantly mutated gene sets.

For our analysis, we used the ReactomeFI 2015 interaction network[7] and the iRefIndex14 interaction network[8] augmented with KEGG pathways[9], where we retained high-confidence interactions in the ReactomeFI 2015 network with scores of 0.75 or higher without considering interaction direction. We also used the driver score *P*-values for the Pancan-no-skin-melanoma-lymph tumor cohort [syn8494939], where we generated gene-level *P*-values for

our analysis using the procedure described in the mutation data processing section. We further thresholded the gene scores by assigning $P = 1$ to all genes with $P > 0.25$.

We ran Hierarchical HotNet using each of the two above interaction networks and each set of gene scores (coding, non-coding, and combined coding plus non-coding) for a total of six runs. Hierarchical HotNet uses a permutation test to evaluate the statistical significance and effect size of the observed hierarchy of gene sets by comparing the subnetwork sizes in the hierarchy for observed and permuted data. For each of our runs, we permuted the assignment of gene scores to network nodes 1000 times, and we reported a representative subnetwork from the hierarchy corresponding to the maximum ratio between observed and expected subnetwork sizes for the consensus analysis.

For each set of driver scores and each interaction network, we compute a *P*-value $P = \Pr(X \geq x)$, where X is a random variable for the maximum ratio between the largest observed and expected subnetwork sizes and x is the observed value of this statistic. We generate these *P*-values empirically using 1,000 permutations of the driver scores within the network. Table S28A shows that Hierarchical HotNet produces statistically significant results on each set of driver scores (coding, non-coding, and combined coding-and-non-coding), and each of the two interaction networks (ReactomeFI 2015 and iRefIndex14+KEGG) that we considered.

Gene scores (two networks)	Observed maximum ratio x	Expected maximum ratio $E[X]$	*P*-value: $\Pr(X \geq x)$
Coding (GS-C)	15.1<br>19.1	1.72<br>1.69	< 0.001<br>< 0.001
Non-coding (GS-N)	3.1<br>5.9	1.67<br>1.67	0.033<br>< 0.001
Combined coding-and-non-coding (GS-CN)	14.3<br>19.6	1.71<br>1.69	< 0.001<br>< 0.001

Table S28A: Hierarchical HotNet observed and expected test statistics (maximum ratio of largest subnetwork sizes), and empirical *P*-value for maximum ratio; top value in each cell corresponds to ReactomeFI 2015 network and bottom value corresponds to iRefIndex14+KEGG network.

For each set of driver scores and each interaction network, we also tested the calibration

of our method by computing the distribution of P-values $P = \Pr(X \geq y)$, where y is a value of this statistic on permuted data. We generate these P-values empirically using 1,000 permutations of the gene scores within the network and compare the resulting distribution of empirical P-values with the uniform distribution. Table S28B shows that Hierarchical HotNet produces calibrated results on each set of driver scores (coding, non-coding, and combined coding-and-non-coding), and each of the two interaction networks (ReactomeFI 2015 and iRefIndex14+KEGG) that we considered. Figure S15 illustrates these distributions with a Q-Q plot for permuted coding driver scores for the ReactomeFI 2015 interaction network.

Gene scores (two networks)	K-S test *P*-value vs. uniform distribution
Coding (GS-C)	0.62<br>0.16
Non-coding (GS-N)	0.89<br>0.69
Combined coding-and-non-coding (GS-CN)	0.46<br>0.066

Table S28B: Hierarchical HotNet Kolmogorov-Smirnov (K-S) test *P*-value vs. uniform distribution; top value in each cell corresponds to ReactomeFI 2015 network and bottom value corresponds to iRefIndex14+KEGG network.

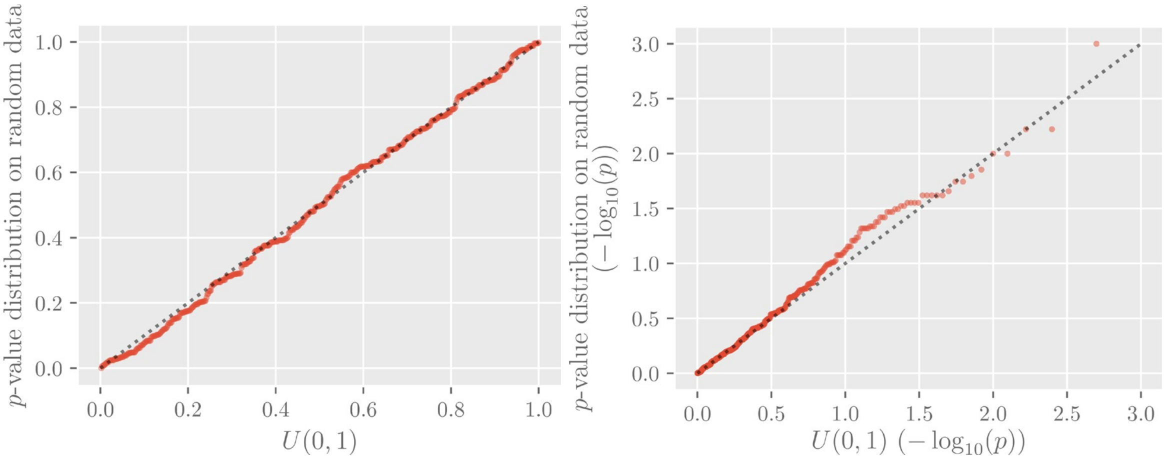

Figure S15: Hierarchical HotNet Q-Q plots of empirical P-value distribution on permuted data (linear scale on left and logarithmic scale on right); permuted coding driver scores on observed ReactomeFI 2015 network.

Hypergeometric analysis

Miguel Vazquez
miguel.vazquez.g@bsc.es

This analysis uses the hypergeometric distribution to perform a gene set enrichment analysis of high-scoring genes in predefined gene sets. We considered three sets of driver scores (coding, non-coding, and combined coding-and-non-coding) and five gene set databases (GO biological processes, CORUM, InterPro, Nature NCI, KEGG pathways). For each set of driver scores and each gene set from one of these databases, we compute the hypergeometric *P*-value of observing high-scoring genes (driver score $P < 0.1$) in the gene set. For this calculation we used the 'phyper' function in the R statistical language. We perform a Benjamini-Hochberg correction for each set of gene scores and pathway database, and we report the union of genes in significantly enriched gene sets (FDR < 0.1).

For the purpose of assessing the validity of the method we performed 100 randomizations of the driver gene scores and computed the enrichment for the entire list of 'biological process' GO terms, using the same criteria as above (driver score $P < 0.1$). Figure S16 shows a qq-plot of these *P*-values (not FDR adjusted) against the uniform distribution using the original and the log-transformed data. The horizontal stretches present in the plots are caused by assigning a p-value of 1 to all the gene sets that were not reported in the original analyses because they received no hits. The difference in steepness apparent on the log-transformed plot between the qq-plot line and the lines from the 100 randomizations are caused by the fact that the different GO terms can show large overlaps and thus their respective statistical tests are not independent.

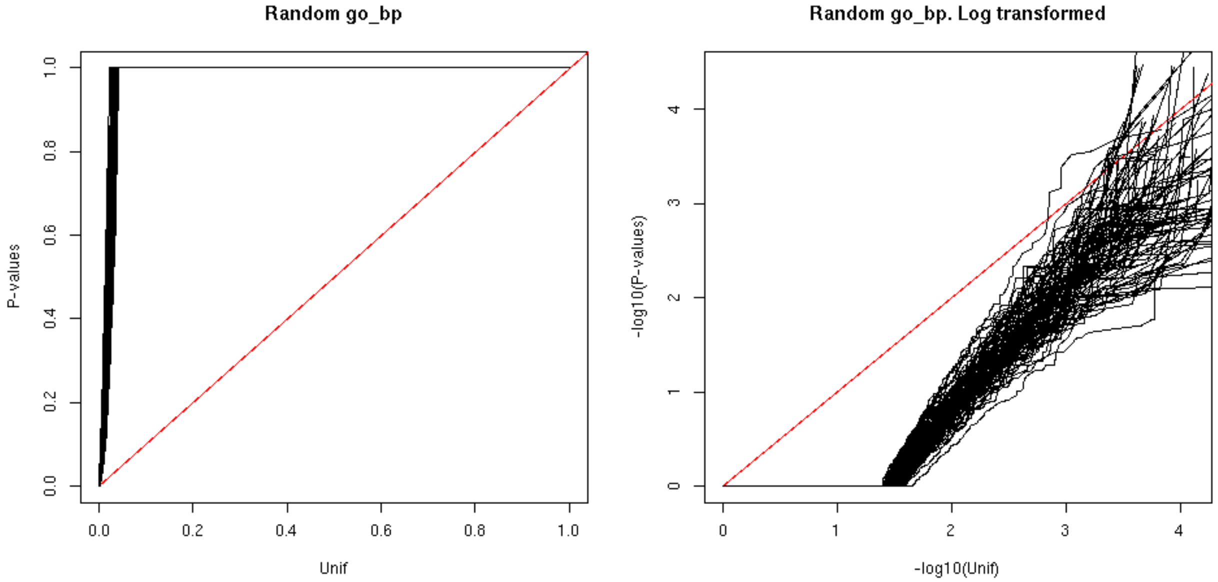

Figure S16: *P*-values for all 'biological process' GO terms plotted against the uniform distribution, for 100 different randomizations of driver gene scores. The right panel shows the data log-transformed. Terms that were not represented in the input gene list have been assigned a *P*-value of 1.

Induced subnetwork analysis

Matthew Reyna (Princeton), Ben Raphael (Princeton)
mreyna@princeton.edu, braphael@princeton.edu

We developed a method to identify subnetworks of a gene-gene or protein-protein interaction network that are induced by sets of highly mutated genes. This method uses a statistics-driven approach to find clusters of topologically close and significantly mutated gene sets.

For our analysis, we used the ReactomeFI 2015 interaction network [7] and the iRefIndex14 interaction network [8] augmented with KEGG pathways [9], where we retained high-confidence interactions in the ReactomeFI 2015 network with scores of 0.75 or higher without considering interaction direction. We also used the driver score *P*-values for the Pancan-no-skin-melanoma-lymph tumor cohort [syn8494939], where we generated gene-level *P*-values for our analysis using the procedure described in the mutation data processing section. We further thresholded the gene scores by assigning $P = 1$ to all genes with $P > 0.25$.

We ran this method using each of the above two networks and each set of gene scores (coding, non-coding, and combined-coding-plus-non-coding) for a total of six runs. For each distinct gene score, this method finds the number of edges in the subnetwork induced by the set

of genes with as large or large scores. The method then computes the maximum ratio of the number of edges on the observed data to the expected number of edges on permuted data across all distinct gene score. The method finally computes an empirical *P*-value comparing the ratio on observed data with the ensemble of ratios on permuted data. For each of our runs, we permuted the assignment of gene scores to network nodes 1000 times, and we reported a representative subnetwork for the gene score threshold corresponding to the maximum ratio between observed and expected number of edges for the consensus analysis.

For each set of gene scores and each interaction network, we compute a *P*-value $P = \Pr(X \geq x)$, where X is a random variable for the maximum ratio between the observed and expected number of edges in the subgraph induced by the high-scoring nodes and x is the observed value of this statistic. We generate these *P*-values empirically using 1,000 permutations of the gene scores within the network. Table S29A shows that the induced subnetwork analysis produces statistically significant results on each set of gene scores (coding, non-coding, and combined coding-and-non-coding), and each of the two interaction networks (ReactomeFI 2015 and iRefIndex14+KEGG) that we considered.

Gene scores (two networks)	Observed maximum ratio x	Expected maximum ratio E[X]	*P*-value: $\Pr(X \geq x)$
Coding (GS-C)	16.9<br>52.6	1.50<br>1.80	< 0.001<br>< 0.001
Non-coding (GS-N)	5.8<br>6.5	1.49<br>1.81	< 0.001<br>0.009
Combined coding-and-non-coding (GS-CN)	14.9<br>41.6	1.50<br>1.83	< 0.001<br>< 0.001

Table S29A: Induced subnetwork analysis observed and expected test statistics (maximum ratio of largest subnetwork sizes), and empirical *P*-value for maximum ratio; top value in each cell corresponds to ReactomeFI 2015 network and bottom value corresponds to iRefIndex14+KEGG network.

For each set of gene scores and each interaction network, we also tested the calibration of our method by computing the distribution of *P*-values $P = \Pr(X \geq y)$, where y is a value of this statistic on permuted data. We generate these *P*-values empirically using 1,000 permutations of

the gene scores within the network and compare the resulting distribution of empirical *P*-values with the uniform distribution. Table S29B shows that the induced subnetwork analysis produces calibrated results on each set of gene scores (coding, non-coding, and combined coding-and-non-coding), and each of the two interaction networks (ReactomeFI 2015 and iRefIndex14+KEGG) that we considered. Figure S17 illustrates these distributions with a Q-Q plot for permuted non-coding driver scores for ReactomeFI 2015 network.

Gene scores<br>(two networks)	K-S test *P*-value vs. uniform distribution
Coding (GS-C)	0.066<br>0.62
Non-coding (GS-N)	0.014<br>0.16
Combined coding-and-non-coding (GS-CN)	0.032<br>0.19

Table S29B: Induced subnetwork analysis Kolmogorov-Smirnov test *P*-value vs. uniform distribution; top value in each cell corresponds to ReactomeFI 2015 network and bottom value corresponds to iRefIndex14+KEGG network.

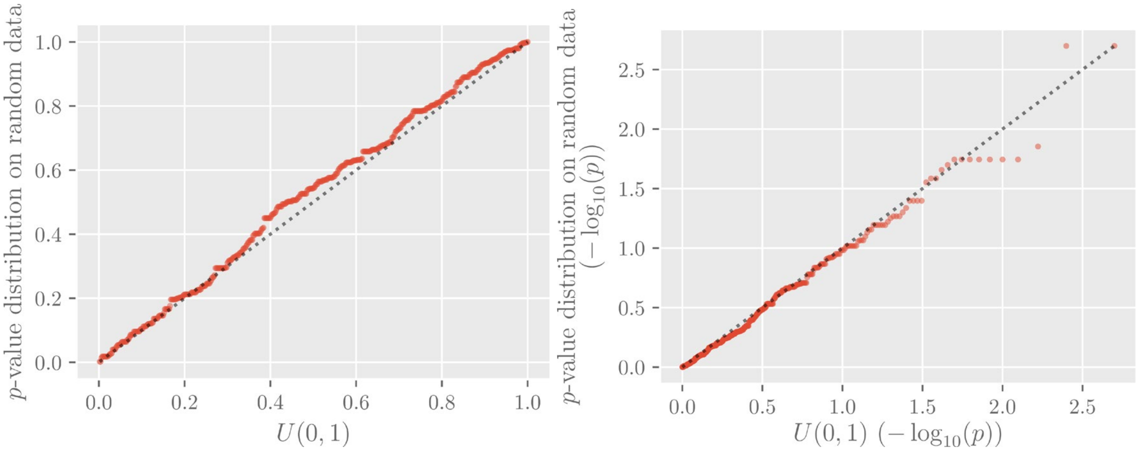

Figure S17: Induced subnetwork analysis Q-Q plots of empirical P-value distribution on permuted data (linear scale on left and logarithmic scale on right); permuted non-coding gene scores on observed ReactomeFI 2015 network.

NBDI

Lieven Verbeke

Lieven.Verbeke@UGent.be, Kathleen.Marchal@UGent.be

Network-based data integration (NBDI) is a unique data integration model[10] that allows for the ranking of pathways according to their importance for contrasting phenotypes. Key to the method is the conversion of all available data to a network representation that interconnects both genes and samples. After the conversion to a network model, the impact of the different genetic and transcriptomic aberrations is diffused through this network. The method can include any type of omics data and because the samples are part of the data model, inferences about individual samples or subsets of samples of interest can be made. In this work, NBDI was extended to allow for the identification of groups of potential driver genes. The network model relies on prior knowledge in the form of known gene interactions (physical or functional) in order to connect genetic aberrations with differentially expressed genes. We used Reactome Functional Interactions, a manually curated, high quality and publicly available source of gene interactions[11]. Hyperconnected genes with more than 2000 interactions were removed, as well as the cluster of olfactory receptor genes.

The integrated data model was constructed using coding mutations, core-promoter mutations **(as defined in syn21416282)**, 3' UTR mutations and gene expression data. 5'UTR variants were omitted because the significant overlap with core promoter variants would unfairly favor these variant types. Gene expression data **(syn5553991)** (including sparsely available gene expression in normal tissue) were median centered per tissue type, after which a log-transformation was applied. We proceeded with the detection of deviating gene expression by exploiting the asymmetry of the expression signal. First the 5% and 95% quantiles are found. Next the side of the distribution containing either the 5% or 95% quantile that is closest to the median observed gene expression values is considered normal, after which that quantile is mirrored around the median and all values in the long tail of the gene expression distribution that are larger (respectively smaller) than the mirrored value are considered exceptional and set to one. All other values are set to zero. Mutations (per data type) were preprocessed as follows. (1) Consensus variant calls for whitelisted samples **(syn7894281)** were filtered using BED annotations as established within the ICGC PAWG project **(syn8103141)**. Malignant lymphoma and melanoma samples were removed because of the abundance of the hypermutator phenotype in these cancer types. (2) Filtered variants were further annotated, per data type, with driver *P*-values **(syn8494939)**. (3) Variants with a variant allele frequency smaller than 0.1 were removed. (4) Individual variants are collapsed to the gene level, retaining the minimum observed p-value per gene. (5) Minus $\log_{10}$(p)-values were transformed to scores in the interval

[0,1] by setting values below 0.1 to 0, thresholding all values above 10, and dividing all transformed scores by the maximum observed value (<=10). (6) Hyper-mutated samples **(syn7894281/syn7814911)** were removed from the resulting variant matrices, as well as genes with fewer than 5 variants in the resulting variant matrices. (7) All datasets were intersected, resulting in a total of 988 pan-cancer samples for which both variant data and gene expression data were available.

After constructing the integrated data model, a connectivity matrix was calculated in order to assess how well connected all entities in the model (genes and samples alike) are connected to all other entities. A random-walk-with-restart was used to derive a connectivity measure[12] running with a fixed probability of restarting the random walk of 0.1. For each potential driver gene in the coding, core promoter and 3'UTR datasets, we extracted that part of the connectivity matrix that expresses how well a genomic variant (e.g., a coding mutation) is connected to transcriptomic variants (abnormal over- or under-expression) of a small network neighborhood (2 hops) centered around the considered driver gene (including the considered gene itself). By taking the median of the extracted connectivity measures, a *base-score* is obtained that can be used to prioritize drivers. The rationale for the proposed approach is the following: a gene exhibiting genomic variations (coding or non-coding variants) will be easily connected to genes exhibiting transcriptomic variation if (1) genomic and transcriptomic variants of the same gene occur in the same samples (as then genomic variants are connected to transcriptomic variants through interconnecting sample nodes) (2) transcriptomic variants occur in the immediate network neighborhood of genomic variants, as than paths connecting genetic cause with transcriptomic consequences can be constructed through a combination of shared sample nodes and gene interactions. The entire model building and scoring procedure is repeated using randomized input data. Variants are permuted across genes and samples, and gene labels are permuted. Gene expression data and gene labels remain unshuffled. To avoid inflating scores for genes that are present in the network when shuffling gene labels, the algorithm guarantees an identical number of genes to be present in the network when using real or shuffled gene labels. Using the randomized data, we obtain a background distribution that can be used to calculate final empirical p-values for each gene/data type combination. In order to assess the added value and robustness of the non-coding variants identified this way, we applied an additional permutation scheme.

The procedure described above (calculating scores using real data and fully permuted data) was repeated 100 times, but each time the base-score is obtained using non-permuted transcriptomic data and coding variants, while the non-coding (core-promoter and 3'UTR) data

are permuted. The base-score obtained with permuted non-coding data is then compared with a full permutation (wherein all data types are permuted) to obtain an empirical p-value for each gene per datatype per run. Finally, the collected p-values are used to calculate an empirical p-value for the originally obtained results (obtained using full non-permuted input data).

For each gene with coding, promoter, 3’ UTR, and/or enhancer mutations, we compute a score for how well each of these sets of mutations is connected to a local set of differentially expressed genes. We compute an empirical *P*-value for each gene and set of mutations using a permutation scheme in which driver scores, gene labels, and sample labels are permuted. This permutation scheme does preserves driver score distributions but not gene mutation frequencies. We report genes with FDR < 0.1.

We show that these empirical *P*-values are not biased under the null-hypothesis (i.e., the data do not contain any signal) by comparing the distribution of *P*-values obtained using random data (cfr. the permutation scheme described in the NBDI method section) against the uniform distribution *U*(0, 1). See Figure S18 for QQ-plots that compare the distribution of empirical *P*-values from observed and random data against the uniform distribution.

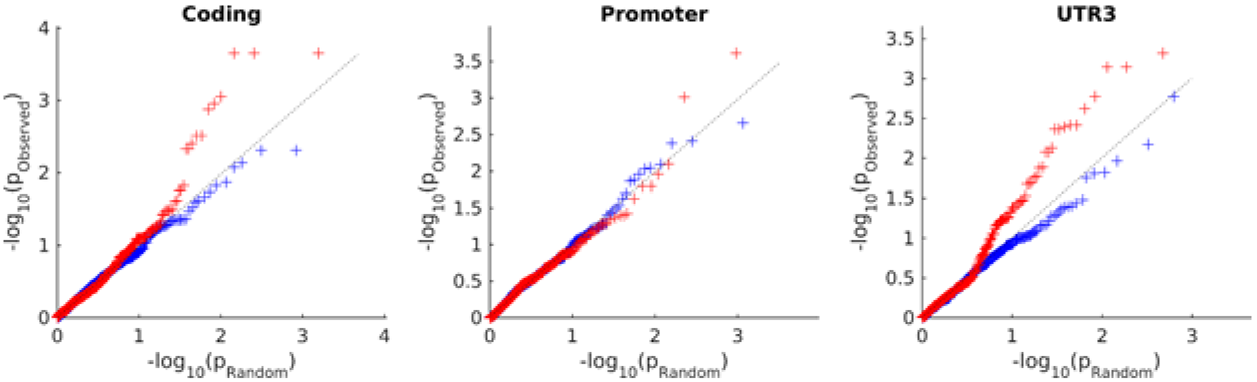

Figure S18: Q-Q plots for Coding, Promoter and 3’UTR *P*-values obtained using real data (red) and random data (blue) for the NBDI analysis.

Using both an Kolmogorov-Smirnov and an Anderson-Darling test (with the latter having better power against inflated tails and the former having more power against deviations in the middle of the distribution), we evaluated whether the obtained random distributions deviate significantly from the theoretical uniform distribution. Table S30 shows that the random data result in *P*-value distributions that do not differ significantly from the uniform distribution (i.e. $p>0.05$). When using real data, only for the 3’UTR data do *P*-values deviate from the uniform distribution.

		p_{KS}	p_{AD}
Coding	real	2.66E-01	5.32E-01
	Random	7.15E-01	5.70E-01
Promoter	Real	1.61E-01	3.22E-01
	Random	1.08E-01	2.16E-01
3'UTR	Real	3.13E-02*	6.25E-02
	Random	1.57E-01	3.14E-01

Table S30: Testing deviations from the theoretical uniform distribution of p-values when using real and random data as input for the NBDI analysis (see text). p_{KS} = Kolmogorov-Smirnov test, p_{AD} = Anderson-Darling test.

SSA-ME

Sergio Pulido-Tamayo (Ghent University) - Kathleen Marchal (Ghent University)
spulido99@gmail.com, Kathleen.marchal@ugent.be

SSA-ME [27808240] searches in an interaction network for small high scoring subnetworks. The score of a subnetwork is defined by the score contributions of the individual genes belonging to the subnetwork. The underlying assumption being that genes that belong to such high scoring subnetwork are more likely drivers. SSA-ME solves the complex problem of detecting high scoring subnetworks using a reinforcement learning framework by searching the local neighborhood around a set of predefined seed genes.

We ran SSA-ME on the Pancan-no-skin-melanoma-lymph tumor cohort [syn8494939] using ReactomeFI 2015 PPI network [syn26656494] as the reference network after removal of edges with confidence scores below 0.75. Note that, unlike [13] we used gene-level scores instead of mutually exclusive mutations between samples to optimize subnetwork selection. Gene level scores were derived from the integrated driver score p-values [syn8494939] as described in the next paragraph.

We considered the integrated driver score p-values for respectively CDS, core promoter, 5' UTR, and 3' UTR, and enhancer elements. If genes were annotated with multiple integrated driver scores in the same element, the smallest value was used. We used enhancer elements linked to 5 or fewer genes, selecting the minimum integrated driver score p-value across its linked enhancers for each gene. We used the minimum between 5'UTR and core promoter for

each gene, as these elements overlap. The Fisher method was used to combine coding and non-coding (core promoter/5'UTR, 3'UTR and enhancer) integrated driver score p-values. The seed genes used by SSA.ME correspond to all genes with combined p-values below 0.25. In each iteration of the algorithm, genes in the close neighbourhood of a seed gene are selected into a small subnetwork with a chance proportional to their gene scores (which are chosen to be uniformly distributed in the first iteration). These small subnetworks are subsequently assigned a score equal to the cumulative signals of the genes in each small subnetwork, i.e. the sum of the –log (combined p-value) of the genes in the selected small subnetwork. Individual gene scores are updated proportional to the scores of the selected small subnetworks to which they belonged as described in [13] . Updating the gene scores modifies the likelihood with which each gene will be selected in subsequent iteration steps into a small subnetwork. The iterative process continues until the method converges to a solution or until a maximum number of iterations is reached. Finally, genes are prioritized individually, reflecting their likelihood to belong to a high scoring subnetwork.

To calculate the significance of the results, we ran small subnetwork analysis 10.000 times with randomized inputs for the integrated driver score p-values of respectively the coding, non-coding and non-coding value added procedures. Randomization of integrated driver score p-values was restricted to genes in the interaction network. The driver p-values of non-coding elements of the same gene are randomized together. The resulting p-value for each gene score obtained after permutation analysis expresses how likely small subnetworks with similar or superior scores were detected using randomized data. As defined above in the non-coding value added procedure, we performed an additional permutation analysis (1.000 permutations) in which only the scores of the non-coding elements were permuted while keeping the integrated driver scores p-values of the coding elements unchanged.

For each set (coding, non-coding, and combined coding and non-coding) of gene scores, SSA-ME defines a *P*-value for each gene. We reported genes with FDR < 0.1.

We also showed that SSA-ME is well calibrated. Figure S19 compares the *P*-values generated by SSA-ME using randomized inputs against the expected uniform distribution *U*(0, 1). Table S31 shows the Kolmogorov-Smirnov test *p*-value vs. uniform distribution.

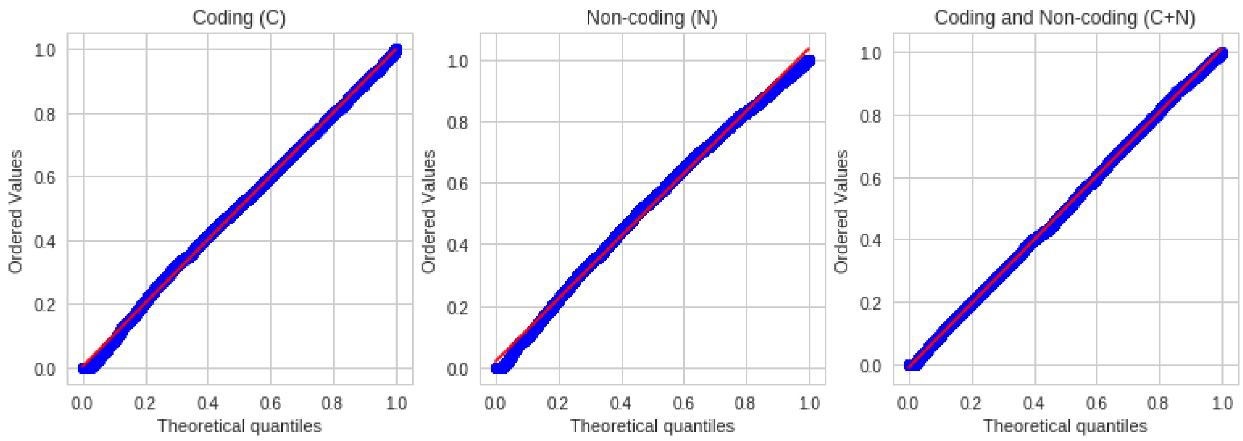

Figure S19: SSA-ME Q-Q plots of empirical *P*-value distribution on randomized data vs. the uniform distribution.

	Random vs Uniform KS p-value	Real vs Uniform KS p-value
Coding (GS-C)	0.734845	0.0
Non-coding (GS-N)	0.093552	0.0
Combined coding-and-non-coding (GS-CN)	0.782797	0.0

Table S31: Kolmogorov-Smirnov test of real and random data for SSA-ME analysis vs. the uniform distribution.

Method results

Each method reports four sets of results, which are available on Synapse (syn21413360):

1. Coding results obtained on the coding data (GS-C);
2. Non-coding results obtained on the non-coding data (GS-N);
3. Combined coding-and-non-coding results on the combined coding-and-non-coding (GS-CN) data; and
4. Union of non-coding results and non-coding value-added (NCVA) results, which are a subset of the method's combined coding-and-non-coding results.

Table S32 shows the number of genes in the results for each method. Note that CanIsoNet does not perform the NCVA procedure, so GS-N results are identical to its GS-N results without NCVA results. Note, too, that Hierarchical HotNet and the induced subnetwork analysis report results for two interaction networks, so their GS-C results are the union of its GS-C results on each of these networks.

Method	Size of coding results	Size of non-coding results	Size of combined coding-and-non-coding results	Size of union of non-coding and NCVA results
ActivePathways	261	390	427	441
CanIsoNet	668	1195	1407	1195
Hierarchical HotNet	421	554	429	640
Hypergeometric analysis	213	363	576	580
Induced subnetwork analysis	63	26	62	30
NBDI	87	247	335	261
SSA-ME	163	275	217	294

Table S32: Number of genes in the results for each method.

Method results comparison

We compared the results for each method to show that most methods report complementary sets of genes with overlapping predictions (Tables S19-S21). In terms of concordance for interpreting coding driver scores, the methods have minimal overlap. For example, the Hypergeometric Analysis and ActivePathways methods (Jaccard index of 0.39 for coding results; Supplementary Data S6) even though ActivePathways is based on the hypergeometric test. In contrast, the overlap of the results run with non-coding data (Table S7-S8) drop two- to four-fold. Thus, the differences in the network-based approaches are further exacerbated when considering genes affected by non-coding mutations compared to coding that may reflect the relative lack of representation of the genes affected by non-coding alterations in pathway

databases. On the other hand, the high overlap in coding is in part due to the presence of high-degree nodes corresponding to well-characterized genes that are often affected by coding mutations.

Pathway and network method consensus

Consensus procedure

We find consensus results across our pathway and network methods using the following procedure:

1. consensus of coding results across methods: pathway-implicated driver (PID-C) genes on coding data,
2. consensus of non-coding results across methods,
3. consensus of combined coding-and-non-coding results across methods: pathway-implicated driver (PID-CN) genes on combined coding-and-non-coding data, and
4. consensus of the union of non-coding and NCVA results across methods: pathway-implicated driver (PID-N) genes on non-coding data.

For each set of results, the consensus results are the set of genes identified by a majority (of at least 4 of 7) methods. Due to the modest overlap between the output of the methods (see the Method results comparison), this straightforward consensus procedure introduces minimal redundancy. The genes in the consensus results are supported by multiple methods that use multiple pathway databases and/or protein-protein interaction networks. In particular, note that all PID-N consensus genes except one (*RAB5B*) were found by at least one method that uses pathway information and at least one method that uses network information. A method may run on multiple pathway databases or networks. In this case, a method reports these results separately, and a gene is considered to be reported by a method if it is found on at least one of the pathway databases or networks that it considers.

Consensus results

The sets of consensus results is available on Synapse (syn11654843). Our analysis emphasizes the PID-C and PID-N gene sets. Table S33 shows the number of genes in each of the consensus results, Table S34 shows the genes in each of the consensus results, Figure S4 shows the overlap of these consensus results, and Figure S6 shows which PID-C and PID-N genes are reported by each of our methods.

The NCVA procedure added 31 genes to the PID-N results that are not in the consensus non-coding results.

Consensus results	Number of genes in results
PID-C	87
PID-N without NCVA	62
PID-CN	106
PID-N	93

Table S33: Size of the consensus results.

Consensus results	Genes
PID-C	*ACVR1B, ACVR2A, AJUBA, AKT1, ALB, APC, ARHGAP35, ARID1A, ARID1B, ARID2, ATM, AXIN1, B2M, BAX, BCL9, BRAF, BRCA2, BRD7, BTG2, CASP8, CBFB, CDH1, CDK12, CDKN1A, CDKN1B, CDKN2A, CREBBP, CTNNB1, DAXX, DDX3X, EGFR, EPHA2, EPHA6, FBXW7, FOXA1, GATA3, HDAC2, HLA-A, HRAS, KANSL1, KAT8, KDM5C, KDM6A, KEAP1, KMT2C, KMT2D, KRAS, MAP2K4, MAP2K7, MAP3K1, MEN1, NF1, NFE2L2, NOTCH1, NOTCH2, NRAS, PBRM1, PIK3CA, PIK3R1, PRKAR1A, PTCH1, PTEN, RAC1, RASA1, RB1, RHOA, RNF43, RPL22, RPL5, RPS6KA3, RRAS2, SF3B1, SMAD4, SMARCA4, SMARCB1, SOX9, SPOP, STAG2, STK11, TAF1, TBL1XR1, TCF7L2, TGFBR2, TP53, USP9X, VHL, YWHAE*
PID-N without NCVA	*ACTA2, ANP32A, ASCL1, BHLHE40, CALM1, CALM2, CDH2, CITED2, CNBP, CNOT2, COL1A2, CTNNB1, DTL, ESRRG, HDAC1, HES1, HNF1B, HNRNPA2B1, HNRNPA3, HNRNPAB, HNRNPH3, HNRNPK, HNRNPR, HNRNPU, HOXA10, HOXB5, HSPA5, ID2, KLF4, MEF2C, MYC, MYH10, NCKAP1, NCKAP1L, NFE2, NR2F1, PLAUR, PLCG1, PSMC5, PTBP1, PTPRK, PTPRM, RAB5B, RBMX, SF3B1, SOD1, SOX2, SPTBN1, SRSF1,*

	SRSF2, SRSF4, SRSF6, SRSF9, TCF4, TERT, TLE4, TNFRSF11B, TOB1, TP53, VAMP2, ZFP36L2, ZNF521
PID-CN	*ACTA2, ACTB, ACVR1B, ACVR2A, AKT1, ALB, APC, ARHGAP35, ARID1A, ARID1B, ARID2, ATM, ATP1B1, AXIN1, B2M, BAX, BCL9, BRAF, CALM1, CALM2, CASP8, CBFB, CBX3, CDH1, CDH2, CDKN1A, CDKN1B, CDKN2A, CITED2, CTNNB1, DAXX, DDX3X, DLX1, DTL, DUSP22, DUSP6, EGFR, EIF4A2, EPHA2, FBXW7, FOXA1, FST, GAP43, GATA3, HDAC1, HES1, HIST1H2AC, HLA-A, HNRNPA2B1, HNRNPA3, HNRNPR, HRAS, ID2, IL2, JAG1, KDM6A, KEAP1, KLF4, KMT2C, KMT2D, KRAS, MAP2K4, MAP2K7, MAP3K1, MEN1, MIB1, MYC, NF1, NFE2L2, NOTCH1, NRAS, NTRK2, PBRM1, PIK3CA, PIK3R1, PLCG1, PTCH1, PTEN, RASA1, RB1, RHOA, RND3, RNF43, RPL22, RPL5, RPS6KA3, SF3B1, SMAD2, SMAD4, SMARCA4, SOX9, SPOP, SRSF3, SRSF9, TCF7L2, TERT, TGFBR2, TNIK, TOB1, TP53, TSC1, USP9X, VHL, YWHAE, ZFP36L2, ZNF595*
PID-N	*ACTA2, ACTB, ANP32A, ASCL1, ATP1B1, BAZ1A, BHLHE40, CALM1, CALM2, CBX3, CDH2, CITED2, CNBP, CNOT2, COL1A2, COL3A1, CTNNB1, DDX3X, DTL, DUSP22, DUSP6, EIF4A2, ESRRG, FST, GAP43, HDAC1, HES1, HIST1H2AC, HIST1H2BO, HIST3H3, HNF1A, HNF1B, HNRNPA2B1, HNRNPA3, HNRNPAB, HNRNPH3, HNRNPK, HNRNPR, HNRNPU, HOXA10, HOXB5, HSPA5, ID2, JAG1, KLF4, LAMB1, MEF2C, MIB1, MYC, MYH10, NCKAP1, NCKAP1L, NFE2, NR2F1, NTRK2, PCDH9, PCF11, PLAUR, PLCG1, PPA1, PSMC5, PTBP1, PTPRC, PTPRK, PTPRM, PYGL, RAB5B, RBMX, RELN, SF3B1, SOD1, SOX2, SOX4, SPTBN1, SRSF1, SRSF2, SRSF4, SRSF6, SRSF9, SUFU, TCF4, TERT, TGFBR2, TLE4, TNFRSF11B, TOB1, TP53, TRIB1, VAMP2, VEGFC, ZFP36L2, ZNF521, ZNF595*

Table S34: Genes in the consensus results.

Moreover, Tables S23 and S24 further summarize the PID-C and PID-N genes, including the number of methods that identify each gene and, for each cohort, the number of samples with mutations in each coding or non-coding element.

In the Pancan-no-skin-melanoma-lymph cohort without hypermutated samples (see above data section), the 87 PID-C genes have coding mutations in 1,659 samples and the 93 PID-N genes have non-coding (promoter, 5' UTR, 3' UTR) mutations 1,105 samples, where non-coding mutations in PID-N genes are present in 151 samples (9.1%) without coding mutations in PID-C genes, constituting 14% of the 1,105 samples with non-coding mutations in PID-N genes.

Mutational signatures of PID genes

The somatic landscape of mutations in cancer is shaped by mutational processes of aging, carcinogens and deficiencies in endogenous DNA repair pathways and manifested in uneven distributions of single nucleotide variants in different nucleotide contexts[14]. To exclude the possibility that mutations in our non-coding consensus are primarily driven by carcinogens and endogeneous processes rather than positive selection indicating candidate driver mechanisms, we investigated the distribution of associated mutation signatures relative to exonic mutations of consensus coding drivers as positive controls, and coding and non-coding mutations of all genes as negative controls. Encouragingly, we found that non-coding mutations in consensus genes were enriched in aging-related mutations of signature 5 (permutation test, $P<10^{-5}$), similarly to exonic mutations in known driver genes. However, mutations of the other aging-related signature 1 were depleted among consensus non-coding genes. We also observed a higher frequency of SNVs of signature 8 (P=0.0017) of unknown mechanism enriched in breast cancer and medulloblastoma[15], signature 40 associated with kidney cancers (P=0.0012), as well as signature 17b of unknown mechanism enriched in multiple cancer types (P=0.0018). The enrichment of aging-associated signatures among our consensus non-coding genes suggests that our results include non-coding driver mutations. The lack of enrichment of major signatures associated with carcinogens and endogenous DNA damage pathways suggests that our non-coding consensus genes are not biased towards the bulk of passenger mutations generated by such signatures.

We assigned each non-coding mutation to the most likely mutational signature developed in the PCAWG project [16]. We thus evaluated the proportion of each mutational signature in non-coding consensus genes. As controls, we sampled two sets of mutations from the same subset cancer samples: protein-coding mutations of consensus coding genes as positive controls, and non-coding mutations of all promoters, enhancers and UTRs as negative controls. We computed enrichment of signatures in non-coding consensus genes relative to signatures in non-coding mutations in all genes using a permutation test. The permutation test generated 100,000 sets of

random mutations and measured the proportion of sets where the random mutations matched or exceeded the number of mutations with a particular signature that was observed in non-coding consensus genes. Random mutations were drawn from the subset of tumor samples captured by mutations non-coding consensus genes and mutation counts or random mutations were matched by relative contributions of different tumor types in captured by mutations non-coding consensus genes.

Validation and annotation of consensus results

This section describes multiple validation and annotation approaches for the consensus results.

Network neighborhood score comparison

For each PID gene, we considered the contribution of the gene's score to the score of its network neighborhood, showing that the scores of PID genes contribute significantly to their detection by pathway and network methods.

For each PID gene *g*, we used Fisher's method to combine the gene-level driver score *P*-values of the first-order network neighbors of *g* both with and without the score of *g* itself. In particular, for gene *g*, let *p*(*g*) be the driver score *P*-value for *g* and *N*(*g*) be the network neighborhood of *g*. Then

$$p_{N(g)}^{\text{without}} = \text{fisher}(p(v) : v \in N(g))$$

is the network score for the network neighborhood of *g* without *g* itself and

$$p_{N(g)}^{\text{with}} = \text{fisher}(p(v) : v \in N(g) \cup \{g\})$$

is the network score for the network neighborhood of *g* with *g*.

If the network neighborhood of *g* has a smaller *P*-value with *g* than without *g*, i.e., $p_{N(g)}^{\text{with}} < p_{N(g)}^{\text{without}}$, then gene *g* improves the score of the network neighborhood, suggesting that the score of gene *g* may provide more evidence for its detection by pathway and network methods than the scores of its network neighbors. Alternatively, if the network neighborhood of *g* has a larger *P*-value with *g* than without *g*, i.e., $p_{N(g)}^{\text{with}} > p_{N(g)}^{\text{without}}$, then gene *g* worsens the score of

the network neighborhood, suggesting that the score of gene *g* may provide less evidence for its detection by pathway and network methods than the scores of its network neighbors.

Using the BioGRID interaction network, we performed this test for every PID-C gene with GS-C gene-level scores and every PID-N gene with GS-N gene-level scores. Each PID-C gene (87/87) improved the score of its network neighborhood (Supplementary Data S9). By sampling 87 genes uniformly at random from the set of network genes with scores, we found that only 19.7 genes were expected to improve their network neighborhood scores by chance ($P < 10^{-6}$). Similarly, each PID-N gene except for *HIST1H2BO* (92/93 genes) improved the score of its network neighborhood (Supplementary Data S10). By sampling 93 genes uniformly at random from the set of network genes with scores, we found that only 28.5 genes were expected to improve their network neighborhood scores by chance ($P < 10^{-6}$).

While this analysis supports the claim that mutations in PID genes make significant contributions to their discovery by pathway and network methods, none of our methods use the approach in this analysis to nominate potential cancer genes. In particular, the values of $p_{N(g)}^{\text{without}}$ and $p_{N(g)}^{\text{with}}$ for gene *g* are not used by our methods.

Expression analysis

We use expression data to validate our results. In particular, we use the PCAWG-3 gene expression data (syn5553991), which is averaged from TopHat2 and STAR-based alignments, with FPKM-UQ normalization. Tissue type and copy number aberrations are known to be covariates for gene expression. We use the following procedures to control for the influence of tissue type:

1. Individual cohorts:
   For each cohort, we consider the set of samples with expression data, and we partition these samples into two sets: the set *A* of samples with a particular mutation and the set *B* of samples without this mutation. We perform the Wilcoxon rank-sum test on the FPKM-UQ expression values of a particular gene in sets *A* and *B* to derive a *P*-value *P* for the effect of the mutation in the cohort on the expression of the gene within the cohort.

   When evaluating expression changes on individual tissue types, we only considered

cases with at least 3 mutations in the cohort because significant expression changes are not possible by the rank-sum test with few mutations in small cohorts. This restriction reduced the number of tests. When evaluating expression across tissue types (Fisher's method), we considered all cases with at least 1 mutation.

For example, there are 11 Thy-AdenoCA patients (set *A*) with TERT promoter mutations and 36 Thy-AdenoCA patients (set *B*) with TERT promoter mutations. Thy-AdenoCA patients with TERT promoter mutations have significantly higher TERT expression (rank-sum $P = 7.1 \times 10^{-7}$) than Thy-AdenoCA patients without TERT promoter mutations.

2. Multiple cohorts:
   First, for each cohort *c*, we consider the set of samples with expression data, and we partition the samples into two sets: the set A_c of samples with a particular mutation and the set B_c of samples without this mutation. We perform the Wilcoxon rank-sum test on the expression values from A_c and B_c to derive a *P*-value P_c for the effect of the mutation on the expression of the gene in cohort *c*. We use Fisher's method to combine the rank-sum *P*-values across all cohorts to derive a *P*-value *P* for the effect of the mutation on the expression of the gene across all cohorts with mutations.

For the PID-C genes, we evaluated expression changes for patients with coding mutations. A total of 34 PID-C genes have statistically significant (FDR < 0.3) expression changes *in cis*, and a total of 5 consensus non-coding genes have statistically significant (FDR < 0.3) expression changes *in cis*. Table S35 provides these genes.

Consensus results	Genes with *in cis* expression changes
PID-C	*ACVR1B, ALB, APC, ARID1A, ARID1B, ATM, AXIN1, BRCA2, BRD7, CBFB, CDH1, CDKN2A, CTNNB1, EGFR, EPHA6, FOXA1, GATA3, HRAS, KDM5C, KDM6A, KMT2C, KRAS, MAP3K1, MEN1, NF1, NOTCH1, PIK3CA, RB1, SMAD4, SMARCA4, STAG2, STK11, TP53, VHL*
PID-N	*DUSP22, TCF4, TERT, TLE4, TP53*

Table S35: PID-C and PID-N genes with statistically significant (FDR < 0.3) *in cis* expression changes.

Tables S11-S18 summarizes the above expression results.

Network annotations

To evaluate interactions between consensus genes, we use a restrictive degree-weighted permutation scheme that compares the observed graph against random graphs sampled uniformly from the set of graphs with the same degree sequence.

We use network interactions and pathway annotations to provide biological context for our consensus results. Both coding and non-coding mutations cluster on the interaction networks (Supplementary Figure S2), where 46 PID-C genes form a connected component in the BioGRID high-confidence interaction network (interaction permutation $P = 9 \times 10^{-4}$ while conditioning on degree sequence, expected 30.6 genes) and 16 PID-N genes form a connected component (permutation $P = 6.1 \times 10^{-2}$, expected 10.2 genes). Moreover, for the union of the PID-C and PID-N genes, 73 genes form a connected component (permutation $P = 2.2 \times 10^{-3}$, expected 56.62 genes). Interactions of well-known cancer genes with candidate drivers suggest potential relationships.

A number (64) of BioGRID high-confidence interactions between PID-C genes suggest that some biological processes may be predominantly targeted by coding driver mutations (interaction permutation $P < 10^{-4}$ while conditioning on degree sequence; 40.0 interactions expected), and a number (18) of interactions between PID-N genes suggest that some biological processes may be predominantly targeted by non-coding driver mutations (permutation $P = 6.8 \times 10^{-2}$; 12.5 expected). However, a large number (67) of interactions between PID-C and PID-N genes suggest that coding and non-coding mutations may target some of the same pathways (permutation $P = 6 \times 10^{-4}$; 45.4 expected), performing complementary roles in disrupting these pathways.

Pathway annotations

We perform a pathway enrichment analysis of our consensus results to add biological context for our consensus coding and consensus non-coding results. Since each of our methods use either pathway databases or networks as prior knowledge, we expect to find pathway enrichment in our results. Using g:Profiler, we perform the hypergeometric test to evaluate the overlap between our consensus coding, consensus non-coding, and the union of the consensus coding and non-coding results with each of 12,061 gene sets representing GO biological processes and Reactome pathways (syn3164548). We use the Benjamini-Hochberg correction[17] to control the FDR of the results. Table S19 describes enriched pathways for PID-C genes, Table S20 describes enriched pathways for PID-N genes, and Table S21 describes enriched pathways for the union of the PID-C and PID-N genes.

Pathway enrichment analysis of consensus results

If a pathway was significantly enriched for the PID-C genes (FDR $< 10^{-6}$) and the pathway is not much more significantly enriched (multiplicative factor of 10^{-3}) for the PID-N genes or the union of the PID-C and PID-N genes, then we say that the enrichment of the pathway is primarily caused by PID-C genes, suggesting a primary role of coding mutations. Similarly, if a pathway was significantly enriched for the PID-N genes (FDR $< 10^{-6}$) and the pathway is not much more significantly enriched (factor of 10^{-3}) for the PID-C genes or the union of the PID-C and PID-N genes, then we say that the enrichment of the pathway is primarily caused by PID-N genes, suggesting a primary role of non-coding mutations. Alternatively, if a pathway was significantly enriched for the union of the PID-C and PID-N genes and the pathway is more significantly enriched (factor of 10^{-6}) than for the PID-C genes or the PID-N genes, then we say that the enrichment of the pathway has comparable contributions from both PID-C and PID-N genes, suggesting complementary roles by coding and non-coding driver mutations in disrupting these pathways.

Splicing factor mutant GSEA analysis

In order to identify expression changes in the splicing factors due to non-coding mutations we performed an analysis using a Wilcoxon rank sum test comparing samples with non-coding mutations in each of the genes to wild type samples in the same tumor type. No results were

significant after *p*-values were corrected using the Benjamini-Hochberg FDR correction. See Tables S11-S18.

GSEA Enrichment Analysis was performed with the default parameters using the same curated pathway gene lists from Seiler *et al.*[18] See Table S25 for curated gene sets. Seiler *et al.* performed the analysis using samples harboring mutations in 5 genes: (*SF3B1*, *SRSF2*, *U2AF1*, *RBM10*, and *FUBP1*), which have confirmed on-target splicing deregulation. However, we only had enough mutant samples to consider *SF3B1*, *RBM10*, and *FUBP1*. *SF3B1* mutants were restricted to missense mutations and *RBM10* and *FUBP1* to truncating mutations. Any tumor types with more than 2 mutant samples in any of the coding mutants was considered. Each tumor type for the coding genes was considered separately as was done in Seiler *et al.* In addition, we considered samples with mutations in the non-coding regions such as the promoter and UTR regions in 17 PID-N genes with splicing annotations (Figure 12C). Multiple tumor types were considered in the non-coding mutant/wild-type enrichment because we did not have enough mutant samples per tumor type; often there was only 1 per tumor type. The wild-type groups for each non-coding enrichment analysis were restricted to the tumor types of the mutant samples. Table S26 shows the number of samples in the mutant/wild-type groups for each mutated gene considered. The GSEA Normalized Enrichment Scores (NES) were clustered using hierarchical clustering of the Euclidean distance between the scores and complete linkage clustering and printed to a heatmap using the R package pheatmap (Figure 5A). NES scores were further clustered with t-Distributed Stochastic Neighbor Embedding using R Package Rtsne and plotted using ggplot2 (Figure 5B, S13).

We evaluated the robustness of these clusters both by computing silhouette scores and by bootstrapping. In the latter case, clusters were redetermined on the bootstrapped data and the Jaccard similarity coefficient was measured to reveal how often the co-clustering (or non co-clustering) of sample pairs in the bootstrap solution agreed with the original solution. In both tests, the pathway clusters P1 and P2 were highly robust with average silhouette scores of 0.48 and 0.31 (Supplementary Figure S11A) and high Jaccard similarity (Supplementary Figures S11B). On the other hand, mutation element clusters C1 and C3 were found to be highly robust (average silhouette scores 0.28 and 0.35; Supplementary Figure S11C), while cluster C2 had nearly half of its samples cluster with the other two clusters (average silhouette score 0.03; Supplementary Figure S11D).

References

1. Paull, E. O. *et al.* Discovering causal pathways linking genomic events to transcriptional states using Tied Diffusion Through Interacting Events (TieDIE). *Bioinformatics* **29**, 2757–2764 (2013).
2. Leiserson, M. D. M. *et al.* Pan-cancer network analysis identifies combinations of rare somatic mutations across pathways and protein complexes. *Nat. Genet.* **47**, 106–114 (2015).
3. Vogelstein, B. *et al.* Cancer genome landscapes. *Science* **339**, 1546–1558 (2013).
4. Stark, C. *et al.* BioGRID: a general repository for interaction datasets. *Nucleic Acids Res.* **34**, D535–9 (2006).
5. Brown, M. B. 400: A Method for Combining Non-Independent, One-Sided Tests of Significance. *Biometrics* **31**, 987–992 (1975).
6. Matthew A. Reyna, Mark D.M. Leiserson, Benjamin J. Raphael. Identifying hierarchies of altered subnetworks. *Bioinformatics* (2018).
7. Fabregat, A. *et al.* The Reactome pathway Knowledgebase. *Nucleic Acids Res.* **44**, D481–7 (2016).
8. Razick, S., Magklaras, G. & Donaldson, I. M. iRefIndex: a consolidated protein interaction database with provenance. *BMC Bioinformatics* **9**, 405 (2008).
9. Kanehisa, M. & Goto, S. KEGG: kyoto encyclopedia of genes and genomes. *Nucleic Acids Res.* **28**, 27–30 (2000).
10. Verbeke, L. P. C. *et al.* Pathway Relevance Ranking for Tumor Samples through Network-Based Data Integration. *PLoS One* **10**, e0133503 (2015).
11. Fabregat, A. *et al.* Reactome graph database: Efficient access to complex pathway data. *PLoS Comput. Biol.* **14**, e1005968 (2018).
12. Fouss, F., Francoisse, K., Yen, L., Pirotte, A. & Saerens, M. An experimental investigation of kernels on graphs for collaborative recommendation and semisupervised classification. *Neural Netw.* **31**, 53–72 (2012).
13. Pulido-Tamayo, S., Weytjens, B., De Maeyer, D. & Marchal, K. SSA-ME Detection of cancer driver genes using mutual exclusivity by small subnetwork analysis. *Sci. Rep.* **6**, 36257 (2016).
14. Alexandrov, L. B. & Stratton, M. R. Mutational signatures: the patterns of somatic mutations hidden in cancer genomes. *Curr. Opin. Genet. Dev.* **24**, 52–60 (2014).
15. Alexandrov, L. B. *et al.* Clock-like mutational processes in human somatic cells. *Nat. Genet.*

47, 1402–1407 (2015).

16. Alexandrov, L. *et al.* The Repertoire of Mutational Signatures in Human Cancer. *bioRxiv* 322859 (2018). doi:10.1101/322859
17. Benjamini, Y. & Hochberg, Y. Controlling the False Discovery Rate: A Practical and Powerful Approach to Multiple Testing. *Journal of the Royal Statistical Society: Series B (Methodological)* **57**, 289–300 (1995).
18. Seiler, M. *et al.* Somatic Mutational Landscape of Splicing Factor Genes and Their Functional Consequences across 33 Cancer Types. *Cell Rep.* **23**, 282–296.e4 (2018).

The ICGC/TCGA Pan-Cancer Analysis of Whole Genomes Consortium (PCAWG)

Steering committee

Peter J Campbell#[1,2], **Gad Getz#**[3,4,5,6], **Jan O Korbel#**[7,8], **Lincoln D Stein#**[9,10] and **Joshua M Stuart#**[11]

Executive committee

Sultan T Al-Sedairy[12], Axel Aretz[13], Cindy Bell[14], Miguel Betancourt[15], Christiane Buchholz[16], Fabien Calvo[17], Christine Chomienne[18], Michael Dunn[19], Stuart Edmonds[20], Eric Green[21], Shailja Gupta[22], Carolyn M Hutter[21], Karine Jegalian[23], Jennifer L Jennings[24,25], Nic Jones[26], Hyung-Lae Kim[27], Youyong Lu[28,29,30], Hitoshi Nakagama[31], Gerd Nettekoven[32], Laura Planko[32], David Scott[26], Tatsuhiro Shibata[33,34], Kiyo Shimizu[35], Lincoln D Stein[9,10], Michael Rudolf Stratton[2], Takashi Yugawa[35], Giampaolo Tortora[36,37], K VijayRaghavan[22], Huanming Yang[38] and Jean C Zenklusen[39]

Ethics and legal working group

Don Chalmers#[40], Yann Joly[41], **Bartha M Knoppers#**[41], Fruzsina Molnár-Gábor[42], Mark Phillips[41], Adrian Thorogood[41] and David Townend[43]

Technical working group

Brice Aminou[44], Javier Bartolome[45], Keith A Boroevich[46,47], Rich Boyce[7], Alvis Brazma[7], Angela N Brooks[3,48,49], Alex Buchanan[50], Ivo Buchhalter[51,52,53], Adam P Butler[2], Niall J Byrne[44], Andy Cafferkey[7], Peter J Campbell[1,2], Zhaohong Chen[54], Sunghoon Cho[55], Wan Choi[56], Peter Clapham[2], Brandi N Davis-Dusenbery[57], Francisco M De La Vega[58,59,60], Jonas Demeulemeester[61,62], Michelle T Dow[54], Lewis Jonathan Dursi[9,63], Juergen Eils[64,65], Roland Eils[51,53,64,65], Kyle Ellrott[50], Claudiu Farcas[54], Nodirjon Fayzullaev[44], Vincent Ferretti[44,66], Paul Flicek[7], Nuno A Fonseca[7,67], Josep Ll Gelpi[45,68], Gad Getz[3,4,5,6], Robert L Grossman[69], Olivier Harismendy[70], Allison P Heath[71], Michael C Heinold[51,53], Julian M Hess[3,72], Oliver Hofmann[73], Jongwhi H Hong[74], Thomas J Hudson[75,76], Barbara Hutter[77,78,79], Carolyn M Hutter[21], Daniel Hübschmann[53,64,80,81,82], Seiya Imoto[83,83], Sinisa Ivkovic[84], Seung-Hyup Jeon[56], Wei Jiao[9], Jongsun Jung[85], Rolf Kabbe[51], Andre Kahles[86,87,88,89,90], Jules NA Kerssemakers[51], Hyung-Lae Kim[27], Hyunghwan Kim[56], Jihoon Kim[91], Youngwook Kim[92,93], Kortine Kleinheinz[51,53], Jan O Korbel[7,8], Michael Koscher[94], Antonios Koures[54], Milena Kovacevic[84], Chris Lawerenz[65], Ignaty Leshchiner[3], Jia Liu[95], Dimitri Livitz[3], George L Mihaiescu[44], Sanja Mijalkovic[84], Ana Mijalkovic Mijalkovic-Lazic[84], Satoru Miyano[83], Naoki Miyoshi[83], Hardeep K Nahal-Bose[44], Hidewaki Nakagawa[47], Mia Nastic[84], Steven J Newhouse[7], Jonathan Nicholson[2], **Brian D O'Connor#**[44,49], David Ocana[7], Kazuhiro Ohi[83], Lucila Ohno-Machado[54], Larsson

Omberg[96], BF Francis Ouellette[97,98], Nagarajan Paramasivam[51,78], Marc D Perry[44,99], Todd D Pihl[100], Manuel Prinz[51], Montserrat Puiggròs[45], Petar Radovic[84], Keiran M Raine[2], Esther Rheinbay[3,6,101], Mara Rosenberg[3,101], Romina Royo[45], Gunnar Rätsch[86,87,88,89,102,103], Gordon Saksena[3], Matthias Schlesner[51,104], Solomon I Shorser[9], Charles Short[7], Heidi J Sofia[21], Jonathan Spring[69], **Lincoln D Stein#**[9,10], Adam J Struck[50], Grace Tiao[3], Nebojsa Tijanic[84], David Torrents[45,105], Peter Van Loo[61,62], Miguel Vazquez[45,106], David Vicente[45], Jeremiah A Wala[3,6,48], Zhining Wang[39], Sebastian M Waszak[8], Joachim Weischenfeldt[8,107,108], Johannes Werner[51,109], Ashley Williams[54], Youngchoon Woo[56], Adam J Wright[9], Qian Xiang[110], **Sergei Yakneen#**[8], Liming Yang[39], Denis Yuen[9], **Christina K Yung#**[44] and **Junjun Zhang#**[44]

Annotations working group

Angela N Brooks[3,48,49], Ivo Buchhalter[51,52,53], Peter J Campbell[1,2], Priyanka Dhingra[111,112], Lars Feuerbach[113], Mark Gerstein[114,115,116], Gad Getz[3,4,5,6], Mark P Hamilton[117], Henrik Hornshøj[118], Todd A Johnson[46], Andre Kahles[86,87,88,89,90], Abdullah Kahraman[119,120,121], Manolis Kellis[3,122], **Ekta Khurana#**[111,112,123,124], Jan O Korbel[7,8], Morten Muhlig Nielsen[118], Jakob Skou Pedersen[118,125], Paz Polak[3,4,6], Jüri Reimand[9,126], Esther Rheinbay[3,6,101], Nicola D Roberts[2], Gunnar Rätsch[86,87,88,89,102,103], Richard Sallari[3], Nasa Sinnott-Armstrong[3,60], Alfonso Valencia[45,105], Miguel Vazquez[45,106], Sebastian M Waszak[8], Joachim Weischenfeldt[8,107,108] and Christian von Mering[121,127]

Quality control working group

Sergi Beltran[128,129], Ivo Buchhalter[51,52,53], Peter J Campbell[1,2], Roland Eils[51,53,64,65], Daniela S Gerhard[130], Gad Getz[3,4,5,6], **Ivo G Gut#**[128,129], Marta Gut[128,129], Barbara Hutter[77,78,79], Daniel Hübschmann[53,64,80,81,82], Kortine Kleinheinz[51,53], Jan O Korbel[7,8], Dimitri Livitz[3], Marc D Perry[44,99], Keiran M Raine[2], Esther Rheinbay[3,6,101], Mara Rosenberg[3,101], Gordon Saksena[3], Matthias Schlesner[51,104], Miranda D Stobbe[128,129], Jean-Rémi Trotta[128], Johannes Werner[51,109] and Justin P Whalley[128]

Novel somatic mutation calling methods

Matthew H Bailey[131,132], Beifang Niu[133], Matthias Bieg[78,134], Paul C Boutros[9,126,135,136], Ivo Buchhalter[51,52,53], Adam P Butler[2], Ken Chen[137], Zechen Chong[138], **Li Ding#**[131,132,139], Oliver Drechsel[129,140], Lewis Jonathan Dursi[9,63], Roland Eils[51,53,64,65], Kyle Ellrott[50], Shadrielle MG Espiritu[9], Yu Fan[141], Robert S Fulton[131,132,139], Shengjie Gao[38], Josep Ll Gelpi[45,68], Mark Gerstein[114,115,116], Gad Getz[3,4,5,6], Santiago Gonzalez[7,8], Ivo G Gut[128,129], Faraz Hach[142,143], Michael C Heinold[51,53], Julian M Hess[3,72], Jonathan Hinton[2], Taobo Hu[144], Vincent Huang[9], Yi Huang[145,146], Barbara Hutter[77,78,79], David R Jones[2], Jongsun Jung[85], Natalie Jäger[51], Hyung-Lae Kim[27], Kortine Kleinheinz[51,53], Sushant Kumar[115,116], Yogesh Kumar[144], Christopher M Lalansingh[9], Ignaty Leshchiner[3], Ivica Letunic[147], Dimitri Livitz[3], Eric Z Ma[144], Yosef E Maruvka[3,72,101], R Jay Mashl[132,148], Michael D McLellan[131,132,139], Andrew Menzies[2], Ana Milovanovic[45], Morten Muhlig Nielsen[118], Stephan Ossowski[129,140,149], Nagarajan Paramasivam[51,78], Jakob Skou Pedersen[118,125], Marc D Perry[44,99], Montserrat

Puiggròs[45], Keiran M Raine[2], Esther Rheinbay[3,6,101], Romina Royo[45], S Cenk Sahinalp[143,150,151], Gordon Saksena[3], Iman Sarrafi[143,151], Matthias Schlesner[51,104], **Jared T Simpson#**[9,152], Lucy Stebbings[2], Chip Stewart[3], Miranda D Stobbe[128,129], Jon W Teague[2], Grace Tiao[3], David Torrents[45,105], Jeremiah A Wala[3,6,48], Jiayin Wang[132,146,153], Wenyi Wang[141], Sebastian M Waszak[8], Joachim Weischenfeldt[8,107,108], Michael C Wendl[132,154,155], Johannes Werner[51,109], David A Wheeler[156,157], Zhenggang Wu[144], Hong Xue[144], Sergei Yakneen[8], Takafumi N Yamaguchi[9], Kai Ye[153,158], Venkata D Yellapantula[159,160], Christina K Yung[44] and Junjun Zhang[44]

Drivers and functional interpretation

Federico Abascal[2], Samirkumar B Amin[161,162,163], Gary D Bader[10], Pratiti Bandopadhayay[3,164,165], Jonathan Barenboim[9], Rameen Beroukhim[3,6,166], Johanna Bertl[118,167], Keith A Boroevich[46,47], Søren Brunak[168,169], Peter J Campbell[1,2], Joana Carlevaro-Fita[170,171,172], Dimple Chakravarty[173,174], Calvin Wing Yiu Chan[51,175], Ken Chen[137], Jung Kyoon Choi[176], Jordi Deu-Pons[177,178], Priyanka Dhingra[111,112], Klev Diamanti[179], Lars Feuerbach[113], J Lynn Fink[45,180], Nuno A Fonseca[7,67], Joan Frigola[177], Carlo Gambacorti-Passerini[181], Dale W Garsed[182,183], **Mark Gerstein#**[114,115,116], **Gad Getz#**[3,4,5,6], Qianyun Guo[125], Ivo G Gut[128,129], David Haan[11], Mark P Hamilton[117], Nicholas J Haradhvala[3,101], Arif O Harmanci[116,184], Mohamed Helmy[185], Carl Herrmann[51,53,186], Julian M Hess[3,72], Asger Hobolth[125,167], Ermin Hodzic[151], Chen Hong[113,175], Henrik Hornshøj[118], Keren Isaev[9,126], Jose MG Izarzugaza[168], Rory Johnson[171,187], Todd A Johnson[46], Malene Juul[118], Randi Istrup Juul[118], Andre Kahles[86,87,88,89,90], Abdullah Kahraman[119,120,121], Manolis Kellis[3,122], Ekta Khurana[111,112,123,124], Jaegil Kim[3], Jong K Kim[188], Youngwook Kim[92,93], Jan Komorowski[179,189], Jan O Korbel[7,8], Sushant Kumar[115,116], Andrés Lanzós[171,172,187], Erik Larsson[86], **Michael S Lawrence#**[3,46,101], Donghoon Lee[116], Kjong-Van Lehmann[86,88,89,90,190], Shantao Li[116], Xiaotong Li[116], Ziao Lin[3,191], Eric Minwei Liu[111,112,192], Lucas Lochovsky[115,116,163], Shaoke Lou[115,116], Tobias Madsen[118], Kathleen Marchal[193,194], Iñigo Martincorena[2], Alexander Martinez-Fundichely[111,112,124], Yosef E Maruvka[3,72,101], Patrick D McGillivray[115], William Meyerson[116,195], Ferran Muiños[178,196], Loris Mularoni[178,196], Hidewaki Nakagawa[47], Morten Muhlig Nielsen[118], Marta Paczkowska[9], Keunchil Park[197,198], Kiejung Park[199], **Jakob Skou Pedersen#**[118,125], Tirso Pons[200], Sergio Pulido-Tamayo[193,194], **Benjamin J Raphael#**[201], Jüri Reimand[9,126], Iker Reyes-Salazar[196], Matthew A Reyna[201], Esther Rheinbay[3,6,101], Mark A Rubin[187,202,203,204,205], Carlota Rubio-Perez[178,196,206], S Cenk Sahinalp[143,150,151], Gordon Saksena[3], Leonidas Salichos[115,116], Chris Sander[48,86,207,208], Steven E Schumacher[3,209], Mark Shackleton[182,183], Ofer Shapira[3,48], Ciyue Shen[208,210], Raunak Shrestha[143], Shimin Shuai[9,10], Nikos Sidiropoulos[108], Lina Sieverling[113,175], Nasa Sinnott-Armstrong[3,60], Lincoln D Stein[9,10], **Joshua M Stuart#**[11], David Tamborero[178,196], Grace Tiao[3], Tatsuhiko Tsunoda[46,211,212,213], Husen M Umer[179,214], Liis Uusküla-Reimand[215,216], Alfonso Valencia[45,105], Miguel Vazquez[45,106], Lieven PC Verbeke[194,217], Claes Wadelius[218], Lina Wadi[9], Jiayin Wang[132,146,153], Jonathan Warrell[115,116], Sebastian M Waszak[8], Joachim Weischenfeldt[8,107,108], **David A Wheeler#**[156,157], Guanming Wu[219], Jun Yu[220], Jing Zhang[116], Xuanping Zhang[146,221], Yan Zhang[116,222,223], Zhongming Zhao[224], Lihua Zou[225] and Christian von Mering[121,127]

Integration of transcriptome and genome

Samirkumar B Amin[161,162,163], Philip Awadalla[9,10], Peter J Bailey[226], **Alvis Brazma#**[7], **Angela N Brooks#**[3,48,49], Claudia Calabrese[7,8], Aurélien Chateigner[44], Isidro Cortés-Ciriano[227,228,229], Brian Craft[230], David Craft[3,231], Chad J Creighton[232], Natalie R Davidson[86,88,89,103,190], Deniz Demircioğlu[233,234], Serap Erkek[8], Nuno A Fonseca[7,67], Milana Frenkel-Morgenstern[235], Mary J Goldman[230], Liliana Greger[7], Jonathan Göke[233,236], Yao He[237], Katherine A Hoadley[238,239], Yong Hou[38,240], Matthew R Huska[241], Andre Kahles[86,87,88,89,90], Ekta Khurana[111,112,123,124], Helena Kilpinen[242], Jan O Korbel[7,8], Fabien C Lamaze[9], Kjong-Van Lehmann[86,88,89,90,190], Chang Li[38,240], Siliang Li[38,240], Xiaobo Li[38,240], Xinyue Li[38], Dongbing Liu[38,240], Fenglin Liu[237,243], Xingmin Liu[38,240], Maximillian G Marin[49], Julia Markowski[241], Matthew Meyerson[3,6,48], Tannistha Nandi[244], Morten Muhlig Nielsen[118], Akinyemi I Ojesina[245,246,247], BF Francis Ouellette[97,98], Qiang Pan-Hammarström[38,248], Peter J Park[228,229], Chandra Sekhar Pedamallu[3,6,166], Jakob Skou Pedersen[118,125], Marc D Perry[44,99], **Gunnar Rätsch#**[86,87,88,89,102,103], Roland F Schwarz[7,81,241,249], Yuichi Shiraishi[83], Reiner Siebert[250,251], Cameron M Soulette[49], Stefan G Stark[89,190,252,253], Oliver Stegle[7,8,254], Hong Su[38,240], Patrick Tan[244,255,256,257], Bin Tean Teh[255,256,257,258,259], Lara Urban[7,8], Jian Wang[38], Sebastian M Waszak[8], Kui Wu[38,240], Qian Xiang[110], Heng Xiong[38,240], Sergei Yakneen[8], Huanming Yang[38], Chen Ye[38,240], Christina K Yung[44], Fan Zhang[237], Junjun Zhang[44], Xiuqing Zhang[38], Zemin Zhang[237,260], Liangtao Zheng[237], Jingchun Zhu[230] and Shida Zhu[38,240]

Integration of epigenome and genome

Hiroyuki Aburatani[261], **Benjamin P Berman#**[262,263,264], Hans Binder[265,266], **Benedikt Brors#**[79,113,267], Huy Q Dinh[262], Lars Feuerbach[113], Shengjie Gao[38], Ivo G Gut[128,129], Simon C Heath[128,129], Steve Hoffmann[265,266,268,269], Charles David Imbusch[113], Ekta Khurana[111,112,123,124], Helene Kretzmer[266,269], Peter W Laird[270], Jose I Martin-Subero[105,271], Genta Nagae[261,272], **Christoph Plass#**[273], Paz Polak[3,4,6], Hui Shen[274], Reiner Siebert[250,251], Nasa Sinnott-Armstrong[3,60], Miranda D Stobbe[128,129], Qi Wang[94], Dieter Weichenhan[273], Sergei Yakneen[8] and Wanding Zhou[274]

Patterns of structural variations, signatures, genomic correlations, retrotransposons, mobile elements

Kadir C Akdemir[137], Eva G Alvarez[275,276,277], Adrian Baez-Ortega[278], **Rameen Beroukhim#**[3,6,166], Paul C Boutros[9,126,135,136], David D L Bowtell[182,183], Benedikt Brors[79,113,267], Kathleen H Burns[279,280], John Busanovich[3,281], **Peter J Campbell#**[1,2], Kin Chan[282], Ken Chen[137], Isidro Cortés-Ciriano[227,228,229], Ana Dueso-Barroso[45], Andrew J Dunford[3], Paul A Edwards[283,284], Xavier Estivill[140,285], Dariush Etemadmoghadam[182,183], Lars Feuerbach[113], J Lynn Fink[45,180], Milana Frenkel-Morgenstern[235], Dale W Garsed[182,183], Mark Gerstein[114,115,116], Dmitry A Gordenin[286], David Haan[11], James E Haber[287], Julian M Hess[3,72], Barbara Hutter[77,78,79], Marcin Imielinski[288,289], David TW Jones[290,291], Young Seok Ju[2,176], Marat D Kazanov[292,293,294], Leszek J Klimczak[295], Youngil Koh[296,297], Jan O Korbel[7,8], Kiran Kumar[3], Eunjung Alice Lee[298], Jake June-Koo Lee[228,229], Yilong Li[2], Andy G Lynch[283,284,299], Geoff Macintyre[283], Florian Markowetz[283,284], Iñigo Martincorena[2], Alexander Martinez-Fundichely[111,112,124], Matthew Meyerson[3,6,48], Satoru Miyano[83], Hidewaki Nakagawa[47], Fabio CP Navarro[115], Stephan Ossowski[129,140,149], Peter J Park[228,229], John V Pearson[300,301], Montserrat Puiggròs[45], Karsten Rippe[81], Nicola D Roberts[2], Steven A Roberts[302], Bernardo

Rodriguez-Martin[275,276,277], Steven E Schumacher[3,209], Ralph Scully[303], Mark Shackleton[182,183], Nikos Sidiropoulos[108], Lina Sieverling[113,175], Chip Stewart[3], David Torrents[45,105], Jose MC Tubio[275,276,277], Izar Villasante[45], Nicola Waddell[300,301], Jeremiah A Wala[3,6,48], Joachim Weischenfeldt[8,107,108], Lixing Yang[304], Xiaotong Yao[288,305], Sung-Soo Yoon[297], Jorge Zamora[2,275,276,277] and Cheng-Zhong Zhang[3,6,48]

Mutation signatures and processes

Ludmil B Alexandrov[2,306], Erik N Bergstrom[307], Arnoud Boot[256,308], Paul C Boutros[9,126,135,136], Kin Chan[282], Kyle Covington[157], Akihiro Fujimoto[47], Gad Getz[3,4,5,6], Dmitry A Gordenin[286], Nicholas J Haradhvala[3,101], Mi Ni Huang[256,308], S. M. Ashiqul Islam[306], Marat D Kazanov[292,293,294], Jaegil Kim[3], Leszek J Klimczak[295], Michael S Lawrence[3,46,101], Iñigo Martincorena[2], John R McPherson[256,308], Sandro Morganella[2], Ville Mustonen[309,310,311], Hidewaki Nakagawa[47], Alvin Wei Tian Ng[312], Serena Nik-Zainal[2,313,314,315], Paz Polak[3,4,6], Stephenie D Prokopec[9], Steven A Roberts[302], **Steven G Rozen**#[256,257,308], Radhakrishnan Sabarinathan[178,196,316], Natalie Saini[286], Tatsuhiro Shibata[33,34], Yuichi Shiraishi[83], **Michael Rudolf Stratton**#[2], **Bin Tean Teh**#[255,256,257,258,259], Ignacio Vázquez-García[2,159,317,318], Yang Wu[256,308], Fouad Yousif[9] and Willie Yu[319]

Germline cancer genome

Ludmil B Alexandrov[2,306], Eva G Alvarez[275,276,277], Adrian Baez-Ortega[278], Matthew H Bailey[131,132], Mattia Bosio[45,129,140], G Steven Bova[320], Alvis Brazma[7], Alicia L Bruzos[275,276,277], Ivo Buchhalter[51,52,53], Carlos D Bustamante[59,60], Atul J Butte[321], Andy Cafferkey[7], Claudia Calabrese[7,8], Peter J Campbell[1,2], Stephen J Chanock[322], Nilanjan Chatterjee[323,324,324], Jieming Chen[116,325], Francisco M De La Vega[58,59,60], Olivier Delaneau[326,327,328], German M Demidov[129,140,149], Anthony DiBiase[329], Li Ding[131,132,139], Oliver Drechsel[129,140], Lewis Jonathan Dursi[9,63], Douglas F Easton[330,331], Serap Erkek[8], Georgia Escaramis[140,332,333], **Xavier Estivill**#[140,285], Erik Garrison[2], Mark Gerstein[114,115,116], Gad Getz[3,4,5,6], Dmitry A Gordenin[286], Nina Habermann[8], Olivier Harismendy[70], Eoghan Harrington[334], Shuto Hayashi[83], Seong Gu Heo[335], José María Heredia-Genestar[336], Aliaksei Z Holik[140], Eun Pyo Hong[335], Xing Hua[322], Kuan-lin Huang[132,337], Seiya Imoto[83,83], Sissel Juul[334], Ekta Khurana[111,112,123,124], Hyung-Lae Kim[27], Youngwook Kim[92,93], Leszek J Klimczak[295], **Jan O Korbel**#[7,8], Roelof Koster[338], Sushant Kumar[115,116], Ivica Letunic[147], Yilong Li[2], Tomas Marques-Bonet[105,128,339,340], R Jay Mashl[132,148], Simon Mayes[341], Michael D McLellan[131,132,139], Lisa Mirabello[322], Francesc Muyas[129,140,149], Hidewaki Nakagawa[47], Arcadi Navarro[105,128,339], Steven J Newhouse[7], Stephan Ossowski[129,140,149], Ji Wan Park[335], Esa Pitkänen[8], Aparna Prasad[129], Raquel Rabionet[129,140,342], Benjamin Raeder[8], Tobias Rausch[8], Steven A Roberts[302], Bernardo Rodriguez-Martin[275,276,277], Vasilisa A Rudneva[8], Gunnar Rätsch[86,87,88,89,102,103], Natalie Saini[286], Matthias Schlesner[51,104], Roland F Schwarz[7,81,241,249], Ayellet V Segre[3,3,343], Tal Shmaya[58], Suyash S Shringarpure[60], Nikos Sidiropoulos[108], Reiner Siebert[250,251], Jared T Simpson[9,152], Lei Song[322], Oliver Stegle[7,8,254], Hana Susak[129,140], Tomas J Tanskanen[344], Grace Tiao[3], Marta Tojo[277], Jose MC Tubio[275,276,277], Daniel J Turner[341], Lara Urban[7,8], Sebastian M Waszak[8], David C Wedge[2,345,346], Joachim Weischenfeldt[8,107,108], David A Wheeler[156,157], Mark H Wright[60], Dai-Ying Wu[58], Tian Xia[347], Sergei Yakneen[8], Kai Ye[153,158], Venkata D Yellapantula[159,160], Jorge Zamora[2,275,276,277] and Bin

Zhu[322]

Tumor subtypes and clinical translation

Fatima Al-Shahrour[348], Gurnit Atwal[9,10,349], Peter J Bailey[226], **Andrew V Biankin#**[226,350,351,352], Paul C Boutros[9,126,135,136], Peter J Campbell[1,2], David K Chang[226,351], Susanna L Cooke[226], Vikram Deshpande[101], Bishoy M Faltas[103], William C Faquin[101], **Levi Garraway#**[48], Gad Getz[3,4,5,6], **Sean M Grimmond#**[353], Syed Haider[9], **Katherine A Hoadley#**[238,239], Wei Jiao[9], Vera B Kaiser[354], Rosa Karlić[355], Mamoru Kato[356], Kirsten Kübler[3,6,101], Alexander J Lazar[357], Constance H Li[9,126], David N Louis[101], Adam Margolin[50], Sancha Martin[2,358], Hardeep K Nahal-Bose[44], G Petur Nielsen[101], Serena Nik-Zainal[2,313,314,315], Larsson Omberg[96], Christine P'ng[9], Marc D Perry[44,99], Paz Polak[3,4,6], Esther Rheinbay[3,6,101], Mark A Rubin[187,202,203,204,205], Colin A Semple[354], Dennis C Sgroi[101], Tatsuhiro Shibata[33,34], Reiner Siebert[250,251], Jaclyn Smith[50], **Lincoln D Stein#**[9,10], Miranda D Stobbe[128,129], Ren X Sun[9], Kevin Thai[44], Derek W Wright[359,360], Chin-Lee Wu[101], Ke Yuan[283,358,361] and Junjun Zhang[44]

Evolution and heterogeneity

David J Adams[2], Pavana Anur[362], Rameen Beroukhim[3,6,166], Paul C Boutros[9,126,135,136], David D L Bowtell[182,183], Peter J Campbell[1,2], Shaolong Cao[141], Elizabeth L Christie[182], Marek Cmero[363,364,365], Yupeng Cun[366], Kevin J Dawson[2], Jonas Demeulemeester[61,62], Stefan C Dentro[2,61,345], Amit G Deshwar[367], Nilgun Donmez[143,151], Ruben M Drews[283], Roland Eils[51,53,64,65], Yu Fan[141], Matthew W Fittall[61], Dale W Garsed[182,183], Moritz Gerstung[7,8], Gad Getz[3,4,5,6], Santiago Gonzalez[7,8], Gavin Ha[3], Kerstin Haase[61], Marcin Imielinski[288,289], Lara Jerman[8,368], Yuan Ji[369,370], Clemency Jolly[61], Kortine Kleinheinz[51,53], Juhee Lee[371], Henry Lee-Six[2], Ignaty Leshchiner[3], Dimitri Livitz[3], Geoff Macintyre[283], Salem Malikic[143,151], Florian Markowetz[283,284], Iñigo Martincorena[2], Thomas J Mitchell[2,284,372], Quaid D Morris[349,373], Ville Mustonen[309,310,311], Layla Oesper[374], Martin Peifer[366], Myron Peto[375], Benjamin J Raphael[201], Daniel Rosebrock[3], Yulia Rubanova[152,349], S Cenk Sahinalp[143,150,151], Adriana Salcedo[9], Matthias Schlesner[51,104], Steven E Schumacher[3,209], Subhajit Sengupta[376], Ruian Shi[373], Seung Jun Shin[253], **Paul T Spellman#**[377], Oliver Spiro[3], Lincoln D Stein[9,10], Maxime Tarabichi[2,61], **Peter Van Loo#**[61,62], Shankar Vembu[373,378], Ignacio Vázquez-García[2,159,317,318], Wenyi Wang[141], **David C Wedge#**[2,345,346], David A Wheeler[156,157], Jeffrey A Wintersinger[185,349,379], Tsun-Po Yang[366], Xiaotong Yao[288,305], Kaixian Yu[380], Ke Yuan[283,358,361] and Hongtu Zhu[381,382]

Exploratory: portals, visualization and software infrastructure

Fatima Al-Shahrour[348], Elisabet Barrera[7], Wojciech Bazant[7], Alvis Brazma[7], Isidro Cortés-Ciriano[227,228,229], Brian Craft[230], David Craft[3,231], Vincent Ferretti[44,66], Nuno A Fonseca[7,67], Anja Füllgrabe[7], Mary J Goldman[230], **David Haussler#**[230,383], Wolfgang Huber[8], Maria Keays[7], Alfonso Muñoz[7], Brian D O'Connor[44,49], Irene Papatheodorou[7], Robert Petryszak[7], Elena Piñeiro-Yáñez[348], Alfonso Valencia[45,105], **Miguel Vazquez#**[45,106], John N Weinstein[384,385], Qian Xiang[110], Junjun Zhang[44] and **Jingchun Zhu#**[230]

Exploratory: mitochondrial variants and HLA/immunogenicity

Peter J Campbell[1,2], Yiwen Chen[141], Chad J Creighton[232], Li Ding[131,132,139], Akihiro Fujimoto[47], Masashi Fujita[47], Gad Getz[3,4,5,6], Leng Han[221], Takanori Hasegawa[83], Shuto Hayashi[83], Seiya Imoto[83,83], Young Seok Ju[2,176], Hyung-Lae Kim[27], Youngwook Kim[92,93], Youngil Koh[296,297], Mitsuhiro Komura[83], Jun Li[141], **Han Liang#**[141], Iñigo Martincorena[2], Satoru Miyano[83], Shinichi Mizuno[386], **Hidewaki Nakagawa#**[47], Keunchil Park[197,198], Eigo Shimizu[83], Yumeng Wang[141,387], John N Weinstein[384,385], Yanxun Xu[388], Rui Yamaguchi[83], Fan Yang[373], Yang Yang[221], Christopher J Yoon[176], Sung-Soo Yoon[297], Yuan Yuan[141], Fan Zhang[237] and Zemin Zhang[237,260]

Exploratory: pathogens

Malik Alawi[389,390], Ivan Borozan[9], Daniel S Brewer[391,392], Colin S Cooper[392,393,394], Nikita Desai[44], Roland Eils[51,53,64,65], Vincent Ferretti[44,66], Adam Grundhoff[389,395], Murat Iskar[396], Kortine Kleinheinz[51,53], **Peter Lichter#**[77,396], Hidewaki Nakagawa[47], Akinyemi I Ojesina[245,246,247], Chandra Sekhar Pedamallu[3,6,166], Matthias Schlesner[51,104], Xiaoping Su[397] and Marc Zapatka[396]

Tumor Specific Providers – Australia (Ovarian cancer)

Kathryn Alsop[182,398], Australian Ovarian Cancer Study Group[182,300,399], **David D L Bowtell#**[182,183], Timothy JC Bruxner[180], Angelika N Christ[180], Elizabeth L Christie[182], Stephen M Cordner[400], Prue A Cowin[182], Ronny Drapkin[401], Dariush Etemadmoghadam[182,183], Sian Fereday[182], Dale W Garsed[182,183], Joshy George[163], Sean M Grimmond[353], Anne Hamilton[182], Oliver Holmes[300,301], Jillian A Hung[402,403], Karin S Kassahn[180,404], Stephen H Kazakoff[300,301], Catherine J Kennedy[405,406], Conrad R Leonard[300,301], Linda Mileshkin[182], David K Miller[180,351,407], Gisela Mir Arnau[182], Chris Mitchell[182], Felicity Newell[300,301], Katia Nones[300,301], Ann-Marie Patch[300,301], John V Pearson[300,301], Michael C Quinn[300,301], Mark Shackleton[182,183], Darrin F Taylor[180], Heather Thorne[182], Nadia Traficante[182], Ravikiran Vedururu[182], Nick M Waddell[301], Nicola Waddell[300,301], Paul M Waring[408], Scott Wood[300,301], Qinying Xu[300,301] and Anna deFazio[409,410,411]

Tumor Specific Providers – Australia (Pancreatic cancer)

Matthew J Anderson[180], Davide Antonello[412], Andrew P Barbour[413,414], Claudio Bassi[412], Samantha Bersani[415], **Andrew V Biankin#**[226,350,351,352], Timothy JC Bruxner[180], Ivana Cataldo[415,416], David K Chang[226,351], Lorraine A Chantrill[351,417], Yoke-Eng Chiew[409], Angela Chou[351,418], Angelika N Christ[180], Sara Cingarlini[36], Nicole Cloonan[419], Vincenzo Corbo[416,420], Maria Vittoria Davi[421], Fraser R Duthie[226,422], J Lynn Fink[45,180], Anthony J Gill[351,418], Janet S Graham[226,423], **Sean M Grimmond#**[353], Ivon Harliwong[180], Oliver Holmes[300,301], Nigel B Jamieson[226,352,424], Amber L Johns[351,407], Karin S Kassahn[180,404], Stephen H Kazakoff[300,301], James G Kench[351,418,425], Luca Landoni[412], Rita T Lawlor[416], Conrad R Leonard[300,301], Andrea Mafficini[416], Neil D Merrett[412,426], David K Miller[180,351,407], Marco

Miotto[412], Elizabeth A Musgrove[226], Adnan M Nagrial[351], Felicity Newell[300,301], Katia Nones[300,301], Karin A Oien[408,427], Marina Pajic[351], Ann-Marie Patch[300,301], John V Pearson[300,301], Mark Pinese[428], Michael C Quinn[300,301], Alan J Robertson[180], Ilse Rooman[351], Borislav C Rusev[416], Jaswinder S Samra[412,418], Maria Scardoni[415], Christopher J Scarlett[351,429], Aldo Scarpa[416], Elisabetta Sereni[412], Katarzyna O Sikora[416], Michele Simbolo[420], Morgan L Taschuk[44], Christopher W Toon[351], Giampaolo Tortora[36,37], Caterina Vicentini[416], Nick M Waddell[301], Nicola Waddell[300,301], Scott Wood[300,301], Jianmin Wu[351], Qinying Xu[300,301] and Nikolajs Zeps[430,431]

Tumor Specific Providers – Australia (Skin cancer)

Lauri A Aaltonen[432], Andreas Behren[433], Hazel Burke[434], Jonathan Cebon[433], Rebecca A Dagg[435], Ricardo De Paoli-Iseppi[436], Ken Dutton-Regester[300], Matthew A Field[437], Anna Fitzgerald[438], Sean M Grimmond[353], **Nicholas K Hayward#**[300,434], Peter Hersey[434], Oliver Holmes[300,301], Valerie Jakrot[434], Peter A Johansson[300], Hojabr Kakavand[436], Stephen H Kazakoff[300,301], Richard F Kefford[439], Loretta MS Lau[440], Conrad R Leonard[300,301], Georgina V Long[441], **Graham J Mann#**[442,443], Felicity Newell[300,301], Katia Nones[300,301], Ann-Marie Patch[300,301], John V Pearson[300,301], Hilda A Pickett[440], Antonia L Pritchard[300], Gulietta M Pupo[444], Robyn PM Saw[441], Sarah-Jane Schramm[445], **Richard A Scolyer#**[410,441,446,447], Mark Shackleton[182,183], Catherine A Shang[438], Ping Shang[441], Andrew J Spillane[441], Jonathan R Stretch[441], Varsha Tembe[445], John F Thompson[441], Ricardo E Vilain[446], Nick M Waddell[301], Nicola Waddell[300,301], James S Wilmott[441], Scott Wood[300,301], Qinying Xu[300,301] and Jean Y Yang[448]

Tumor Specific Providers – Canada (Pancreatic cancer)

John Bartlett[449,450], Prashant Bavi[451], Ivan Borozan[9], Dianne E Chadwick[452], Michelle Chan-Seng-Yue[451], Sean Cleary[451,453], Ashton A Connor[453,454], Karolina Czajka[76], Robert E Denroche[451], Neesha C Dhani[455], Jenna Eagles[76], Vincent Ferretti[44,66], Steven Gallinger[451,453,454], Robert C Grant[451,454], David Hedley[455], Michael A Hollingsworth[456], **Thomas J Hudson#**[75,76], Gun Ho Jang[451], Jeremy Johns[76], Sangeetha Kalimuthu[451], Sheng-Ben Liang[457], Ilinca Lungu[451,458], Xuemei Luo[9], Faridah Mbabaali[76], **John D McPherson#**[76,451,459], Treasa A McPherson[454], Jessica K Miller[76], Malcolm J Moore[455], Faiyaz Notta[451,460], Danielle Pasternack[76], Gloria M Petersen[461], Michael H A Roehrl[126,451,462,463,464], Michelle Sam[76], Iris Selander[454], Stefano Serra[408], Sagedeh Shahabi[457], **Lincoln D Stein#**[9,10], Morgan L Taschuk[44], Sarah P Thayer[456], Lee E Timms[76], Gavin W Wilson[9,451], Julie M Wilson[451] and Bradly G Wouters[465]

Tumor Specific Providers – Canada (Prostate cancer)

Timothy A Beck[44,466], Vinayak Bhandari[9], **Paul C Boutros#**[9,126,135,136], **Robert G Bristow#**[126,467,468,469,470], Colin C Collins[143], Shadrielle MG Espiritu[9], Neil E Fleshner[471], Natalie S Fox[9], Michael Fraser[9], Syed Haider[9], Lawrence E Heisler[472], Vincent Huang[9], Emilie Lalonde[9], Julie Livingstone[9], John D McPherson[76,451,459], Alice Meng[473], Veronica Y Sabelnykova[9], Adriana Salcedo[9], Yu-Jia Shiah[9], Theodorus Van der Kwast[474] and Takafumi N Yamaguchi[9]

Tumor Specific Providers – China (Gastric cancer)

Shuai Ding[475], Daiming Fan[476], Yong Hou[38,240], Yi Huang[145,146], Lin Li[38], Siliang Li[38,240], Dongbing Liu[38,240], Xingmin Liu[38,240], **Youyong Lu**#[28,29,30], Yongzhan Nie[476,477], Hong Su[38,240], Jian Wang[38], Kui Wu[38,240], Xiao Xiao[146], Rui Xing[29,478], **Huanming Yang**#[38], Shanlin Yang[475], Yingyan Yu[479], Xiuqing Zhang[38], Yong Zhou[38] and Shida Zhu[38,240]

Tumor Specific Providers – EU: France (Renal cancer)

Rosamonde E Banks[480], Guillaume Bourque[481,482], Alvis Brazma[7], Paul Brennan[483], **Mark Lathrop**#[482], Louis Letourneau[484], Yasser Riazalhosseini[482], Ghislaine Scelo[483], **Jörg Tost**#[485], Naveen Vasudev[486] and Juris Viksna[487]

Tumor Specific Providers – EU: United Kingdom (Breast cancer)

Sung-Min Ahn[488], Ludmil B Alexandrov[2,306], Samuel Aparicio[489], Laurent Arnould[490], MR Aure[491], Shriram G Bhosle[2], E Birney[7], Ake Borg[492], S Boyault[493], AB Brinkman[494], JE Brock[495], A Broeks[496], Adam P Butler[2], AL Børresen-Dale[491], C Caldas[497,498], Peter J Campbell[1,2], Suet-Feung Chin[497,498], Helen Davies[2,313,314], C Desmedt[499,500], L Dirix[501], S Dronov[2], Anna Ehinger[502], JE Eyfjord[503], A Fatima[209], JA Foekens[504], PA Futreal[505], Øystein Garred[506,507], Moritz Gerstung[7,8], Dilip D Giri[508], D Glodzik[2], Dorthe Grabau[509], Holmfridur Hilmarsdottir[503], GK Hooijer[510], Jocelyne Jacquemier[511], SJ Jang[512], Jon G Jonasson[503], Jos Jonkers[513], HY Kim[511], Tari A King[514,515], Stian Knappskog[2,516,516], G Kong[511], S Krishnamurthy[517], SR Lakhani[518], Anita Langerød[491], Denis Larsimont[519], HJ Lee[512], JY Lee[520], Ming Ta Michael Lee[505], Yilong Li[2], Ole Christian Lingjærde[521], Gaetan MacGrogan[522], JWM Martens[504], Sancha Martin[2,358], Iñigo Martincorena[2], Andrew Menzies[2], Sandro Morganella[2], Ville Mustonen[309,310,311], Serena Nik-Zainal[2,313,314,315], Sarah O'Meara[2], I Pauporté[18], Sarah Pinder[523], X Pivot[524], Elena Provenzano[525], CA Purdie[526], Keiran M Raine[2], M Ramakrishna[2], K Ramakrishnan[2], Jorge Reis-Filho[508], AL Richardson[209], M Ringnér[492], Javier Bartolomé Rodriguez[45], FG Rodríguez-González[169], G Romieu[527], Roberto Salgado[408], Torill Sauer[521], R Shepherd[2], AM Sieuwerts[504], PT Simpson[518], M Smid[504], C Sotiriou[54], PN Span[528], Lucy Stebbings[2], Ólafur Andri Stefánsson[529], Alasdair Stenhouse[530], **Michael Rudolf Stratton**#[2], HG Stunnenberg[240,531], Fred Sweep[532], BK Tan[533], Jon W Teague[2], Gilles Thomas[534], AM Thompson[530], S Tommasi[535], I Treilleux[536,537], Andrew Tutt[209], NT Ueno[382], S Van Laere[501], Peter Van Loo[61,62], GG Van den Eynden[501], P Vermeulen[501], Alain Viari[416], A Vincent-Salomon[531], David C Wedge[2,345,346], Bernice Huimin Wong[538], Lucy Yates[2], X Zou[2], CHM van Deurzen[539], MJ van de Vijver[408] and L van't Veer[540,541]

Tumor Specific Providers – Germany (Malignant lymphoma)

Ole Ammerpohl[542,543], Sietse Aukema[544,545], Anke K Bergmann[546], Stephan H Bernhart[265,266,269],

Hans Binder[265,266], Arndt Borkhardt[547], Christoph Borst[548], Benedikt Brors[79,113,267], Birgit Burkhardt[549], Alexander Claviez[550], Roland Eils[51,53,64,65], Maria Elisabeth Goebler[551], Andrea Haake[542], Siegfried Haas[548], Martin Hansmann[552], Jessica I Hoell[547], Steve Hoffmann[265,266,268,269], Michael Hummel[553], Daniel Hübschmann[53,64,80,81,82], Dennis Karsch[554], Wolfram Klapper[544], Kortine Kleinheinz[51,53], Michael Kneba[554], Jan O Korbel[7,8], Helene Kretzmer[266,269], Markus Kreuz[555], Dieter Kube[556], Ralf Küppers[557], Chris Lawerenz[65], Dido Lenze[553], Peter Lichter[77,396], Markus Loeffler[555], Cristina López[251,542], Luisa Mantovani-Löffler[558], Peter Möller[559], German Ott[560], Bernhard Radlwimmer[396], Julia Richter[542,544], Marius Rohde[561], Philip C Rosenstiel[562], Andreas Rosenwald[563], Markus B Schilhabel[562], Matthias Schlesner[51,104], Stefan Schreiber[564], **Reiner Siebert#**[250,251], Peter F Stadler[265,266,269], Peter Staib[565], Stephan Stilgenbauer[566], Stephanie Sungalee[8], Monika Szczepanowski[544], Umut H Toprak[53,567], Lorenz HP Trümper[556], Rabea Wagener[251,542] and Thorsten Zenz[79]

Tumor Specific Providers – Germany (Pediatric Brain cancer)

Ivo Buchhalter[51,52,53], Juergen Eils[64,65], Roland Eils[51,53,64,65], Volker Hovestadt[396], Barbara Hutter[77,78,79], David TW Jones[290,291], Natalie Jäger[51], Christof von Kalle[81], Marcel Kool[94,290], Jan O Korbel[7,8], Andrey Korshunov[94], Pablo Landgraf[568], Chris Lawerenz[65], Hans Lehrach[569], **Peter Lichter#**[77,396], Paul A Northcott[570], Stefan M Pfister[94,290,571], Bernhard Radlwimmer[396], Guido Reifenberger[568], Matthias Schlesner[51,104], Hans-Jörg Warnatz[569], Joachim Weischenfeldt[8,107,108], Stephan Wolf[572], Marie-Laure Yaspo[569] and Marc Zapatka[396]

Tumor Specific Providers – Germany (Prostate cancer)

Yassen Assenov[573], Benedikt Brors[79,113,267], Juergen Eils[64,65], Roland Eils[51,53,64,65], Lars Feuerbach[113], Clarissa Gerhauser[273], Jan O Korbel[7,8], Chris Lawerenz[65], Hans Lehrach[569], Sarah Minner[574], Christoph Plass[273], **Guido Sauter#**[575], Thorsten Schlomm[107,576], Nikos Sidiropoulos[108], Ronald Simon[575], **Holger Sültmann#**[79,577], Hans-Jörg Warnatz[569], Dieter Weichenhan[273], Joachim Weischenfeldt[8,107,108] and Marie-Laure Yaspo[569]

Tumor Specific Providers – India (Oral cancer)

Nidhan K Biswas[578], Luca Landoni[412], Arindam Maitra[578], **Partha P Majumder#**[578] and **Rajiv Sarin#**[579]

Tumor Specific Providers – Italy (Pancreatic cancer)

Davide Antonello[412], Stefano Barbi[420], Claudio Bassi[412], Samantha Bersani[415], Giada Bonizzato[416], Cinzia Cantù[416], Ivana Cataldo[415,416], Sara Cingarlini[36], Vincenzo Corbo[416,420], Maria Vittoria Davi[421], Angelo P Dei Tos[580], Matteo Fassan[581], Sonia Grimaldi[416], Luca Landoni[412], Rita T Lawlor[416], Claudio Luchini[415], Andrea Mafficini[416], Giuseppe Malleo[412], Giovanni Marchegiani[412], Michele

Milella[36], Marco Miotto[412], Salvatore Paiella[412], Antonio Pea[412], Paolo Pederzoli[412], Borislav C Rusev[416], Andrea Ruzzenente[412], Roberto Salvia[412], Maria Scardoni[415], **Aldo Scarpa#**[416], Elisabetta Sereni[412], Michele Simbolo[420], Nicola Sperandio[416], Giampaolo Tortora[36,37] and Caterina Vicentini[416]

Tumor Specific Providers – Japan (Biliary tract cancer)

Yasuhito Arai[33], Natsuko Hama[33], Nobuyoshi Hiraoka[582], Fumie Hosoda[33,33], Mamoru Kato[356], Hiromi Nakamura[33], Hidenori Ojima[583], Takuji Okusaka[584], **Tatsuhiro Shibata#**[33,34], Yasushi Totoki[33] and Tomoko Urushidate[34]

Tumor Specific Providers – Japan (Gastric cancer)

Hiroyuki Aburatani#[261], Yasuhito Arai[33], Masashi Fukayama[585], Natsuko Hama[33], Fumie Hosoda[33,33], Shumpei Ishikawa[586], Hitoshi Katai[587], Mamoru Kato[356], Hiroto Katoh[586], Daisuke Komura[586], Genta Nagae[261,272], Hiromi Nakamura[33], Hirofumi Rokutan[356], Mihoko Saito-Adachi[356], **Tatsuhiro Shibata#**[33,34], Akihiro Suzuki[261,588], Hirokazu Taniguchi[589], Kenji Tatsuno[261], Yasushi Totoki[33], Tetsuo Ushiku[585], Shinichi Yachida[33,590] and Shogo Yamamoto[261]

Tumor Specific Providers – Japan (Liver cancer)

Hiroyuki Aburatani[261], Hiroshi Aikata[591], Koji Arihiro[591], Shun-ichi Ariizumi[592], Keith A Boroevich[46,47], Kazuaki Chayama[591], Akihiro Fujimoto[47], Masashi Fujita[47], Mayuko Furuta[47], Kunihito Gotoh[593], Natsuko Hama[33], Takanori Hasegawa[83], Shinya Hayami[594], Shuto Hayashi[83], Satoshi Hirano[595], Seiya Imoto[83,83], Mamoru Kato[356], Yoshiiku Kawakami[591], Kazuhiro Maejima[47], Satoru Miyano[83], Genta Nagae[261,272], **Hidewaki Nakagawa#**[47], Hiromi Nakamura[33], Toru Nakamura[595], Kaoru Nakano[47], Hideki Ohdan[591], Aya Sasaki-Oku[47], **Tatsuhiro Shibata#**[33,34], Yuichi Shiraishi[83], Hiroko Tanaka[83], Yasushi Totoki[33], Tatsuhiko Tsunoda[46,211,212,213], Masaki Ueno[594], Rui Yamaguchi[83], Masakazu Yamamoto[592] and Hiroki Yamaue[594]

Tumor Specific Providers – Singapore (Biliary tract cancer)

Su Pin Choo[596], Ioana Cutcutache[256,308], Narong Khuntikeo[412,597], John R McPherson[256,308], Choon Kiat Ong[598], Chawalit Pairojkul[408], Irinel Popescu[599], **Steven G Rozen#**[256,257,308], **Patrick Tan#**[244,255,256,257] and **Bin Tean Teh#**[255,256,257,258,259]

Tumor Specific Providers – South Korea (Blood cancer)

Keun Soo Ahn[600], Hyung-Lae Kim[27], Youngil Koh[296,297] and **Sung-Soo Yoon#**[297]

Tumor Specific Providers – Spain (Chronic Lymphocytic Leukemia)

Marta Aymerich[601], **Elias Campo#**[602,603], Josep Ll Gelpi[45,68], Ivo G Gut[128,129], Marta Gut[128,129], Armando Lopez-Guillermo[604], Carlos López-Otín[605], Xose S Puente[605], Romina Royo[45] and David Torrents[45,105]

Tumor Specific Providers – United Kingdom (Bone cancer)

Fernanda Amary[606], Daniel Baumhoer[607], Sam Behjati[2], Bodil Bjerkehagen[607,608], **Peter J Campbell#**[1,2], **Adrienne M Flanagan#**[609], PA Futreal[505], Ola Myklebost[516], Nischalan Pillay[610], Patrick Tarpey[611], Roberto Tirabosco[612] and Olga Zaikova[613]

Tumor Specific Providers – United Kingdom (Chronic myeloid disorders)

Jacqueline Boultwood[614], David T Bowen[2], Adam P Butler[2], **Peter J Campbell#**[1,2], Mario Cazzola[615], Carlo Gambacorti-Passerini[181], Anthony R Green[284], Eva Hellstrom-Lindberg[616], Luca Malcovati[615], Sancha Martin[2,358], Jyoti Nangalia[617], Elli Papaemmanuil[2] and Paresh Vyas[300,618]

Tumor Specific Providers – United Kingdom (Esophageal cancer)

Yeng Ang[619], Hugh Barr[620], Duncan Beardsmore[621], Matthew Eldridge[283], **Rebecca C Fitzgerald#**[314], James Gossage[622], Nicola Grehan[314], George B Hanna[623], Stephen J Hayes[624,625], Ted R Hupp[626], David Khoo[627], Jesper Lagergren[616,628], Laurence B Lovat[242], Shona MacRae[384], Maria O'Donovan[314], J Robert O'Neill[629], Simon L Parsons[630], Shaun R Preston[631], Sonia Puig[632], Tom Roques[633], Grant Sanders[239], Sharmila Sothi[634], Simon Tavaré[283], Olga Tucker[635], Richard Turkington[636], Timothy J Underwood[637] and Ian Welch[638]

Tumor Specific Providers – United Kingdom (Prostate cancer)

Daniel M Berney[639], Johann S De Bono[393], G Steven Bova[320], Daniel S Brewer[391,392], Adam P Butler[2], Declan Cahill[640], Niedzica Camacho[393], **Colin S Cooper#**[392,393,394], Nening M Dennis[640], Tim Dudderidge[640,641], Sandra E Edwards[393], **Rosalind A Eeles#**[393,640], Cyril Fisher[640], Christopher S Foster[642,643], Mohammed Ghori[2], Pelvender Gill[618], Vincent J Gnanapragasam[372,644], Gunes Gundem[192], Freddie C Hamdy[645], Steve Hawkins[283], Steven Hazell[640], William Howat[372], William B Isaacs[646], Katalin Karaszi[618], Jonathan D Kay[242], Vincent Khoo[640], Zsofia Kote-Jarai[393], Barbara Kremeyer[2], Pardeep Kumar[640], Adam Lambert[618], Daniel A Leongamornlert[2,393], Naomi Livni[640], Yong-Jie Lu[639,647], Hayley J Luxton[242], Andy G Lynch[283,284,299], Luke Marsden[618], Charlie E Massie[283],

Lucy Matthews[393], Erik Mayer[640,648], Ultan McDermott[2], Sue Merson[393], Thomas J Mitchell[2,284,372], David E Neal[283,372], Anthony Ng[649], David Nicol[640], Christopher Ogden[640], Edward W Rowe[640], Nimish C Shah[372], Jon W Teague[2], Sarah Thomas[640], Alan Thompson[640], Peter Van Loo[61,62], Clare Verrill[618,650], Tapio Visakorpi[320], Anne Y Warren[372,651], David C Wedge[2,345,346], Hayley C Whitaker[242], Jorge Zamora[2,275,276,277], Hongwei Zhang[647] and Nicholas van As[640]

Tumor Specific Providers – United States (TCGA)

Adam Abeshouse[192], Nishant Agrawal[69], Rehan Akbani[314,652], Hikmat Al-Ahmadie[192], Monique Albert[450], Kenneth Aldape[397,653], Adrian Ally[656],Yeng Ang[619], Elizabeth L Appelbaum[132,242], Joshua Armenia[654], Sylvia Asa[630,655], J Todd Auman[657], Matthew H Bailey[131,132], Miruna Balasundaram[656], Saianand Balu[239], Jill Barnholtz-Sloan[658,659], Hugh Barr[620], John Bartlett[449,450], Oliver F Bathe[660,661], Stephen B Baylin[324,641], Duncan Beardsmore[621], Christopher Benz[662], Andrew Berchuck[663], Benjamin P Berman[262,263,264], Rameen Beroukhim[3,6,166], Mario Berrios[664], Darell Bigner[,665], Michael Birrer[101], Tom Bodenheimer[239], Lori Boice[632], Moiz S Bootwalla[664], Marcus Bosenberg[666], Reanne Bowlby[656], Jeffrey Boyd[667], Russell R Broaddus[397], Malcolm Brock[668], Denise Brooks[656], Susan Bullman[3,166], Samantha J Caesar-Johnson[39], Thomas E Carey[669], Rebecca Carlsen[656], Robert Cerfolio[670], Vishal S Chandan[671], Hsiao-Wei Chen[619,654], Andrew D Cherniack[3,3,48,166], Jeremy Chien[672], Juok Cho[3], Eric Chuah[656], Carrie Cibulskis[3], Kristian Cibulskis[3], Leslie Cope[673], Matthew G Cordes[132,633], Kyle Covington[157], Erin Curley[674], Bogdan Czerniak[397,627], Ludmila Danilova[673], Ian J Davis[675], Timothy Defreitas[3], John A Demchok[39], Noreen Dhalla[656], Rajiv Dhir[676], Li Ding[131,132,139], HarshaVardhan Doddapaneni[157], Adel El-Naggar[397,627], Ina Felau[39], Martin L Ferguson[677], Gaetano Finocchiaro[678], Kwun M Fong[679], Scott Frazer[3], William Friedman[680], Catrina C Fronick[132,633], Lucinda A Fulton[132], Robert S Fulton[131,132,139], Stacey B Gabriel[3], Jianjiong Gao[654], Nils Gehlenborg[3,681], Jeffrey E Gershenwald[682,683], Gad Getz[3,4,5,6], Ronald Ghossein[508], Nasra H Giama[684], Richard A Gibbs[157], Carmen Gomez[685], James Gossage[622], Ramaswamy Govindan[131], Nicola Grehan[314], George B Hanna[623], D Neil Hayes[239,686,687], Stephen J Hayes[624,625], Apurva M Hegde[384,385], David I Heiman[3], Zachary Heins[192], Austin J Hepperla[239], Katherine A Hoadley[238,239], Andrea Holbrook[664], Robert A Holt[656], Alan P Hoyle[239], Ralph H Hruban[673,673], Jianhong Hu[157], Mei Huang[632], David Huntsman[688], Ted R Hupp[626], Jason Huse[192], **Carolyn M Hutter#**[21], Christine A Iacobuzio-Donahue[508], Michael Ittmann[689,690], Joy C Jayaseelan[157], Stuart R Jefferys[239], Corbin D Jones[691], Steven JM Jones[692], Hartmut Juhl[693], Koo Jeong Kang[694], Beth Karlan[695], Katayoon Kasaian[692], Electron Kebebew[696,697], David Khoo[627], Hark Kyun Kim[698], Jaegil Kim[3], Tari A King[514,515], Viktoriya Korchina[157], Ritika Kundra[619,654], Jesper Lagergren[616,628], Phillip H Lai[664], Peter W Laird[270], Eric Lander[3], Michael S Lawrence[3,46,101], Alexander J Lazar[357], Xuan Le[699], Darlene Lee[656], Douglas A Levine[192,700], Lora Lewis[157], Tim Ley[701], Haiyan Irene Li[656], Pei Lin[3], W M Linehan[702], Eric Minwei Liu[111,112,192], Fei Fei Liu[373], Laurence B Lovat[242], Yiling Lu[385], Lisa Lype[703], Yussanne Ma[656], Shona MacRae[384], Dennis T Maglinte[664,704], Elaine R Mardis[132,667,705], Jeffrey Marks[412,706], Marco A Marra[656], Thomas J Matthew[49], Michael Mayo[656], Karen McCune[707], Michael D McLellan[131,132,139], Samuel R Meier[3], Shaowu Meng[239], Matthew Meyerson[3,6,48], Piotr A Mieczkowski[238], Tom Mikkelsen[708], Christopher A Miller[132], Gordon B Mills[,709], Richard A Moore[656], Carl Morrison[408,710], Lisle E Mose[239], Catherine D Moser[684], Andrew J Mungall[656], Karen Mungall[656], David Mutch[711], Donna M Muzny[712], Jerome Myers[713], Yulia Newton[49], Michael S Noble[3], Peter O'Donnell[714], Brian Patrick O'Neill[715], Angelica Ochoa[192], Akinyemi I Ojesina[245,246,247], Joong-Won Park[716], Joel S

Parker[717], Simon L Parsons[630], Harvey Pass[718], Alessandro Pastore[86], Chandra Sekhar Pedamallu[3,6,166], Nathan A Pennell[719], Charles M Perou[720], Gloria M Petersen[461], Nicholas Petrelli[721], Olga Potapova[722], Shaun R Preston[631], Sonia Puig[632], Janet S Rader[723], Suresh Ramalingam[724], W Kimryn Rathmell[725], Victor Reuter[508], Sheila M Reynolds[703], Matthew Ringel[726], Jeffrey Roach[727], Lewis R Roberts[684], A Gordon Robertson[656], Tom Roques[633], Mark A Rubin[187,202,203,204,205], Sara Sadeghi[656], Gordon Saksena[3], Charles Saller[728], Francisco Sanchez-Vega[619,654], Chris Sander[48,86,207,208], Grant Sanders[239], Dirk Schadendorf[77,729], Jacqueline E Schein[656], Heather K Schmidt[132], Nikolaus Schultz[654], Steven E Schumacher[3,209], Richard A Scolyer[410,441,446,447], Raja Seethala[730], Yasin Senbabaoglu[86], Troy Shelton[674], Yan Shi[239], Juliann Shih[3,166], Ilya Shmulevich[703], Craig Shriver[731], Sabina Signoretti[166,171,732], Janae V Simons[239], Samuel Singer[412,733], Payal Sipahimalani[656], Tara J Skelly[238], Karen Smith-McCune[707], Nicholas D Socci[86], Heidi J Sofia[21], Matthew G Soloway[717], Anil K Sood[734], Sharmila Sothi[634], Angela Tam[656], Donghui Tan[238], Roy Tarnuzzer[39], Nina Thiessen[656], R Houston Thompson[735], Leigh B Thorne[632], Ming Tsao[630,655], Olga Tucker[635], Richard Turkington[636], Christopher Umbricht[279,621,736], Timothy J Underwood[637], David J Van Den Berg[664], Erwin G Van Meir[737], Umadevi Veluvolu[238], Douglas Voet[3], Jiayin Wang[132,146,153], Linghua Wang[157], Zhining Wang[39], Paul Weinberger[738], John N Weinstein[384,385], Daniel J Weisenberger[664], Ian Welch[638], David A Wheeler[156,157], Dennis Wigle[739], Matthew D Wilkerson[238], Richard K Wilson[132,740], Boris Winterhoff[741], Maciej Wiznerowicz[742,743], Tina Wong[132,656], Winghing Wong[744], Liu Xi[157], Liming Yang[39], Christina Yau[662], Venkata D Yellapantula[159,160], **Jean C Zenklusen**#[39], Hailei Zhang[3], Hongxin Zhang[654] and Jiashan Zhang[39]

# Denotes **working group or project co-leader**

Author Affiliations

1. Department of Haematology, University of Cambridge, Cambridge CB2 2XY, UK.
2. Wellcome Sanger Institute, Wellcome Genome Campus, Hinxton, Cambridge, CB10 1SA, UK.
3. Broad Institute of MIT and Harvard, Cambridge, MA 02142, USA.
4. Center for Cancer Research, Massachusetts General Hospital, Boston, MA 02129, USA.
5. Department of Pathology, Massachusetts General Hospital, Boston, MA 02115, USA.
6. Harvard Medical School, Boston, MA 02115, USA.
7. European Molecular Biology Laboratory, European Bioinformatics Institute (EMBL-EBI), Wellcome Genome Campus, Hinxton, Cambridge, CB10 1SD, UK.
8. Genome Biology Unit, European Molecular Biology Laboratory (EMBL), Heidelberg 69117, Germany.
9. Computational Biology Program, Ontario Institute for Cancer Research, Toronto, ON M5G 0A3, Canada.
10. Department of Molecular Genetics, University of Toronto, Toronto, ON M5S 1A8, Canada.
11. Biomolecular Engineering Department, University of California Santa Cruz, Santa Cruz, CA 95064, USA.
12. King Faisal Specialist Hospital and Research Centre, Al Maather, Riyadh 12713, Saudi Arabia.
13. DLR Project Management Agency, Bonn 53227, Germany.
14. Genome Canada, Ottawa, ON K2P 1P1, Canada.
15. Instituto Carlos Slim de la Salud, Mexico City, Mexico.
16. Federal Ministry of Education and Research, Berlin 10117, Germany.

17. Institut Gustave Roussy, Villejuif 94805, France.
18. Institut National du Cancer (INCA), Boulogne-Billancourt 92100, France.
19. The Wellcome Trust, London NW1 2BE, UK.
20. Prostate Cancer Canada, Toronto, ON M5C 1M1, Canada.
21. National Human Genome Research Institute, National Institutes of Health, Bethesda, MD 20892, USA.
22. Department of Biotechnology, Ministry of Science & Technology, Government of India, New Delhi, Delhi 110003, India.
23. Science Writer, Garrett Park, MD 20896, USA.
24. Adaptive Oncology Initiative, Ontario Institute for Cancer Research, Toronto, ON M5G 0A3, Canada.
25. International Cancer Genome Consortium (ICGC)/ICGC Accelerating Research in Genomic Oncology (ARGO) Secretariat, Toronto, ON M5G 0A3, Canada.
26. Cancer Research UK, London EC1V 4AD, UK.
27. Department of Biochemistry, College of Medicine, Ewha Womans University, Seoul 07895, South Korea.
28. Chinese Cancer Genome Consortium, Shenzhen 518083, China.
29. Laboratory of Molecular Oncology, Key Laboratory of Carcinogenesis and Translational Research (Ministry of Education), Peking University Cancer Hospital & Institute, Beijing 100142, China.
30. Peking University Cancer Hospital & Institute, Key Laboratory of Carcinogenesis and Translational Research (Ministry of Education), Peking University Cancer Hospital & Institute, Beijing 100142, China.
31. National Cancer Center, Tokyo 104-0045, Japan.
32. German Cancer Aid, Bonn 53113, Germany.
33. Division of Cancer Genomics, National Cancer Center Research Institute, National Cancer Center, Tokyo 104-0045, Japan.
34. Laboratory of Molecular Medicine, Human Genome Center, The Institute of Medical Science, The University of Tokyo, Minato-ku, Tokyo 108-8639, Japan.
35. Japan Agency for Medical Research and Development, Chiyoda-ku, Tokyo 100-0004, Japan.
36. Medical Oncology, University and Hospital Trust of Verona, Verona 37134, Italy.
37. University of Verona, Verona 37129, Italy.
38. BGI-Shenzhen, Shenzhen 518083, China.
39. National Cancer Institute, National Institutes of Health, Bethesda, MD 20892, USA.
40. Centre for Law and Genetics, University of Tasmania, Sandy Bay Campus, Hobart, TAS 7001, Australia.
41. Centre of Genomics and Policy, McGill University and Génome Québec Innovation Centre, Montreal, QC H3A 1A4, Canada.
42. Heidelberg Academy of Sciences and Humanities, Heidelberg 69120, Germany.
43. CAPHRI Research School, Maastricht University, Maastricht, ER 6200MD, The Netherlands.
44. Genome Informatics Program, Ontario Institute for Cancer Research, Toronto, ON M5G 0A3, Canada.
45. Barcelona Supercomputing Center (BSC), Barcelona 08034, Spain.
46. Laboratory for Medical Science Mathematics, RIKEN Center for Integrative Medical Sciences, Yokohama, Kanagawa 230-0045, Japan.

47. RIKEN Center for Integrative Medical Sciences, Yokohama, Kanagawa 230-0045, Japan.
48. Dana-Farber Cancer Institute, Boston, MA 02215, USA.
49. University of California Santa Cruz, Santa Cruz, CA 95064, USA.
50. Oregon Health & Science University, Portland, OR 97239, USA.
51. Division of Theoretical Bioinformatics, German Cancer Research Center (DKFZ), Heidelberg 69120, Germany.
52. Heidelberg Center for Personalized Oncology (DKFZ-HIPO), German Cancer Research Center, Heidelberg 69120, Germany.
53. Institute of Pharmacy and Molecular Biotechnology and BioQuant, Heidelberg University, Heidelberg 69120, Germany.
54. University of California San Diego, San Diego, CA 92093, USA.
55. PDXen Biosystems Inc, Seoul 4900, South Korea.
56. Electronics and Telecommunications Research Institute, Daejeon 34129, South Korea.
57. Seven Bridges Genomics, Charlestown, MA 02129, USA.
58. Annai Systems, Inc, Carlsbad, CA 92013, USA.
59. Department of Biomedical Data Science, Stanford University School of Medicine, Stanford, CA 94305, USA.
60. Department of Genetics, Stanford University School of Medicine, Stanford, CA 94305, USA.
61. The Francis Crick Institute, London NW1 1AT, UK.
62. University of Leuven, Leuven B-3000, Belgium.
63. The Hospital for Sick Children, Toronto, ON M5G 0A4, Canada.
64. Heidelberg University, Heidelberg 69120, Germany.
65. New BIH Digital Health Center, Berlin Institute of Health (BIH) and Charité - Universitätsmedizin Berlin, Berlin 10117, Germany.
66. Department of Biochemistry and Molecular Medicine, University of Montreal, Montreal, QC H3C 3J7, Canada.
67. CIBIO/InBIO - Research Center in Biodiversity and Genetic Resources, Universidade do Porto, Vairão 4485-601, Portugal.
68. Department Biochemistry and Molecular Biomedicine, University of Barcelona, Barcelona 08028, Spain.
69. University of Chicago, Chicago, IL 60637, USA.
70. Division of Biomedical Informatics, Department of Medicine, & Moores Cancer Center, UC San Diego School of Medicine, San Diego, CA 92093, USA.
71. Children's Hospital of Philadelphia, Philadelphia, PA 19146, USA.
72. Massachusetts General Hospital Center for Cancer Research, Charlestown, MA 02129, USA.
73. University of Melbourne Centre for Cancer Research, Melbourne, VIC 3010, Australia.
74. Syntekabio Inc, Daejon 34025, South Korea.
75. AbbVie, North Chicago, IL 60064, USA.
76. Genomics Research Program, Ontario Institute for Cancer Research, Toronto, ON M5G 0A3, Canada.
77. German Cancer Consortium (DKTK), Heidelberg 69120, Germany.
78. Heidelberg Center for Personalized Oncology (DKFZ-HIPO), German Cancer Research Center (DKFZ), Heidelberg 69120, Germany.
79. National Center for Tumor Diseases (NCT) Heidelberg, Heidelberg 69120, Germany.
80. Department of Pediatric Immunology, Hematology and Oncology, University Hospital,

Heidelberg 69120, Germany.
81. German Cancer Research Center (DKFZ), Heidelberg 69120, Germany.
82. Heidelberg Institute for Stem Cell Technology and Experimental Medicine (HI-STEM), Heidelberg 69120, Germany.
83. The Institute of Medical Science, The University of Tokyo, Tokyo 108-8639, Japan.
84. Seven Bridges, Charlestown, MA 02129, USA.
85. Genome Integration Data Center, Syntekabio, Inc, Daejon, 34025, South Korea.
86. Computational Biology Center, Memorial Sloan Kettering Cancer Center, New York, NY 10065, USA.
87. Department of Biology, ETH Zurich, Zürich 8093, Switzerland.
88. Department of Computer Science, ETH Zurich, Zurich 8092, Switzerland.
89. SIB Swiss Institute of Bioinformatics, Lausanne 1015, Switzerland.
90. University Hospital Zurich, Zurich, 8091, Switzerland.
91. Health Sciences Department of Biomedical Informatics, University of California San Diego, La Jolla, CA 92093, USA.
92. Department of Health Sciences and Technology, Sungkyunkwan University School of Medicine, Seoul 06351, South Korea.
93. Samsung Genome Institute, Seoul 06351, South Korea.
94. Functional and Structural Genomics, German Cancer Research Center (DKFZ), Heidelberg 69120, Germany.
95. Leidos Biomedical Research, Inc, McLean, VA 22102, USA.
96. Sage Bionetworks, Seattle, WA 98109, USA.
97. Department of Cell and Systems Biology, University of Toronto, Toronto, ON M5S 3G5, Canada.
98. Genome Informatics, Ontario Institute for Cancer Research, Toronto, ON M5G 2C4, Canada.
99. Department of Radiation Oncology, University of California San Francisco, San Francisco, CA 94518, USA.
100. CSRA Incorporated, Fairfax, VA 22042, USA.
101. Massachusetts General Hospital, Boston, MA 02114, USA.
102. University Hospital Zurich, Zurich 8091, Switzerland.
103. Weill Cornell Medical College, New York, NY 10065, USA.
104. Bioinformatics and Omics Data Analytics, German Cancer Research Center (DKFZ), Heidelberg 69120, Germany.
105. Institució Catalana de Recerca i Estudis Avançats (ICREA), Barcelona 08010, Spain.
106. Department of Clinical and Molecular Medicine, Faculty of Medicine and Health Sciences, Norwegian University of Science and Technology, Trondheim 7030, Norway.
107. Department of Urology, Charité Universitätsmedizin Berlin, Berlin 10117, Germany.
108. Finsen Laboratory and Biotech Research & Innovation Centre (BRIC), University of Copenhagen, Copenhagen 2200, Denmark.
109. Department of Biological Oceanography, Leibniz Institute of Baltic Sea Research, Rostock 18119, Germany.
110. Ontario Institute for Cancer Research, Toronto, ON M5G 0A3, Canada.
111. Department of Physiology and Biophysics, Weill Cornell Medicine, New York, NY 10065, USA.
112. Institute for Computational Biomedicine, Weill Cornell Medicine, New York, NY 10021,

USA.
113. Division of Applied Bioinformatics, German Cancer Research Center (DKFZ), Heidelberg 69120, Germany.
114. Department of Computer Science, Yale University, New Haven, CT 06520, USA.
115. Department of Molecular Biophysics and Biochemistry, Yale University, New Haven, CT 06520, USA.
116. Program in Computational Biology and Bioinformatics, Yale University, New Haven, CT 06520, USA.
117. Department of Internal Medicine, Stanford University, Stanford, CA 94305, USA.
118. Department of Molecular Medicine (MOMA), Aarhus University Hospital, Aarhus N 8200, Denmark.
119. Clinical Bioinformatics, Swiss Institute of Bioinformatics, Geneva 1202, Switzerland.
120. Institute for Pathology and Molecular Pathology, University Hospital Zurich, Zurich 8091, Switzerland.
121. Institute of Molecular Life Sciences, University of Zurich, Zurich 8057, Switzerland.
122. MIT Computer Science and Artificial Intelligence Laboratory, Massachusetts Institute of Technology, Cambridge, MA 02139, USA.
123. Controlled Department and Institution, New York, NY 10065, USA.
124. Englander Institute for Precision Medicine, Weill Cornell Medicine, New York, NY 10065, USA.
125. Bioinformatics Research Centre (BiRC), Aarhus University, Aarhus 8000, Denmark.
126. Department of Medical Biophysics, University of Toronto, Toronto, ON M5S 1A8, Canada.
127. Institute of Molecular Life Sciences and Swiss Institute of Bioinformatics, University of Zurich, Zurich 8057, Switzerland.
128. CNAG-CRG, Centre for Genomic Regulation (CRG), Barcelona Institute of Science and Technology (BIST), Barcelona 08028, Spain.
129. Universitat Pompeu Fabra (UPF), Barcelona 08003, Spain.
130. Office of Cancer Genomics, National Cancer Institute, National Institutes of Health, Bethesda, MD 20892, USA.
131. Alvin J. Siteman Cancer Center, Washington University School of Medicine, St Louis, MO 63110, USA.
132. The McDonnell Genome Institute at Washington University, St Louis, MO 63108, USA.
133. Computer Network Information Center, Chinese Academy of Sciences, Beijing 100190, China.
134. Center for Digital Health, Berlin Institute of Health and Charitè - Universitätsmedizin Berlin, Berlin 10117, Germany.
135. Department of Pharmacology, University of Toronto, Toronto, ON M5S 1A8, Canada.
136. University of California Los Angeles, Los Angeles, CA 90095, USA.
137. University of Texas MD Anderson Cancer Center, Houston, TX 77030, USA.
138. Department of Genetics and Informatics Institute, University of Alabama at Birmingham, Birmingham, AL 35294, USA.
139. Department of Genetics, Department of Medicine, Washington University in St Louis, St Louis, MO 63110, USA.
140. Centre for Genomic Regulation (CRG), The Barcelona Institute of Science and Technology, Barcelona 08003, Spain.

141. Department of Bioinformatics and Computational Biology, The University of Texas MD Anderson Cancer Center, Houston, TX 77030, USA.
142. Department of Urologic Sciences, University of British Columbia, Vancouver, BC V5Z 1M9, Canada.
143. Vancouver Prostate Centre, Vancouver, BC V6H 3Z6, Canada.
144. Division of Life Science and Applied Genomics Center, Hong Kong University of Science and Technology, Clear Water Bay, Hong Kong, China.
145. Geneplus-Shenzhen, Shenzhen 518122, China.
146. School of Computer Science and Technology, Xi'an Jiaotong University, Xi'an 710048, China.
147. Biobyte solutions GmbH, Heidelberg 69126, Germany.
148. Division of Oncology, Washington University School of Medicine, St Louis, MO 63110, USA.
149. Institute of Medical Genetics and Applied Genomics, University of Tübingen, Tübingen 72076, Germany.
150. Indiana University, Bloomington, IN 47405, USA.
151. Simon Fraser University, Burnaby, BC V5A 1S6, Canada.
152. Department of Computer Science, University of Toronto, Toronto, ON M5S 1A8, Canada.
153. School of Electronic and Information Engineering, Xi'an Jiaotong University, Xi'an 710048, China.
154. Department of Genetics, Washington University School of Medicine, St Louis, MO 63110, USA.
155. Department of Mathematics, Washington University in St Louis, St Louis, MO 63130, USA.
156. Department of Molecular and Human Genetics, Baylor College of Medicine, Houston, TX 77030, USA.
157. Human Genome Sequencing Center, Baylor College of Medicine, Houston, TX 77030, USA.
158. The First Affiliated Hospital, Xi'an Jiaotong University, Xi'an 710049, China.
159. Department of Epidemiology and Biostatistics, Memorial Sloan Kettering Cancer Center, New York, NY 10065, USA.
160. The McDonnell Genome Institute at Washington University, Department of Genetics, Department of Medicine, Siteman Cancer Center, Washington University in St Louis, St Louis, MO 63108, USA.
161. Department of Genomic Medicine, The University of Texas MD Anderson Cancer Center, Houston, TX 77030, USA.
162. Quantitative & Computational Biosciences Graduate Program, Baylor College of Medicine, Houston, TX 77030, USA.
163. The Jackson Laboratory for Genomic Medicine, Farmington, CT 06032, USA.
164. Dana-Farber/Boston Children's Cancer and Blood Disorders Center, Boston, MA 02215, USA.
165. Department of Pediatrics, Harvard Medical School, Boston, MA 02115, USA.
166. Department of Medical Oncology, Dana-Farber Cancer Institute, Boston, MA 02115, USA.
167. Department of Mathematics, Aarhus University, Aarhus 8000, Denmark.
168. Technical University of Denmark, Lyngby 2800, Denmark.
169. University of Copenhagen, Copenhagen 2200, Denmark.
170. Department for BioMedical Research, University of Bern, Bern 3008, Switzerland.
171. Department of Medical Oncology, Inselspital, University Hospital and University of Bern,

Bern 3010, Switzerland.
172. Graduate School for Cellular and Biomedical Sciences, University of Bern, Bern 3012, Switzerland.
173. Department of Genitourinary Medical Oncology - Research, Division of Cancer Medicine, The University of Texas MD Anderson Cancer Center, Houston, TX 77030, USA.
174. Department of Urology, Icahn School of Medicine at Mount Sinai, New York, NY 10029, USA.
175. Faculty of Biosciences, Heidelberg University, Heidelberg 69120, Germany.
176. Korea Advanced Institute of Science and Technology, Daejeon 34141, South Korea.
177. Institute for Research in Biomedicine (IRB Barcelona), The Barcelona Institute of Science and Technology, Barcelona 8003, Spain.
178. Research Program on Biomedical Informatics, Universitat Pompeu Fabra, Barcelona 08002, Spain.
179. Science for Life Laboratory, Department of Cell and Molecular Biology, Uppsala University, Uppsala SE-75124, Sweden.
180. Queensland Centre for Medical Genomics, Institute for Molecular Bioscience, The University of Queensland, St Lucia, Brisbane, QLD 4072, Australia.
181. University of Milano Bicocca, Monza 20052, Italy.
182. Peter MacCallum Cancer Centre, University of Melbourne, Melbourne, VIC 3000, Australia.
183. Sir Peter MacCallum Department of Oncology, Peter MacCallum Cancer Centre, University of Melbourne, Melbourne, VIC 3052, Australia.
184. Center for Precision Health, School of Biomedical Informatics, The University of Texas Health Science Center, Houston, TX 77030, USA.
185. The Donnelly Centre, University of Toronto, Toronto, ON M5S 3E1, Canada.
186. Health Data Science Unit, University Clinics, Heidelberg 69120, Germany.
187. Department for Biomedical Research, University of Bern, Bern 3008, Switzerland.
188. Research Core Center, National Cancer Centre Korea, Goyang-si 410-769, South Korea.
189. Institute of Computer Science, Polish Academy of Sciences, Warsawa 01-248, Poland.
190. Department of Biology, ETH Zurich, Wolfgang-Pauli-Strasse 27, 8093 Zürich, Switzerland.
191. Harvard University, Cambridge, MA 02138, USA.
192. Memorial Sloan Kettering Cancer Center, New York, NY 10065, USA.
193. Department of Information Technology, Ghent University, Ghent B-9000, Belgium.
194. Department of Plant Biotechnology and Bioinformatics, Ghent University, Ghent B-9000, Belgium.
195. Yale School of Medicine, Yale University, New Haven, CT 06520, USA.
196. Institute for Research in Biomedicine (IRB Barcelona), Barcelona 08028, Spain.
197. Division of Hematology-Oncology, Samsung Medical Center, Sungkyunkwan University School of Medicine, Seoul 06351, South Korea.
198. Samsung Advanced Institute for Health Sciences and Technology, Sungkyunkwan University School of Medicine, Seoul 06351, South Korea.
199. Cheonan Industry-Academic Collaboration Foundation, Sangmyung University, Cheonan 31066, South Korea.
200. Spanish National Cancer Research Centre, Madrid 28029, Spain.
201. Department of Computer Science, Princeton University, Princeton, NJ 08540, USA.
202. Bern Center for Precision Medicine, University Hospital of Bern, University of Bern, Bern

3008, Switzerland.
203. Englander Institute for Precision Medicine, Weill Cornell Medicine and New York Presbyterian Hospital, New York, NY 10021, USA.
204. Meyer Cancer Center, Weill Cornell Medicine, New York, NY 10065, USA.
205. Pathology and Laboratory, Weill Cornell Medical College, New York, NY 10021, USA.
206. Vall d'Hebron Institute of Oncology: VHIO, Barcelona 08035, Spain.
207. cBio Center, Dana-Farber Cancer Institute, Harvard Medical School, Boston, MA 02115, USA.
208. Department of Cell Biology, Harvard Medical School, Boston, MA 02115, USA.
209. Department of Cancer Biology, Dana-Farber Cancer Institute, Boston, MA 02215, USA.
210. cBio Center, Dana-Farber Cancer Institute, Boston, MA 02215, USA.
211. CREST, Japan Science and Technology Agency, Tokyo 113-0033, Japan.
212. Department of Medical Science Mathematics, Medical Research Institute, Tokyo Medical and Dental University, Bunkyo-ku, Tokyo 113-8510, Japan.
213. Laboratory for Medical Science Mathematics, Department of Biological Sciences, Graduate School of Science, The University of Tokyo, Bunkyo-ku, Tokyo 113-0033, Japan.
214. Science for Life Laboratory, Department of Oncology-Pathology, Karolinska Institutet, Stockholm 17121, Sweden.
215. Department of Gene Technology, Tallinn University of Technology, Tallinn 12616, Estonia.
216. Genetics & Genome Biology Program, SickKids Research Institute, The Hospital for Sick Children, Toronto, ON M5G 1X8, Canada.
217. Department of Information Technology, Ghent University, Interuniversitair Micro-Electronica Centrum (IMEC), Ghent B-9000, Belgium.
218. Science for Life Laboratory, Department of Immunology, Genetics and Pathology, Uppsala University, Uppsala SE-75108, Sweden.
219. Oregon Health & Sciences University, Portland, OR 97239, USA.
220. Department of Medicine and Therapeutics, The Chinese University of Hong Kong, Shatin, NT, Hong Kong, China.
221. The University of Texas Health Science Center at Houston, Houston, TX 77030, USA.
222. Department of Biomedical Informatics, College of Medicine, The Ohio State University, Columbus, OH 43210, USA.
223. The Ohio State University Comprehensive Cancer Center (OSUCCC – James), Columbus, OH 43210, USA.
224. The University of Texas School of Biomedical Informatics (SBMI) at Houston, Houston, TX 77030, USA.
225. Department of Biochemistry and Molecular Genetics, Feinberg School of Medicine, Northwestern University, Chicago, IL 60637, USA.
226. Wolfson Wohl Cancer Research Centre, Institute of Cancer Sciences, University of Glasgow, Bearsden, Glasgow G61 1QH, UK.
227. Centre for Molecular Science Informatics, Department of Chemistry, University of Cambridge, Cambridge CB2 1EW, UK.
228. Department of Biomedical Informatics, Harvard Medical School, Boston, MA 02115, USA.
229. Ludwig Center at Harvard Medical School, Boston, MA 02115, USA.
230. UC Santa Cruz Genomics Institute, University of California Santa Cruz, Santa Cruz, CA 95064, USA.

231. Physics Division, Optimization and Systems Biology Lab, Massachusetts General Hospital, Boston, MA 02114, USA.
232. Department of Medicine, Baylor College of Medicine, Houston, TX 77030, USA.
233. Computational and Systems Biology, Genome Institute of Singapore, Singapore 138672, Singapore.
234. School of Computing, National University of Singapore, Singapore 117417, Singapore.
235. The Azrieli Faculty of Medicine, Bar-Ilan University, Safed 13195, Israel.
236. National Cancer Centre Singapore, Singapore 169610, Singapore.
237. Peking University, Beijing 100871, China.
238. Department of Genetics, University of North Carolina at Chapel Hill, Chapel Hill, NC 27599, USA.
239. Lineberger Comprehensive Cancer Center, University of North Carolina at Chapel Hill, Chapel Hill, NC 27599, USA.
240. China National GeneBank-Shenzhen, Shenzhen 518083, China.
241. Berlin Institute for Medical Systems Biology, Max Delbrück Center for Molecular Medicine, Berlin 13125, Germany.
242. University College London, London WC1E 6BT, UK.
243. School of Life Sciences, Peking University, Beijing 100180, China.
244. Genome Institute of Singapore, Singapore 138672, Singapore.
245. Department of Epidemiology, University of Alabama at Birmingham, Birmingham, AL 35294, USA.
246. HudsonAlpha Institute for Biotechnology, Huntsville, AL 35806, USA.
247. O'Neal Comprehensive Cancer Center, University of Alabama at Birmingham, Birmingham, AL 35294, USA.
248. Department of Biosciences and Nutrition, Karolinska Institutet, Stockholm 14183, Sweden.
249. German Cancer Consortium (DKTK), Partner site Berlin.
250. Human Genetics, University of Kiel, Kiel 24118, Germany.
251. Institute of Human Genetics, Ulm University and Ulm University Medical Center, Ulm 89081, Germany.
252. Computational & Systems Biology Program, Memorial Sloan Kettering Cancer Center, New York, NY 10065, USA.
253. Korea University, Seoul 02481, South Korea.
254. Division of Computational Genomics and Systems Genetics, German Cancer Research Center (DKFZ), Heidelberg 69120, Germany.
255. Cancer Science Institute of Singapore, National University of Singapore, Singapore 169609, Singapore.
256. Programme in Cancer & Stem Cell Biology, Duke-NUS Medical School, Singapore 169857, Singapore.
257. SingHealth, Duke-NUS Institute of Precision Medicine, National Heart Centre Singapore, Singapore 169609, Singapore.
258. Institute of Molecular and Cell Biology, Singapore 169609, Singapore.
259. Laboratory of Cancer Epigenome, Division of Medical Science, National Cancer Centre Singapore, Singapore 169610, Singapore.
260. BIOPIC, ICG and College of Life Sciences, Peking University, Beijing 100871, China.
261. Genome Science Division, Research Center for Advanced Science and Technology, The

University of Tokyo, Tokyo 153-8904, Japan.
262. Center for Bioinformatics and Functional Genomics, Cedars-Sinai Medical Center, Los Angeles, CA 90048, USA.
263. Department of Biomedical Sciences, Cedars-Sinai Medical Center, Los Angeles, CA 90048, USA.
264. The Hebrew University Faculty of Medicine, Jerusalem 91120, Israel.
265. Bioinformatics Group, Department of Computer Science, University of Leipzig, Leipzig 04109, Germany.
266. Interdisciplinary Center for Bioinformatics, University of Leipzig, Leipzig 04109, Germany.
267. German Cancer Consortium (DKTK), German Cancer Research Center (DKFZ), Heidelberg 69120, Germany.
268. Computational Biology, Leibniz Institute on Aging - Fritz Lipmann Institute (FLI), Jena 07745, Germany.
269. Transcriptome Bioinformatics, LIFE Research Center for Civilization Diseases, University of Leipzig, Leipzig 04109, Germany.
270. Center for Epigenetics, Van Andel Research Institute, Grand Rapids, MI 49503, USA.
271. Institut d'Investigacions Biomèdiques August Pi i Sunyer (IDIBAPS), Barcelona 08036, Spain.
272. Research Center for Advanced Science and Technology, The University of Tokyo, Minato-ku, Tokyo 108-8639, Japan.
273. Cancer Epigenomics, German Cancer Research Center (DKFZ), Heidelberg 69120, Germany.
274. Van Andel Research Institute, Grand Rapids, MI 49503, USA.
275. Centre for Research in Molecular Medicine and Chronic Diseases (CiMUS), Universidade de Santiago de Compostela, Santiago de Compostela 15706, Spain.
276. Department of Zoology, Genetics and Physical Anthropology, (CiMUS), Universidade de Santiago de Compostela, Santiago de Compostela 15706, Spain.
277. The Biomedical Research Centre (CINBIO), Universidade de Vigo, Vigo 36310, Spain.
278. Transmissible Cancer Group, Department of Veterinary Medicine, University of Cambridge, Cambridge CB3 0ES, UK.
279. Department of Pathology, Johns Hopkins University School of Medicine, Baltimore, MD 21205, USA.
280. McKusick-Nathans Institute of Genetic Medicine, Sidney Kimmel Comprehensive Cancer Center, Johns Hopkins University School of Medicine, Baltimore, MD 21205, USA.
281. Foundation Medicine, Inc, Cambridge, MA 02141, USA.
282. University of Ottawa Faculty of Medicine, Department of Biochemistry, Microbiology and Immunology, Ottawa, ON K1H 8M5, Canada.
283. Cancer Research UK Cambridge Institute, University of Cambridge, Cambridge CB2 0RE, UK.
284. University of Cambridge, Cambridge CB2 1TN, UK.
285. Quantitative Genomics Laboratories (qGenomics), Barcelona 08950, Spain.
286. Genome Integrity and Structural Biology Laboratory, National Institute of Environmental Health Sciences (NIEHS), Durham, NC 27709, USA.
287. Brandeis University, Waltham, MA 02254, USA.
288. New York Genome Center, New York, NY 10013, USA.
289. Weill Cornell Medicine, New York, NY 10065, USA.
290. Hopp Children's Cancer Center (KiTZ), Heidelberg 69120, Germany.

291. Pediatric Glioma Research Group, German Cancer Research Center (DKFZ), Heidelberg 69120, Germany.
292. A.A. Kharkevich Institute of Information Transmission Problems, Moscow 127051, Russia.
293. Oncology and Immunology, Dmitry Rogachev National Research Center of Pediatric Hematology, Moscow 117997, Russia.
294. Skolkovo Institute of Science and Technology, Moscow 121205, Russia.
295. Integrative Bioinformatics Support Group, National Institute of Environmental Health Sciences (NIEHS), Durham, NC 27709, USA.
296. Center For Medical Innovation, Seoul National University Hospital, Seoul 03080, South Korea.
297. Department of Internal Medicine, Seoul National University Hospital, Seoul 03080, South Korea.
298. Division of Genetics and Genomics, Boston Children's Hospital, Harvard Medical School, Boston, MA 02115, USA.
299. School of Medicine/School of Mathematics and Statistics, University of St Andrews, St Andrews, Fife KY16 9SS, UK.
300. Department of Genetics and Computational Biology, QIMR Berghofer Medical Research Institute, Brisbane, QLD 4006, Australia.
301. Institute for Molecular Bioscience, University of Queensland, St Lucia, Brisbane, QLD 4072, Australia.
302. School of Molecular Biosciences and Center for Reproductive Biology, Washington State University, Pullman, WA 99164, USA.
303. Cancer Research Institute, Beth Israel Deaconess Medical Center, Boston, MA 02215, USA.
304. Ben May Department for Cancer Research, Department of Human Genetics, The University of Chicago, Chicago, IL 60637, USA.
305. Tri-Institutional PhD Program in Computational Biology and Medicine, Weill Cornell Medicine, New York, NY 10065, USA.
306. Department of Cellular and Molecular Medicine and Department of Bioengineering and Moores Cancer Center, University of California San Diego, La Jolla, CA 92093, USA.
307. Department of Cellular and Molecular Medicine and Department of Bioengineering and Moores Cancer Center, University of California, San Diego, La Jolla, CA 92093, USA.
308. Centre for Computational Biology, Duke-NUS Medical School, Singapore 169857, Singapore.
309. Department of Computer Science, University of Helsinki, Helsinki 00014, Finland.
310. Institute of Biotechnology, University of Helsinki, Helsinki 00014, Finland.
311. Organismal and Evolutionary Biology Research Programme, University of Helsinki, Helsinki 00014, Finland.
312. Programme in Cancer & Stem Cell Biology, Centre for Computational Biology, Duke-NUS Medical School, Singapore 169857, Singapore.
313. Academic Department of Medical Genetics, University of Cambridge, Addenbrooke's Hospital, Cambridge CB2 0QQ, UK.
314. MRC Cancer Unit, University of Cambridge, Cambridge CB2 0XZ, UK.
315. The University of Cambridge School of Clinical Medicine, Cambridge CB2 0SP, UK.
316. National Centre for Biological Sciences, Tata Institute of Fundamental Research, Bangalore 560065, India.

317. Department of Applied Mathematics and Theoretical Physics, Centre for Mathematical Sciences, University of Cambridge, Cambridge CB3 0WA, UK.
318. Department of Statistics, Columbia University, New York, NY 10027, USA.
319. Duke-NUS Medical School, Singapore 169857, Singapore.
320. Faculty of Medicine and Health Technology, Tampere University and Tays Cancer Center, Tampere University Hospital, Tampere FI-33014, Finland.
321. Bakar Computational Health Sciences Institute and Department of Pediatrics, University of California, San Francisco, CA 94158-2549, USA.
322. Division of Cancer Epidemiology and Genetics, National Cancer Institute, National Institutes of Health, Bethesda, MD 20892, USA.
323. Department of Biostatistics, Bloomberg School of Public Health, Johns Hopkins University, Baltimore, MD 21205, USA.
324. Department of Oncology, The Johns Hopkins School of Medicine, The Sidney Kimmel Comprehensive Cancer Center at Johns Hopkins University, Baltimore, MD 21230, USA.
325. Integrated Graduate Program in Physical and Engineering Biology, Yale University, New Haven, CT 06520, USA.
326. Department of Computational Biology, University of Lausanne, Lausanne 1015, Switzerland.
327. Department of Genetic Medicine and Development, University of Geneva Medical School, Geneva CH 1211, Switzerland.
328. Swiss Institute of Bioinformatics, University of Geneva, Geneva CH 1211, Switzerland.
329. Independent Consultant, Wellesley 02481, USA.
330. Centre for Cancer Genetic Epidemiology, Department of Oncology, University of Cambridge, Cambridge CB1 8RN, UK.
331. Centre for Cancer Genetic Epidemiology, Department of Public Health and Primary Care, University of Cambridge, Cambridge CB1 8RN, UK.
332. CIBER Epidemiología y Salud Pública (CIBERESP), Madrid 28029, Spain.
333. Research Group on Statistics, Econometrics and Health (GRECS), UdG, Barcelona 8041, Spain.
334. Oxford Nanopore Technologies, New York, NY 10013, USA.
335. Department of Medical Genetics, College of Medicine, Hallym University, Chuncheon 24252, South Korea.
336. Institute of Evolutionary Biology (UPF-CSIC), Department of Experimental and Health Sciences, Universitat Pompeu Fabra, Barcelona 08003, Spain.
337. Icahn School of Medicine at Mount Sinai, New York, NY 10029, USA.
338. Laboratory of Translational Genomics, Division of Cancer Epidemiology and Genetics, National Cancer Institute, National Institutes of Health, Bethesda, MD 20892, USA.
339. Department of Experimental and Health Sciences, Institute of Evolutionary Biology (UPF-CSIC), Universitat Pompeu Fabra, Barcelona 08003, Spain.
340. Institut Català de Paleontologia Miquel Crusafont, Universitat Autònoma de Barcelona, Barcelona 08193, Spain.
341. Applications Department, Oxford Nanopore Technologies, Oxford OX4 4DQ, UK.
342. Department of Genetics, Microbiology and Statistics, University of Barcelona, IRSJD, IBUB, Barcelona 08028, Spain.
343. Department of Ophthalmology and Ocular Genomics Institute, Massachusetts Eye and Ear,

Harvard Medical School, Boston, MA 02114, USA.
344. Department of Medical and Clinical Genetics, Genome-Scale Biology Research Program, University of Helsinki, Helsinki 00100, Finland.
345. Big Data Institute, Li Ka Shing Centre, University of Oxford, Oxford OX3 7LF, UK.
346. Oxford NIHR Biomedical Research Centre, University of Oxford, Oxford OX4 2PG, UK.
347. School of Electronic Information and Communications, Huazhong University of Science and Technology, Wuhan, Hubei 430074, China.
348. Bioinformatics Unit, Spanish National Cancer Research Centre (CNIO), Madrid 28029, Spain.
349. Vector Institute, Toronto, ON M5G 0A3, Canada.
350. South Western Sydney Clinical School, Faculty of Medicine, University of NSW, Liverpool, NSW 2170, Australia.
351. The Kinghorn Cancer Centre, Cancer Division, Garvan Institute of Medical Research, University of NSW, Sydney, NSW 2010, Australia.
352. West of Scotland Pancreatic Unit, Glasgow Royal Infirmary, Glasgow G31 2ER, UK.
353. University of Melbourne Centre for Cancer Research, Melbourne, VIC 3052, Australia.
354. MRC Human Genetics Unit, MRC IGMM, University of Edinburgh, Edinburgh EH4 2XU, UK.
355. Bioinformatics Group, Division of Molecular Biology, Department of Biology, Faculty of Science, University of Zagreb, Zagreb 10000, Croatia.
356. Department of Bioinformatics, Division of Cancer Genomics, National Cancer Center Research Institute, National Cancer Center, Tokyo 104-0045, Japan.
357. Departments of Pathology, Genomic Medicine and Translational Molecular Pathology, The University of Texas MD Anderson Cancer Center, Houston, TX 77030, USA.
358. University of Glasgow, Glasgow G61 1BD, UK.
359. MRC-University of Glasgow Centre for Virus Research, Glasgow G61 1QH, UK.
360. Wolfson Wohl Cancer Research Centre, Institute of Cancer Sciences, University of Glasgow, Bearsden, Glasgow G61 1BD, UK.
361. School of Computing Science, University of Glasgow, Glasgow G12 8RZ, UK.
362. Molecular and Medical Genetics, Oregon Health and Science University, Portland, OR 97201, USA.
363. Department of Surgery, University of Melbourne, Parkville, VIC 3010, Australia.
364. The Murdoch Children's Research Institute, Royal Children’s Hospital, Parkville, VIC 3052, Australia.
365. Walter + Eliza Hall Institute, Parkville, VIC 3052, Australia.
366. University of Cologne, Cologne 50931, Germany.
367. The Edward S. Rogers Sr. Department of Electrical and Computer Engineering, University of Toronto, Toronto, ON M5S 3G4, Canada.
368. University of Ljubljana, Ljubljana 1000, Slovenia.
369. Department of Public Health Sciences, The University of Chicago, Chicago, IL 60637, USA.
370. Research Institute, NorthShore University HealthSystem, Evanston, IL 60201, USA.
371. Department of Statistics, University of California Santa Cruz, Santa Cruz, CA 95064, USA.
372. Cambridge University Hospitals NHS Foundation Trust, Cambridge CB2 0QQ, UK.
373. University of Toronto, Toronto, ON M5G 2M9, Canada.
374. Department of Computer Science, Carleton College, Northfield, MN 55057, USA.
375. Molecular and Medical Genetics, Oregon Health & Science University, Portland, OR 97239,

USA.
376. Center for Psychiatric Genetics, NorthShore University HealthSystem, Evanston, IL 60201, USA.
377. Molecular and Medical Genetics, Knight Cancer Institute, Oregon Health & Science University, Portland, OR 97219, USA.
378. Argmix Consulting, North Vancouver, BC V7M 2J5, Canada.
379. Department of Computer Science, University of Toronto, Toronto, ON M5S 2E4, Canada.
380. Department of Biostatistics, The University of Texas MD Anderson Cancer Center, Houston, TX 77030, USA.
381. Department of Biostatistics, University of North Carolina at Chapel Hill, Chapel Hill, NC 27599, USA.
382. The University of Texas MD Anderson Cancer Center, Houston, TX 77030, USA.
383. Howard Hughes Medical Institute, University of California Santa Cruz, Santa Cruz, CA 95065, USA.
384. Cancer Unit, MRC University of Cambridge, Cambridge CB2 0XZ, UK.
385. Department of Bioinformatics and Computational Biology and Department of Systems Biology, The University of Texas MD Anderson Cancer Center, Houston, TX 77030, USA.
386. Department of Health Sciences, Faculty of Medical Sciences, Kyushu University, Fukuoka 812-8582, Japan.
387. Baylor College of Medicine, Houston, TX 77030, USA.
388. Department of Applied Mathematics and Statistics, Johns Hopkins University, Baltimore, MD 21218, USA.
389. Heinrich Pette Institute, Leibniz Institute for Experimental Virology, Hamburg 20251, Germany.
390. University Medical Center Hamburg-Eppendorf, Bioinformatics Core, Hamburg 20246, Germany.
391. Earlham Institute, Norwich NR4 7UZ, UK.
392. Norwich Medical School, University of East Anglia, Norwich NR4 7TJ, UK.
393. The Institute of Cancer Research, London SW7 3RP, UK.
394. University of East Anglia, Norwich NR4 7TJ, UK.
395. German Center for Infection Research (DZIF), Partner Site Hamburg-Borstel-Lübeck-Riems, Hamburg, Germany.
396. Division of Molecular Genetics, German Cancer Research Center (DKFZ), Heidelberg 69120, Germany.
397. Department of Pathology, The University of Texas MD Anderson Cancer Center, Houston, TX 77030, USA.
398. Sir Peter MacCallum Department of Oncology, Peter MacCallum Cancer Centre, University of Melbourne, Melbourne, VIC 3000, Australia.
399. QIMR Berghofer Medical Research Institute, Brisbane, QLD 4006, Australia.
400. Victorian Institute of Forensic Medicine, Southbank, VIC 3006, Australia.
401. University of Pennsylvania, Philadelphia, PA 19104, USA.
402. Centre for Cancer Research, The Westmead Institute for Medical Research, Sydney, NSW 2145, Australia.
403. Department of Gynaecological Oncology, Westmead Hospital, Sydney, NSW 2145, Australia.

404. Genetics and Molecular Pathology, SA Pathology, Adelaide, SA 5000, Australia.
405. Centre for Cancer Research, The Westmead Institute for Medical Research, The University of Sydney, Sydney, NSW 2145, Australia.
406. Department of Gynaecological Oncology, Westmead Hospital, Sydney, NSW 2006, Australia.
407. Garvan Institute of Medical Research, Darlinghurst, NSW 2010 Australia.
408. Department of Clinical Pathology, University of Melbourne, Melbourne, VIC 3052, Australia.
409. Centre for Cancer Research, The Westmead Institute for Medical Research, and Department of Gynaecological Oncology, Westmead Hospital, Sydney, NSW 2145, Australia.
410. The University of Sydney, Sydney, NSW 2006, Australia.
411. The Westmead Institute for Medical Research. The University of Sydney. The Department of Gynaecological Oncology, Westmead Hospital, Westmead, NSW 2145, Australia.
412. Department of Surgery, Pancreas Institute, University and Hospital Trust of Verona, Verona 37134, Italy.
413. Department of Surgery, Princess Alexandra Hospital, Woolloongabba QLD 4102, Australia.
414. Surgical Oncology Group, Diamantina Institute, The University of Queensland, Woolloongabba, Brisbane, QLD 4102, Australia.
415. Department of Diagnostics and Public Health, University and Hospital Trust of Verona, Verona 37134, Italy.
416. ARC-Net Centre for Applied Research on Cancer, University and Hospital Trust of Verona, Verona 37134, Italy.
417. Illawarra Shoalhaven Local Health District L3 Illawarra Cancer Care Centre, Wollongong Hospital, Wollongong NSW 2500, Australia.
418. University of Sydney, Sydney, NSW 2006, Australia.
419. School of Biological Sciences, The University of Auckland, Auckland 1010, New Zealand.
420. Department of Pathology and Diagnostics, University and Hospital Trust of Verona, Verona 37134, Italy.
421. Department of Medicine, Section of Endocrinology, University and Hospital Trust of Verona, Verona 37134, Italy.
422. Department of Pathology, Queen Elizabeth University Hospital, Glasgow G51 4TF, UK.
423. Department of Medical Oncology, Beatson West of Scotland Cancer Centre, Glasgow G12 0YN, UK.
424. Academic Unit of Surgery, School of Medicine, College of Medical, Veterinary and Life Sciences, University of Glasgow, Glasgow Royal Infirmary, Glasgow G4 OSF, UK.
425. Tissue Pathology and Diagnostic Oncology, Royal Prince Alfred Hospital, Camperdown, NSW 2050, Australia.
426. Discipline of Surgery, Western Sydney University, Penrith, NSW 2751, Australia.
427. Institute of Cancer Sciences, College of Medical Veterinary and Life Sciences, University of Glasgow, Glasgow G12 8QQ, UK.
428. The Kinghorn Cancer Centre, Cancer Division, Garvan Institute of Medical Research, University of NSW, Sydney, NSW 2109, Australia.
429. School of Environmental and Life Sciences, Faculty of Science, The University of Newcastle, Ourimbah, NSW 2258, Australia.
430. Eastern Clinical School, Monash University, Melbourne, VIC 3128, Australia.

431. Epworth HealthCare, Richmond, VIC 3121, Australia.
432. Applied Tumor Genomics Research Program, Research Programs Unit, University of Helsinki, Helsinki 00290, Finland.
433. Olivia Newton-John Cancer Research Institute, La Trobe University, Heidelberg, VIC 3084, Australia.
434. Melanoma Institute Australia, The University of Sydney, Wollstonecraft NSW 2065, Australia.
435. Children's Hospital at Westmead, The University of Sydney, Westmead, NSW 2145, Australia.
436. Melanoma Institute Australia, The University of Sydney, Sydney, NSW 2065, Australia.
437. Australian Institute of Tropical Health and Medicine, James Cook University, Douglas, QLD 4814, Australia.
438. Bioplatforms Australia, North Ryde, NSW 2109, Australia.
439. Melanoma Institute Australia, Macquarie University, Wollstonecraft NSW, 2109, Australia.
440. Children's Medical Research Institute, Westmead, NSW 2145 Australia.
441. Melanoma Institute Australia, The University of Sydney, Wollstonecraft 2065, NSW, Australia.
442. Melanoma Institute Australia, The University of Sydney, Wollstonecraft, NSW 2065, Australia.
443. Westmead Institute for Medical Research, University of Sydney, Westmead, NSW 2145 Australia.
444. Centre for Cancer Research, The Westmead Millennium Institute for Medical Research, University of Sydney, Westmead Hospital, Westmead, NSW 2145, Australia.
445. Centre for Cancer Research, Westmead Institute for Medical Research, Westmead, NSW 2145, Australia.
446. Discipline of Pathology, Sydney Medical School, The University of Sydney, Sydney, NSW 2065, Australia.
447. Royal Prince Alfred Hospital, Sydney, NSW 2050, Australia.
448. School of Mathematics and Statistics, The University of Sydney, Sydney, NSW 2006 Australia.
449. Diagnostic Development, Ontario Institute for Cancer Research, Toronto, ON M5G 0A3, Canada.
450. Ontario Tumour Bank, Ontario Institute for Cancer Research, Toronto, ON M5G 0A3, Canada.
451. PanCuRx Translational Research Initiative, Ontario Institute for Cancer Research, Toronto, ON M5G 0A3, Canada.
452. BioSpecimen Sciences Program, University Health Network, Toronto, ON M5G 2C4, Canada, Toronto, ON M5G 2C4, Canada.
453. Hepatobiliary/Pancreatic Surgical Oncology Program, University Health Network, Toronto, ON M5G 2C4, Canada.
454. Lunenfeld-Tanenbaum Research Institute, Mount Sinai Hospital, Toronto, ON M5G 1X5, Canada.
455. Division of Medical Oncology, Princess Margaret Cancer Centre, Toronto, ON M5G 2M9, Canada.
456. University of Nebraska Medical Center, Omaha, NE 68198-6880, USA.

457. BioSpecimen Sciences Program, University Health Network, Toronto, ON M5G 2C4, Canada.
458. Transformative Pathology, Ontario Institute for Cancer Research, Toronto, ON M5G 0A3, Canada.
459. Department of Biochemistry and Molecular Medicine, University California at Davis, Sacramento, CA 95817, USA.
460. University Health Network, Princess Margaret Cancer Centre, Toronto, ON M5G 1L7, Canada.
461. Department of Health Sciences Research, Mayo Clinic, Rochester, MN 55905, USA.
462. BioSpecimen Sciences, Laboratory Medicine (Toronto), Medical Biophysics, PanCuRX, Toronto, ON M5S 1A8, Canada.
463. Department of Laboratory Medicine and Pathobiology, University of Toronto, Toronto, ON M5S 1A8, Canada.
464. Department of Pathology, Human Oncology and Pathogenesis Program, Memorial Sloan Kettering Cancer Center, New York, NY 10053, USA.
465. Department of Medical Biophysics, University of Toronto, Toronto, ON M5G 1L7, Canada.
466. Human Longevity Inc, San Diego, CA 92121, USA.
467. CRUK Manchester Institute and Centre, Manchester M20 4GJ, UK.
468. Department of Radiation Oncology, University of Toronto, Toronto, ON M5S 1A8, Canada.
469. Manchester Cancer Research Centre, Cancer Division, FBMH, University of Manchester, Manchester M20 4GJ, UK.
470. Radiation Medicine Program, Princess Margaret Cancer Centre, Toronto, ON M5G 2M9, Canada.
471. Department of Surgical Oncology, Princess Margaret Cancer Centre, Toronto, ON M5G 2M9, Canada.
472. Genome Informatics Program, Ontario Institute for Cancer Research, Toronto, ON M5G 2C4, Canada.
473. STTARR Innovation Facility, Princess Margaret Cancer Centre, Toronto, ON M5G 1L7, Canada.
474. Department of Pathology, Toronto General Hospital, Toronto, ON M5G 2C4, Canada.
475. Hefei University of Technology, Anhui 230009, China.
476. State Key Laboratory of Cancer Biology, and Xijing Hospital of Digestive Diseases, Fourth Military Medical University, Shaanxi 710032, China.
477. Fourth Military Medical University, Shaanxi 710032, China.
478. Laboratory of Molecular Oncology, Key Laboratory of Carcinogenesis and Translational Research (Ministry of Education), Peking University Cancer Hospital & Institute, Beijing,100142, China.
479. Department of Surgery, Ruijin Hospital, Shanghai Jiaotong University School of Medicine, Shanghai 200025, China.
480. Leeds Institute of Medical Research, University of Leeds, St James's University Hospital, Leeds LS9 7TF, UK.
481. Canadian Center for Computational Genomics, McGill University, Montreal, QC H3A 0G1, Canada.
482. Department of Human Genetics, McGill University, Montreal, QC H3A 1B1, Canada.
483. International Agency for Research on Cancer, Lyon 69008, France.

484. McGill University and Genome Quebec Innovation Centre, Montreal, QC H3A 0G1, Canada.
485. Centre National de Génotypage, CEA - Institute de Génomique, Evry 91000, France.
486. Leeds Institute of Medical Research @ St James's, University of Leeds, St James's University Hospital, Leeds LS9 7TF, UK.
487. Institute of Mathematics and Computer Science, University of Latvia, Riga LV 1459, Latvia.
488. Department of Oncology, Gil Medical Center, Gachon University, Incheon 405-760, South Korea.
489. Department of Molecular Oncology, BC Cancer Agency, Vancouver, BC V5Z 1L3, Canada.
490. Los Alamos National Laboratory, Los Alamos, NM 87545, USA.
491. Department of Genetics, Institute for Cancer Research, Oslo University Hospital, The Norwegian Radium Hospital, Oslo O310, Norway.
492. Lund University, Lund 223 62, Sweden.
493. Translational Research Lab, Centre Léon Bérard, Lyon 69373, France.
494. Department of Molecular Biology, Faculty of Science, Radboud Institute for Molecular Life Sciences, Radboud University, Nijmegen 6500 HB, The Netherlands.
495. Department of Pathology, Brigham and Women's Hospital, Harvard Medical School, Boston, MA 02115, USA.
496. Department of Molecular Pathology, The Netherlands Cancer Institute, Amsterdam 1066 CX, The Netherlands.
497. Cancer Research UK Cambridge Institute, University of Cambridge, Li Ka Shing Centre, Cambridge CB2 0RE, UK.
498. Department of Oncology, University of Cambridge, Cambridge CB2 1TN, UK.
499. Breast Cancer Translational Research Laboratory JC Heuson, Institut Jules Bordet, Brussels 1000, Belgium.
500. Laboratory for Translational Breast Cancer Research, Department of Oncology, KU Leuven, Leuven 3000, Belgium.
501. Translational Cancer Research Unit, GZA Hospitals St.-Augustinus, Center for Oncological Research, Faculty of Medicine and Health Sciences, University of Antwerp, Antwerp 2000, Belgium.
502. Department of Gynecology & Obstetrics, Department of Clinical Sciences, Skåne University Hospital, Lund University, Lund SE-221 85, Sweden.
503. Icelandic Cancer Registry, Icelandic Cancer Society, Reykjavik 125, Iceland.
504. Department of Medical Oncology, Josephine Nefkens Institute and Cancer Genomics Centre, Erasmus Medical Center, Rotterdam 3015 CN, The Netherlands.
505. National Genotyping Center, Institute of Biomedical Sciences, Academia Sinica, Taipei 115, Taiwan.
506. Department of Pathology, Oslo University Hospital Ulleval, Oslo 0450, Norway.
507. Faculty of Medicine and Institute of Clinical Medicine, University of Oslo, Oslo NO-0316, Norway.
508. Department of Pathology, Memorial Sloan Kettering Cancer Center, New York, NY 10065, USA.
509. Department of Pathology, Skåne University Hospital, Lund University, Lund SE-221 85, Sweden.
510. Department of Pathology, Academic Medical Center, Amsterdam 1105 AZ, The Netherlands.

511. Department of Pathology, College of Medicine, Hanyang University, Seoul 133-791, South Korea.
512. Department of Pathology, Asan Medical Center, College of Medicine, Ulsan University, Songpa-gu, Seoul 05505, South Korea.
513. The Netherlands Cancer Institute, Amsterdam 1066 CX, The Netherlands.
514. Department of Surgery, Brigham and Women's Hospital, Dana-Farber Cancer Institute, Boston, MA 02115, USA.
515. Department of Surgery, Memorial Sloan-Kettering Cancer Center, New York, NY 10065, USA.
516. Department of Clinical Science, University of Bergen, Bergen 5020, Norway.
517. Morgan Welch Inflammatory Breast Cancer Research Program and Clinic, The University of Texas MD Anderson Cancer Center, Houston, TX 77030, USA.
518. The University of Queensland Centre for Clinical Research, The Royal Brisbane & Women's Hospital, Herston, QLD 4029, Australia.
519. Department of Pathology, Institut Jules Bordet, Brussels 1000, Belgium.
520. Institute for Bioengineering and Biopharmaceutical Research (IBBR), Hanyang University, Seoul 133-791, South Korea.
521. University of Oslo, Oslo 0316, Norway.
522. Institut Bergonié, Bordeaux 33076, France.
523. Department of Research Oncology, Guy's Hospital, King's Health Partners AHSC, King's College London School of Medicine, London SE1 9RT, UK.
524. University Hospital of Minjoz, INSERM UMR 1098, Besançon 25000, France.
525. Cambridge Breast Unit, Addenbrooke's Hospital, Cambridge University Hospital NHS Foundation Trust and NIHR Cambridge Biomedical Research Centre, Cambridge CB2 2QQ, UK.
526. East of Scotland Breast Service, Ninewells Hospital, Aberdeen AB25 2XF, UK.
527. Oncologie Sénologie, ICM Institut Régional du Cancer, Montpellier 34298, France.
528. Department of Radiation Oncology, Radboud University Nijmegen Medical Centre, Nijmegen 6525 GA, The Netherlands.
529. University of Iceland, Reykjavik 101, Iceland.
530. Dundee Cancer Centre, Ninewells Hospital, Dundee DD2 1SY, UK.
531. Institut Curie, INSERM Unit 830, Paris 75248, France.
532. Department of Laboratory Medicine, Radboud University Nijmegen Medical Centre, Nijmegen 6525 GA, The Netherlands.
533. Department of General Surgery, Singapore General Hospital, Singapore 169608, Singapore.
534. Universite Lyon, INCa-Synergie, Centre Léon Bérard, Lyon 69008, France.
535. Giovanni Paolo II / I.R.C.C.S. Cancer Institute, Bari BA 70124, Italy.
536. Department of Biopathology, Centre Léon Bérard, Lyon 69008, France.
537. Université Claude Bernard Lyon 1, Villeurbanne 69100, France.
538. NCCS-VARI Translational Research Laboratory, National Cancer Centre Singapore, Singapore 169610, Singapore.
539. Department of Pathology, Erasmus Medical Center Rotterdam, Rotterdam 3015 GD, The Netherlands.
540. Division of Molecular Carcinogenesis, The Netherlands Cancer Institute, Amsterdam 1066 CX, The Netherlands.
541. Division of Molecular Carcinogenesis, The Netherlands Cancer Institute, Amsterdam, The

Netherlands.
542. Institute of Human Genetics, Christian-Albrechts-University, Kiel 24118, Germany.
543. Institute of Human Genetics, Ulm University and Ulm University Medical Center of Ulm, Ulm 89081, Germany.
544. Hematopathology Section, Institute of Pathology, Christian-Albrechts-University, Kiel 24118, Germany.
545. Institute of Human Genetics, University of Ulm and University Hospital of Ulm, Ulm 89081, Germany.
546. Department of Human Genetics, Hannover Medical School, Hannover 30625, Germany.
547. Department of Pediatric Oncology, Hematology and Clinical Immunology, Heinrich-Heine-University, Düsseldorf 40225, Germany.
548. Department of Internal Medicine/Hematology, Friedrich-Ebert-Hospital, Neumünster 24534, Germany.
549. Pediatric Hematology and Oncology, University Hospital Muenster, Muenster 24534, Germany.
550. Department of Pediatrics, University Hospital Schleswig-Holstein, Kiel 24105, Germany.
551. Department of Medicine II, University of Würzburg, Würzburg, Germany.
552. Senckenberg Institute of Pathology, University of Frankfurt Medical School, Frankfurt 60596, Germany.
553. Institute of Pathology, Charité – University Medicine Berlin, Berlin 10117, Germany.
554. Department for Internal Medicine II, University Hospital Schleswig-Holstein, Kiel 24105, Germany.
555. Institute for Medical Informatics Statistics and Epidemiology, University of Leipzig, Leipzig 04109, Germany.
556. Department of Hematology and Oncology, Georg-Augusts-University of Göttingen, Göttingen 37073, Germany.
557. Institute of Cell Biology (Cancer Research), University of Duisburg-Essen, Essen D-45147, Germany.
558. MVZ Department of Oncology, PraxisClinic am Johannisplatz, Leipzig 04109, Germany.
559. Institute of Pathology, Ulm University and University Hospital of Ulm, Ulm 89081, Germany.
560. Department of Pathology, Robert-Bosch-Hospital, Stuttgart, Germany, Stuttgart 70376, Germany.
561. University Hospital Giessen, Pediatric Hematology and Oncology, Giessen 35392, Germany.
562. Institute of Clinical Molecular Biology, Christian-Albrechts-University, Kiel 24118, Germany.
563. Institute of Pathology, University of Wuerzburg, Wuerzburg 97070, Germany.
564. Department of General Internal Medicine, University Kiel, Kiel 24118, Germany.
565. Clinic for Hematology and Oncology, St.-Antonius-Hospital, Eschweiler D-52249, Germany.
566. Department for Internal Medicine III, University of Ulm and University Hospital of Ulm, Ulm 89081, Germany.
567. Neuroblastoma Genomics, German Cancer Research Center (DKFZ), Heidelberg 69120, Germany.
568. University of Düsseldorf, Düsseldorf 40225, Germany.
569. Department of Vertebrate Genomics/Otto Warburg Laboratory Gene Regulation and Systems Biology of Cancer, Max Planck Institute for Molecular Genetics, Berlin 14195, Germany.

570. St. Jude Children's Research Hospital, Memphis, TN 38105-3678, USA.
571. Heidelberg University Hospital, Heidelberg 69120, Germany.
572. Genomics and Proteomics Core Facility High Throughput Sequencing Unit, German Cancer Research Center (DKFZ), Heidelberg 69120, Germany.
573. Epigenomics and Cancer Risk Factors, German Cancer Research Center (DKFZ), Heidelberg 69120, Germany.
574. University Medical Center Hamburg-Eppendorf, Hamburg 20251, Germany.
575. Institute of Pathology, University Medical Center Hamburg-Eppendorf, Hamburg 20251, Germany.
576. Martini-Clinic, Prostate Cancer Center, University Medical Center Hamburg-Eppendorf, Hamburg 20095, Germany.
577. Division of Cancer Genome Research, German Cancer Research Center (DKFZ), Heidelberg 69120, Germany.
578. National Institute of Biomedical Genomics, Kalyani 741235, West Bengal, India.
579. Advanced Centre for Treatment Research & Education in Cancer, Tata Memorial Centre, Navi Mumbai, Maharashtra 410210, India.
580. Department of Pathology, General Hospital of Treviso, Department of Medicine, University of Padua, Treviso 31100, Italy.
581. Department of Medicine (DIMED), Surgical Pathology Unit, University of Padua, Padua 35121, Italy.
582. Department of Hepatobiliary and Pancreatic Oncology, Hepatobiliary and Pancreatic Surgery Division, Division of Pathology and Clinical Laboratories, National Cancer Center Hospital, Chuo-ku, Tokyo, 104-0045, Japan.
583. Department of Pathology, Keio University School of Medicine, Tokyo 160-8582, Japan.
584. Department of Hepatobiliary and Pancreatic Oncology, National Cancer Center Hospital, Tokyo, 104-0045 Japan.
585. Department of Pathology, Graduate School of Medicine, The University of Tokyo, Bunkyo-ku, Tokyo 113-0033, Japan.
586. Preventive Medicine, Graduate School of Medicine, The University of Tokyo, Tokyo 113-0033, Japan.
587. Gastric Surgery Division, Division of Pathology and Clinical Laboratories, National Cancer Center Hospital, Tokyo 104-0045, Japan.
588. Department of Gastroenterology and Hepatology, Yokohama City University Graduate School of Medicine, Kanagawa 236-0004, Japan.
589. Laboratory of Molecular Medicine, Human Genome Center, The Institute of Medical Science, University of Tokyo, Tokyo 108-8639, Japan.
590. Department of Cancer Genome Informatics, Graduate School of Medicine, Osaka University, Osaka 565-0871, Japan.
591. Hiroshima University, Hiroshima 734-8553, Japan.
592. Tokyo Women's Medical University, Tokyo 162-8666, Japan.
593. Osaka International Cancer Center, Osaka 541-8567, Japan.
594. Wakayama Medical University, Wakayama 641-8509, Japan.
595. Hokkaido University, Sapporo 060-8648, Japan.
596. Division of Medical Oncology, National Cancer Centre, Singapore 169610, Singapore.
597. Cholangiocarcinoma Screening and Care Program and Liver Fluke and Cholangiocarcinoma

Research Centre, Faculty of Medicine, Khon Kaen University, Khon Kaen 40002, Thailand.
598. Lymphoma Genomic Translational Research Laboratory, National Cancer Centre, Singapore 169610, Singapore.
599. Center of Digestive Diseases and Liver Transplantation, Fundeni Clinical Institute, Bucharest 022328, Romania.
600. Division of Hepatobiliary and Pancreatic Surgery, Department of Surgery, School of Medicine, Keimyung University Dongsan Medical Center, Daegu 41931, South Korea.
601. Pathology, Hospital Clinic, Institut d'Investigacions Biomèdiques August Pi i Sunyer (IDIBAPS), University of Barcelona, Barcelona 8034, Spain.
602. Anatomia Patológica, Hospital Clinic, Institut d'Investigacions Biomèdiques August Pi i Sunyer (IDIBAPS), University of Barcelona, Barcelona 8036, Spain.
603. Spanish Ministry of Science and Innovation, Madrid 28046, Spain.
604. Hematology, Hospital Clinic, Institut d'Investigacions Biomèdiques August Pi i Sunyer (IDIBAPS), University of Barcelona, Barcelona 8034, Spain.
605. Department of Biochemistry and Molecular Biology, Faculty of Medicine, University Institute of Oncology-IUOPA, Oviedo 33006, Spain.
606. Royal National Orthopaedic Hospital - Bolsover, London W1W 5AQ, UK.
607. Department of Pathology, Oslo University Hospital, The Norwegian Radium Hospital, Oslo O310, Norway.
608. Institute of Clinical Medicine and Institute of Oral Biology, University of Oslo, Oslo O310, Norway.
609. Department of Pathology (Research), University College London Cancer Institute, London WC1E 6BT, UK.
610. Research Department of Pathology, University College London Cancer Institute, London, WC1E 6BT, UK.
611. East Anglian Medical Genetics Service, Cambridge University Hospitals NHS Foundation Trust, Cambridge CB2 0QQ, UK.
612. Royal National Orthopaedic Hospital - Stanmore, Stanmore, Middlesex HA7 4LP, UK.
613. Division of Orthopaedic Surgery, Oslo University Hospital, Oslo 0379, Norway.
614. Radcliffe Department of Medicine, University of Oxford, Oxford OX3 9DU, UK.
615. University of Pavia, Pavia 27100, Italy.
616. Karolinska Institute, Stockholm SE-171 76, Sweden.
617. Wellcome Sanger Institute, Wellcome Genome Campus, Hinxton, Cambridge, CB10 1SD, UK.
618. University of Oxford, Oxford OX3 9DU, UK.
619. Salford Royal NHS Foundation Trust, Salford M6 8HD, UK.
620. Gloucester Royal Hospital, Gloucester GL1 3NL, UK.
621. Royal Stoke University Hospital, Stoke-on-Trent ST4 6QG, UK.
622. St Thomas's Hospital, London SE1 7EH, UK.
623. Imperial College NHS Trust, Imperial College, London W2 INY, UK.
624. Department of Histopathology, Salford Royal NHS Foundation Trust, Salford M6 8HD, UK.
625. Faculty of Biology, Medicine and Health, The University of Manchester, Manchester M13 9PL, UK.
626. Edinburgh Royal Infirmary, Edinburgh EH16 4SA, UK.
627. Barking Havering and Redbridge University Hospitals NHS Trust, Romford, RM7 0AG, UK.

628. King's College London and Guy's and St Thomas' NHS Foundation Trust, London SE1 7EH, UK.
629. Cambridge Oesophagogastric Centre, Cambridge University Hospitals NHS Foundation Trust, Cambridge, CB2 0QQ.
630. Nottingham University Hospitals NHS Trust, Nottingham NG7 2UH, UK.
631. St Luke's Cancer Centre, Royal Surrey County Hospital NHS Foundation Trust, Guildford GU2 7XX, UK.
632. University of North Carolina at Chapel Hill, Chapel Hill, NC 27599, USA.
633. Norfolk and Norwich University Hospital NHS Trust, Norwich NR4 7UY, UK.
634. University Hospitals Coventry and Warwickshire NHS Trust, Coventry CV2 2DX, UK.
635. University Hospitals Birmingham NHS Foundation Trust, Birmingham B15 2GW, UK.
636. Centre for Cancer Research and Cell Biology, Queen's University, Belfast BT9 7AB, UK.
637. School of Cancer Sciences, Faculty of Medicine, University of Southampton, Southampton SO17 1BJ, UK.
638. Wythenshawe Hospital, Manchester M23 9LT, UK.
639. Barts Cancer Institute, Barts and the London School of Medicine and Dentistry, Queen Mary University of London, London EC1M 6BQ, UK.
640. Royal Marsden NHS Foundation Trust, London and Sutton SW3 6JJ, UK.
641. University Hospital Southampton NHS Foundation Trust, Southampton SO16 6YD, UK.
642. HCA Laboratories, London W1G 8AQ, UK.
643. University of Liverpool, Liverpool L69 3BX, UK.
644. Academic Urology Group, Department of Surgery, University of Cambridge, Cambridge CB2 0QQ, UK.
645. University of Oxford, Oxford, OX3 9DU, UK.
646. Department of Urology, James Buchanan Brady Urological Institute, Johns Hopkins University School of Medicine, Baltimore, MD 21205, USA.
647. Second Military Medical University, Shanghai 200433, China.
648. Department of Surgery and Cancer, Imperial College, London W2 INY, UK.
649. The Chinese University of Hong Kong, Shatin, NT, Hong Kong, China.
650. Nuffield Department of Surgical Sciences, John Radcliffe Hospital, University of Oxford, Headington, Oxford OX3 9DU, UK.
651. Department of Histopathology, Cambridge University Hospitals NHS Foundation Trust, Cambridge CB2 0QQ, UK.
652. Department of Bioinformatics and Computational Biology / Department of Systems Biology, The University of Texas MD Anderson Cancer Center, Houston, TX 77030, USA.
653. Laboratory of Pathology, Center for Cancer Research, National Cancer Institute, Bethesda, MD 20892, USA.
654. Center for Molecular Oncology, Memorial Sloan Kettering Cancer Center, New York, NY 10065, USA.
655. University Health Network, Toronto, ON M5G 2C4, Canada.
656. Canada's Michael Smith Genome Sciences Center, BC Cancer Agency, Vancouver, BC V5Z 4S6, Canada.
657. Department of Pathology and Laboratory Medicine, School of Medicine, University of North Carolina at Chapel Hill, Chapel Hill, NC 27599, USA.
658. Department of Population and Quantitative Health Sciences, Case Western Reserve

University School of Medicine, Cleveland, OH 44016, USA.
659. Research Health Analytics and Informatics, University Hospitals Cleveland Medical Center, Cleveland, OH 44106, USA.
660. Arnie Charbonneau Cancer Institute, University of Calgary, Calgary, AB T2N 4N2, Canada.
661. Departments of Surgery and Oncology, University of Calgary, Calgary, AB T2N 4N2, Canada.
662. Buck Institute for Research on Aging, Novato, CA 94945, USA.
663. Duke University Medical Center, Durham, NC 27710, USA.
664. USC Norris Comprehensive Cancer Center, University of Southern California, Los Angeles, CA 90033, USA.
665. The Preston Robert Tisch Brain Tumor Center, Duke University Medical Center, Durham, NC 27710, USA.
666. Departments of Dermatology and Pathology, Yale University, New Haven, CT 06510, USA.
667. Fox Chase Cancer Center, Philadelphia, PA 19111, USA.
668. Department of Surgery, Division of Thoracic Surgery, The Johns Hopkins University School of Medicine, Baltimore, MD 21287, USA.
669. University of Michigan Comprehensive Cancer Center, Ann Arbor, MI 48109, USA.
670. University of Alabama at Birmingham, Birmingham, AL 35294, USA.
671. Division of Anatomic Pathology, Mayo Clinic, Rochester, MN 55905, USA.
672. Division of Experimental Pathology, Mayo Clinic, Rochester, MN 55905, USA.
673. Department of Oncology, The Johns Hopkins School of Medicine, The Sidney Kimmel Comprehensive Cancer Center at Johns Hopkins University, Baltimore, MD 21287, USA.
674. International Genomics Consortium, Phoenix, AZ 85004, USA.
675. Departments of Pediatrics and Genetics, University of North Carolina at Chapel Hill, Chapel Hill, NC 27599, USA.
676. Department of Pathology, UPMC Shadyside, Pittsburgh, PA 15232, USA.
677. Center for Cancer Genomics, National Cancer Institute, National Institutes of Health, Bethesda, MD 20892, USA.
678. Istituto Neurologico Besta, Department of Neuro-Oncology, Milano 20133, Italy.
679. University of Queensland Thoracic Research Centre, The Prince Charles Hospital, Brisbane, QLD 4032, Australia.
680. Department of Neurosurgery, University of Florida, Gainesville, FL 32610, USA.
681. Center for Biomedical Informatics, Harvard Medical School, Boston, MA 02115, USA.
682. Department of Cancer Biology, The University of Texas MD Anderson Cancer Center, Houston, TX 77030, USA.
683. Department of Surgical Oncology, The University of Texas MD Anderson Cancer Center, Houston, TX 77030, USA.
684. Division of Gastroenterology and Hepatology, Mayo Clinic, Rochester, MN 55905, USA.
685. University of Miami, Sylvester Comprehensive Cancer Center, Miami, FL 33136, USA.
686. Department of Internal Medicine, Division of Medical Oncology, Lineberger Comprehensive Cancer Center, University of North Carolina at Chapel Hill, Chapel Hill, NC 27599, USA.
687. University of Tennessee Health Science Center for Cancer Research, Memphis, TN 38163, USA.
688. Centre for Translational and Applied Genomics, British Columbia Cancer Agency, Vancouver, BC V5Z 1L3, Canada.

689. Department of Pathology & Immunology, Baylor College of Medicine, Houston, TX 77030, USA.
690. Michael E. DeBakey Veterans Affairs Medical Center, Houston, TX 77030, USA.
691. Carolina Center for Genome Sciences, University of North Carolina at Chapel Hill, Chapel Hill, NC 27599, USA.
692. Canada’s Michael Smith Genome Sciences Centre, BC Cancer Agency, Vancouver, BC V5Z 4S6, Canada.
693. Indivumed GmbH, Hamburg 20251, Germany.
694. Division of Hepatobiliary and Pancreatic Surgery, Department of Surgery, School of Medicine, Keimyung University Dong-san Medical Center, Daegu 41931, South Korea.
695. Women's Cancer Program at the Samuel Oschin Comprehensive Cancer Institute, Cedars-Sinai Medical Center, Los Angeles, CA 90048, USA.
696. Department of Surgery, The George Washington University, School of Medicine and Health Science, Washington, DC 20052, USA.
697. Endocrine Oncology Branch, Center for Cancer Research, National Cancer Institute, National Institutes of Health, Bethesda, MD 20892, USA.
698. National Cancer Center, Gyeonggi 10408, South Korea.
699. ILSbio, LLC Biobank, Chestertown, MD 21620, USA.
700. Gynecologic Oncology, NYU Laura and Isaac Perlmutter Cancer Center, New York University, New York, NY 10016, USA.
701. Division of Oncology, Stem Cell Biology Section, Washington University School of Medicine, St. Louis, MO 63110, USA.
702. Urologic Oncology Branch, Center for Cancer Research, National Cancer Institute, National Institutes of Health, Bethesda, MD 20892, USA.
703. Institute for Systems Biology, Seattle, WA 98109, USA.
704. Center for Personalized Medicine, Department of Pathology and Laboratory Medicine, Children’s Hospital Los Angeles, Los Angeles, CA 90027, USA.
705. Institute for Genomic Medicine, Nationwide Children's Hospital, Columbus, OH 43215, USA.
706. Department of Surgery, Duke University, Durham, NC 27710, USA.
707. Department of Obstetrics, Gynecology and Reproductive Services, University of California San Francisco, San Francisco, CA 94143, USA.
708. Departments of Neurology and Neurosurgery, Henry Ford Hospital, Detroit, MI 48202, USA.
709. Oregon Health & Science University (OHSU) Knight Cancer Institute, Portland, OR 97210, USA.
710. Department of Pathology, Roswell Park Cancer Institute, Buffalo, NY 14263, USA.
711. Department of Obstetrics and Gynecology, Division of Gynecologic Oncology, Washington University School of Medicine, St. Louis, MO 63110, USA.
712. Department of Palliative, Rehabilitation and Integrative Medicine, The University of Texas MD Anderson Cancer Center, Houston, TX 77030, USA.
713. Penrose St. Francis Health Services, Colorado Springs, CO 80907, USA.
714. The University of Chicago, Chicago, IL 60637, USA.
715. Department of Neurology, Mayo Clinic, Rochester, MN 55905, USA.
716. Center for Liver Cancer, Research Institute and Hospital, National Cancer Center, Gyeonggi

410-769, South Korea.
717. Department of Genetics and Lineberger Comprehensive Cancer Center, University of North Carolina at Chapel Hill, Chapel Hill, NC 27599, USA.
718. NYU Langone Medical Center, New York, NY 10016, USA.
719. Department of Hematology and Medical Oncology, Cleveland Clinic, Cleveland, OH 44195, USA.
720. Department of Genetics, Department of Pathology and Laboratory Medicine, School of Medicine, University of North Carolina at Chapel Hill, Chapel Hill, NC 27599, USA.
721. Helen F. Graham Cancer Center at Christiana Care Health Systems, Newark, DE 19713, USA.
722. Cureline, Inc, South San Francisco, CA 94080, USA.
723. Department of Obstetrics and Gynecology, Medical College of Wisconsin, Milwaukee, WI 53226, USA.
724. Hematology and Medical Oncology, Winship Cancer Institute of Emory University, Atlanta, GA 30322, USA.
725. Vanderbilt Ingram Cancer Center, Vanderbilt University, Nashville, TN 37232, USA.
726. Ohio State University College of Medicine and Arthur G. James Comprehensive Cancer Center, Columbus, OH 43210, USA.
727. Research Computing Center, University of North Carolina at Chapel Hill, Chapel Hill, NC 27599, USA.
728. Analytical Biological Services, Inc, Wilmington, DE 19801, USA.
729. Department of Dermatology, University Hospital Essen, Westdeutsches Tumorzentrum & German Cancer Consortium, Essen 45122, Germany.
730. University of Pittsburgh, Pittsburgh, PA 15213, USA.
731. Murtha Cancer Center, Walter Reed National Military Medical Center, Bethesda, MD 20889, USA.
732. Brigham and Women's Hospital, Harvard Medical School, Boston, MA 02115, USA.
733. Department of Surgery, Memorial Sloan Kettering Cancer Center, New York, NY 10065, USA.
734. Department of Gynecologic Oncology and Reproductive Medicine, and Center for RNA Interference and Non-Coding RNA, The University of Texas MD Anderson Cancer Center, Houston, TX 77030, USA.
735. Department of Urology, Mayo Clinic, Rochester, MN 55905, USA.
736. Department of Surgery, Johns Hopkins University School of Medicine, Baltimore, MD 21205, USA.
737. Departments of Neurosurgery and Hematology and Medical Oncology, Winship Cancer Institute and School of Medicine, Emory University, Atlanta, GA 30322, USA.
738. Georgia Regents University Cancer Center, Augusta, GA 30912, USA.
739. Thoracic Oncology Laboratory, Mayo Clinic, Rochester, MN 55905, USA.
740. Institute for Genomic Medicine, Nationwide Children’s Hospital, Columbus, OH 43205, USA.
741. Department of Obstetrics & Gynecology, Division of Gynecologic Oncology, Mayo Clinic, Rochester, MN 55905, USA.
742. International Institute for Molecular Oncology, Poznań 60-203, Poland.
743. Poznan University of Medical Sciences, Poznań 61-701, Poland.

744. Edison Family Center for Genome Sciences and Systems Biology, Washington University, St. Louis, MO 63110, USA.
