# Supplementary material for: Pathway and network analysis of more than 2500 whole cancer genomes
Source: Nat Commun. 2020 Feb 5;11:729. doi: 10.1038/s41467-020-14367-0 (PMC7002574; doi:10.1038/s41467-020-14367-0)
Supplement: Supplementary file 2 — Description of Additional Supplementary Files [file 41467_2020_14367_MOESM2_ESM.pdf]

Supplementary Data 1: Summary of the mutation, expression, and pathway/network data used by each of the seven analysis methods.

Supplementary Data 2: PID-C genes. List of 87 genes identified by a majority (≥ 4/7) of pathway and network methods on GS-C data.

Supplementary Data 3: PID-N genes. List of 93 genes identified by a majority (≥ 4/7) of pathway and network methods on GS-N data or with non-coding value-added (NCVA) procedure.

Supplementary Data 4: PID-CN genes. List of 106 genes identified by a majority (≥ 4/7) of pathway and network methods on GS-CN data.

Supplementary Data 5: PID-N genes without NCVA. List of 62 genes identified by a majority (≥ 4/7) of pathway and network methods on GS-N data. No NCVA genes were added.

Supplementary Data 6: Pairwise similarity (Jaccard index) of the coding results for each pair of methods on coding gene scores (GS-C).

Supplementary Data 7: Pairwise similarity (Jaccard index) of methods based on the union of the results on non-coding gene scores (GS-N) and the non-coding-value-added results.

Supplementary Data 8: Pairwise similarity (Jaccard index) of methods runs using the combined coding-and-non-coding gene scores (GS-CN).

Supplementary Data 9: PID-C neighborhoods. Neighborhood scores (Fisher's method on coding gene scores of network neighbors) of PID-C genes either without or with central PID-C gene.

Supplementary Data 10: PID-N neighborhoods. Neighborhood scores (Fisher's method on non-coding gene scores of network neighbors) of PID-C genes either without or with central PID-C gene.

Supplementary Data 11: Expression changes for core promoter mutations in PID-N genes within tissue type. Expression changes for samples with core promoter mutations in PID-C genes for tissue types with three or more core promoter mutations in gene. Wilcoxon rank-sum test evaluates expression changes, and Benjamini Hochberg procedure used to correct for multiple testing. Expression changes with FDR < 0.1 are highlighted in green and expression changes with 0.1 ≤ FDR < 0.5 are highlighted in yellow.

Supplementary Data 12: Expression changes for core promoter mutations in PID-N genes across tissue types. Expression changes for samples with core promoter mutations in PID-C genes across tissue types. Wilcoxon rank-sum test evaluates expression changes, and Benjamini Hochberg procedure used to correct for multiple testing. Expression changes with FDR < 0.1 are highlighted in green and expression changes with 0.1 ≤ FDR < 0.5 are highlighted in yellow.

Supplementary Data 13: Expression changes for 5' UTR mutations in PID-N genes within tissue type. Expression changes for samples with 5' UTR mutations in PID-C genes for tissue types with three or more 5' UTR mutations in gene. Wilcoxon rank-sum test evaluates expression changes, and Benjamini Hochberg procedure used to correct for multiple testing. Expression changes with FDR < 0.1 are highlighted in green and expression changes with 0.1 ≤ FDR < 0.5 are highlighted in yellow.

Supplementary Data 14: Expression changes for 5' UTR mutations in PID-N genes across tissue types. Expression changes for samples with 5' UTR mutations in PID-C genes across tissue types. Wilcoxon rank-sum test evaluates expression changes, and Benjamini-Hochberg procedure used to correct for multiple testing. Expression changes with FDR < 0.1 are highlighted in green and expression changes with 0.1 ≤ FDR < 0.5 are highlighted in yellow.

Supplementary Data 15: Expression changes for 3' UTR mutations in PID-N genes within tissue type. Expression changes for samples with 3' UTR mutations in PID-C genes for tissue types with three or more 3' UTR mutations in gene. Wilcoxon rank-sum test evaluates expression changes, and Benjamini Hochberg procedure used to correct for multiple testing. Expression changes with FDR < 0.1 are highlighted in green and expression changes with 0.1 ≤ FDR < 0.5 are highlighted in yellow.

Supplementary Data 16: Expression changes for 3' UTR mutations in PID-N genes across tissue types. Expression changes for samples with 3' UTR mutations in PID-C genes across tissue types. Wilcoxon rank-sum test evaluates expression changes, and Benjamini-Hochberg procedure used to correct for multiple testing. Expression changes with FDR < 0.1 are highlighted in green and expression changes with 0.1 ≤ FDR < 0.5 are highlighted in yellow.

Supplementary Data 17: Expression changes for coding mutations in PID-C genes within tissue type. Expression changes for samples with coding mutations in PID-C genes for tissue types with three or more coding mutations in gene. Wilcoxon rank-sum test evaluates expression changes, and Benjamini Hochberg procedure used to correct for multiple testing. Expression changes with FDR < 0.1 are highlighted in green and expression changes with 0.1 ≤ FDR < 0.5 are highlighted in yellow.

Supplementary Data 18: Expression changes for coding mutations in PID-C genes across tissue types. Expression changes for samples with coding mutations in PID-C genes across tissue types. Wilcoxon rank-sum test evaluates expression changes, and Benjamini Hochberg procedure used to correct for multiple testing. Expression changes with FDR < 0.1 are highlighted in green and expression changes with 0.1 ≤ FDR < 0.5 are highlighted in yellow.

Supplementary Data 19: Pathway annotations for PID-C genes. Pathway annotations from GO processes and Reactome pathways for PID-C genes with g:Profiler pathway analysis.

Supplementary Data 20: Pathway annotations for PID-N genes. Pathway annotations from GO processes and Reactome pathways for PID-N genes using g:Profiler pathway analysis.

Supplementary Data 21: Pathway annotations for the union of PID-C and PID-N genes. Pathway annotations from GO processes and Reactome pathways for the union of PID-C and PID-N genes using g:Profiler pathway analysis.

Supplementary Data 22: g:Profiler pathway overlap modules. Pathway modules from g:Profiler overlap map.

Supplementary Data 23: PID-C gene list. Coding driver scores, number and tissue types of mutations on coding regions of the genome, and contributing methods are identified for each PID-C gene.

Supplementary Data 24: PID-N gene list. Non-coding (core promoter, 5' UTR, 3' UTR, enhancer) driver scores, number and tissue types of mutations of somatic mutations on non-coding regions

Supplementary Data 25: Splicing GSEA gene sets. Curated gene sets used in each of the GSEA analyses of splicing factor mutations.

Supplementary Data 26: Splicing GSEA mutant wildtype counts. Counts of mutant and wildtype samples in each of the GSEA analyses of splicing factor mutations.
